# A comparative study on normal and obese mice indicates that the secretome of mesenchymal stromal cells is influenced by tissue environment and physiopathological conditions

**DOI:** 10.1186/s12964-020-00614-w

**Published:** 2020-07-29

**Authors:** Serife Ayaz-Guner, Nicola Alessio, Mustafa B. Acar, Domenico Aprile, Servet Özcan, Giovanni Di Bernardo, Gianfranco Peluso, Umberto Galderisi

**Affiliations:** 1 Department of Molecular Biology and Genetics, Faculty of Life and Natural Science, Abdullah Gül University, Kayseri, Turkey; 2 Department of Experimental Medicine, Luigi Vanvitelli Campania University, Naples, Italy; 3 Genome and Stem Cell Center (GENKOK), Erciyes University, Kayseri, Turkey; 4 Department of Biology, Faculty of Sciences; Erciyes University, Kayseri, Turkey; 5 Research Institute on Ecosystems (IRET); CNR, Naples, Italy; 6 Sbarro Institute for Cancer Research and Molecular Medicine, Center for Biotechnology, Temple University, 1900 N. 12th St, Philadelphia, PA 19107-6799 USA

**Keywords:** Obesity, Mesenchymal stromal cells, Secretome

## Abstract

**Background:**

The term mesenchymal stromal cells (MSCs) designates an assorted cell population comprised of stem cells, progenitor cells, fibroblasts, and stromal cells. MSCs contribute to the homeostatic maintenance of many organs through paracrine and long-distance signaling.

Tissue environment, in both physiological and pathological conditions, may affect the intercellular communication of MSCs.

**Methods:**

We performed a secretome analysis of MSCs isolated from subcutaneous adipose tissue (sWAT) and visceral adipose tissue (vWAT), and from bone marrow (BM), of normal and obese mice.

**Results:**

The MSCs isolated from tissues of healthy mice share a common core of released factors: components of cytoskeletal and extracellular structures; regulators of basic cellular functions, such as protein synthesis and degradation; modulators of endoplasmic reticulum stress; and counteracting oxidative stress. It can be hypothesized that MSC secretome beneficially affects target cells by the horizontal transfer of many released factors. Each type of MSC may exert specific signaling functions, which could be determined by looking at the many factors that are exclusively released from every MSC type.

The vWAT-MSCs release factors that play a role in detoxification activity in response to toxic substances and drugs. The sWAT-MSC secretome contains proteins involved in in chondrogenesis, osteogenesis, and angiogenesis. Analysis of BM-MSC secretome revealed that these cells exert a signaling function by remodeling extracellular matrix structures, such as those containing glycosaminoglycans. Obesity status profoundly modified the secretome content of MSCs, impairing the above-described activity and promoting the release of inflammatory factors.

**Conclusion:**

We demonstrated that the content of MSC secretomes depends on tissue microenvironment and that pathological condition may profoundly alter its composition.

**Video abstract**

## Background

*Mesenchymal stromal cells* (MSCs) are an heterogeneous cell population comprised of stem cells, progenitor cells, fibroblasts, and stromal cells. MSCs reside in the stromal component of several tissues and organs, including bone marrow, cord blood, dental pulp, and adipose tissue. Stem cells present in MSCs can be differentiated into chondrocytes, osteocytes, adipocytes, and other mesodermal cell types. MSCs contribute to the homeostatic maintenance of many organs through paracrine and long-distance signaling [1]. For this reason, MSCs and their products are under scrutiny in numerous clinical trials, to treat several human diseases [2, 3].

MSCs within different tissues are exposed to peculiar microenvironments that impact their phenotypes and functions, with specific modulations of cell proliferation, differentiation, self-renewal, and survival. Many investigations have focused on the biology of bone marrow-derived (BM) and white adipose tissue-derived (WAT) MSCs, since these tissue sources are the most used for isolating MSCs that are employed in cell therapy. Furthermore, BM and WAT resident MSCs play a key role in organismal physiopathology, given the wide distribution of these tissues within the body [1]. Some studies have shown that BM-MSCs and WAT-MSCs differ in their transcriptional profiles, surface antigen expressions, differentiation potentials, and biological functions, such as their effects on cancer cells [4–7]. Pathological conditions may alter the microenvironment surrounding MSCs a d impair their functions. Some findings have demonstrated that MSC dysfunctions are associated with several diseases, including diabetes, lupus, psoriasis, rheumatoid arthritis, and metabolic syndrome [8, 9].

Tissue environment, in both physiological and pathological conditions, may significantly affect the intercellular communication of MSCs, which occurs through cell-cell contact, soluble factors (growth factors, hormones, cytokines, metabolites, etc.), and the release of extracellular vesicles (EVs). These vesicles range from 30 to 1000 nm and carry many bioactive molecules, surface receptors, and genetic information (DNA, diverse types of RNAs). EVs interact with target cells, which may be close to or distant from the originating cell. EV signaling can occur either through ligand-receptor interaction at the target cell’s surface or through the fusion of vesicles with cell plasma membranes (endocytosis) [10].

MSC tissue homeostasis and regeneration activities occur mainly through the release of soluble factors and EVs that enter blood circulation and can reach target tissues throughout the body. This MSC property is also the basis of their therapeutic effectiveness in cell therapy treatments [1, 11].

In this context, the aim of our study was to evaluate how physiological and pathological changes in the MSC microenvironment affect secretome composition and hence MSC functions. We decided to perform an unbiased analysis of the entire proteome content of MSC secretome. Specifically, we collected and analyzed the secretomes of MSCs obtained from subcutaneous and visceral WAT, as well as from bone marrow, of normal and obese mice. Obesity leads to WAT dysfunction, promoting chronic inflammation and cardiovascular and metabolic pathologies. The body contains subcutaneous, visceral, and bone marrow fat depots, whose distribution and functions are altered in obese individuals [12, 13]. We chose obesity for the pathological condition, since this disease greatly affects the fat depots where MSCs reside.

## Material and methods

### Animals

Six C57BL/6 inbred male mice age 3 weeks were purchased from Charles River (Wilmington, MA, USA). As the study involved animals, it was approved by the Italian Ministry of Health (n. 317-2016PR), and mice were handled in compliance with the protocols approved by the Animal Care and Use Committee of University Campania Luigi Vanvitelli. After arrival, the mice were divided into two groups and were fed either a high-fat diet (HFD) (Research Diets, New Brunswick, NJ, USA) or a normal diet (ND) for 10 weeks. At the end of this treatment, the mice were euthanized, and tissue samples were harvested for the experiments laid out below.

The high-fat diet consisted of 60% fat from lard, 20% carbohydrates, and 20% protein (total 5.21 kcal/g), whereas the normal diet consisted of 10% fat, 70% carbohydrates, and 20% protein (total 3.82 kcal/g). Food intake and body weight were measured once a week until the end of the experiments.

### Glucose measurement

At the end of high and normal fat treatments, blood glucose levels were determined in fasting mice by tail bleeding, using a Contour blood glucose meter (Ascensia Diabetes Care, Parsippany, NJ, USA) according to the manufacturer’s instructions.

### MSC isolation and cultivation

We harvested MSCs from the bone marrow of mice’s femurs and tibias by inserting a 21-gauge needle into the shaft of the bone and flushing it with alpha-MEM. The cells from one mouse were plated onto two 100-mm dishes with alpha-MEM containing 15% FBS. After 48 h, we discarded the nonadherent cells and washed the adherent cells with PBS (phosphate-buffered saline) 1X. We then incubated the cells for 7 to 10 days in a proliferating medium in order to reach confluence (P0). Cells were grown until passage 3 for secretome harvest.

We collected MSCs from 500 mg of subcutaneous WAT (sWAT) surrounding the hips of the mice and from 1 g of visceral WAT (vWAT) obtained from the abdomen area. Tissues were digested for 1 h at 37 °C in a DMEM solution containing collagenase type II (1 mg/ml). Samples were filtered through cell strainers (70 μm mesh), centrifuged, and washed three times with PBS 1X. Cells were plated onto 100-mm dishes with alpha-MEM containing 15% FBS. We then incubated the cells for 7 days in a proliferating medium in order to reach confluence (P0). Cells were grown until passage 3 for secretome harvest.

### Secretome harvest

MSC cultures (80% confluent) were washed extensively with PBS and then transferred to a chemically-defined, serum-free culture medium for overnight incubation. Then, the conditioned media containing MSC secretion were collected and used for liquid chromatography-mass spectrometry (LC-MS) analysis.

### Secretome preparation for LC-MS/MS analysis

Five mL of secretomes were collected from culture dishes without disturbing the attached cells, at which point culture debris were removed by centrifugation at 10.000 g. Supernatants were used for StartaClean beads protein pooling. Dried beads were mixed with 1x Laemmli gel loading buffer and run on a gradient gel 4–15% SDS-PAGE (Criterion TGX Stain Free Precast Gels, BIO-RAD, USA). Electrophoresis was carried out at 100 V. After electrophoresis, gels were stained with Coomassie, and the gel lanes of interest were excised for in-gel digestion.

After digestion, the peptides were eluted from the gel matrix by immersion of the reaction tube in an ultrasonic bath for 5 min, with sequential elution of 0.4% formic acid in 3% ACN, 0.4% formic acid in 50% ACN, and 0.4% formic acid in 100% ACN. The supernatant containing the peptides was centrifuged, transferred to low binding tubes, and desalted with ZipTip C18 (Millipore, Merck). After that, the extracted peptides were dried and stored at − 80 °C until the LC-MS/MS analysis.

### LC-MS/MS analysis

Tandem mass spectrometric analysis was carried out using an AB SCIEX TripleTOF 5600+ instrument (AB SCIEX, Redwood City, CA, USA) coupled to an Eksigent expert nano-LC 400 system (AB SCIEX). MS and MS/MS data were acquired using Analyst® TF v.1.6 (AB SCIEX).

Mass spectrometry data was analyzed using ProteinPilot 4.5 Beta (AB SCIEX) for peptide identification.

### Gene ontology analysis

The proteins expressed in the secretomes were analyzed using the PANTHER (Protein ANalysis THrough Evolutionary Relationships - http://www.pantherdb.org) software. In PANTHER, the protein classification was performed according to the ontology terms: cellular component, protein class, molecular function, biological processes, and pathway. For the PANTHER analysis, we used the statistics overrepresentation (default setting), comparing classifications of multiple clusters of lists to a reference list in order to statistically identify overrepresentation of PANTHER ontologies. The selected *p*-value was set at 0.05. We followed the developers’ instructions for running a PANTHER analysis [14].

### Pathway analysis

Differentially-expressed proteins were imported into Reactome software for detailed pathway identification [15, 16]. The Reactome Knowledgebase (https://reactome.org) provides molecular details of cellular processes as an ordered network of molecular transformations in a single consistent data model. We submitted LC/MS data as a single column of identifiers (UniProt IDs), and the software mapped them to pathways. Over-representation and pathway-topology analyses were conducted. Over-representation analysis is based on statistical hypergeometric distribution, and it evaluates whether certain specific Reactome pathways are enriched in the submitted data. This analysis produced a probability score, which was then corrected for false discovery rate (FDR) using the Benjamani-Hochberg method. The FDR was set at *p* < 0.05.

## Results

We fed two groups of mice (three mice per group) with a high-fat diet (HFD) or a normal diet (ND) for 10 weeks. In the ND group, the average weight increased from 21.0 ± 2.5 g to 26 ± 2.3 g, while in the HFD group, the weight started from 20.6 ± 2.3 g rose to 44.2 ± 4.5 g. The HFD treatment induced hyperglycemia (170 ± 6.5 mg/dL in ND versus 280 ± 15.5 mg/dL in HFD), determined by blood glucose measurement.

We then isolated and cultivated MSCs from BM, visceral WAT (vWAT), and subcutaneous WAT (sWAT) of both normal and obese mice to evaluate their in vitro properties. We verified by flow cytometry that MSCs expressed the surface antigens CD105, CD90, and CD73 and were able to differentiate into adipocytes, chondrocytes, and osteocytes (Additional file 1).

We grew MSCs in vitro until passage 3 and then collected secretomes for the analysis of their proteome content.

We had three biological replicates for each type of MSC culture (BM-MSC, sWAT-MSC, and vWAT-MSC secretomes); globally, we collected 18 secretome samples—9 from HFD-treated mice and 9 from ND-treated mice. We performed LC-MS/MS analyses on peptides from the tryptic digestion of secretome samples. Each sample had two technical replicates (Additional file 2).

We employed high-resolution MS in a search of the Protein Metrics database, wherein several hundred proteins were identified in all the experimental conditions (Additional file 2). We merged data from technical and biological replicates through a Venn diagram analysis, thereby obtaining a list of proteins expressed in the various experimental conditions (Table 1).

**Table 1:** Number of proteins per secretome

	HFD	ND
**BM-MSCs**	444	487
**sWAT -MSCs**	510	573
**vWAT-MSCs**	381	257

### Gene ontology (GO) analysis in samples from ND-treated mice

GO implements an enrichment analysis of ontology terms in the proteomic profile of interest. An ontology term consists of a set of proteins with relations that operate between them. We matched our experimental data to reference ontology terms by using PANTHER’s GO enrichment analysis, and we identified the ontology terms that were overrepresented in our datasets compared to a reference mouse protein set.

We focused our GO analysis on ontological terms belonging to the following GO domains (hierarchical biological clusters): cellular components, protein classes, molecular functions, biological processes, and pathways. For each experimental condition, we identified dozens of ontologies (Additional file 3). We then performed a Venn diagram analysis to combine the data of all experimental conditions in order to find both the specific and the common ontologies among the secretomes of BM-MSCs, vWAT-MSCs, and sWAT-MSCs from ND-treated mice. The most representative ontologies are depicted in Tables 1 and 2.

**Table 2:** .

	Common GO among vWAT sWAT BM	GO vWAT specific	GO sWAT specific	GO BM specific
**COMMON AND SPECIFIC GENE ONTOLOGY (GO) ENTITIES IN ND SAMPLES**				
**GO CELLULAR COMPONENT**	**Arp2/3 protein complex**		**Chaperonin containing T-complex**	**Chaperonin containing T-complex**
	**Actin filament**			**Lysosome**
	**Extracellular space (ECM)**			
	**Collagen containing ECM**			
	**Cytosolic small ribosomal subunit**			
	**Cytosolic large ribosomal subunit**			
	**Proteasome core complex**			
**GO PROTEIN CLASS**	**Non-motor actin binding protein**	**Peroxidase**	**Growth factor**	**Cytokine**
	**Actin and actin related protein**	**Reductase**	**Metalloprotease**	**Metalloprotease**
	**Extracellular matrix structural protein**		**Nucleic acid binding protein**	**Serine protease**
	**Oxidoreductase**		**Transporter**	**Nucleic acid binding protein**
	**Ribosomal protein**			**Transporter**
	**Protease inhibitor**			
	**Hsp90 family chaperone**			
	**G protein coupled receptor**			
	**Calmodulin-related**			
	**Zinc finger transcription factor**			
	**Immunoglobulins**			
**GO MOLECULAR FUNCTION**	**Extracellular matrix binding**	**Protein**	**Growth factor activity**	**Protein serine/threonine kinase activity**
	**Integrin binding**	**serine/threonine kinase activity**	**Carboxypeptidase activity**	
	**Structural constituent of ribosome**			
	**Protease binding**			
	**Serine type endopeptidase**			
	**Metalloendopeptidase**			
	**Transition ion metal binding**			
	**ATP binding**			
	**G protein coupled receptor activity**			
	**Transmembrane transporter activity**			
**CHANGES IN HFD SAMPLES**				
**GO CELLULAR COMPONENT**				**Ribosomal protein ABSENT**
**GO PROTEIN CLASS**		**Peroxidase ABSENT**		
**GO MOLECULAR FUNCTION**		**Transition ion metal binding ABSENT**	**Transmembrane transporter activity ABSENT**	**Structural constituent of ribosome ABSENT**
			**Growth factor activity ABSENT**	

**Fig. 1:**
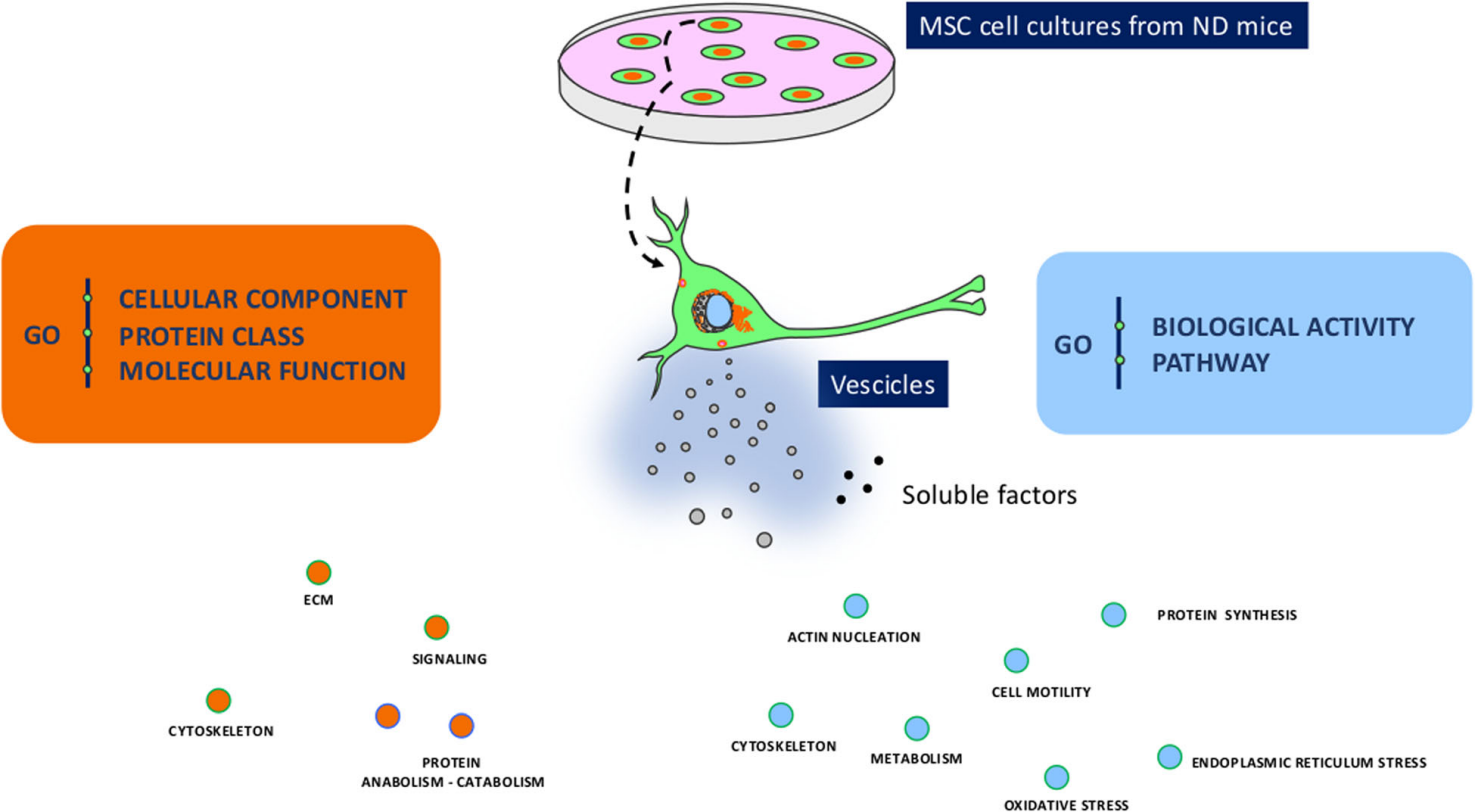
Main GO ontologies identified in secretome samples. The pictures depict some common ontologies identified by PANTHER analysis in the secretomes of vWAT-MSCs, sWAT-MSCs, and BM-MSCs. In orange are factors classified according GO Biological activity and GO Pathway, while in blue are classified according GO Cellular component, GO Protein class and GO molecular function

Cellular component, protein class, and molecular function GO analyses demonstrated that proteins belonging to cytoskeleton and extracellular matrix (ECM) structures, those belonging to signaling networks, those belonging to the oxy-redox class, and those involved in protein anabolism/catabolism were overrepresented in the secretomes of MSCs from ND-treated mice (Table 2, Fig. 1). Of note, in the secretomes of BM- and sWAT-MSCs, we also identified proteins belonging to chaperone, growth factor, and cytokine families (Table 2, Fig. 1). Biological process and pathway GO analyses showed that proteins involved in actin nucleation, cell motility, protein synthesis, endoplasmic reticulum stress, oxidative stress, and metabolism were overrepresented in the secretomes of MSCs from ND-treated mice (Table 3, Fig. 1). In addition, the vWAT-MSCs secreted several proteins involved in responding to toxic substances and drugs, as well as proteins that play a role in the small molecule metabolic process. The secretomes of sWAT-MSCs and BM-MSCs contained proteins that regulate leukocyte and granulocyte chemotaxis, as well as negative regulators of cell death (Table 3). In BM-MSC secretome, many proteins were seen that are involved in metabolism (carbohydrate, pyruvate, and lipid metabolic processes) (Table 3). Of great interest, sWAT-MSCs released many factors that modulate proliferation and differentiation of several cell types involved in angiogenesis, chondrogenesis, and osteogenesis (Table 3).

**Table 3:** .

	Common GO among vWAT sWAT BM	GO vWAT specific	GO sWAT specific	GO BM specific
**COMMON AND SPECIFIC GENE ONTOLOGY ENTITIES IN ND SAMPLES**				
**GO BIOLOGICAL PROCESS**	**Arp2/3 complex-mediated actin nucleation**	**Carbohydrate metabolic process**	**Response to toxic substance**	**Carbohydrate metabolic process**
	**Actin filament organization**	**Response to toxic substance**	**Response to inorganic substance**	**Cellular lipid metabolic process**
	**Cell motility**	**Response to inorganic substance**	**Small molecule metabolic process**	**Regulation of leukocyte chemotaxis**
	**Collagen fibril organization**	**Drug metabolic process**	**Glutathione metabolic process**	**Regulation of leukocyte migration**
	**Ribosomal large unit assembly**	**Small molecule metabolic process**	**Cellular lipid metabolic process**	**Granulocyte chemotaxis**
	**Translation**	**Tissue remodeling**	**Amino acid metabolism**	**Negative regulation of cell death**
	**Regulation of peptidase activity**		**Response to hypoxia**	**Chemokine-mediated signaling pathway**
	**Response to endoplasmic reticulum stress**		**Tissue remodeling**	
	**Chaperone-mediated protein folding**		**Angiogenesis**	
	**Proteasome-mediated ubiquitin dependent protein catabolic process**		**Endothelial cell proliferation**	
			**Positive regulation of epithelial cell proliferation**	
	**Response to oxidative stress**		**Regulation of leukocyte chemotaxis**	
	**Glucose 6-phosphate metabolic process**		**Regulation of leukocyte migration**	
	**Glycolytic process**		**Granulocyte chemotaxis**	
	**ATP metabolic process**		**Bone morphogenesis**	
			**Chondrocyte differentiation**	
			**Regulation of cellular response to growth factor stimulus**	
			**Negative regulation of cell death**	
**GO PATHWAYS**	**Cytoskeletal regulation by Rho GTPase**	**FGF signaling pathway**	**FGF signaling pathway**	**Pyruvate metabolism**
	**Integrin signaling pathway**	**EGF receptor signaling pathway**	**EGF receptor signaling pathway**	**Plasminogen activating cascade**
	**Glycolysis**			
	**Pentose phosphate pathway**			
	**De novo purine biosynthesis**			
	**Blood coagulation**			
	**Inflammation mediated by chemokine and cytokine signaling pathway**			
**CHANGES IN HFD SAMPLES**				
**GO BIOLOGICAL PROCESS**		**Drug metabolic process ABSENT**	**Cellular lipid metabolic process ABSENT**	**Ribosomal large subunit assembly ABSENT**
		**Small molecule metabolic process ABSENT**	**Endothelial cell proliferation ABSENT**	
			**Response to Interleukin-1**	**Translation ABSENT**
				**Carbohydrate metabolic process ABSENT**
				**Cellular lipid metabolic process ABSENT**
				**ATP metabolic process ABSENT**
				**Bone morphogenesis**
				**Chondrocyte differentiation**
				**Tissue morphogenesis**
				**ERK1 and ERK2 cascade**
				**Response to Interleukin-1**
**GO PATHWAYS**		**Blood coagulation ABSENT**	**CCKR signaling map**	**Pyruvate metabolism ABSENT**
		**FGF signaling pathway ABSENT**	**Plasminogen activating cascade**	**De novo purine biosynthesis ABSENT**
		**EGF receptor signaling pathway ABSENT**	**Gonadotropin-releasing hormone receptor pathway**	

### Gene ontology (GO) analysis in samples from HFD-treated mice

We evaluated how obesity affected the GO ontologies of MSC-secreted proteins. Importantly, in samples from obese mice, we observed the absence of some GO terms found in normal mice and the presence of a few new ontologies (Tables 2 and 3). Specifically, in vWAT samples from HFD-treated mice, proteins involved in response to drugs and small molecule metabolism were absent. Additionally, factors involved in oxy-redox or transition metal ion binding activities were not found (Tables 2 and 3). In the sWAT-MSC secretome, several proteins associated with lipid metabolism and some growth factors were no longer present in samples from obese mice (Tables 2 and 3). Two new GO ontology groups were present in the sWAT-MSC secretome obtained from HFD-treated mice: response to interleukin-1 (IL-1) and cholecystokinin (CCK)B/gastrin receptors (CCKR) signaling map. IL-1 pathway is intensely activated during inflammation and may contribute to chronic inflammation, associated with obesity [17]. The gastrin cholecystokinin B receptors trigger signaling pathways, which influence the expression of genes that are involved in cell survival, angiogenesis, and invasion [18].

In the secretomes of BM-MSCs obtained from obese mice, several ontologies associated with metabolism and protein synthesis were absent. Of note, in these samples, we also observed GO terms associated with IL-1 pathway (Tables 2 and 3). BM-MSCs from obese mice released several proteins that modulate chondrogenesis and osteogenesis; these factors were absent in the secretome from normal mice.

### Reactome analysis in samples from ND-treated mice

Experimental data analysis with GO gives a general view of the most significant ontology groups present in the datasets, but it cannot directly define the most important proteins in the analyzed proteomes. However, this can be achieved with Reactome analysis. In this analysis, any event that modifies the state of a biological molecule is defined as a ‘reaction’. Specifically, binding, activation, translocation, degradation, and all other biochemical events involving a catalyst are considered reactions [15, 16]. The assumption is that a given protein group found in the experimental data reflects a key functional importance for the phenotype(s) under analysis if all the proteins are part of the same Reactome pathway.

**Fig. 2:**
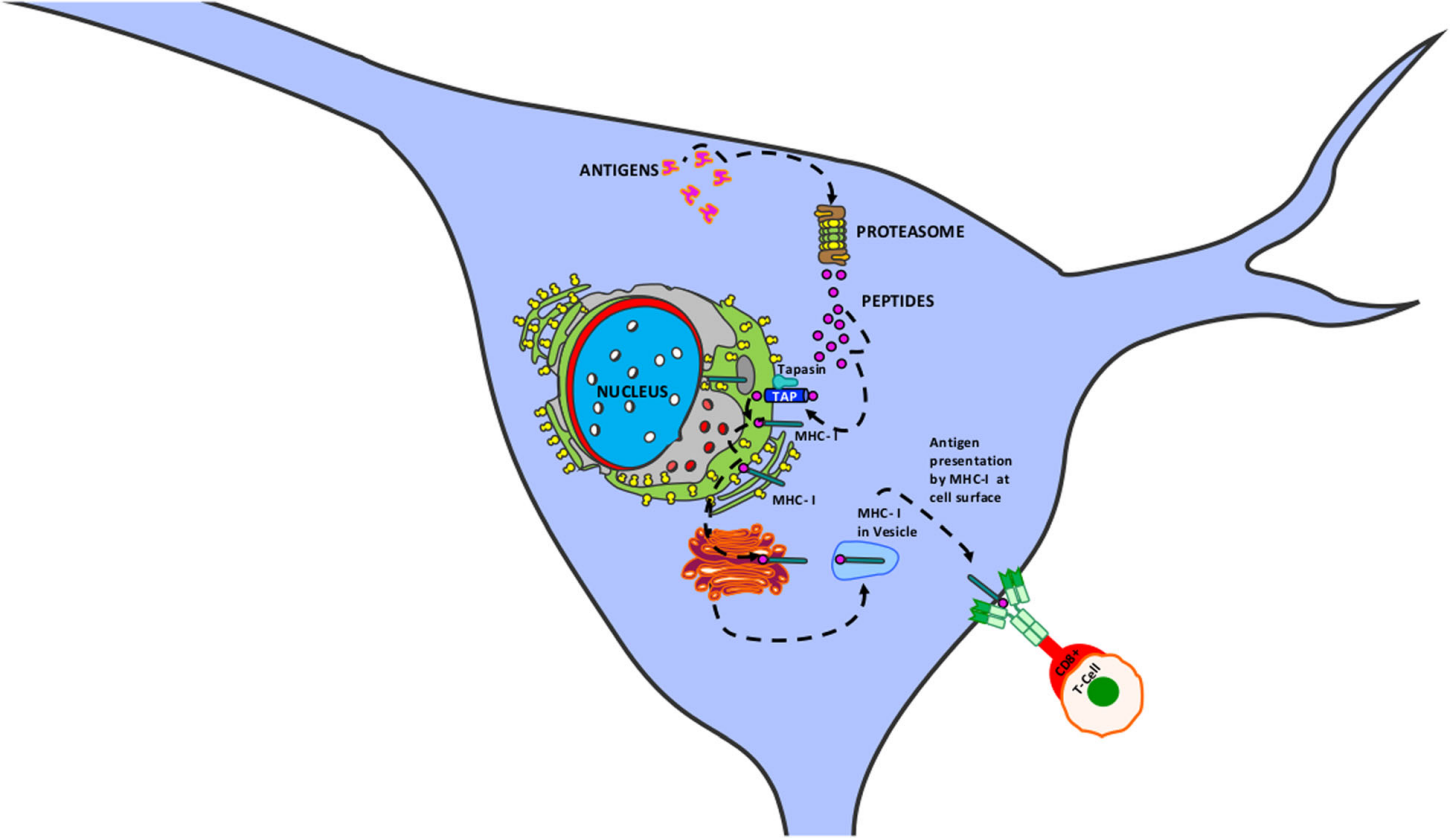
Cross-presentation of soluble exogenous antigens (endosomes) pathway. The pathway consists of three main networks: antigen processing—cross-presentation; antigen presentation—folding, assembly, and peptide loading of class I MHC; and antigen processing—ubiquitination and proteasome degradation. During the presentation process, antigen proteins are degraded into peptides by proteases in the proteasome. Peptides are then delivered to the endoplasmic reticulum (ER) through heat shock proteins and the transporter associated with antigen processing (TAP), which transport peptides from cytosol into the ER lumen. Several ER chaperones (calnexin, tapasin, calreticulin, etc.) contribute to MHC-I assembly. Peptides are loaded into the MHC-I peptide binding groove; this complex exits the ER and is transported to Golgi and then to the cell surface by exocytic vesicles. Naïve T cells (CD8+) are activated by interacting with peptide-MHC-I complexes. Additional file 4 reports the proteins of vWAT-MSC, sWAT-MSC, and BM-MSC secretomes that belong to the above-indicated networks

The secretome contents of vWAT-MSCs, sWAT-MSCs, and BM-MSCs from ND-treated mice were assigned to 27, 13, and 17 Reactome pathways, respectively (Table 4). Three pathways were in common among the secretomes: cross presentation of soluble antigens (endosomes); post-translational protein phosphorylation; and SCF-beta-TrCP mediated degradation of Emi1. These three networks are associated with the identified GO terms that are present in all secretomes coming from MSCs of ND-treated mice. For example, within the ontologies associated with endoplasmic reticulum stress (Table 3, Fig. 1), the most significant network is the endosome pathway leading to antigen processing (Table 4). In vWAT-MSC secretomes, the Reactome analysis identified 14 proteins out of 51 in the reference list. In sWAT-MSC and BM-MSC secretomes, 17 and 14 proteins belonging to this network, respectively, were present (Fig. 2; Additional file 4).

**Table 4:** .

**vWAT ND REACTOME PATHS (27)**	
**APC/C:Cdc20 mediated degradation of Securin**	
**APC/C:Cdh1 mediated degradation of Cdc20 and other APC/C:Cdh1 targeted proteins in late mitosis/early G1**	
**Autodegradation of Cdh1 by Cdh1:APC/C**	
**CDK-mediated phosphorylation and removal of Cdc6**	
**CDT1 association with the CDC6:ORC:origin complex**	
**Chk1/Chk2(Cds1) mediated inactivation of Cyclin B:Cdk1 complex**	
**Cross-presentation of soluble exogenous antigens (endosomes)**	
**Defective CFTR causes cystic fibrosis**	
**Degradation of AXIN**	
**Eukaryotic Translation Termination**	
**Formation of a pool of free 40S subunits**	
**Hh mutants abrogate ligand secretion**	
**Hh mutants that don’t undergo autocatalytic processing are degraded by ERAD**	
**HSF1 activation**	
**L13a-mediated translational silencing of Ceruloplasmin expression**	
**Mycobacterium tubercolisis biological processes**	
**Orc1 removal from chromatin**	
**Peptide chain elongation**	
**Platelet degranulation**	
**Post-translational protein phosphorylation**	
**Regulation of activated PAK-2p34 by proteasome mediated degradation**	
**Regulation of ornithine decarboxylase (ODC)**	
**Regulation of RAS by GAPs**	
**Response to elevated platelet cytosolic Ca2+**	
**SCF-beta-TrCP mediated degradation of Emi1**	
**Selenocysteine synthesis**	
**Vif-mediated degradation of APOBEC3G**	
**sWAT ND REACTOME PATHS (13)**	
**Anchoring fibril formation**	
**Assembly of collagen fibrils and other multimeric structures**	
**Cross-presentation of soluble exogenous antigens (endosomes)**	
**Crosslinking of collagen fibrils**	
**Elastic fibre formation**	
**Hh mutants that don’t undergo autocatalytic processing are degraded by ERAD**	
**Laminin interactions**	
**Mycobacterium tuberculosis biological processes**	
**Post-translational protein phosphorylation**	
**Prefoldin mediated transfer of substrate to CCT/TriC**	
**Regulation of ornithine decarboxylase (ODC)**	
**SCF-beta-TrCP mediated degradation of Emi1**	
**Vif-mediated degradation of APOBEC3G**	
**BM ND REACT PATHS (17)**	
**Assembly of collagen fibrils and other multimeric structures**	
**Collagen chain trimerization**	
**Collagen degradation**	
**Cross-presentation of soluble exogenous antigens (endosomes)**	
**Crosslinking of collagen fibrils**	
**Defective B4GALT1 causes B4GALT1-CDG (CDG-2d)**	
**Defective CHST6 causes MCDC1**	
**Defective ST3GAL3 causes MCT12 and EIEE15**	
**Diseases associated with glycosaminoglycan metabolism**	
**ECM proteoglycans**	
**Elastic fibre formation**	
**Laminin interactions**	
**Molecules associated with elastic fibres**	
**Platelet degranulation**	
**Post-translational protein phosphorylation**	
**Regulation of Insulin-like Growth Factor (IGF) transport and uptake by Insulin-like Growth Factor Binding Proteins (IGFBPs)**	
**SCF-beta-TrCP mediated degradation of Emi1**

The most significant network in protein anabolism/catabolism ontologies (Fig. 1) is the post-translational protein phosphorylation (Table 4; Additional file 4).

The Reactome pathway “SCF-beta-TrCP mediated degradation of Emi1” indicates Emi1 protein destruction in early mitosis by the SCFβTrCP/Slimb Ubiquitin Ligase, which activates the anaphase-promoting complex to allow cell cycle progression [19]. This network cannot be assigned to a single GO entity; rather it refers to several ontologies associated with cell signaling (Tables 2 and 3).

**Fig. 3:**
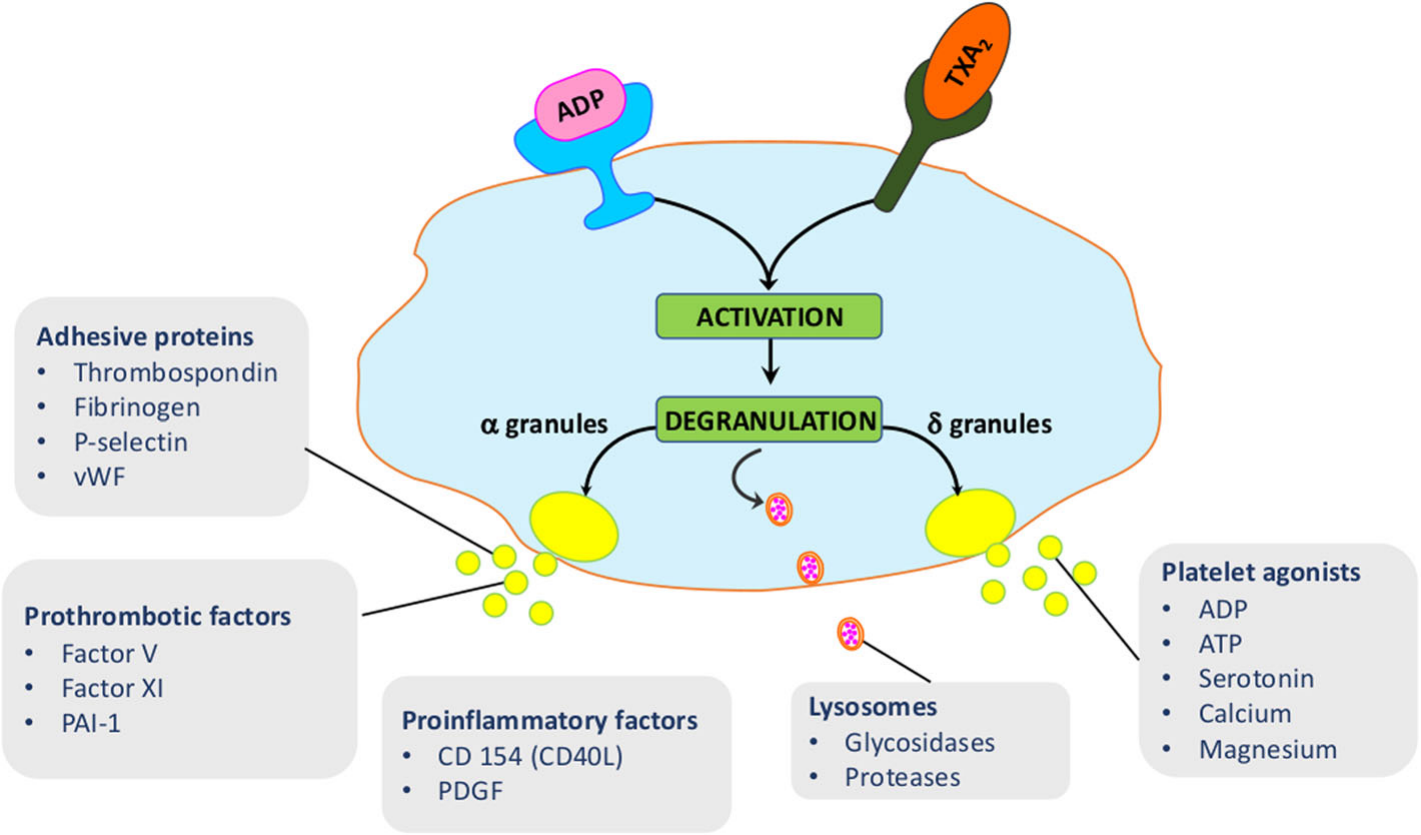
Platelet degranulation pathway. This pathway consists of several networks: ABCC4 accumulation of dense granule contents; exocytosis of platelet dense granule content; surface deployment of platelet dense granule membrane components; exocytosis of platelet alpha granule contents; surface deployment of platelet alpha granule membrane components; release of platelet cytosolic components; release of platelet secretory granule components; and exocytosis of proactivator polypeptide. Platelets are activated following the interaction between ligands, such as ADP and TXA2 (Tromboxane A2), and their cognate receptors on the platelet cell surface. After activation, platelets release the contents of three distinct types of preformed intracellular vesicles. Dense granules (δ granules) contain platelet agonists, and lysosomes contain glycosidases and acid proteases. The α granules release adhesive proteins, prothrombotic factors, and pro-inflammatory factors. Additional file 4 reports the proteins of vWAT-MSC, sWAT-MSC, and BM-MSC secretomes that belong to these networks

Several Reactome pathways specifically identified in the vWAT-MSC secretome can be associated with protein anabolism/catabolism GO terms, including: formation of a pool of free 40S subunits; peptide chain elongation; and eukaryotic translation termination (Table 4). Selenocysteine synthesis appears to be the most significant pathway that could be associated with the oxy-redox GO terms. Many other pathways involved in cell cycle regulation were found in the vWAT-MSC secretome besides the SCF-beta-TrCP mediated degradation of Emi1 that was in common with other secretomes. Notably, Reactome analysis identified a pathway named platelet degranulation, which can refer to several GO terms listed in Tables 3 and 4 (Fig. 3). Activated platelets rapidly release the contents of distinct types of preformed intracellular vesicles (granules), such as dense granules, alpha granules, and lysosomes. Dense granule components contribute to hemostasis and coagulation, but they also play a role in cancer metastasis. Alpha granules contain cytokines, growth factors, regulators of the coagulation cascade, pro- and anti-inflammatory factors, and other bioactive factors that contribute to a number of disease processes [20].

**Fig. 4:**
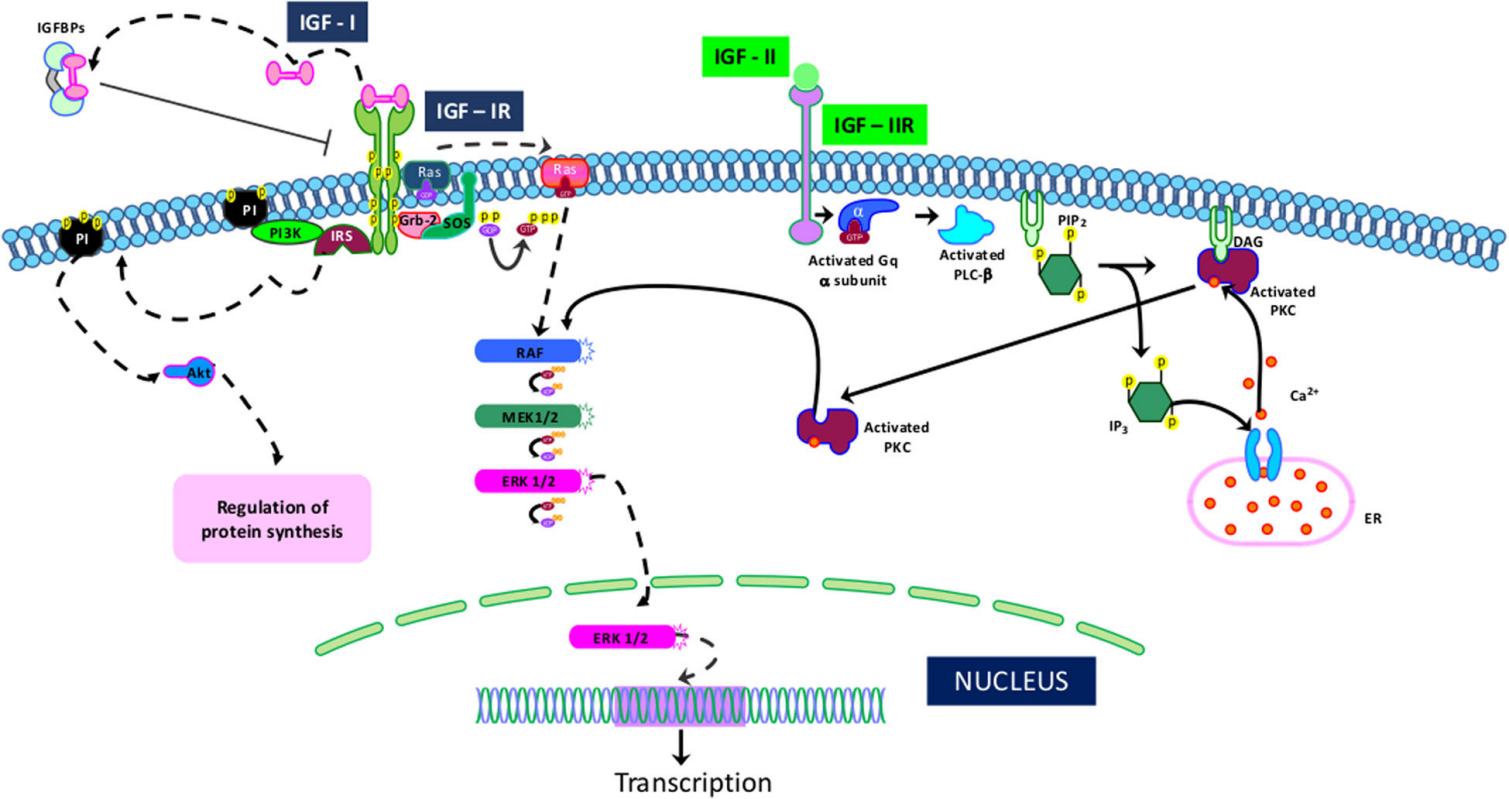
Regulation of insulin-like growth factor (IGF) transport and uptake by insulin-like growth factor binding proteins (IGFBPs) pathway. The pathway consists of several networks: IGFBP1 binds with IGF, forming IGF:IGFBP1; IGFBP2 binds with IGF, forming IGF:IGFBP2; IGFBP4 binds with IGF, forming IGF:IGFBP4; IGFBP6 binds with IGF, forming IGF:IGFBP6; PAAP-A proteolyzes IGF:IGFBP4; FAM20C phosphorylates FAM20C substrates. IGF-I binds to its receptor (IGF-IR), which leads to IRS/PI3K phosphorylation and subsequent downstream activation of AKT. Alternatively, IGF-I can activate Shc/Grb-2/Sos phosphorylation and complex formation. This event promotes the activation of the Ras/Raf/MEK/MAPK cascade. IGF-I binds to the hybrid IGF-IR/IR receptor, activating PI3K and MAPK pathways. The IGF-II/IGF-IIR complex can activate an alternative pathway that is associated with the G protein and phospholipase C (PLC). The result of the PLC activity is the production of diacylglycerol (DAG) and inositol triphosphate (IP3), which in turn can activate protein kinase C (PKC) and the RAF/MEK/ERK pathway. IGF-I also binds with IGF-IIR, and IGF-II also binds with IGF-IR. It not well-known which pathways are activated following these interactions. IGFBP proteins bind with either IGF-I or IGF-II and modulate their activities

In the sWAT-MSC secretome, several pathways are associated with cytoskeleton and ECM GO ontologies, including: crosslinking of collagen fibrils; laminin interactions; and anchoring fibril formation (Table 4). Additionally, the BM-MSC cells release factors that belong to pathways related to cytoskeleton and ECM organization (Table 4). In addition, the secretome of BM-MSCs contain proteins belonging to the platelet degranulation pathway, as reported for the vWAT-MSC secretome. Regulation of the insulin-like growth factor pathway is a peculiar network identified in the secretome of BM-MSCs (Fig. 4).

### Reactome analysis in samples from HFD-treated mice

The secretome contents of vWAT-MSCs, sWAT-MSCs, and BM-MSCs obtained from obese mice were assigned to 25, 15 and 20 Reactome pathways, respectively (Table 5). Most of the Reactome pathways found in the corresponding secretomes obtained from normal mice were also present in samples from obese mice. In particular, the three pathways that were in common among the secretomes of sWAT-MSCs, vWAT-MSCs, and BM-MSCs in normal mice were also identified in obese mice.

**Table 5:** .

**vWAT HFD REACTOME PATHS (25)**	
**Anchoring fibril formation**	
**APC/C:Cdc20 mediated degradation of Securin**	
**APC/C:Cdh1 mediated degradation of Cdc20 and other APC/C:Cdh1 targeted proteins in late mitosis/early G1**	
**Assembly of collagen fibrils and other multimeric structures**	
**Autodegradation of Cdh1 by Cdh1:APC/C**	
**CDK-mediated phosphorylation and removal of Cdc6**	
**CDT1 association with the CDC6:ORC:origin complex**	
**Chk1/Chk2(Cds1) mediated inactivation of Cyclin B:Cdk1 complex**	
**Collagen chain trimerization**	
**Collagen degradation**	
**Cross-presentation of soluble exogenous antigens (endosomes)**	
**Crosslinking of collagen fibrils**	
**Defective CFTR causes cystic fibrosis**	
**Degradation of AXIN**	
**Hh mutants abrogate ligand secretion**	
**Hh mutants that don’t undergo autocatalytic processing are degraded by ERAD**	
**HSF1 activation**	
**Orc1 removal from chromatin**	
**Platelet degranulation**	
**Post-translational protein phosphorylation**	
**Regulation of activated PAK-2p34 by proteasome mediated degradation**	
**Regulation of ornithine decarboxylase (ODC)**	
**Regulation of RAS by GAPs**	
**SCF-beta-TrCP mediated degradation of Emi1**	
**Vif-mediated degradation of APOBEC3G**	
**sWAT HFD REACT PATHS (15)**	
**Assembly of collagen fibrils and other multimeric structures**	
**Autodegradation of Cdh1 by Cdh1:APC/C**	
**Cross-presentation of soluble exogenous antigens (endosomes)**	
**Crosslinking of collagen fibrils**	
**Defective B4GALT1 causes B4GALT1-CDG (CDG-2d)**	
**Elastic fibre formation**	
**Hh mutants abrogate ligand secretion**	
**Hh mutants that don’t undergo autocatalytic processing are degraded by ERAD**	
**Laminin interactions**	
**Mycobacterium tuberculosis biological processes**	
**Platelet degranulation**	
**Post-translational protein phosphorylation**	
**Regulation of ornithine decarboxylase (ODC)**	
**SCF-beta-TrCP mediated degradation of Emi1**	
**Vif-mediated degradation of APOBEC3G**	
**BM HFD REACT PATHS (20)**	
**Anchoring fibril formation**	
**Assembly of collagen fibrils and other multimeric structures**	
**Collagen biosynthesis and modifying enzymes**	
**Collagen chain trimerization**	
**Collagen degradation**	
**Collagen formation**	
**Cross-presentation of soluble exogenous antigens (endosomes)**	
**Crosslinking of collagen fibrils**	
**Defective B4GALT1 causes B4GALT1-CDG (CDG-2d)**	
**Degradation of the extracellular matrix**	
**ECM proteoglycans**	
**Elastic fibre formation**	
**HSF1 activation**	
**Laminin interactions**	
**Molecules associated with elastic fibres**	
**NCAM1 interactions**	
**Neutrophil degranulation**	
**Platelet degranulation**	
**Post-translational protein phosphorylation**	
**Regulation of Insulin-like Growth Factor (IGF) transport and uptake by Insulin-like Growth Factor Binding Proteins (IGFBPs)**	

A deep examination into the secretome of vWAT-MSCs shows that the selenocysteine synthesis pathway present in samples from normal mice was absent in samples coming from obese mice.

The sWAT-MSCs of HFD-treated samples secreted proteins belonging to the platelet degranulation pathway that were absent in the corresponding ND-treated samples. Thus, in obese mice, all three types of MSCs release factors activating platelets. This overproduction of platelet-activating factors may contribute to the chronic inflammation associated with obesity. The release of proteins belonging to the neutrophil degranulation pathway from BM-MSCs, seen in obese mice, could further exacerbate inflammation.

### Identification of proteins specifically expressed in samples from ND- and HFD-treated mice

**Fig. 5:**
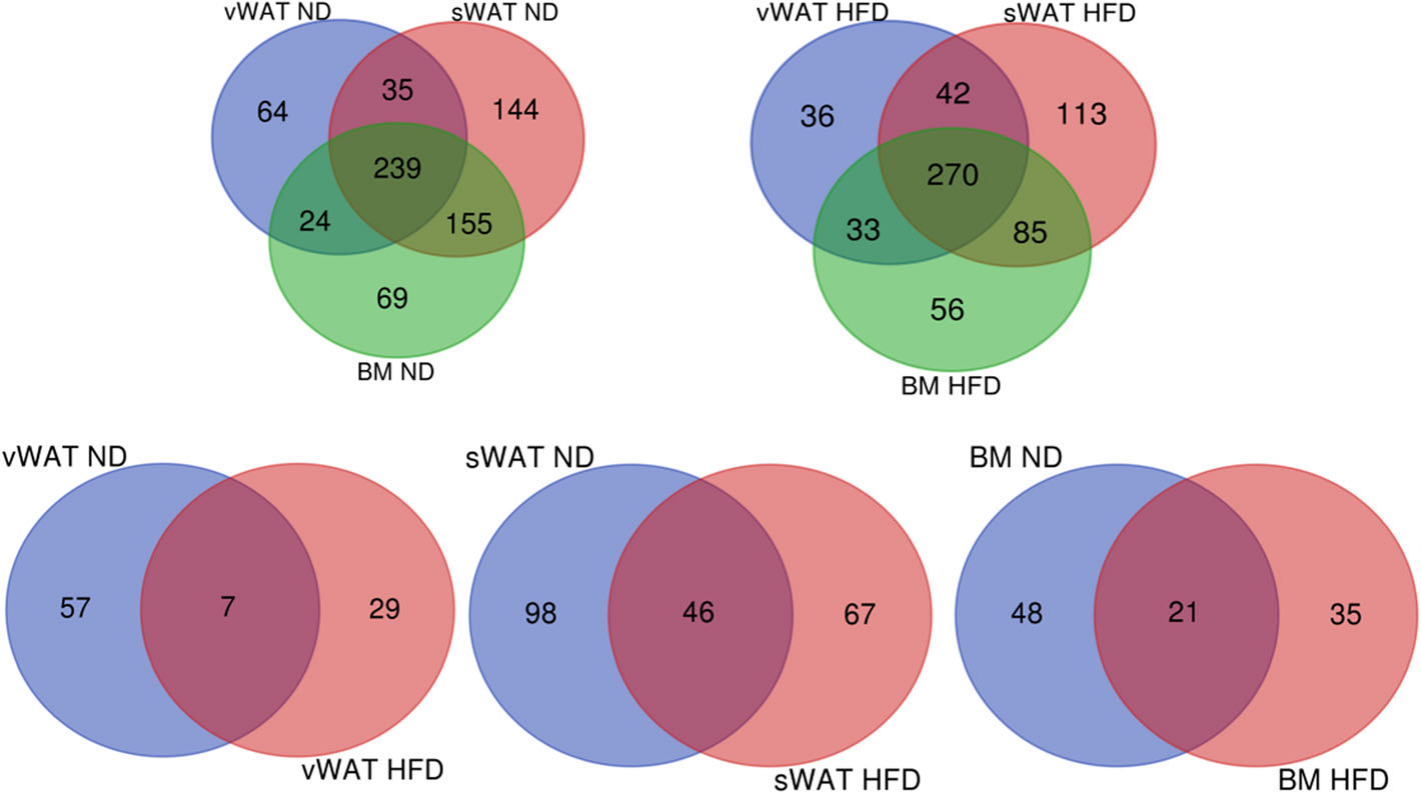
Venn diagram analysis. Top left: Venn diagram showing common and specific proteins among secretomes obtained from vWAT-MSCs, sWAT-MSCs, and BM-MSCs isolated from samples taken from normal mice (ND). Top right: Venn diagram showing common and specific proteins among secretomes obtained from vWAT-MSCs, sWAT-MSCs, and BM-MSCs isolated from samples taken from obese mice (HFD). Bottom: Venn diagram comparison of vWAT-MSCs from normal mice with vWAT-MSCs from obese mice. The same procedure was applied for sWAT-MSCs and BM-MSCs. Numbers indicate common and specific proteins for every comparison

We performed a Venn diagram analysis to identify common and specific proteins in the different environmental and pathological conditions. The MSCs isolated from different tissues in normal mice released only partially overlapping factors (Fig. 5). Specifically, 64 proteins were found exclusively in the secretome of vWAT-MSCs, while 144 and 69 were exclusively present in the secretomes of sWAT-MSCs and BM-MSCs, respectively. Additionally, in obese mice, MSCs from different sources shared only part of their secretomes.

We then compared the proteins exclusively present in vWAT-MSCs between normal and obese mice. The pathological condition greatly affected the secretome composition: only 7 proteins were found both in normal and obese secretome samples, while 57 were exclusively present in the secretome of normal samples and 29 were exclusively present in the secretome of obese samples (Fig. 5). The secretomes of sWAT-MSCs and BM-MSCs were also greatly modified by obesity (Fig. 5).

We then focused on proteins exclusively released by vWAT-MSCs, sWAT-MSCs, or BM-MSCs isolated from samples taken from normal and obese mice (Table 6, Additional file 2). The most significant proteins released exclusively from the vWAT-MSCs of normal mice belong to several networks. For example, Ptgr1 and Csfr1 are part of the modulation of the immune system. Ptgr1 is involved in a key step of the metabolic inactivation of leukotriene B4, whose levels increase during inflammation [21]. Csfr1 signaling is fundamental to the differentiation and survival of the mononuclear phagocyte system and macrophages [22]. Catalase and GSR are components of the redox activity network. Catalase protects cells from the toxic effects of hydrogen peroxide, and GSR maintains high levels of reduced glutathione in the cell cytoplasm [23]. BLVRA, CRAT, Nampt, and Sorcin are part of metabolic networks. BLVRA reduces biliverdin (a byproduct of heme catabolism) to bilirubin, which is an antioxidant and has a role in lowering risk of metabolic syndromes. Obese individuals with high visceral adiposity have low bilirubin levels [24]. CRAT—i.e., carnitine acetyltransferase—is a mitochondrial enzyme that catalyzes the interconversion of acetylcarnitine and acetyl-CoA. Studies have shown that it is a positive regulator of total body glucose tolerance and muscle activity, and its activity is inhibited by obesity and lipid stress [25]. Nampt, also called Visfatin, is an adipokine that influences metabolic homeostasis and whose level increases significantly with obesity, due to increased body mass index [26]. Sorcin is a protein involved in maintaining calcium within the endoplasmic reticulum by inhibiting ryanodine receptor activity; its impairment is associated with metabolic syndromes [27].

**Table 6:** Proteins specifically expressed in the indicated secretomes

	**vWAT ND**	**sWAT ND**	**BM ND**
**Growth factor activity and differentiation**		Ang	Gmfb
		Angptl4	Manf
		Fstl3	
		Pgf	
**Modulation of immune system**	Ptgr1	Cd81	Ccl9
	Csfr1		Ifi30
**Redox activity**	Catalase	Glc	
	Gsr	Prdx5	
		Prdx6	
**Metabolism**	Blvra		Aldh1a3
	Crat		Aldh1a2
	Nampt		Me1
	Sorcin		
**ECM**			Cemip
			Itih3
			Vcan
	**vWAT HFD**	**sWAT HFD**	**BM HFD**
**Growth factor activity and differentiation**	Hdgf	Igf2	Fstl3
		Ostf1	
		Tgm2	
**Modulation of immune system**			Cfh
**Redox activity**			
**Metabolism**		Fdps	Lipa
		Pla1a	
**Miscellaneous/pathological conditions**		Hyou1	
		Mt1	

All of these proteins have a positive role in several aspects of organismal homeostasis, and their presence is lost in the secretomes of vWAT-MSCs in samples taken from obese mice.

The most significant proteins released exclusively from sWAT-MSCs from normal mice belong to the following networks: redox activity, modulation of immune system, growth factor activities, and differentiation network (Table 6). Ang, Fstl3, Pgf, and Angptl4 are part of this last network. Ang (angiogenin), Pgf (placenta growth factor), and Angptl4 (angiopoietin-like 4) could be the key players in angiogenesis of the sWAT-MSC secretome, as evidenced in the Reactome analysis [28–30]. Fstl3 (follistatin) may be one the most important components of the sWAT-MSC secretome, since it conducts key functions in regulation of fat accumulation and insulin sensitivity, modulation of hematopoiesis, and control of bone formation [31–33]. The GCL, Prdx5, and Prdx6 proteins are part of the redox activity network. GCL (glutamate cysteine ligase) is an enzyme of the cellular glutathione biosynthetic pathway; together with Prdx5 and Prdx6, it is fundamental in controlling reactive oxygen levels and in counteracting oxidative stress [34, 35].

The tissue development and differentiation functions—along with the anti-oxidant activity present in the secretome of sWAT-MSCs from normal mice—are absent in samples from obese mice. Instead, in the secretomes from obese mice, factors are present whose activities are strictly associated with negative outputs of obesity. For example, Ostf1 (osteoclast stimulation factor 1) can promote osteoporosis, Tgm2 is involved in negative artery remodeling, and IGF2 can contribute to senescence of MSCs [36–38].

BM-MSCs release factors involved in growth and differentiation of neural cells, such as glia maturation factor-β (GMFB) and mesencephalic astrocyte-derived neurotrophic factor (MANF) [39, 40]. These cells also release proteins that regulate energy metabolism, such as Me1 (malic enzyme), Aldh1a2, and Aldh1a3 (aldehyde dehydrogenase) [41, 42]. BM-MSCs also secrete many proteins associated with glycosaminoglycan formation and degradation. All these factors were absent in the secretomes of cells isolated from tissue samples of obese mice.

## Discussion

Release of signaling factors is a key activity of MSCs; for this reason, several studies have analyzed their secretome content. Nevertheless, a systemic investigation of the microenvironment’s influence on MSC secretome composition, either in physiological or pathological conditions, is still lacking. Indeed, the microenvironment—with structural and trophic support, topographical information, and pathophysiological cues—can greatly affect cell behavior [43].

The literature contains findings that address specific aspects of MSC secretome. For example, some researchers have analyzed the cytokines released by adipose tissue-derived and bone marrow-derived MSCs, while others have focused their attention on secreted neuroregulators or on factors involved in hepatic lineage development and differentiation [8, 44, 45]. Some researchers have analyzed the contents of extracellular vesicles released by adipose tissue-derived MSCs [8, 46]. Others have performed secretome analysis with low-resolution techniques, which has not provided exhaustive information [47, 48].

Our study aimed to fill certain gaps in secretome analysis of MSCs by performing a comparison analysis of the impact of physiological (tissue of origin) and pathological (obesity) cues. The choice to analyze MSCs from visceral WAT and subcutaneous WAT was not trivial, since these tissues have distinct metabolic and inflammatory functions [49]. Indeed, the vast majority of studies have analyzed the biological properties of MSCs derived from subcutaneous fat, and only a few have analyzed those derived from visceral fat. However, the latter fat depot contributes remarkably to the negative effects of obesity on human health. In this context, we evaluated the effect of obesity on MSC secretory activity, since this condition affects the size, function, and inflammatory state of adipose tissues and modifies the stem cell niches present in these tissues [12, 49].

Our study clearly showed that tissue microenvironment significantly affects secretome composition of MSCs and hence their signaling activity. First, it should be emphasized that most of the proteins found in the MSC secretomes lack the signal peptide present at the N-terminus of many proteins that are destined for the secretory pathway [50]. This suggests that many of them are not freely circulating within extracellular fluids but are rather encapsulated in EVs.

The MSCs isolated from bone marrow, visceral WAT, and subcutaneous WAT of healthy mice share a common core of released factors: components of cytoskeletal and extracellular structures; regulators of basic cellular functions, such as protein synthesis and degradation; modulators of endoplasmic reticulum stress; and counteracting oxidative stress. It can be hypothesized that MSC secretome beneficially affects target cells by contributing to their main biological activities through EV-mediated horizontal transfer of structural cellular components and of regulators of cellular anabolism and catabolism processes.

However, each type of MSCs may exert specific signaling functions, which could be determined by looking at the many factors that are exclusively released from each MSC type. The vWAT-MSCs release factors that have a peculiar role in detoxification activity in response to toxic substances and drugs as well as many factors involved in the synthesis of selenocysteine, which is present in the active sites of several enzymes (glutathione peroxidase, thioredoxin reductase, and iodothyronine deiodinase) that participate in oxidation-reduction reactions [51]. These functions of MSCs within vWAT could have a potential role in preserving the tissue’s healthiness, since many findings have demonstrated that adipose tissue is a potential site of reactive oxygen species (ROS) and toxin accumulation [52]. Obesity status almost completely negated the release of these adipose tissue “protective factors”.

The sWAT-MSC secretome contains many proteins involved in tissue development and differentiation, such as factors participating in chondrogenesis, osteogenesis, and angiogenesis. This last process seems to be highly supported by sWAT-MSC signaling, since these cells released angiogenin, placenta growth factor, and Angptl4, which have a prominent role in angiogenic processes [28–30]. At the same time, we find only a few factors involved in adipogenesis [53]. This may indicate that their levels are below the limit of detection for our technique and/or that MSCs are not the main producers of such factors. It is well-known that MSCs play a key role in immunomodulation; our study demonstrated that the sWAT-MSCs release many proteins involved in chemotaxis and migration of immune cells. Obesity negatively impacted sWAT-MSC secretome: the anti-oxidant (GCL, Prdx5, Prdx6) and tissue development (Ang, Angptl4, Fstl3, Pgf) activities were lost, while factors promoting osteoporosis and negative vessel remodeling were acquired.

The analysis of BM-MSC secretome in tissue from normal mice revealed that these cells exert a signaling function through a very active remodeling of extracellular matrix structures; factors (CEMIP, Itih3, VCAN) that reshape (build/degrade) glycosaminoglycans were only present in their secretome. These cells also seemed to play a role in metabolism control by releasing dozen of factors, some of them found exclusively in their secretome (Aldh1a3, Aldh1a2, Me1). Of great interest, in BM-MSC secretome includes factors that promote growth and differentiation of glia and neurons, such as glia maturation factor-β (GMFB) and mesencephalic astrocyte-derived neurotrophic factor (MANF) [39, 40]. The presence of such factors matches the hypothesized crosstalk between osteogenic and neurogenic niches, which relies on partial overlap of the molecular and secretome profiles as well as on the intimate relationship with vessels [54]. At the same time, the trophic effects of GMFB and MANF apply not only to neurons and glia but also to other cell types [40, 55].

How does a pathological modification of tissue microenvironment affect the secretome composition of MSCs? Obesity, with its associated chronic inflammation status, profoundly modifies the secretome content of MSCs. Obesity status almost completely negated the release of factors that promote tissue renewal and homeostasis. In obese mice, vWAT-MSCs lost their particular detoxification and ROS scavenging functions. Anti-oxidant activities were also impaired in the secretomes of sWAT-MSCs and BM-MSCs. This occurrence could negatively impact the health of obese individuals. High-caloric intake produces an excess of energy substrates for cellular metabolic pathways, which in turn increase ROS production that cannot be buffered. In obese individuals, the ROS increment alters cellular functions and may induce cellular senescence, as shown by us and other researchers. Obesity negatively impacted the sWAT-MSC secretome, since its anti-oxidant (GCL, Prdx5, Prdx6) and tissue development (Ang, Angptl4, Fstl3, Pgf) activities were lost, while factors promoting osteoporosis and negative vessel remodeling were acquired. These events were associated with secretion of pro-inflammatory cytokines, associated with the IL-1 signaling pathway and platelet degranulation. The release of inflammatory factors belonging to these pathways was also detected in the BM-MSCs secretome in obese mice, along with cytokines promoting neutrophil degranulation.

## Conclusion

We demonstrated that the content of MSC secretomes depends on tissue microenvironment and that pathological condition may profoundly alter its composition. This study demonstrates that MSCs isolated from different tissues both share common functions and perform unique tasks. This finding may pave the way to better understanding the role of MSCs in tissue renewal and homeostasis. Moreover, it may further contribute to selection of the correct MSC source(s) for clinical purposes. In cell therapy treatments, the choice of adipose tissue-derived MSCs or bone marrow-derived MSCs is not irrelevant and may have profound consequences on the clinical outcomes.

## Supplementary information

**Additional file 1 **Flow cytometry analysis and differentiation of MSCs. Panel A: BM-MSCs, vWAT-MSCs, and sWAT-MSCs were trypsinized; washed with PBS; and incubated with either anti-CD105 FITC (sc-18,893 FITC), anti-CD90 PE (SC-1914 PE), or anti-CD73 PE (sc-398,260). The antibodies were used according to the manufacturer’s procedures (Santa Cruz Biotechnology CA, USA). After 30 min of incubation with the antibodies at room temperature, cells were washed with PBS and resuspended in FACS buffer on a Guava EasyCyte flow cytometer for data acquisition (Merck Millipore MA, USA). We performed data analysis with a standard procedure using EasyCyte software. A minimum of 5000 cells per sample were analyzed and gated for forward scatter (FSC) versus side scatter (SSC) channel signals. In each panel, the histograms show positive cells from obese (blue) and normal (green) mice. ND = Normal Diet-treated mice; HFD = High Fat Diet-treated mice. In red are the negative controls. Panel B: Adipocyte, osteocyte, and chondrocyte differentiation of MSCs obtained from obese and normal mice. The figure shows representative images of Oil Red O (adipocytes), Alizarin Red S (osteocytes), and Alcian blue (chondrocytes) staining for every experimental condition. The method for differentiation is described below. *Adipogenic differentiation.* MSCs were treated for 15 days in a mesenchymal stem cell adipogenic differentiation medium (PT-3004-KT; Lonza, Walkersville, MD, USA). The medium contained insulin (recombinant), dexamethasone, indomethacin, and 3-isobuty-l-methyl-xanthine (IBMX). Lipid droplets were revealed by staining with Oil Red O. *Osteogenic differentiation.* MSCs were treated for 15 days in a mesenchymal stem cell osteogenic differentiation medium (PT-3002-KT; Lonza). The medium contained dexamethasone, ascorbate, and glycerophosphate. Staining with Alizarin Red S revealed calcium deposits in differentiated osteocytes. *Chondrogenic differentiation.* MSCs were seeded as pellets in 96 round-bottom multi-wells and cultured in a chondrogenic medium composed of DMEM, 1% FBS, 50 nM ascorbate-2-phosphate (Sigma-Aldrich, St. Louis, MO, USA), 0.1 mM dexamethasone (Sigma-Aldrich, MO, USA), and 10 ng/mL human transforming growth factor (hTGF)-β1 (PeproTech, London, UK). After 21 days, Alcian blue staining was performed.

**Additional file 2.** List of proteins identified in MSC secretome. “ND HFD tech biol replicates” spreadsheet: The sheet shows the list of proteins found in vWAT-MSCs, sWAT-MSCs, and BM-MSCs isolated from samples taken from ND-treated mice designated as 1, 2, and 3 and from HFD-treated mice designated as 4, 5, and 6. For each biological sample, there were two technical replicates (A, B). Proteins were listed with their UniProt identifiers. “ND HFD common data” spreadsheet: The proteins secreted by vWAT-MSCs isolated from samples taken from mouse 1, 2, and 3 were analyzed with a Venn graph to find common data. The procedure was also performed for sWAT-MSCs and BM-MSCs. The sheet also lists proteins isolated from samples taken from mice 4, 5, and 6, which were analyzed with the same method. “Venn comparison in ND or HFD” spreadsheet: The sheet shows the result of Venn diagram comparison among vWAT-MSCs, sWAT-MSCs, and BM-MSCs coming from ND- and HFD-treated mice. “Venn comparison in ND vs. HFD” spreadsheet: The sheet shows the result of Venn diagram comparison of vWAT-MSCs from ND-treated mice versus vWAT-MSCs from HFD-treated mice. The same procedure was employed for sWAT-MSCs and BM-MSCs.

**Additional file 3.** GO analysis carried out with PANTHER. The list shows ontology terms overrepresented in the secretomes of vWAT-MSCs, sWAT-MSCs, and BM-MSCs taken from ND- and HFD-treated mice. Ontology terms were classified as: cellular components, protein classes, molecular functions, biological processes, and pathways.

**Additional file 4.** Reactome analysis. The report of pathway analysis of proteins present in the secretomes of vWAT-MSCs, sWAT-MSCs, and BM-MSCs isolated from samples taken from ND- and HFD-treated mice.

## Data Availability

The datasets used and/or analysed during the current study are available from the corresponding author on reasonable request.

## Abbreviations

BM: Bone marrow
BM-MSC: Bone marrow mesenchymal stromal cells
ECM: Extracellular matrix
GO: Gene ontology
HFD: High fat diet
LC-MS: Liquid chromatography-mass spectrometry
ND: Normal diet
sWAT: Subcutaneous white adipose tissue
sWAT-MSC: Subcutaneous white adipose tissue-derived mesenchymal stromal cells
vWAT: Visceral white adipose tissue
vWAT-MSC: Visceral white adipose tissue-derived mesenchymal stromal cells
