# Supplementary material for: A comparative study on normal and obese mice indicates that the secretome of mesenchymal stromal cells is influenced by tissue environment and physiopathological conditions
Source: Cell Commun Signal. 2020 Jul 29;18:118. doi: 10.1186/s12964-020-00614-w (PMC7388533; doi:10.1186/s12964-020-00614-w)
Supplement: Supplementary file 2 — Additional file 1 Flow cytometry analysis and differentiation of MSCs. Panel A: BM-MSCs, vWAT-MSCs, and sWAT-MSCs were trypsinized; washed with PBS; and incubated with either anti-CD105 FITC (sc-18,893 FITC), anti-CD90 PE (SC-1914 PE), or anti-CD73 PE (sc-398,260). The antibodies were used according to the manufacturer’s procedures (Santa Cruz Biotechnology CA, USA). After 30 min of incubation with the antibodies at room temperature, cells were washed with PBS and resuspended in FACS buffer on a Guava EasyCyte flow cytometer for data acquisition (Merck Millipore MA, USA). We performed data analysis with a standard procedure using EasyCyte software. A minimum of 5000 cells per sample were analyzed and gated for forward scatter (FSC) versus side scatter (SSC) channel signals. In each panel, the histograms show positive cells from obese (blue) and normal (green) mice. ND = Normal Diet-treated mice; HFD = High Fat Diet-treated mice. In red are the negative controls. Panel B: Adipocyte, osteocyte, and chondrocyte differentiation of MSCs obtained from obese and normal mice. The figure shows representative images of Oil Red O (adipocytes), Alizarin Red S (osteocytes), and Alcian blue (chondrocytes) staining for every experimental condition. The method for differentiation is described below. Adipogenic differentiation. MSCs were treated for 15 days in a mesenchymal stem cell adipogenic differentiation medium (PT-3004-KT; Lonza, Walkersville, MD, USA). The medium contained insulin (recombinant), dexamethasone, indomethacin, and 3-isobuty-l-methyl-xanthine (IBMX). Lipid droplets were revealed by staining with Oil Red O. Osteogenic differentiation. MSCs were treated for 15 days in a mesenchymal stem cell osteogenic differentiation medium (PT-3002-KT; Lonza). The medium contained dexamethasone, ascorbate, and glycerophosphate. Staining with Alizarin Red S revealed calcium deposits in differentiated osteocytes. Chondrogenic differentiation. MSCs were seeded as pellets in 96 roun [file 12964_2020_614_MOESM2_ESM.pdf]

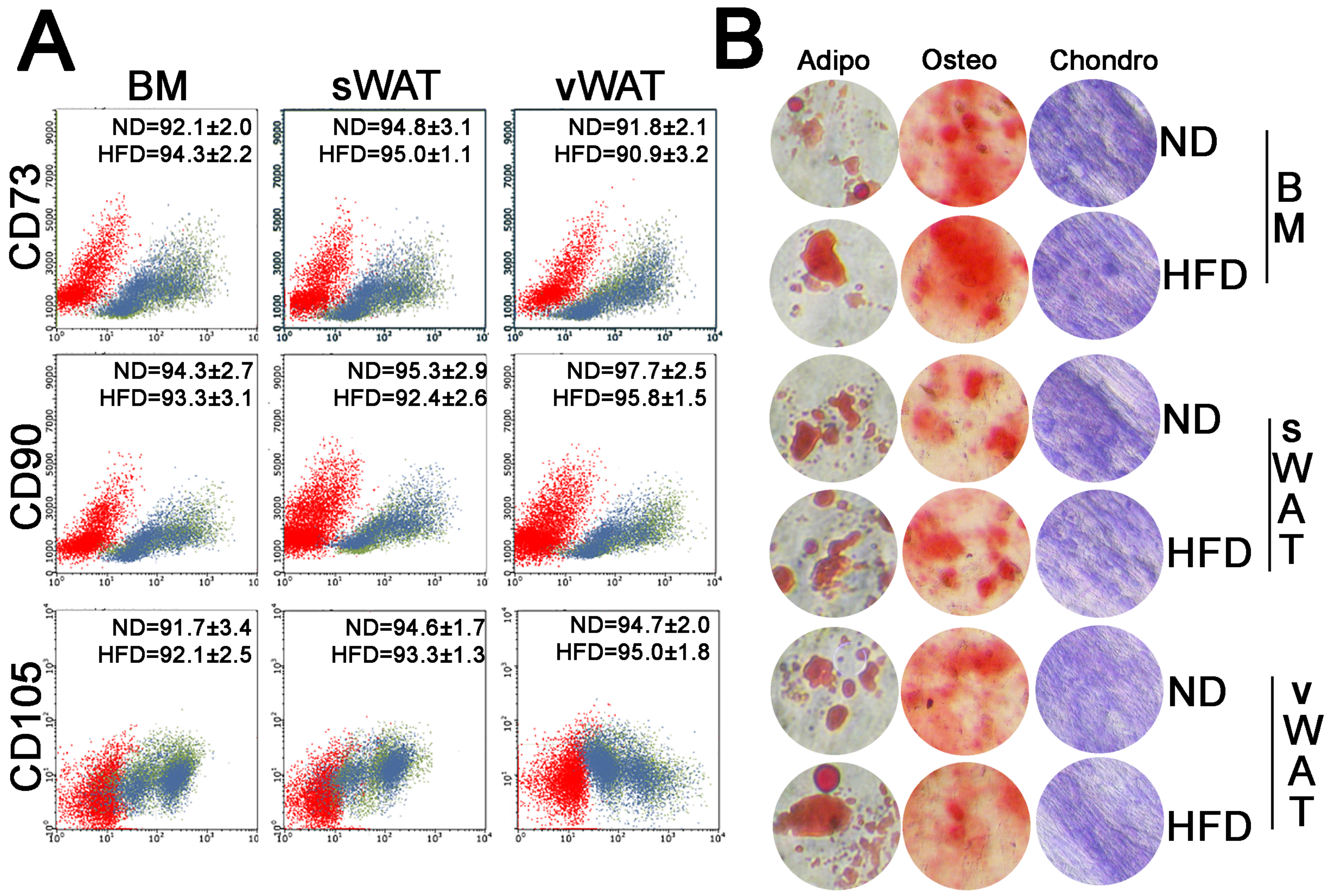
A
BM
sWAT
vWAT
CD73
CD90
CD105
ND=92.1±2.0
HFD=94.3±2.2
ND=94.8±3.1
HFD=95.0±1.1
ND=91.8±2.1
HFD=90.9±3.2
ND=94.3±2.7
HFD=93.3±3.1
ND=95.3±2.9
HFD=92.4±2.6
ND=97.7±2.5
HFD=95.8±1.5
ND=91.7±3.4
HFD=92.1±2.5
ND=94.6±1.7
HFD=93.3±1.3
ND=94.7±2.0
HFD=95.0±1.8
B
Adipo
Osteo
Chondro
ND
HFD
BM
ND
HFD
sWAT
ND
HFD
vWAT
