# Supplementary material for: A comparative study on normal and obese mice indicates that the secretome of mesenchymal stromal cells is influenced by tissue environment and physiopathological conditions
Source: Cell Commun Signal. 2020 Jul 29;18:118. doi: 10.1186/s12964-020-00614-w (PMC7388533; doi:10.1186/s12964-020-00614-w)
Supplement: Supplementary file 5 — Additional file 4. Reactome analysis. The report of pathway analysis of proteins present in the secretomes of vWAT-MSCs, sWAT-MSCs, and BM-MSCs isolated from samples taken from ND- and HFD-treated mice. [file 12964_2020_614_MOESM5_ESM.pdf]

reactome

vWAT-MSC secretome from ND mice
Pathway Analysis Report

Table of Contents

1. Introduction
2. Properties
3. Genome-wide overview
4. Most significant pathways
5. Pathways details
6. Identifiers found
7. Identifiers not found

1. Introduction

Reactome is a curated database of pathways and reactions in human biology. Reactions can be considered as pathway 'steps'. Reactome defines a 'reaction' as any event in biology that changes the state of a biological molecule. Binding, activation, translocation, degradation and classical biochemical events involving a catalyst are all reactions. Information in the database is authored by expert biologists, entered and maintained by Reactome's team of curators and editorial staff. Reactome content frequently cross-references other resources e.g. NCBI, Ensembl, UniProt, KEGG (Gene and Compound), ChEBI, PubMed and GO. Orthologous reactions inferred from annotation for Homo sapiens are available for 17 non-human species including mouse, rat, chicken, puffer fish, worm, fly, yeast, rice, and Arabidopsis. Pathways are represented by simple diagrams following an SBGN-like format.

Reactome's annotated data describe reactions possible if all annotated proteins and small molecules were present and active simultaneously in a cell. By overlaying an experimental dataset on these annotations, a user can perform a pathway over-representation analysis. By overlaying quantitative expression data or time series, a user can visualize the extent of change in affected pathways and its progression. A binomial test is used to calculate the probability shown for each result, and the p-values are corrected for the multiple testing (Benjamini–Hochberg procedure) that arises from evaluating the submitted list of identifiers against every pathway.

To learn more about our Pathway Analysis, please have a look at our relevant publications:

Fabregat A, Sidiropoulos K, Garapati P, Gillespie M, Hausmann K, Haw R, … D'Eustachio P (2016). The reactome pathway knowledgebase. Nucleic Acids Research, 44(D1), D481–D487. https://doi.org/10.1093/nar/gkv1351.

Fabregat A, Sidiropoulos K, Viteri G, Forner O, Marin-Garcia P, Arnau V, … Hermjakob H (2017). Reactome pathway analysis: a high-performance in-memory approach. BMC Bioinformatics, 18.

2. Properties

- This is an **overrepresentation** analysis: A statistical (hypergeometric distribution) test that determines whether certain Reactome pathways are over-represented (enriched) in the submitted data. It answers the question 'Does my list contain more proteins for pathway X than would be expected by chance?' This test produces a probability score, which is corrected for false discovery rate using the Benjamani-Hochberg method.
- 320 out of 339 identifiers in the sample were found in Reactome, where 9055 pathways were hit by at least one of them.
- IntAct interactors were included to increase the analysis background. This greatly increases the size of Reactome pathways, which maximises the chances of matching your submitted identifiers to the expanded pathway, but will include interactors that have not undergone manual curation by Reactome and may include interactors that have no biological significance, or unexplained relevance.
- This report is filtered to show only results for species 'Mus musculus' and resource 'all resources'.
- The unique ID for this analysis (token) is MjAyMDAzMjQxMjA1MDZfMzE3OA%3D%3D. This ID is valid for at least 7 days in Reactome's server. Use it to access Reactome services with your data.

3. Genome-wide overview

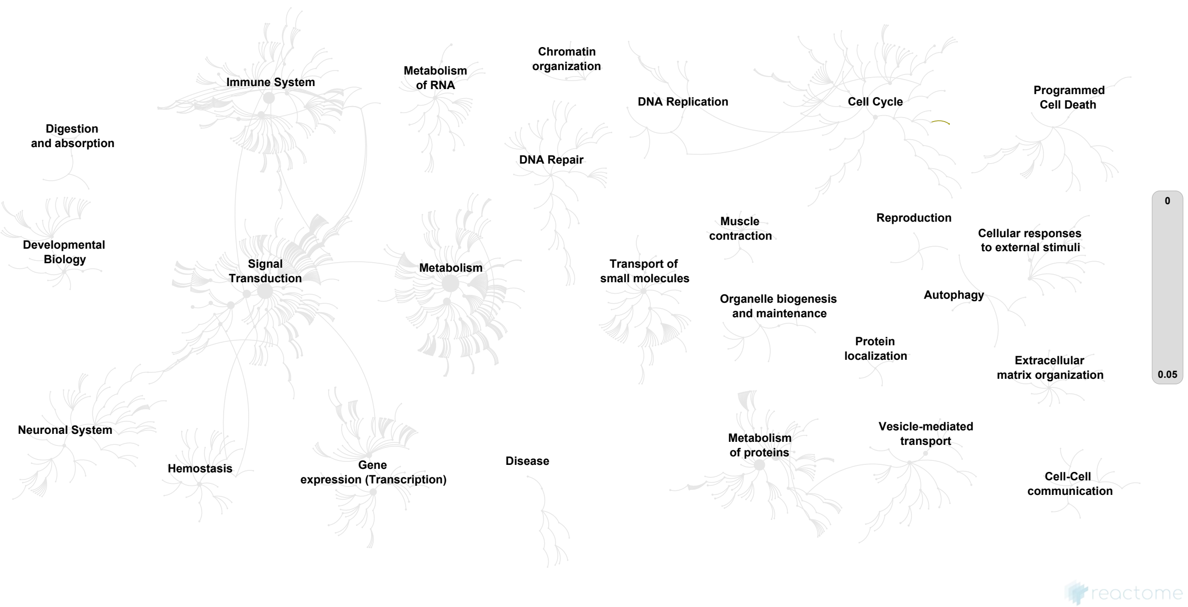

This figure shows a genome-wide overview of the results of your pathway analysis. Reactome pathways are arranged in a hierarchy. The center of each of the circular "bursts" is the root of one top-level pathway, for example "DNA Repair". Each step away from the center represents the next level lower in the pathway hierarchy. The color code denotes over-representation of that pathway in your input dataset. Light grey signifies pathways which are not significantly over-represented.

4. Most significant pathways

The following table shows the 25 most relevant pathways sorted by p-value.

Pathway name	Entities				Reactions	
	found	ratio	p-value	FDR*	found	ratio
Chk1/Chk2(Cds1) mediated inactivation of Cyclin B:Cdk1 complex	6 / 14	0.001	0.044	1	1 / 4	5.13e-04
HSF1 activation	5 / 11	9.09e-04	0.052	1	5 / 6	7.69e-04
Regulation of ornithine decarboxylase (ODC)	14 / 50	0.004	0.077	1	1 / 4	5.13e-04
Cross-presentation of soluble exogenous antigens (endosomes)	14 / 51	0.004	0.086	1	1 / 4	5.13e-04
Regulation of RUNX2 expression and activity	14 / 52	0.004	0.097	1	1 / 5	6.41e-04
Ubiquitin-dependent degradation of Cyclin D	14 / 53	0.004	0.108	1	1 / 3	3.84e-04
Ubiquitin Mediated Degradation of Phosphorylated Cdc25A	14 / 53	0.004	0.108	1	1 / 3	3.84e-04
p53-Independent DNA Damage Response	14 / 53	0.004	0.108	1	1 / 3	3.84e-04
p53-Independent G1/S DNA damage checkpoint	14 / 53	0.004	0.108	1	1 / 3	3.84e-04
Platelet degranulation	30 / 131	0.011	0.12	1	5 / 8	0.001
Autodegradation of the E3 ubiquitin ligase COP1	14 / 54	0.004	0.12	1	1 / 5	6.41e-04
CDT1 association with the CDC6:ORC:origin complex	14 / 59	0.005	0.191	1	1 / 3	3.84e-04
Degradation of AXIN	14 / 59	0.005	0.191	1	2 / 8	0.001
Dectin-1 mediated noncanonical NF-kB signaling	14 / 59	0.005	0.191	1	1 / 8	0.001
NIK-->noncanonical NF-kB signaling	14 / 59	0.005	0.191	1	1 / 9	0.001
AUF1 (hnRNP D0) binds and destabilizes mRNA	16 / 64	0.005	0.193	1	3 / 4	5.13e-04
Post-translational protein phosphorylation	25 / 114	0.009	0.195	1	1 / 1	1.28e-04
Regulation of Insulin-like Growth Factor (IGF) transport and uptake by Insulin-like Growth Factor Binding Proteins (IGFBPs)	26 / 120	0.01	0.206	1	6 / 6	7.69e-04
Degradation of GLI1 by the proteasome	15 / 61	0.005	0.224	1	2 / 5	6.41e-04
FBXL7 down-regulates AURKA during mitotic entry and in early mitosis	15 / 62	0.005	0.241	1	3 / 6	7.69e-04

Pathway name	Entities				Reactions	
	found	ratio	p-value	FDR*	found	ratio
Autodegradation of Cdh1 by Cdh1:APC/C	14 / 62	0.005	0.241	1	1 / 2	2.56e-04
Asymmetric localization of PCP proteins	14 / 62	0.005	0.241	1	1 / 4	5.13e-04
SCF(Skp2)-mediated degradation of p27/p21	14 / 62	0.005	0.241	1	1 / 7	8.97e-04
Activation of BAD and translocation to mitochondria	8 / 23	0.002	0.242	1	4 / 4	5.13e-04
The role of GTSE1 in G2/M progression after G2 checkpoint	17 / 79	0.007	0.272	1	5 / 10	0.001

* False Discovery Rate

4. Cross-presentation of soluble exogenous antigens (endosomes) (R-MMU-1236978)

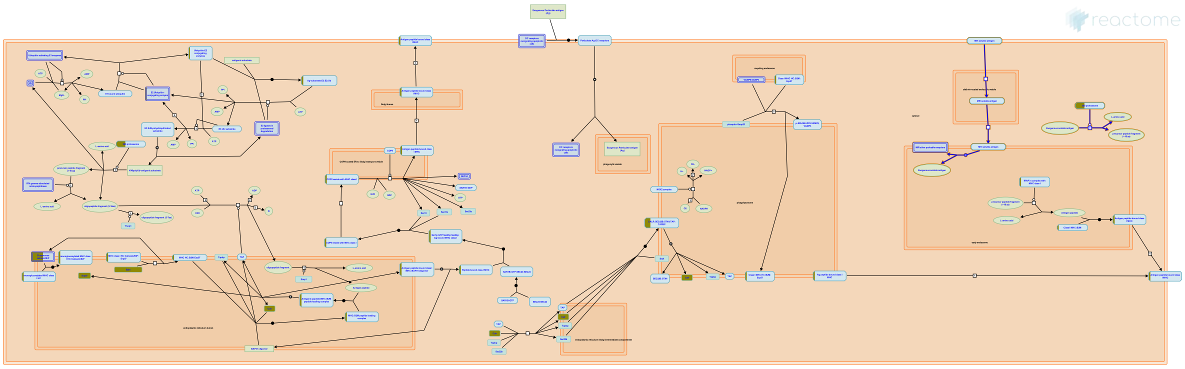

Inferred from: Cross-presentation of soluble exogenous antigens (endosomes).

This event has been computationally inferred from an event that has been demonstrated in another species.

The inference is based on the homology mapping from PANTHER. Briefly, reactions for which all involved PhysicalEntities (in input, output and catalyst) have a mapped orthologue/paralogue (for complexes at least 75% of components must have a mapping) are inferred to the other species. High level events are also inferred for these events to allow for easier navigation.

More details and caveats of the event inference in Reactome. For details on PANTHER see also: http://www.pantherdb.org/about.jsp

References

Edit history

Date	Action	Author
2020-03-08	Created	Cook J

Entities found in this pathway (14)

Input	UniProt Id	Input	UniProt Id	Input	UniProt Id
Psma1	Q9R1P4	Psma2	P49722	Psma3	O70435
Psma4	Q9R1P0	Psma5	Q9Z2U1	Psma6	Q9QUM9
Psma7	Q9Z2U0	Psmb1	O09061	Psmb2	Q9R1P3
Psmb3	Q9R1P1	Psmb4	P99026	Psmb5	O55234
Psmb8	P28063	Psme1	P97371		

10. Platelet degranulation (R-MMU-114608)

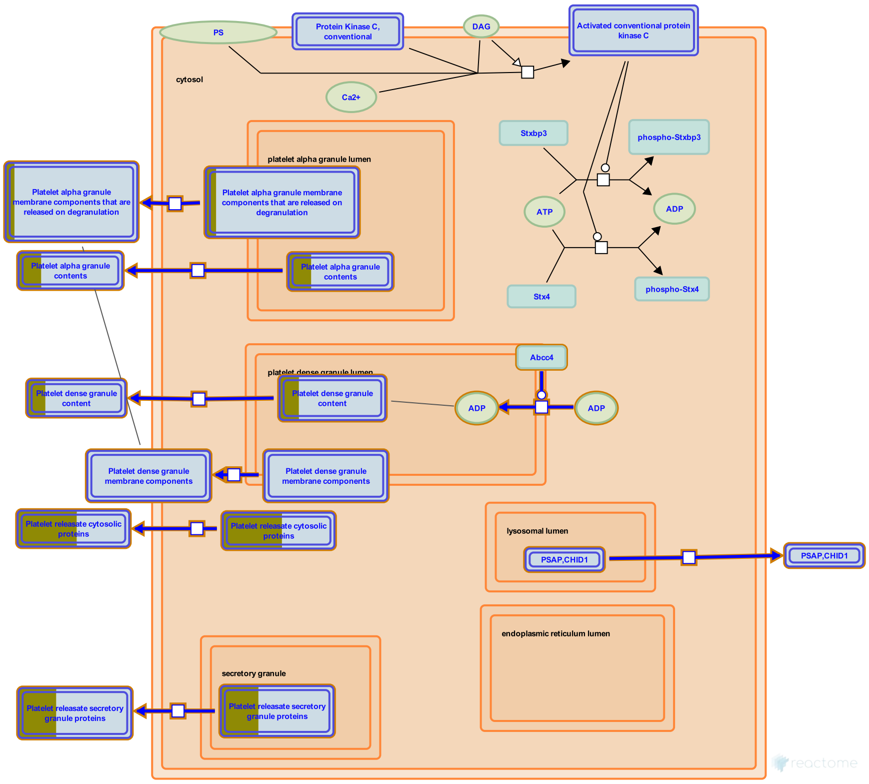

Inferred from: Platelet degranulation .

This event has been computationally inferred from an event that has been demonstrated in another species.

The inference is based on the homology mapping from PANTHER. Briefly, reactions for which all involved PhysicalEntities (in input, output and catalyst) have a mapped orthologue/paralogue (for complexes at least 75% of components must have a mapping) are inferred to the other species. High level events are also inferred for these events to allow for easier navigation.

More details and caveats of the event inference in Reactome. For details on PANTHER see also: http://www.pantherdb.org/about.jsp

References

Edit history

Date	Action	Author
2020-03-08	Created	Cook J

Entities found in this pathway (30)

Input	UniProt Id	Input	UniProt Id	Input	UniProt Id
A2m	Q6GQT1	Actg1	P63260	Actn1	Q7TPR4
Ahsg	P29699	Alb	P07724	Aldoa	P05064
Anxa5	P48036	Apoh	Q01339	Calm1	P0DP26
Ecm1	Q61508	Flna	Q8BTM8	Fn1	P11276
Igf2	P09535	Itih4	A6X935	Lgals3bp	Q07797

Input	UniProt Id
Plg	P20918
Serping1	P97290
Sytl4	Q9R0Q1
Thbs1	P35441
Tmsb4x	P20065
Qsox1	Q8BND5
Sod1	P08228
Tagln2	Q9WVA4
Timp1	P12032
Vcl	Q64727
Serpine1	P22777
Sparc	P07214
Tf	Q921I1
Tln1	P26039
Wdr1	O88342

6. Identifiers found

Below is a list of the input identifiers that have been found or mapped to an equivalent element in Reactome, classified by resource.

Entities (320)

Input	UniProt Id	Input	UniProt Id	Input	UniProt Id
A2m	P01023	Acta2	P62737	Actb	P60709
Actg1	P63260	Actn1	P12814	Actr2	P61160
Actr3	F1P679	Ahcy	P23526	Ahsg	P02765
Ak2	P54819	Akr1a1	P14550	Akr1b1	P15121
Alb	P02768	Aldoa	P04075	Anxa1	P04083
Anxa2	P07355	Anxa4	Q804G7	Anxa5	P08758
Apoe	P02649	Apoh	P02749	Arhgdia	F1PL93
Arhgdib	P52566	Arpc1b	E2RMT4	Arpc2	F1PAG6
Arpc3	E2R985	Arpc4	E2QWU0	Arpc5	O15511
Asah1	Q13510	Asl	P04424	Atic	P31335
Atox1	Q9TT99	Atp5f1a	P25705	Atp5f1b	J9NT37
Atp5f1d	P30049	Atp6v1a	P38606	B2m	P61769
Bgn	P28653	Blvra	P53004	Blvrb	P30043
C1qa	E1BSP0	C1qc	P02747	C1ra	Q8CG16
C3	P01024	Calm1	P0DP23	Calr	P27797
Calu	O43852	Cap1	Q01518	Capg	Q9BPX3
Cat	P04040	Ccn1	O00622	Ccn2	P29279
Cd14	P08571	Cdh11	P55288	Cdh2	P19022
Cep162	Q5TB80	Cfl1	P23528	Cfp	P27918
Chmp4b	Q7ZVC4	Ckb	P12277	Cmpk1	P30085
Cndp2	Q96KP4	Cnn2	Q99439	Col12a1	Q99715
Col1a1	P02453	Col1a2	P02465	Col2a1	P02458
Col3a1	P02461	Col5a2	P05997	Col6a1	P12109
Comp	Q9R0G6	Coro1a	P31146	Csf1	P09603
Csf1r	A7Z067	Csrp1	P21291	Cst3	P01034
Cstb	P04080	Ctsa	P10619	Ctsb	P07858
Ctsd	P07339	Ctsl	P07711	Ctss	P25774
Ctsz	Q9UBR2	Cycs	P99999	Dbi	P07108
Dld	P09622	Dpysl2	P47942	Ecm1	Q16610
Eef1a1	P62630	Eef1b	P24534	Eef1g	P26641
Eef2	P13639	Efhd2	F1SUW2	Eif2s1	P05198
Eif4b	P23588	Eif5a	P63241	Eno1	P06733
Ero1a	Q8R180	F2	P00734	Fabp4	P04117
Fabp5	Q01469	Fah	P16930	Fbln1	P23142
Fbln2	P98095	Fh	P07954	Fkbp1a	P62942
Flna	P21333	Flnb	A0A0G2JXT8	Fn1	P02751
Fscn1	Q16658	Fstl1	Q12841	Fth1	P02794
Ftl1	P02793	Gapdh	P04406	Gdi1	P31150
Gdi2	P50395	Glud1	P26443	Gm20390	E9PZF0
Gm2a	P17900	Gnpda1	P46926	Got1	P17174
Got2	P00505	Gpi	P06744	Gpnmb	A5A766

Input	UniProt Id	Input	UniProt Id	Input	UniProt Id
Grn	P28799	Gsn	P06396	Gsr	P00390-2
Gzme	P08884	H1-4	P10412	H1-5	P16401
H2-D1	P14427	H4c1	P62805	H4c11	P62805
H4c12	P62805	H4c14	P62805	H4c2	P62805
H4c3	P62805	H4c4	P62805	H4c6	P62805
H4c8	P62805	H4c9	P62805	H4f16	P62806
Hba	P01966	Hbb-bs	A8DUK4	Hexa	P06865
Hist1h4m	P62806	Hmox1	P09601	Hnrnpa1	F6XNZ3
Hnrnpa2b1	P22626	Hprt1	P00492	Hsp90aa1	P82995
Hsp90ab1	P34058	Hsp90b1	P14625	Hspa4	O88600
Hspa5	P11021	Hspa8	P11142	Hspg2	F1MER7
Igf2	P01344	Igfbp7	Q16270	Impa1	O55023
Iqgap1	P46940	Itih2	P19823	Itih4	Q14624
Itm2b	Q9Y287	Kpnb1	Q14974	Krt1	P04264
Krt10	P13645	Krt2	P35908	Krt5	F1SGG6
Krt73	Q86Y46	Krt76	Q01546	Krt77	Q7Z794
Krt78	Q8N1N4	Krt79	Q5XKE5	Lcp1	P13796
Ldha	P00340	Ldhb	P00337	Lgals1	P09382
Lgals3	P17931	Lgals3bp	Q08380	Lmna	P02545
Lox	P28300	Loxl2	Q9Y4K0	Lpl	P11151
Lrp1	E1BGJ0	Lum	P51884	Lyz2	P08905
Man2b1	O00754	Marcks	A0A287BRL8	Mdh1	P40925
Mdh2	P40926	Mfge8	Q08431	Mif	P14174
Mmp12	P39900	Mmp2	P08253	Msn	Q9W002
Mydgf	Q969H8	Myh9	F1MQ37	Myl6	P60661
Naglu	P54802	Nampt	P43490	Ncl	P09405
Nedd8	Q71UE8	Nid1	P14543	Nme1	P15531
Npc2	P61916	Npm1	P13084	Nucb1	Q02818
P4hb	P07237	Pdia3	F6WRR4	Pdia6	Q15084
Pdxk	O00764	Pea15	Q15121	Pebp1	P30086
Pfn1	P07737	Pgam1	P18669	Pgd	P52209
Pgk1	P00558	Pgls	O95336	Pgm1	P36871
Pgp	A6NDG6	Pkm	P14618	Plg	P00747
Plod1	Q02809	Pnp	P00491	Ppia	P62937
Ppib	P23284	Prdx1	P35700	Prdx2	Q61171
Prdx6	P30041	Prkcsh	P14314	Psma1	P25786
Psma2	P25787	Psma3	P25788	Psma4	P25789
Psma5	P28066	Psma6	P60900	Psma7	O14818
Psmb1	P20618	Psmb2	P49721	Psmb3	P49720
Psmb4	P28070	Psmb5	P28074	Psmb8	P28062
Psme1	Q06323	Ptgr1	Q14914	Qsox1	O00391
Rac1	P63000	Rad23b	Q4KMA2	Ralgapa2	Q2PPJ7
Rpl12	P30050	Rpl14	P50914	Rpl19	P84098
Rpl24	P83731	Rpl27	P61353	Rpl30	P62888
Rpl4	P36578	Rpl6	Q02878	Rpl7a	P62424
Rpl8	P62917	Rplp0	P05388	Rplp2	P05387
Rps12	P25398	Rps14	P62263	Rps2	P15880
Rps21	P63220	Rps28	P62857	Rps8	P62241
Rpsa	P08865	S100a11	P31949	Sema7a	F1NIZ9

Input	UniProt Id	Input	UniProt Id	Input	UniProt Id
Serpinb6a	Q6P9U0	Serpinc1	P01008	Serpine1	P05121
Serpine2	A0A287AYW8	Serping1	P05155	Serpinh1	P50454
Sfpq	A0A0G2K8K0	Snrpd3	E2RQ80, J9P8R0	Sod1	P00441
Sod2	P09671	Sparc	P09486	Spp1	P10451
Sri	P30626	Stip1	P31948	Sytl4	Q96C24
Tagln2	P37802	Taldo1	P37837	Tbca	O75347
Tcn2	P20062	Tf	P02787	Thbs1	P07996
Thbs2	P35442	Timp1	P01033	Timp2	P16035
Tkt	P29401	Tln1	Q9Y490	Tmsb4x	P62328
Tpi1	P60174	Tpm4	Q6IRU2	Try10	Q792Z1
Ttr	P02766	Tubb4b	P68371	Tubb5	P07437
Txn	P10639	Txndc5	Q8NBS9	Txnrd1	Q16881
Ube2n	P61088	Vapa	Q9P0L0	Vat1	Q99536
Vcl	P18206	Vcp	P55072	Vim	P02543
Wdr1	O75083	Ybx1	P62960	Ywhab	Q5XGC8
Ywhae	P62260	Ywhag	Q0V9W8	Ywhah	Q28DR3
Ywhaq	Q5BL40	Ywhaz	Q6P4Z5		

Input	Ensembl Id	Input	Ensembl Id	Input	Ensembl Id
Acta2	ENSG00000107796	Actb	ENST00000331789	Anxa1	ENSG00000135046
Anxa2	ENSG00000182718	Apoe	ENSG00000130203	Atp5f1b	ENSG00000110955
B2m	ENSG00000166710	C1qc	ENSGALP00000007599	Calr	ENSG00000179218
Cat	ENSG00000121691	Ccn1	ENSG00000145386	Ccn2	ENSG00000118523
Cfl1	ENSG00000172757	Cnn2	ENSG00000064666	Col1a1	ENSG00000108821
Col1a2	ENSG00000164692	Csf1	ENSG00000184371	Csf1r	ENSG00000182578
Csrp1	ENSG00000159176	Ctsd	ENSG00000117984	Cycs	ENSG00000172115
Fabp4	ENSG00000170323	Fn1	ENSG00000115414	Fscn1	ENSG00000075618
Fth1	ENST00000273550	Hmox1	ENSG00000100292	Hnrnpa2b1	ENSG00000122566
Hsp90aa1	ENSG00000080824	Hsp90b1	ENSG00000166598	Hspa5	ENSG00000044574
Hspa8	ENSG00000109971	Igf2	ENST00000337883, ENST00000381406	Igfbp7	ENSG00000163453
Lcp1	ENSG00000136167	Lgals3	ENSG00000131981	Lmna	ENSG00000160789
Lpl	ENSG00000175445	Mif	ENSG00000240972	Mmp2	ENSG00000087245
Msn	ENSG00000147065	Mydgf	ENSG00000074842	Nampt	ENSG00000105835
Pdia6	ENSG00000143870	Ppia	ENSG00000196262	Psmb8	ENSG00000204264
Rplp0	ENSG00000089157	Serpine1	ENSG00000106366	Serpinh1	ENSG00000149257
Sod1	ENSG00000142168	Sod2	ENSG00000112096	Sparc	ENSG00000113140
Spp1	ENSG00000118785	Taldo1	ENSG00000177156	Tf	ENSG00000117525
Thbs1	ENSG00000137801	Timp1	ENSG00000102265	Tln1	ENSG00000137076
Txnrd1	ENSG00000198431	Vapa	ENST00000340541	Vim	ENSG00000026025

Interactors (255)

Input	UniProt Id	Interacts with	Input	UniProt Id	Interacts with
Actb	P60709	P13569	Actg1	P63261	P23528
Actn1	P12814	P12931	Actr2	A7MB62	Q95107
Actr3	Q99JY9	P34152	Ahcy	P23526	Q8IWL3
Ahnak	Q09666-2	Q5SY16	Ahnak2	Q8IVF2-3	P24593
Ahsg	P12763	P17931	Akap12	Q02952	P00533
Alb	P02768	P02768	Aldoa	P05064	P63101

Input	UniProt Id	Interacts with	Input	UniProt Id	Interacts with
Anxa1	P04083	Q13546	Anxa2	EBI-8785901, P07355	Q8NBP7
Apoe	P02649	P02649	Apoh	P02749	P08519
Arhgdia	P52565	P60953	Arpc1b	O15143	O00401
Arpc3	O15145	P48552	Arpc4	P59998	O00401
Arpc5	O15511	P00533	Atic	P31939	P00533
Atox1	O00244	P35670	Atp5f1a	Q03265	P63101
Atp5f1b	P06576	P05919	Atp5f1d	P30049	P05919
Atp6v1a	P38606	Q9H444	B2m	P61769	P61769
Basp1	Q91XV3	Q62108	Blvra	P53004	P28482
C3	P01024-PRO_0000005911, P01024-PRO_0000005908, P01024	P08603, P05156	Calm1	P62158	P14416
Calr	P27797	P30101	Calu	O43852	P04049
Cap1	Q13114	Q99558	Capg	P40121	Q8IUQ4
Cat	P24270	P19096	Ccn1	P20248	Q9H211
Ccn2	P29279	P02751	Ccn5	O76076	P49639
Cd14	EBI-11177705, EBI-11177518	O43474	Cdh2	P15116	P18031
Cep162	Q5TB80	Q8IW35, O43303	Cfl1	P23528	P53667
Cfl2	Q549N0	P23528	Cfp	P27918	O75344
Chmp4b	Q9H444	Q8WUX9, Q9Y3E7	Col1a1	P02452	P02751
Col3a1	P02461-1	P09486	Comp	P49747	O00244
Coro1a	P31146	P40763	Coro1c	Q9ULV4	P19320
Csf1	P07141	P05480	Csf1r	P07333	Q8CIH5
Csrp1	P21291	Q16790	Ctsa	P10619	P08670
Ctsb	P07858	P02760	Ctsd	P07339	P04578
Cycs	P99999	Q9Y4K3	Dld	O08749	Q9CQV8
Dnpep	Q19087	O17761	Dstn	P60981	P60709
Ecm1	Q16610	Q9NQ94	Eef1a1	P68104	Q00987
Eef1b	P24534	P26641	Eef1g	P26641, P26641-2	P29692, P24534
Eef2	P58252	P63101	Eif2s1	P05198	Q9Z2B5
Eif4b	P23588	P53350	Eif5a	P63242	P63101
Eno1	P06733	P03496	Ero1a	Q96HE7	P07237
Erp29	P30040	Q969S0	Fabp4	P15090	P08670
Fah	P16930	P00533	Fbln1	P23142	P02647
Fbln2	P98095	Q9H2F3	Fh	P07954	P21673
Fkbp1a	P62942	P36896	Flna	P21333	O14786
Flnb	O75369	Q13233	Fn1	P02751	P07585
Fscn1	Q16658	P54278	Fstl1	Q12841	P08571
Fth1	P02794	P04792	Gapdh	P04406	P00558
Gdi1	P31150	P51149	Gdi2	P50395	Q8BMD2
Got1	P17174	P00533	Gpnmb	Q14956-1, Q14956	P00533
Grn	P28799	P07237	Gsn	P06396	P32121
H1-4	P10412	Q9Y468	H1-5	P16401	P19320
H4c1	P62805	P33992, P25205, P49736	H4c11	P62805	P33992, P25205, P49736
H4c12	P62805	P33992, P25205, P49736	H4c14	P62805	P33992, P25205, P49736
H4c2	P62805	P33992, P25205, P49736	H4c3	P62805	P33992, P25205, P49736

Input	UniProt Id	Interacts with	Input	UniProt Id	Interacts with
H4c4	P62805	P33992, P25205, P49736	H4c6	P62805	P33992, P25205, P49736
H4c8	P62805	P33992, P25205, P49736	H4c9	P62805	P33992, P25205, P49736
Hmox1	P09601	Q9NUX5	Hnrnpa1	P09651	P22626
Hnrnpa2b1	P22626	P67809	Hprt1	P00492	Q96G04
Hsp90aa1	P07900	Q99558	Hsp90ab1	P08238	O14757
Hsp90b1	P14625	P11021	Hspa4	P34932	P11142
Hspa5	P11021	P13569	Hspa8	P11142	Q99558
Hspe1	P61604	P19320	Hspg2	P98160-PRO_0000391621, P98160-PRO_0000391622, P98160	P35968
Iqgap1	B5DFH1	Q5M824	Itm2b	Q9Y287	P04578
Kpnb1	Q14974	Q99558	Krt1	P04264	O15552
Krt10	P13645	P19320	Krt76	Q01546	P14373
Krt79	Q5XKE5	A1L190	Lasp1	Q14847-2	Q8TDS5
Ldha	P00338	P11142	Ldhb	P07195	P19320
Lgals1	P09382	P35968	Lgals3	P16110	Q9Z0P7
Lgals3bp	Q08380	Q9BZR8	Lmna	P48678	P31750
Lox	P28300	Q9UBX5, P15502	Lrp1	Q07954	P02649
Lum	P51884	P50281	Lxn	Q9BS40	Q9UQK1
Mdh1	P40925	P00533	Mdh2	P40926	P19320
Mif	P14174	P14174	Mmp2	P08253	P16035
Msn	P26038	P16070	Mtpn	P62775	Q5XI32
Myh9	P35579	O00255	Myl6	P60660-2	P46940
Nampt	P43490	P43490	Ncl	P19338	Q00987
Nedd8	Q15843	P68104	Nme1	P15531	P10911
Npc2	P61916	16113, O15118	Npm1	P06748	Q8N726
Nsfl1c	O35987	Q01853	Nucb1	Q02818	P10823
P4hb	P07237	Q6PJG9	Pdcd5	O14737	Q9NRD5
Pdia3	P30101	P30304	Pdia6	Q15084	P11021
Pea15	Q15121	P28482	Pebp1	P30086	P16050
Pfn1	P07737	O08816	Pgam1	P18669	P12004
Pgk1	P00558, P00558-1	O15379	Pkm	P14618-1	Q16665
Plod1	Q02809	P55795	Pls3	Q9NRY6	P49639
Ppia	P62937	O00267	Ppib	P23284	P40855
Ppic	P45877	Q8N9N5	Prdx1	Q06830	P04156
Prdx2	P32119	P10599	Prdx6	P30041	Q7KZN9
Prkcsh	P14314	P04578	Psma1	P25786	P21673
Psma2	P25787	Q9Y5K5	Psma3	P25788	Q12846
Psma4	P25789	Q16665	Psma5	P28066	Q9Y5K5
Psma6	P60900	Q9Y5K5	Psma7	O14818	Q16665
Psmb1	P20618	Q9Y5K5	Psmb2	P49721	Q9Y5K5
Psmb3	P49720	Q9Y5K5	Psmb4	P28070	Q9Y5K5
Psmb5	P28074	Q9Y5K5	Psmb8	P28062-2	Q13114
Psme1	Q06323	Q9UL46	Ptk7	Q13308	P35222
Rac1	P63000	Q13177	Rad23b	P54727	P53350
Rcn3	Q96D15	Q9UMX1	Rnh1	P13489	P03950
Rpl12	P30050	P27824	Rpl14	P50914	Q99558

Input	UniProt Id	Interacts with	Input	UniProt Id	Interacts with
Rpl19	P84098	Q5S007	Rpl24	P83731	Q99558
Rpl27	P61353	Q99558	Rpl30	P14120	Q12499
Rpl4	P36578	Q99558	Rpl6	Q02878	Q99558
Rpl7a	P62424	Q99558	Rpl8	P62917	Q99558
Rplp0	P05388	P03372	Rps12	P25398	P03372
Rps14	P62263	Q9Y3D8	Rps2	O74892	O13648
Rps8	P62241	Q15843	Rpsa	P08865	P03372
Rrbp1	Q9P2E9	P27824	S100a11	P31949	P04271
S100a4	P26447	Q00987	S100a6	P06703	Q00987
Sema7a	O75326	O60486	Serpinc1	P01008	P01008
Serpine1	P05121	Q8NBQ5	Serpinf1	P36955	Q9P2X3
Serping1	E9PK97	Q9BRI3	Serpinh1	P50454	P03372
Sfpq	P23246	Q13882	Sh3bgrl	O75368	P00533
Snrpd3	P62318	P14678	Sod1	P00441	P29692
Sod2	P04179	P04179	Spp1	P10451	P16070
Sri	P30626	P30626	Stip1	P31948	P07900
Sytl4	Q96C24	Q86TI0	Tf	P02787	O00501
Thbs1	P07996-PRO_0000035842	P16671	Thbs2	Q03350	Q07954
Timp1	P01033	P14780	Timp2	P16035	P08253
Tkt	Q16832	P29353	Tln1	Q9Y490, P26039	P05556
Tmsb10	P63313	Q9NUX5	Tmsb4x	P20065	Q8WWQ8
Tpi1	P60174	P12004	Tpm4	P67936	Q9NRD5
Tpt1	P13693	P29692	Ttr	P02766	Q15109
Tubb4b	P68371	Q5S007	Tubb5	P04350	P22736
Txn	P10599	P19883	Txndc5	Q86UY0	O43597
Txnrd1	Q16881	Q03135	Ube2n	P61088	Q9UNE7
Vapa	Q9WV55	Q9QXM1	Vat1	P54219-3	Q9Y5Y5
Vcl	Q64727	Q8VI36	Vcp	P55072	O96017
Vim	P08670	P11021	Wdr1	O75083	P62993
Ybx1	P67809	Q9NZI8, Q9Y6M1	Ywhab	P35213	Q9JLT6
Ywhae	P62258	P30304	Ywhag	P61981	P30304, O14757
Ywhah	Q04917	P30304	Ywhaq	P27348	P30304
Ywhaz	P63104	P30304			

Input	ChEBI Id	Interacts with	Input	ChEBI Id	Interacts with
Actb	P60709	18348	Actg1	P63261	18348
Actn1	P12814	18348	Cfl1	P23528	18348
Dstn	P60981	16618	Flna	P21333	18348
Flnb	O75369	18348	Iqgap1	P46940	16618
Kpnb1	Q14974	18348	Ldha	P00338	18348
Lrp1	Q07954	29108	Msn	P26038	18348
Myh9	P35579	18348	Myl6	P60660	18348
Npc2	P61916	16113	Pkm	P14618-1	18319
Rac1	P63000	15996	Tln1	Q9Y490	18348

7. Identifiers not found

These 19 identifiers were not found neither mapped to any entity in Reactome.

Aebp1	Afm	Anxa3	Cd5l	Chil3	Cnn3	Cpq	Dkk3
Fkbp10	Hars1	Map4	Mroh6	Pdia4	Postn	Prl7c1	Ptms
Rexo2	Tagln	Twf1					

reactome

sWAT-MSC secretome from ND mice
Pathway Analysis Report

Table of Contents

1. Introduction
2. Properties
3. Genome-wide overview
4. Most significant pathways
5. Pathways details
6. Identifiers found
7. Identifiers not found

1. Introduction

Reactome is a curated database of pathways and reactions in human biology. Reactions can be considered as pathway 'steps'. Reactome defines a 'reaction' as any event in biology that changes the state of a biological molecule. Binding, activation, translocation, degradation and classical biochemical events involving a catalyst are all reactions. Information in the database is authored by expert biologists, entered and maintained by Reactome’s team of curators and editorial staff. Reactome content frequently cross-references other resources e.g. NCBI, Ensembl, UniProt, KEGG (Gene and Compound), ChEBI, PubMed and GO. Orthologous reactions inferred from annotation for Homo sapiens are available for 17 non-human species including mouse, rat, chicken, puffer fish, worm, fly, yeast, rice, and Arabidopsis. Pathways are represented by simple diagrams following an SBGN-like format.

Reactome's annotated data describe reactions possible if all annotated proteins and small molecules were present and active simultaneously in a cell. By overlaying an experimental dataset on these annotations, a user can perform a pathway over-representation analysis. By overlaying quantitative expression data or time series, a user can visualize the extent of change in affected pathways and its progression. A binomial test is used to calculate the probability shown for each result, and the p-values are corrected for the multiple testing (Benjamini–Hochberg procedure) that arises from evaluating the submitted list of identifiers against every pathway.

To learn more about our Pathway Analysis, please have a look at our relevant publications:

Fabregat A, Sidiropoulos K, Garapati P, Gillespie M, Hausmann K, Haw R, … D’Eustachio P (2016). The reactome pathway knowledgebase. Nucleic Acids Research, 44(D1), D481–D487. https://doi.org/10.1093/nar/gkv1351.

Fabregat A, Sidiropoulos K, Viteri G, Forner O, Marin-Garcia P, Arnau V, … Hermjakob H (2017). Reactome pathway analysis: a high-performance in-memory approach. BMC Bioinformatics, 18.

2. Properties

- This is an **overrepresentation** analysis: A statistical (hypergeometric distribution) test that determines whether certain Reactome pathways are over-represented (enriched) in the submitted data. It answers the question ‘Does my list contain more proteins for pathway X than would be expected by chance?’ This test produces a probability score, which is corrected for false discovery rate using the Benjamani-Hochberg method.
- 486 out of 532 identifiers in the sample were found in Reactome, where 10159 pathways were hit by at least one of them.
- IntAct interactors were included to increase the analysis background. This greatly increases the size of Reactome pathways, which maximises the chances of matching your submitted identifiers to the expanded pathway, but will include interactors that have not undergone manual curation by Reactome and may include interactors that have no biological significance, or unexplained relevance.
- This report is filtered to show only results for species 'Mus musculus' and resource 'all resources'.
- The unique ID for this analysis (token) is MjAyMDAzMjQxMjA5NDRfMzE4Mg%3D%3D. This ID is valid for at least 7 days in Reactome’s server. Use it to access Reactome services with your data.

3. Genome-wide overview

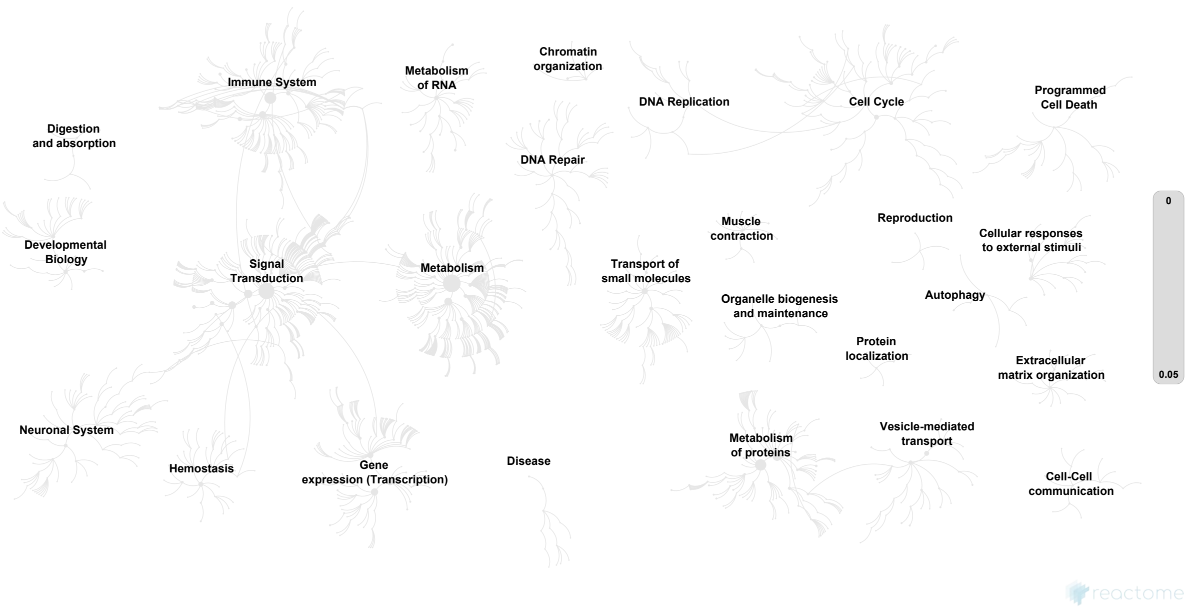

This figure shows a genome-wide overview of the results of your pathway analysis. Reactome pathways are arranged in a hierarchy. The center of each of the circular "bursts" is the root of one top-level pathway, for example "DNA Repair". Each step away from the center represents the next level lower in the pathway hierarchy. The color code denotes over-representation of that pathway in your input dataset. Light grey signifies pathways which are not significantly over-represented.

4. Most significant pathways

The following table shows the 25 most relevant pathways sorted by p-value.

Pathway name	Entities				Reactions	
	found	ratio	p-value	FDR*	found	ratio
Crosslinking of collagen fibrils	12 / 25	0.002	0.054	1	13 / 13	0.002
Association of TriC/CCT with target proteins during biosynthesis	6 / 11	9.09e-04	0.093	1	1 / 1	1.28e-04
Antagonism of Activin by Follistatin	3 / 4	3.31e-04	0.104	1	2 / 2	2.56e-04
Laminin interactions	11 / 29	0.002	0.198	1	7 / 8	0.001
HSF1 activation	6 / 11	9.09e-04	0.199	1	5 / 6	7.69e-04
Chk1/Chk2(Cds1) mediated inactivation of Cyclin B:Cdk1 complex	6 / 14	0.001	0.204	1	1 / 4	5.13e-04
NCAM1 interactions	9 / 24	0.002	0.237	1	3 / 3	3.84e-04
Regulation of ornithine decarboxylase (ODC)	17 / 50	0.004	0.246	1	2 / 4	5.13e-04
Regulation of Insulin-like Growth Factor (IGF) transport and uptake by Insulin-like Growth Factor Binding Proteins (IGFBPs)	38 / 120	0.01	0.248	1	5 / 6	7.69e-04
Post-translational protein phosphorylation	36 / 114	0.009	0.26	1	1 / 1	1.28e-04
Cross-presentation of soluble exogenous antigens (endosomes)	17 / 51	0.004	0.271	1	4 / 4	5.13e-04
Anchoring fibril formation	6 / 16	0.001	0.295	1	2 / 4	5.13e-04
Assembly of collagen fibrils and other multimeric structures	22 / 69	0.006	0.303	1	20 / 26	0.003
Activation of C3 and C5	3 / 7	5.79e-04	0.314	1	3 / 3	3.84e-04
AUF1 (hnRNP D0) binds and destabilizes mRNA	21 / 64	0.005	0.345	1	3 / 4	5.13e-04
Elastic fibre formation	14 / 44	0.004	0.355	1	13 / 16	0.002
The role of GTSE1 in G2/M progression after G2 checkpoint	24 / 79	0.007	0.376	1	5 / 10	0.001
Regulation of RUNX2 expression and activity	16 / 52	0.004	0.39	1	1 / 5	6.41e-04
Ubiquitin-dependent degradation of Cyclin D	16 / 53	0.004	0.418	1	1 / 3	3.84e-04
p53-Independent DNA Damage Response	16 / 53	0.004	0.418	1	1 / 3	3.84e-04
p53-Independent G1/S DNA damage checkpoint	16 / 53	0.004	0.418	1	1 / 3	3.84e-04
Ubiquitin Mediated Degradation of Phosphorylated Cdc25A	16 / 53	0.004	0.418	1	1 / 3	3.84e-04

Pathway name	Entities				Reactions	
	found	ratio	p-value	FDR*	found	ratio
RHO GTPases activate IQGAPs	10 / 33	0.003	0.447	1	5 / 5	6.41e-04
Autodegradation of the E3 ubiquitin ligase COP1	16 / 54	0.004	0.447	1	1 / 5	6.41e-04
Transport of gamma-carboxylated protein precursors from the endoplasmic reticulum to the Golgi apparatus	3 / 9	7.44e-04	0.463	1	3 / 9	0.001

* False Discovery Rate

11. Cross-presentation of soluble exogenous antigens (endosomes) (R-MMU-1236978)

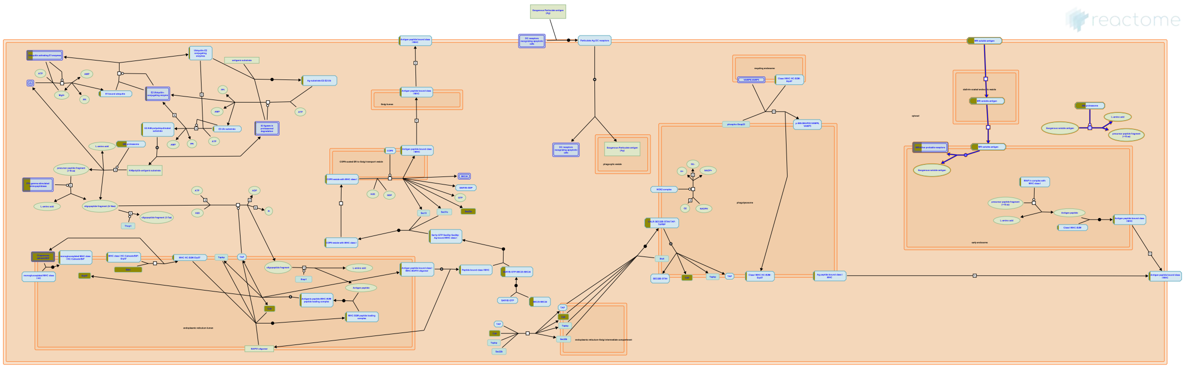

Inferred from: Cross-presentation of soluble exogenous antigens (endosomes).

This event has been computationally inferred from an event that has been demonstrated in another species.

The inference is based on the homology mapping from PANTHER. Briefly, reactions for which all involved PhysicalEntities (in input, output and catalyst) have a mapped orthologue/paralogue (for complexes at least 75% of components must have a mapping) are inferred to the other species. High level events are also inferred for these events to allow for easier navigation.

More details and caveats of the event inference in Reactome. For details on PANTHER see also: http://www.pantherdb.org/about.jsp

References

Edit history

Date	Action	Author
2020-03-08	Created	Cook J

Entities found in this pathway (17)

Input	UniProt Id	Input	UniProt Id	Input	UniProt Id
Mrc2	Q64449	Psma1	Q9R1P4	Psma2	P49722
Psma3	O70435	Psma4	Q9R1P0	Psma5	Q9Z2U1
Psma6	Q9QUM9	Psma7	Q9Z2U0	Psmb1	O09061
Psmb2	Q9R1P3	Psmb3	Q9R1P1	Psmb4	P99026
Psmb5	O55234	Psmb6	Q60692	Psmb7	P70195
Psmc2	P46471	Psmd2	Q8VDM4		

6. Identifiers found

Below is a list of the input identifiers that have been found or mapped to an equivalent element in Reactome, classified by resource.

Entities (486)

Input	UniProt Id	Input	UniProt Id	Input	UniProt Id
A2m	P01023	Acta2	P62738	Actb	P60709
Actg1	P63261	Actn1	P12814	Actn4	O43707
Actr2	P61160	Actr3	F1P679	Adam15	Q13444
Adam9	Q13443	Adamts1	Q9UHI8	Adamts5	Q9UNA0
Adss2	P30520	Ahcy	P23526	Ahsg	P02765
Aimp1	Q12904	Ak2	P54819	Akr1a1	P14550
Akr1b1	P15121	Alb	P02768	Aldoa	P04075
Ang	P21570	Angptl4	Q9Z1P8	Anxa1	P04083
Anxa2	P07355	Anxa5	P08758	Anxa6	P08133
Ap2a1	O95782	Aplp2	Q06481	Apoh	P02749
Arcn1	Q5XJY5	Arhgdia	Q99PT1	Arhgdib	P52566
Arpc1b	E2RMT4	Arpc3	E2R985	Arpc4	E2QWU0
Asah1	Q13510	Asl	P04424	Atic	P31939
Atox1	Q9TT99	Atp1a1	P05023	Atp5f1b	J9NT37
Atp6ap2	O75787	Atp6v1a	P38606	Atp6v1b2	L7N2R0
Axl	P30530	B2m	P61769	B4galt1	P15291
Bag3	Q5U2U8	Bgn	P21810	Blvrb	P30043
Bmp1	P13497	C1ra	Q8CG16	C3	P01024
C4b	P01029	Cald1	Q62736	Calr	P27797
Calu	O43852	Cand1	Q86VP6	Cant1	Q8WVQ1
Canx	P35564	Cap1	Q01518	Capg	Q9BPX3
Capn2	P17655	Capza2	P47755	Cbr3	F1N8Y3
Cck	K9J7V7	Ccl2	P13500	Ccl7	Q03366
Ccl8	Q09141	Ccn1	O00622	Ccn2	P29279
Cct2	P78371	Cct3	P49368	Cct5	P48643
Cct7	Q99832	Cct8	P50990	Cd81	P35762
Cdc37	Q16543	Cdc42	P60953	Cdh11	P55288
Cdh2	P19022	Cfl1	P23528	Ckb	P12277
Cltc	Q00610	Cmpk1	P30085	Cndp2	Q96KP4
Cnn2	Q99439	Col12a1	Q99715	Col1a1	P02452
Col1a2	P08123	Col3a1	P02461	Col4a1	P02462
Col4a2	P08572	Col5a1	P20908	Col5a2	P05997
Col6a1	P12109	Col6a2	P12110	Col6a3	P12111
Colec12	Q5KU26	Comp	P49747	Copa	Q8CIE6
Copg1	Q9QZE5	Cotl1	Q14019	Cpe	P16870
Creg1	O75629	Crk	A0A287A2R9	Crlf1	F1ML91
Csf1	P09603	Csrp1	P21291	Cst3	P01034
Cst6	P04080	Cstb	P04080	Ctsa	P10619
Ctsb	P07858	Ctsh	P09668	Ctsl	P07711
Ctsz	Q9UBR2	Cxcl12	Q5EBF6	Cxcl5	P50228
Cycs	P99999	Dag1	Q14118	Dbi	P07108

Input	UniProt Id	Input	UniProt Id	Input	UniProt Id
Dcn	P07585	Dctn2	Q13561	Ddah1	P56965
Dpysl2	P47942	Ecm1	Q16610	Eef1a1	P62630
Eef1b	P24534	Eef1g	P26641	Eef2	P13639
Efemp1	Q12805	Efemp2	O95967	Efhd2	F1SUW2
Ehd1	Q641Z6	Ehd2	Q4V8H8	Eif2s1	P05198
Eif2s3x	Q9Z0N1	Eif3c	Q99613	Eif3i	Q13347
Eif4h	Q15056	Eif5a	P63241	Eloc	Q15369
Emilin1	Q9Y6C2	Emilin2	Q9BXX0	Eno1	P06733
Eno3	P13929	Ero1a	E2RNW5	Erp44	Q9BS26
Ext1	Q16394	Ext2	Q93063	F2	J9NSF9
F3	P13726	F5	P12259	Fabp5	Q01469
Fah	P16930	Fam3c	Q92520	Fasn	E7F5V3
Fbln1	P23142	Fbln2	P98095	Fbln5	Q9UBX5
Fbn1	P35555	Fermt2	A0A0G2JWC7	Fh	Q148D3
Fkbp1a	P62942	Flna	P21333	Flnb	A0A0G2JXT8
Flnc	D3ZHA0	Fmod	Q06828	Fscn1	Q16658
Fst	P47931	Fstl1	Q12841	Fstl3	O95633
Ftl1	P02793	G6pdx	Q00612	Gaa	P10253
Galnt2	Q10471	Gapdh	P04406	Gars1	P41250
Gas6	Q14393	Gclc	P48506	Gclm	P48507
Gdi1	P31150	Gdi2	P50395	Ggh	Q92820
Glb1	P16278	Glo1	J9NRV6	Glrx3	O76003
Gm20390	E9PZF0	Gm20547	B8JJN0	Gm2a	P17900
Gng12	Q9UBI6	Got1	P17174	Got2	P00505
Gpc1	P35052	Gpc4	O75487	Gpi	P06744
Grn	P28799	Gsn	P06396	Gstm1	A4IFG0, Q9N0V4
Gstm2	F6Q751	Gsto1	P78417	Gstp1	P09211
H4c1	P62805	H4c11	P62805	H4c12	P62805
H4c14	P62805	H4c2	P62805	H4c3	P62805
H4c4	P62805	H4c6	P62805	H4c8	P62805
H4c9	P62805	H4f16	P62804	Hdgf	P51858
Hexa	P06865	Hexb	P07686	Hist1h4m	P62804
Hmgb1	P09429	Hmox1	P09601	Hnrnpa1	P04256
Hnrnpa2b1	P22626	Hnrnpf	Q794E4	Hprt1	P00492
Hsp90aa1	P82995	Hsp90ab1	P34058	Hsp90b1	P14625
Hspa1a	P0DMV8	Hspa4	O88600	Hspa5	P11021
Hspa8	P11142	Hspb1	P14602	Hspd1	A4II42
Hspg2	P98160	Htra1	Q92743	Hyou1	Q9Y4L1
Idh1	O75874	Igfbp2	P47877	Igfbp4	P22692
Igfbp6	P47880	Igfbp7	Q16270	Inhba	Q04998
Ipo5	O00410	Iqgap1	P46940	Itga5	P08648
Itgb1	P05556	Itih2	P19823	Itm2b	Q9Y287
Kif5b	P33176	Kpnb1	Q14974	Krt1	P04264
Krt10	E1C6Q9	Krt2	G3MZ71	Krt77	G3MYU2
Krt78	A6QNX5	Krt79	Q148H7	Lama2	P24043
Lama4	Q16363	Lamb1	P07942	Lamb2	P11047, P55268
Lamc1	P11047	Lap3	Q01532	Ldha	P00340
Ldlr	P35951	Lgals1	P09382	Lgals3	P17931
Lgals3bp	Q08380	Lgmn	Q9R0J8	Lmna	P48678-3

Input	UniProt Id	Input	UniProt Id	Input	UniProt Id
Lox	P28300	Loxl1	Q08397	Loxl2	Q9Y4K0
Loxl3	P58215	Lpl	P11151	Lrp1	E1BGJ0
Lta4h	P09960	Ltbp2	Q14767	Lum	P51884
Lyz2	P08905	Man2a1	Q16706	Man2b1	O00754
Mapk1	P28482	Mapre1	Q61166	Marcks	A0A287BRL8
Mcpt8	P43430	Mdh1	P40925	Mdh2	P40926
Mfap5	Q13361	Mfge8	Q08431	Mif	P14174
Minpp1	Q9UNW1	Mmp12	P39900	Mmp19	Q99542
Mmp2	P08253	Mmp3	P08254	Mrc2	Q9UBG0
Msn	P26038	Mt2	P02798	Mtap	Q13126
Myh9	F1MQ37	Myl12a	Q6ZWQ9	Ncam1	P13591
Ncl	P09405	Nedd4	P46935	Nedd8	Q71UE8
Nid1	P14543	Nid2	Q14112	Nme1	P15531
Npepps	P55786	Nqo1	P15559	Nrp1	A4III3
Nt5c	Q8TCD5	Ntf3	P20783	Nucb1	Q02818
Nudc	Q6NV13	Oat	P04181	Ogn	P20774
Ostf1	Q92882	P4hb	P07237	Pabpc1	P11940
Pcbp1	Q15365	Pcolce	Q15113	Pcsk9	Q8NBP7
Pdap1	Q13442	Pdcd6ip	Q8WUM4	Pdia3	F6WRR4
Pdia6	Q15084	Pdlim5	Q96HC4	Pea15	Q15121
Pebp1	P30086	Pfn1	P07737	Pgam1	P18669
Pgd	Q9DCD0	Pgf	E2QZR3	Pgk1	P00558
Pgls	Q9CQ60	Pgm1	P36871	Pkm	P14618
Pla1a	Q53H76	Plau	P00749	Plod1	Q02809
Plod2	O00469	Plod3	O60568	Pnp	P00491
Ppia	P62937	Ppib	P23284	Ppid	F1RTY6
Ppp2r1a	P30153	Prdx2	Q61171	Prdx5	P99029
Prdx6	P30041	Prep	Q5JRX3	Prkcsh	P14314
Prnp	P04156	Pros1	P07225	Prss23	O95084
Psma1	P25786	Psma2	P25787	Psma3	P25788
Psma4	P25789	Psma5	P28066	Psma6	P60900
Psma7	O14818	Psmb1	P20618	Psmb2	P49721
Psmb3	P49720	Psmb4	P28070	Psmb5	P28074
Psmb6	P28072	Psmb7	Q99436	Psmc2	P35998
Psmd2	Q13200	Ptpa	P9WIA1	Ptx3	P26022
Pvr	Q5U334	Pxdn	Q92626	Qpct	Q16769
Qsox1	O00391	Rab10	P61026	Rab2a	P53994
Rab7a	P51149	Rac1	P63000	Rack1	P63243
Rad23b	Q4KMA2	Ran	P62828	Rcn1	Q15293
Rdx	P35241	Rpl12	P30050	Rpl19	P84098
Rpl24	P83731	Rpl4	P36578	Rpl5	P46777
Rpl6	Q02878	Rpl8	P62917	Rplp0	P05388
Rplp1	P05386	Rplp2	P05387	Rps14	P62263
Rps15a	P62244	Rps16	P62249	Rps2	P15880
Rps23	P62266	Rps28	P62857	Rps4x	P62701
Rps5	P46782	Rps7	P62081	Rps8	P62241
Rpsa	P08865	Rsu1	D4A8F2	Rtn4	Q99P72
S100a11	P31949	Sat2	Q96QD8	Sdcbp	O00560
Sdccag8	Q86SQ7	Sec23a	F1SHL3	Sema7a	F1NIZ9

Input	UniProt Id	Input	UniProt Id	Input	UniProt Id
Serpinb6a	Q6P9U0	Serpinc1	P01008	Serpine1	P05121
Serpine2	A0A287AYW8	Serping1	P05155	Serpinh1	P50454
Set	Q63945	Slit3	F1RR92	Sod1	P00441
Sod2	P09671	Sod3	O09164	Spon2	Q9BUD6
Spp1	P10451	Sptbn1	Q62261	Stip1	P31948
Tagln2	P37802	Taldo1	P37837	Tars1	P26639
Tcn2	P20062	Tcp1	P17987	Tf	P02787
Tgfbi	Q15582	Thbs1	P07996	Thbs2	P35442
Thy1	P04216	Timp1	P01033	Timp2	P16035
Tkt	P29401	Tll1	O43897	Tln1	Q9Y490
Tmsb4x	P62328	Tpi1	P60174	Tpm4	P09495
Tpp1	Q96AP0	Tuba1a	Q71U36	Tuba4a	P68366
Tubb2a	Q13885	Tubb4b	P68371	Tubb5	P04350
Tubb6	Q9BUF5	Txndc5	Q8NBS9	Txnrd1	Q16881
Uba1	Q02053	Ube2d3	P61077	Ube2i	Q28CQ4
Ube2n	Q9EQX9	Ugdh	O60701	Ugp2	Q16851
Usp5	E1C8W4	Vapa	Q9P0L0	Vat1	Q99536
Vcam1	A7MBB0	Vcl	P18206	Vcp	P55072
Vegfa	P15692	Vegfd	O43915	Vim	P02543
Vps35	Q54C24	Wdr1	O75083	Xdh	F1S3Y7
Xpnpep1	Q54G06	Yars	P54577	Ybx1	P62960
Ywhab	F1SDR7	Ywhae	P62260	Ywhag	F2Z4Z1
Ywhah	F2Z4Y1	Ywhaq	F1SA98	Ywhaz	F2Z558

Input	Ensembl Id	Input	Ensembl Id	Input	Ensembl Id
Acta2	ENSG00000107796	Actb	ENST00000331789	Angptl4	ENSG00000167772
Anxa1	ENSG00000135046	Anxa2	ENSG00000182718	Atp5f1b	ENSG00000110955
B2m	ENSG00000166710	Calr	ENSG00000179218	Ccl2	ENSG00000108691
Ccn1	ENSG00000145386	Ccn2	ENSG00000118523	Cdc42	ENSG00000070831
Cfl1	ENSG00000172757	Cnn2	ENSG00000064666	Col1a1	ENSG00000108821
Col1a2	ENSG00000164692	Csf1	ENSG00000184371	Csrp1	ENSG00000159176
Cxcl12	ENSG00000107562	Cycs	ENSG00000172115	F3	ENSG00000117525
Fasn	ENSG00000169710	Fscn1	ENSG00000075618	Gsto1	ENSG00000148834
Hdgf	ENSG00000143321	Hmox1	ENSG00000100292	Hnrnpa2b1	ENSG00000122566
Hnrnpf	ENSG00000169813	Hsp90aa1	ENSG00000080824	Hsp90b1	ENSG00000166598
Hspa1a	ENSG00000204389, ENSG00000215328, ENSG00000234475, ENSG00000235941, ENSG00000237724	Hspa5	ENSG00000044574	Hspa8	ENSG00000109971
Hspb1	ENSG00000106211	Hspd1	ENSG00000144381	Hyou1	ENSG00000149428
Igfbp7	ENSG00000163453	Itga5	ENSG00000161638	Itgb1	ENSG00000150093
Lgals3	ENSG00000131981	Lmna	ENSG00000160789	Lpl	ENSG00000175445
Mif	ENSG00000240972	Mmp2	ENSG00000087245	Mmp3	ENSG00000149968
Msn	ENSG00000147065	Mt2	ENSG00000125148	Mtap	ENSG00000099810
Ncam1	ENSG00000149294	Pdia6	ENSG00000143870	Ppia	ENSG00000196262
Rplp0	ENSG00000089157	Serpine1	ENSG00000106366	Serpinh1	ENSG00000149257
Sod1	ENSG00000142168	Sod2	ENSG00000112096	Spp1	ENSG00000118785
Taldo1	ENSG00000177156	Tcp1	ENSG00000120438	Tf	ENSG00000117525
Thbs1	ENSG00000137801	Timp1	ENSG00000102265	Tln1	ENSG00000137076
Tpp1	ENSG00000166340	Txnrd1	ENSG00000198431	Vapa	ENST00000340541

Input	UniProt Id	Interacts with	Input	UniProt Id	Interacts with
Cxcl12	P48061	P78556, P13501, O15444, Q99616	Cycs	P99999	Q9Y4K3
Dag1	Q14118	Q14118	Dcn	P07585	P02751
Dctn2	Q13561	P10636-8	Dnpep	Q19087	O17761
Dstn	P60981	P60709	Ecm1	Q16610	Q9NQ94
Eef1a1	P68104	Q9NYA1	Eef1b	P24534	P26641
Eef1g	P26641, P26641-2	P29692, P24534	Eef2	P58252	P63101
Efemp1	Q12805	P24592	Efemp2	O95967	Q9UBX5, P15502
Ehd1	Q9H4M9	16618, Q9H1K0	Ehd2	Q9NZN3	Q9H1K0
Eif2s1	P05198	Q9Z2B5	Eif3c	Q99613	P47813
Eif4h	Q15056-2	Q92796	Eif5a	P63242	P63101
Eloc	Q15369	Q16665	Emilin1	Q9Y6C2-2, Q9Y6C2	Q00994
Eno1	P06733	P03496	Ero1a	Q96HE7	P07237
Erp29	P30040	Q969S0	F3	P13726	P08709
Fah	P16930	P00533	Fam3c	Q92520	Q15223
Fasn	P12785	P12785	Fbln1	P23142	P02647
Fbln2	P98095	Q9H2F3	Fbln5	Q9UBX5	P15502
Fbn1	P35555	P15502	Fermt2	Q96AC1	P35222
Fh	P07954	P21673	Fhl1	Q13642	P04156
Fkbp1a	P62942	P36896	Flna	P21333	O14786
Flnb	O75369	Q13233	Flnc	Q14315	O75923
Fmod	P13605	P08603	Fscn1	Q16658	P54278
Fst	P19883	P10599	Fstl1	Q12841	P08571
G3bp1	Q13283	Q04637	Galnt2	Q10471	Q9HC36
Gapdh	P04406	P00558	Gdi1	P31150	P51149
Gdi2	P50395	Q8BMD2	Glb1	P16278	P08670
Glrx3	O76003	Q16790	Gng12	Q9UBI6	Q14192
Got1	P17174	P00533	Gpc4	O75487	Q9NRD5
Grn	P28799	P98160	Gsn	P06396	P32121
Gsto1	P78417	P04792	Gstp1	P09211	P22735
H4c1	P62805	P33992, P25205, P49736	H4c11	P62805	P33992, P25205, P49736
H4c12	P62805	P33992, P25205, P49736	H4c14	P62805	P33992, P25205, P49736
H4c2	P62805	P33992, P25205, P49736	H4c3	P62805	P33992, P25205, P49736
H4c4	P62805	P33992, P25205, P49736	H4c6	P62805	P33992, P25205, P49736
H4c8	P62805	P33992, P25205, P49736	H4c9	P62805	P33992, P25205, P49736
Hdgf	P51858	P78527	Hexb	P07686	P06865
Hmgb1	P63158	P06746	Hmox1	P09601	Q9NUX5
Hnrnpa1	P09651-2	Q92973	Hnrnpa2b1	P22626	P67809
Hnrnpf	P52597	O43347	Hprt1	P00492	Q96G04
Hsp90aa1	P07900	Q15185	Hsp90ab1	P08238	O14757
Hsp90b1	P14625	P11021	Hspa1a	P0DMV8	P13569
Hspa4	P34932	P11142	Hspa5	P11021	P13569
Hspa8	P11142	Q99558	Hspb1	P04792	O00273
Hspd1	P10809	Q16822	Hspe1	P61604	P19320

Input	UniProt Id	Interacts with	Input	UniProt Id	Interacts with
Hspg2	P98160-PRO_0000391621, P98160-PRO_0000391622, P98160	P35968	Htra1	Q92743	P10636-8
Hyou1	Q9Y4L1	P11021	Igfbp4	P22692	P05019
Igfbp6	P24592	P24592	Inhba	P08476	P21674
Ipo5	O00410	P19320	Iqgap1	P46940	P60709
Itga5	P08648	P97333	Itgb1	P05556	P78536
Itm2b	Q9Y287	P04578	Kif5b	Q61768	O35685
Kpnb1	Q14974	Q99558	Krt1	P04264	P19320
Krt10	P13645	P19320	Krt79	Q5XKE5	A1L190
Lama4	Q16363	P04637	Lamc1	P02468	P10493
Lap3	P28838	Q13185	Lasp1	Q14847-2	Q8TDS5
Ldha	P00338	P11142	Ldlr	P01130	Q8NBP7
Lgals1	P09382	P35968	Lgals3	P17931	P09237
Lgals3bp	Q08380	Q9BZR8	Lmna	P02545	Q8NC56
Lox	P28300	Q9UBX5, P15502	Lrp1	Q07954	P02649
Lum	P51884	P50281	Mapk1	P63086	Q63155
Mapre1	Q15691	P11021	Mdh1	P40925	P00533
Mdh2	P40926	P19320	Mif	P14174	P14174
Mmp2	P08253	P16035	Mrc2	Q9UBG0	P02454
Msn	P26038	P16070	Mtap	Q9V813	Q7KNM2
Mtpn	P62775	Q5XI32	Myh9	P35579	O00255
Myl12a	P19105	Q14164	Naca	P38879	P15731
Nap1l1	P55209	Q09472, Q92793	Nap1l4	Q99733	P04608
Nbl1	Q61477	P12023	Ncl	P19338	Q00987
Nedd4	P46934-4	Q9HAU4	Nedd8	Q15843	P68104
Nid2	Q8IV28	P10745	Nme1	P15531	P10911
Nqo1	P15559	P15559	Nrp1	P97333	P08648
Ntf3	P20783	P07174	Nucb1	Q02818	P10823
Nudc	Q9Y266	P53041	Ostf1	Q92882	P42858
P4hb	P07237	Q6PJG9	Pabpc1	P11940	Q92900
Pcbp1	Q15365	P11940	Pcolce	Q15113	P18828
Pcsk9	Q8NBP7	P08253	Pdcd5	O14737	Q9NRD5
Pdcd6ip	Q8WUM4	P17931	Pdia3	P30101	P30304
Pdia6	Q15084	P11021	Pdlim1	O00151	P14136
Pea15	Q15121	P28482	Pebp1	P30086	P16050
Pfn1	P07737	P60709	Pgam1	P18669	P12004
Pgf	P49763	P17948	Pgk1	P00558, P00558-1	O15379
Pkm	P14618-1	Q16665	Pla1a	EBI-3954042	P78527
Plod1	Q02809	P55795	Plod3	O60568	Q9UMD9
Ppia	P62937	O00267	Ppib	P23284	P40855
Ppic	P45877	Q8N9N5	Ppid	Q08752	P63244
Ppp1r7	Q15435	Q6VY07	Ppp2r1a	P30153	P13569
Prdx2	P32119	P10599	Prdx5	P30044	P00441
Prdx6	P30041	Q7KZN9	Prkcsh	P14314	P04578
Prnp	P10279, P04156	P04156	Psma1	P25786	P21673
Psma2	P25787	Q9Y5K5	Psma3	P25788	Q12846
Psma4	P25789	Q16665	Psma5	P28066	Q9Y5K5
Psma6	P60900	Q9Y5K5	Psma7	O14818	Q16665
Psmb1	P20618	Q9HAP6	Psmb2	P49721	Q9Y5K5

Input	UniProt Id	Interacts with	Input	UniProt Id	Interacts with
Psmb3	P49720	Q9Y5K5	Psmb4	P28070	Q9Y5K5
Psmb5	P28074	Q9Y5K5	Psmb6	P28072	Q9Y5K5
Psmb7	Q99436	Q9Y5K5	Psmc2	P35998	P38936
Psmd2	Q13200	Q9Y5K5, Q16186	Ptpa	P18433	P12931
Ptx3	P26022	P09038	Pvr	P15151	P11686
Rab10	P61026	Q96CV9	Rab2a	P61019	Q96FJ0
Rac1	P63000	Q13177	Rack1	P63244	Q14694
Rad23b	P54727	P53350	Ran	P62826	P47813
Rcn1	Q15293	Q969Y2	Rcn3	Q96D15	Q9UMX1
Rdx	P35241	P10911	Rnh1	P13489	P03950
Rpl12	P30050	P27824	Rpl19	P84098	P03372
Rpl24	P83731	Q99558	Rpl4	P36578	Q99558
Rpl5	P47962	P23804	Rpl6	Q02878	Q99558
Rpl8	P62917	Q99558	Rplp0	P05388	P03372
Rplp1	P05386	Q9GZQ8	Rps14	P62263	Q9Y3D8
Rps15a	P62244	Q15843	Rps16	P62249	Q15843
Rps2	O74892	O13648	Rps23	Q8T3U2	O97394
Rps4x	P62701	Q15843	Rps7	P62081	Q00987
Rps8	P62241	Q15843	Rpsa	P08865	P03372
Rsu1	Q15404	P48059	Rtn4	Q9NQC3	P05919
S100a11	P31949	P04271	S100a4	P26447	Q00987
S100a6	P06703	Q02790	Sat2	Q96F10	P21673
Sdcbp	O00560	Q5VTY9	Sdccag8	Q86SQ7	P61088
Sdf4	Q9BRK5	Q13418	Sec23a	Q15436	P48023
Sema7a	O75326	O60486	Septin11	Q9NVA2	Q16181
Serbp1	Q8NC51	P67809	Serpinc1	P01008	P01008
Serpine1	P05121	Q8NBQ5	Serpinf1	P36955	Q9P2X3
Serping1	E9PK97	Q9BRI3	Serpinh1	P50454	P03372
Set	Q01105	P10126	Sod1	P00441	P29692
Sod2	P04179	P04179	Spp1	P10451	P16070
Sptbn1	Q01082	Q13813	Stip1	P31948	P07900
Tars1	P26639	P19320	Tcp1	P17987	Q13882
Tf	P02787	P08034	Tgm2	P21980	P02751
Thbs1	P07996-PRO_0000035842	P16671	Thbs2	Q03350	Q07954
Timp1	P01033	P14780	Timp2	P16035	P08253
Tkt	Q16832	P29353	Tln1	Q9Y490, P26039	P05556
Tmsb10	P63313	Q9NUX5	Tmsb4x	P20065	Q8WWQ8
Tpi1	P60174	P12004	Tpm4	P67936	Q9NRD5
Tpp1	Q96AP0	Q92900	Tpt1	P13693	P29692
Tuba1a	Q71U36	P10636-8	Tuba4a	P68366	P30622
Tubb2a	Q13885	P04183	Tubb4b	P68371	Q5S007
Tubb5	P04350	P22736	Tubb6	Q922F4	Q62108
Txndc5	Q86UY0	O43597	Txnrd1	Q16881	Q03135
Uba1	P22314	Q9UNE7	Ube2d3	P61077	P98170
Ube2i	Q7KZS0	P60709	Ube2n	P61088	P98170
Ugp2	Q16851	O76003	Usp5	P45974-2	Q9NWF9
Vapa	Q9P0L0	P27824	Vasn	Q6EMK4	P22735
Vat1	P54219-3	P29033	Vcam1	P19320	P40429
Vcl	Q64727	Q8VI36	Vcp	P55072	O96017

Input	UniProt Id	Interacts with	Input	UniProt Id	Interacts with
Vegfa	EBI-2895491	Q16665	Vegfd	O43915	Q15836
Vim	P08670	P11021	Vps35	Q9EQH3	P63101
Wdr1	O75083	P62993	Xpnpep1	Q9NQW7-3	P69892
Yars	P54577	P09874	Ybx1	P67809	Q99697
Ywhab	P35213	Q9JLT6	Ywhae	P62258	P30304
Ywhag	P61981	P11940	Ywhah	Q04917	P30304
Ywhaq	P27348	P30304	Ywhaz	P63101	P63017

Input	ChEBI Id	Interacts with	Input	ChEBI Id	Interacts with
Actb	P60709	16851	Actg1	P63261	16851
Actn1	P12814	16851	Actn4	O43707	16851
Angptl4	Q9BY76	28815	Atp1a1	P05023	16618
Cand1	Q86VP6	16851	Cdc42	P60953	15996
Cfl1	P23528	16851	Cltc	Q00610	16618
Dstn	P60981	16618	Ehd1	Q9H4M9	16618
Flna	P21333	16851	Flnb	O75369	16851
Flnc	Q14315	16851	Iqgap1	P46940	16618
Itgb1	P05556	16618	Kif5b	P33176	16851
Kpnb1	Q14974	16851	Ldha	P00338	16851
Lrp1	Q07954	29108	Msn	P26038	16851
Myh9	P35579	16851	Niban2	Q96TA1	17283
Pkm	P14618-1	18319	Rab7a	P51149	16851
Rac1	P63000	15996	Rack1	P63244	16851
Ran	P62826	17552, 15996	Rdx	P35241	16851
Sptbn1	Q01082	16618	Tln1	Q9Y490	18348

7. Identifiers not found

These 46 identifiers were not found neither mapped to any entity in Reactome.

Aars1	Abracl	Aebp1	Angptl2	Anxa3	Ccdc80	Cd248	Clic1
Clic4	Cpxm1	Crip1	Crip2	Dkk3	Dpp3	Eprs1	Fkbp10
GaskbB	Glipr2	Gm20431	Hars1	Heg1	Hint1	Krt90	Ly6a
Map4	Metrnl	Oaf	Olfml3	Pcdhgc3	Pdia4	Postn	Prl2c3
Prl7c1	Ptms	Rars1	Rexo2	Rnase4	Scpep1	Serpina3n	Srpx2
Svep1	Tagln	Tgfbr3	Twf1	Uap1l1	Ufm1		

reactome

BM-MSC secretome from ND mice
Pathway Analysis Report

Table of Contents

1. Introduction
2. Properties
3. Genome-wide overview
4. Most significant pathways
5. Pathways details
6. Identifiers found
7. Identifiers not found

1. Introduction

Reactome is a curated database of pathways and reactions in human biology. Reactions can be considered as pathway 'steps'. Reactome defines a 'reaction' as any event in biology that changes the state of a biological molecule. Binding, activation, translocation, degradation and classical biochemical events involving a catalyst are all reactions. Information in the database is authored by expert biologists, entered and maintained by Reactome’s team of curators and editorial staff. Reactome content frequently cross-references other resources e.g. NCBI, Ensembl, UniProt, KEGG (Gene and Compound), ChEBI, PubMed and GO. Orthologous reactions inferred from annotation for Homo sapiens are available for 17 non-human species including mouse, rat, chicken, puffer fish, worm, fly, yeast, rice, and Arabidopsis. Pathways are represented by simple diagrams following an SBGN-like format.

Reactome's annotated data describe reactions possible if all annotated proteins and small molecules were present and active simultaneously in a cell. By overlaying an experimental dataset on these annotations, a user can perform a pathway over-representation analysis. By overlaying quantitative expression data or time series, a user can visualize the extent of change in affected pathways and its progression. A binomial test is used to calculate the probability shown for each result, and the p-values are corrected for the multiple testing (Benjamini–Hochberg procedure) that arises from evaluating the submitted list of identifiers against every pathway.

To learn more about our Pathway Analysis, please have a look at our relevant publications:

Fabregat A, Sidiropoulos K, Garapati P, Gillespie M, Hausmann K, Haw R, … D’Eustachio P (2016). The reactome pathway knowledgebase. Nucleic Acids Research, 44(D1), D481–D487. https://doi.org/10.1093/nar/gkv1351.

Fabregat A, Sidiropoulos K, Viteri G, Forner O, Marin-Garcia P, Arnau V, … Hermjakob H (2017). Reactome pathway analysis: a high-performance in-memory approach. BMC Bioinformatics, 18.

2. Properties

- This is an **overrepresentation** analysis: A statistical (hypergeometric distribution) test that determines whether certain Reactome pathways are over-represented (enriched) in the submitted data. It answers the question 'Does my list contain more proteins for pathway X than would be expected by chance?' This test produces a probability score, which is corrected for false discovery rate using the Benjamani-Hochberg method.
- 418 out of 456 identifiers in the sample were found in Reactome, where 8662 pathways were hit by at least one of them.
- IntAct interactors were included to increase the analysis background. This greatly increases the size of Reactome pathways, which maximises the chances of matching your submitted identifiers to the expanded pathway, but will include interactors that have not undergone manual curation by Reactome and may include interactors that have no biological significance, or unexplained relevance.
- This report is filtered to show only results for species 'Mus musculus' and resource 'all resources'.
- The unique ID for this analysis (token) is MjAyMDAzMjQxMjEzMThfMzE4NA%3D%3D. This ID is valid for at least 7 days in Reactome's server. Use it to access Reactome services with your data.

3. Genome-wide overview

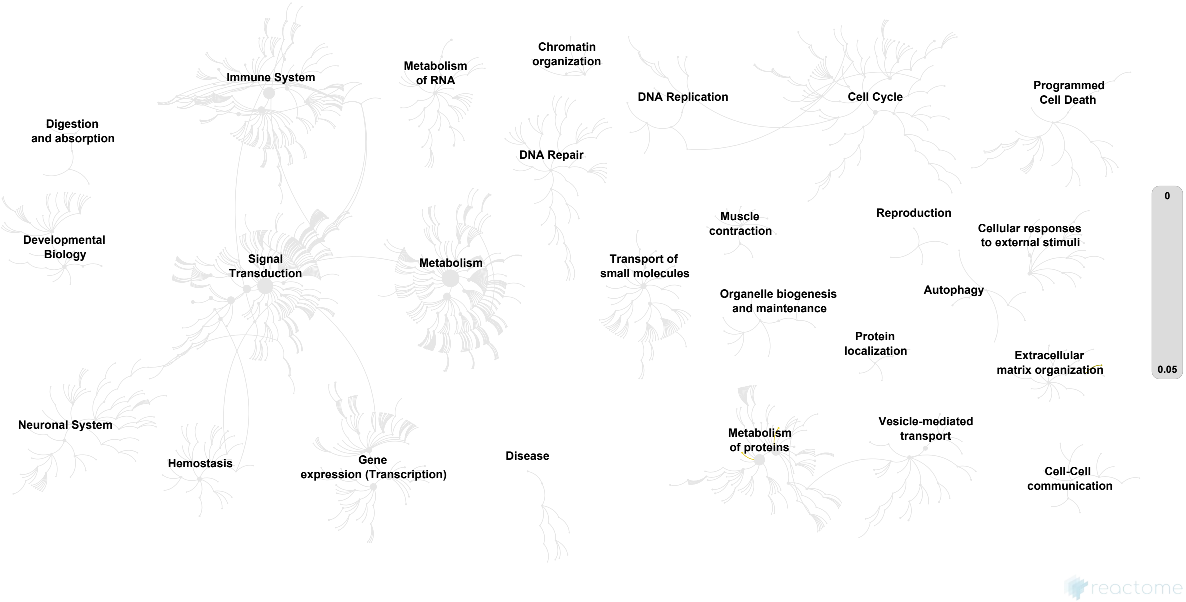

This figure shows a genome-wide overview of the results of your pathway analysis. Reactome pathways are arranged in a hierarchy. The center of each of the circular "bursts" is the root of one top-level pathway, for example "DNA Repair". Each step away from the center represents the next level lower in the pathway hierarchy. The color code denotes over-representation of that pathway in your input dataset. Light grey signifies pathways which are not significantly over-represented.

4. Most significant pathways

The following table shows the 25 most relevant pathways sorted by p-value.

Pathway name	Entities				Reactions	
	found	ratio	p-value	FDR*	found	ratio
Regulation of Insulin-like Growth Factor (IGF) transport and uptake by Insulin-like Growth Factor Binding Proteins (IGFBPs)	42 / 120	0.01	0.009	1	5 / 6	7.69e-04
Post-translational protein phosphorylation	40 / 114	0.009	0.01	1	1 / 1	1.28e-04
Crosslinking of collagen fibrils	11 / 25	0.002	0.037	1	13 / 13	0.002
Antagonism of Activin by Follistatin	3 / 4	3.31e-04	0.07	1	2 / 2	2.56e-04
Laminin interactions	11 / 29	0.002	0.085	1	7 / 8	0.001
Assembly of collagen fibrils and other multimeric structures	22 / 69	0.006	0.098	1	23 / 26	0.003
Chk1/Chk2(Cds1) mediated inactivation of Cyclin B:Cdk1 complex	6 / 14	0.001	0.116	1	1 / 4	5.13e-04
NCAM1 interactions	9 / 24	0.002	0.118	1	1 / 3	3.84e-04
Association of TriC/CCT with target proteins during biosynthesis	5 / 11	9.09e-04	0.12	1	1 / 1	1.28e-04
HSF1 activation	5 / 11	9.09e-04	0.12	1	5 / 6	7.69e-04
Elastic fibre formation	14 / 44	0.004	0.161	1	13 / 16	0.002
ECM proteoglycans	18 / 57	0.005	0.193	1	12 / 15	0.002
Dissolution of Fibrin Clot	6 / 17	0.001	0.214	1	17 / 18	0.002
Activation of C3 and C5	3 / 7	5.79e-04	0.228	1	3 / 3	3.84e-04
Non-integrin membrane-ECM interactions	13 / 42	0.003	0.288	1	5 / 9	0.001
Cross-presentation of soluble exogenous antigens (endosomes)	14 / 51	0.004	0.316	1	4 / 4	5.13e-04
Anchoring fibril formation	5 / 16	0.001	0.324	1	2 / 4	5.13e-04
Molecules associated with elastic fibres	10 / 36	0.003	0.341	1	7 / 9	0.001
Transport of gamma-carboxylated protein precursors from the endoplasmic reticulum to the Golgi apparatus	3 / 9	7.44e-04	0.354	1	3 / 9	0.001
Collagen formation	27 / 106	0.009	0.363	1	52 / 76	0.01
Platelet degranulation	33 / 131	0.011	0.367	1	5 / 8	0.001
TP53 Regulates Transcription of Death Receptors and Ligands	1 / 2	1.65e-04	0.375	1	1 / 1	1.28e-04
Collagen degradation	16 / 62	0.005	0.387	1	15 / 20	0.003

Pathway name	Entities				Reactions	
	found	ratio	p-value	FDR*	found	ratio
Regulation of ornithine decarboxylase (ODC)	13 / 50	0.004	0.395	1	1 / 4	5.13e-04
Keratan sulfate degradation	6 / 22	0.002	0.414	1	3 / 6	7.69e-04

* False Discovery Rate

5. Pathways details

For every pathway of the most significant pathways, we present its diagram, as well as a short summary, its bibliography and the list of inputs found in it.

1. Regulation of Insulin-like Growth Factor (IGF) transport and uptake by Insulin-like Growth Factor Binding Proteins (IGFBPs) (R-MMU-381426)

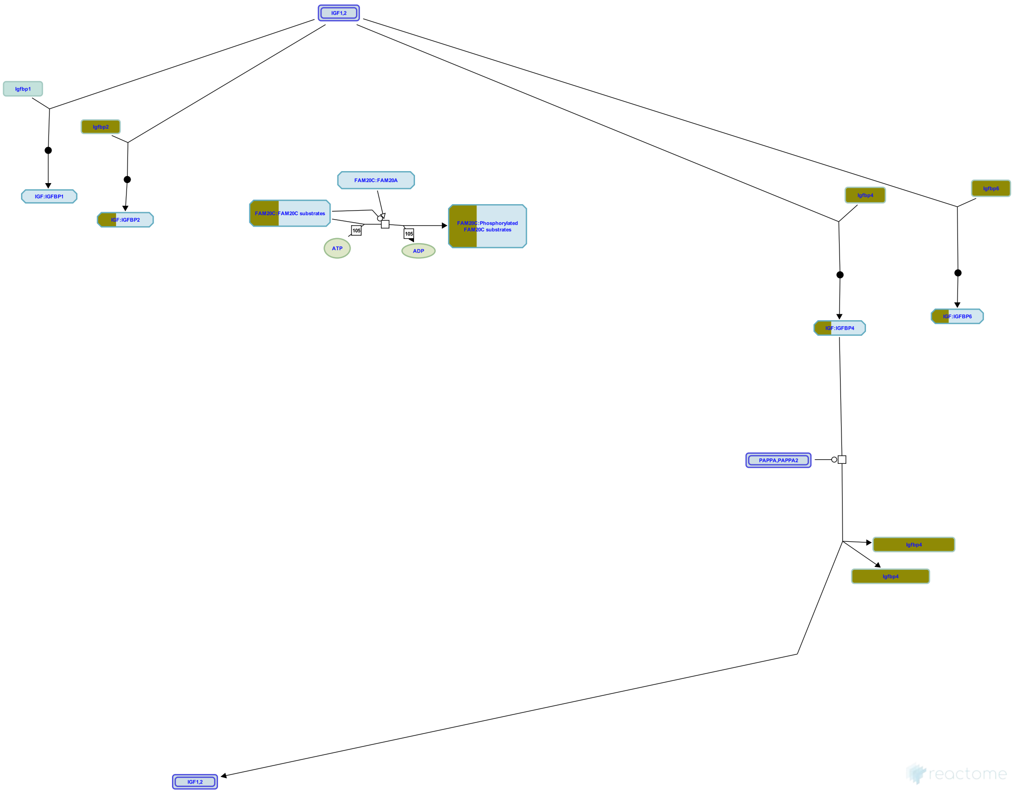

Cellular compartments: extracellular region.

Inferred from: Regulation of Insulin-like Growth Factor (IGF) transport and uptake by Insulin-like Growth Factor Binding Proteins (IGFBPs).

This event has been computationally inferred from an event that has been demonstrated in another species.

The inference is based on the homology mapping from PANTHER. Briefly, reactions for which all involved PhysicalEntities (in input, output and catalyst) have a mapped orthologue/paralogue (for complexes at least 75% of components must have a mapping) are inferred to the other species. High level events are also inferred for these events to allow for easier navigation.

More details and caveats of the event inference in Reactome. For details on PANTHER see also: http://www.pantherdb.org/about.jsp

References

Edit history

Date	Action	Author
2020-03-08	Created	Cook J

Entities found in this pathway (42)

Input	UniProt Id	Input	UniProt Id	Input	UniProt Id
Ahsg	P29699	Alb	P07724	Apoe	P08226
C3	P01027	C4b	P01029	Calu	O35887
Ccn1	P18406	Cdh2	P15116	Cp	Q61147
Csf1	P07141	Cst3	P21460	F5	O88783
Fbn1	Q61554	Fn1	P11276	Fstl1	Q62356
Gas6	Q61592	Hsp90b1	P08113	Igfbp2	P47877
Igfbp3	P47878	Igfbp4	P47879	Igfbp6	P47880
Igfbp7	Q61581	Itih2	Q61703	Lamb1	P02469
Lamb2	Q61292	Lamc1	P02468	Lgals1	P16045
Mfge8	P21956	Nucb1	Q02819	P4hb	P09103
Pcsk9	Q80W65	Pdia6	Q922R8	Prkcsh	O08795
Prss23	Q9D6X6	Qsox1	Q8BND5	Rcn1	Q05186
Serpinc1	P32261	Sparcl1	P70663	Spp1	P10923
Tf	Q921I1	Timp1	P12032	Vcan	Q62059

16. Cross-presentation of soluble exogenous antigens (endosomes) (R-MMU-1236978)

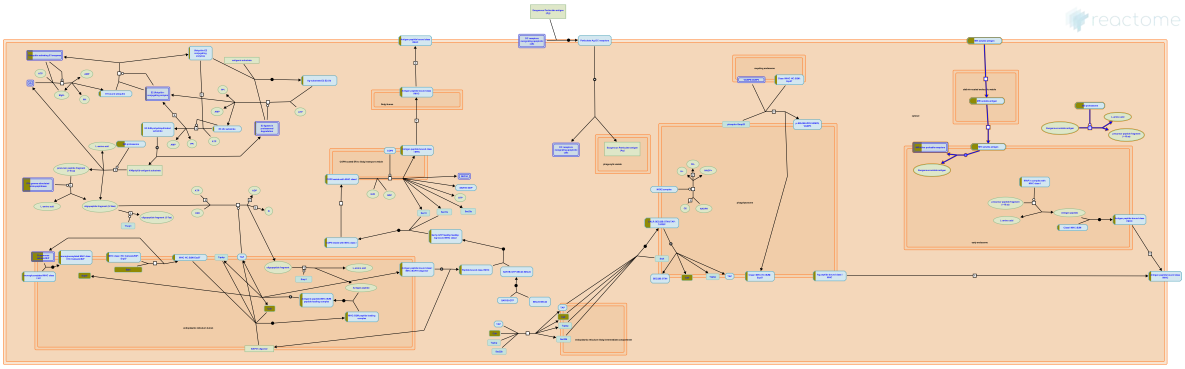

Inferred from: Cross-presentation of soluble exogenous antigens (endosomes).

This event has been computationally inferred from an event that has been demonstrated in another species.

The inference is based on the homology mapping from PANTHER. Briefly, reactions for which all involved PhysicalEntities (in input, output and catalyst) have a mapped orthologue/paralogue (for complexes at least 75% of components must have a mapping) are inferred to the other species. High level events are also inferred for these events to allow for easier navigation.

More details and caveats of the event inference in Reactome. For details on PANTHER see also: http://www.pantherdb.org/about.jsp

References

Edit history

Date	Action	Author
2020-03-08	Created	Cook J

Entities found in this pathway (14)

Input	UniProt Id	Input	UniProt Id	Input	UniProt Id
Mrc2	Q64449	Psma1	Q9R1P4	Psma2	P49722
Psma4	Q9R1P0	Psma5	Q9Z2U1	Psma6	Q9QUM9
Psma7	Q9Z2U0	Psmb2	Q9R1P3	Psmb3	Q9R1P1
Psmb4	P99026	Psmb5	O55234	Psmb6	Q60692
Psmb8	P28063	Psme1	P97371		

6. Identifiers found

Below is a list of the input identifiers that have been found or mapped to an equivalent element in Reactome, classified by resource.

Entities (418)

Input	UniProt Id	Input	UniProt Id	Input	UniProt Id
A2m	P01023	Acta2	P62739	Actb	P60709
Actg1	Z4YHI2	Actr2	P61160	Actr3	F1P679
Adam15	Q13444	Adam9	Q13443	Adamts1	Q9UHI8
Adamts5	Q9UNA0	Adm	F7AC43	Ahsg	P02765
Ak1	P00568	Ak2	P54819	Akr1a1	P14550
Akr1b1	P15121	Alb	P02768	Aldh1a2	O94788
Aldh1a3	P47895	Aldoa	P04075	Anxa2	Q07936
Anxa5	P08758	Apoe	P02649	Apoh	P02749
Arcn1	Q5XJY5	Arg1	P05089	Arhgdia	F1PL93
Arhgdib	P52566	Arpc1b	E2RMT4	Arpc3	E2R985
Asah1	Q13510	Atic	P31335	Atox1	Q9TT99
Atp6ap2	O75787	Axl	P30530	B2m	P61769
B4galt1	P15291	Bag3	Q5U2U8	Bgn	P21810
Blvrb	P30043	Bmp1	P13497	C1qa	E1BSP0
C1qc	Q02105	C1ra	Q8CG16	C3	P01024
C4b	P01029	Calr	P27797	Calu	O43852
Cant1	Q8WVQ1	Cap1	Q01518	Capg	Q9BPX3
Cck	K9J7V7	Ccl2	P13500	Ccl7	Q03366
Ccl8	Q09141	Ccl9	Q5FVN3	Ccn1	O00622
Ccn2	P29279	Cct2	P78371	Cct4	P50991
Cct5	P48643	Cct7	Q99832	Cd14	P08571
Cdh11	P55288	Cdh2	P19022	Cemip	Q8WUJ3
Cfl1	P23528	Ckb	P12277	Cltc	Q00610
Clu	P10909	Cmpk1	P30085	Cndp2	Q96KP4
Cnn2	Q99439	Col1a1	P02452	Col1a2	P08123
Col2a1	P02458	Col3a1	P02461	Col4a1	P02462
Col4a2	P08572	Col5a1	P20908	Col5a2	P05997
Col6a1	P12109	Col6a2	P12110	Col6a3	P12111
Colec12	Q5KU26	Cotl1	Q14019	Cp	P00450
Cpe	P16870	Creg1	O75629	Crlf1	R4GH98
Csf1	P09603	Csrp1	P21291	Cst3	P01034
Cstb	P04080	Ctsa	P10619	Ctsb	P07858
Ctsh	P09668	Ctsl	P07711	Ctss	P25774
Ctsz	Q9UBR2	Cxcl12	Q5EBF6	Cxcl5	P50228
Dag1	Q14118	Dbi	P07108	Dcn	P07585
Ddx39b	Q13838	Des	P17661	Dld	P09622
Dpysl2	P47942	Ecm1	Q16610	Eef1a1	P62630
Eef1b	P24534	Eef1g	P26641	Eef2	P13639
Efemp1	Q12805	Efemp2	O95967	Eif4a1	P60842
Eif5a	P63241	Emilin1	Q9Y6C2	Emilin2	Q9BXX0
Eno1	P06733	Erp44	Q9BS26	Ext1	Q16394

Input	UniProt Id	Input	UniProt Id	Input	UniProt Id
Ext2	Q93063	Ezr	P15311	F2	P00734
F3	P13726	F5	P12259	Fabp5	Q01469
Fbln1	P23142	Fbln2	P98095	Fbln5	Q9UBX5
Fbn1	P35555	Fermt2	F1PFB4	Fkbp1a	P62942
Flna	P21333	Flnb	A0A0G2JXT8	Flnc	E2RP26
Fmod	Q06828	Fn1	P02751	Fscn1	Q16658
Fst	P47931	Fstl1	Q12841	Ftl1	P02793
Gaa	P10253	Ganab	Q14697	Gapdh	P04406
Gas6	Q14393	Gdi2	P50395	Glo1	Q04760
Gm20390	E9PZF0	Gm20547	B8JJN0	Gm2a	P17900
Gmfb	F6WBZ2	Got2	P00505	Gpc1	P35052
Gpc4	O75487	Gpi	P06744	Grn	P28799
Gsn	P06396	Gstm1	A4IFG0, Q9N0V4	Gstm2	F6Q751
Gsto1	P78417	Gstp1	P09211	H2-D1	P14427
H2-Q4	Q8HWB2	H4c1	P62805	H4c11	P62805
H4c12	P62805	H4c14	P62805	H4c2	P62805
H4c3	P62805	H4c4	P62805	H4c6	P62805
H4c8	P62805	H4c9	P62805	H4f16	P62804
Hba	P01966	Hdgf	P51858	Hexa	P06865
Hexb	P07686	Hist1h4m	P62804	Hk1	P19367
Hmox1	P09601	Hnrnpa1	F6XNZ3	Hnrnpa2b1	P22626
Hnrnpa3	F6YRE5	Hnrnpk	P61979	Hp	P00738
Hprt1	P00492	Hsp90aa1	P82995	Hsp90ab1	P34058
Hsp90b1	P14625	Hspa4	O88600	Hspa5	P11021
Hspa8	P11142	Hspd1	A4II42	Hspg2	P98160
Htra1	Q92743	Hyou1	Q9Y4L1	Idh1	O75874
Ifi30	B3SP85	Igfbp2	P47877	Igfbp3	P17936
Igfbp4	P22692	Igfbp6	P47880	Igfbp7	Q16270
Inhba	Q04998	Inhbb	Q04999	Iqgap1	P46940
Islr	O14498	Isyna1	Q9JHU9	Itgb1	P05556
Itih2	P19823	Itih3	Q06033	Itm2b	Q9Y287
Kpnb1	Q14974	Krt1	P04264	Krt2	P35908
Krt5	F1SGG6	Krt76	Q01546	Krt77	Q7Z794
Krt78	Q8N1N4	Krt79	Q5XKE5	Lama2	P24043
Lama4	Q16363	Lamb1	P07942	Lamb2	P11047, P55268
Lamc1	P11047	Lcn2	P80188	Lcp1	P13796
Ldha	P00340	Ldhb	P00337	Lgals1	P09382
Lgals3	P17931	Lgals3bp	Q08380	Lgmn	Q9R0J8
Lmna	P48678-3	Lox	P28300	Loxl1	Q08397
Loxl2	Q9Y4K0	Loxl3	P58215	Lpl	P11151
Lrp1	E1BGJ0	Ltbp2	Q14767	Lum	P51884
Lyz2	P08905	Man2a1	Q16706	Man2b1	O00754
Manf	P55145	Marcks	A0A287BRL8	Mdh1	P40925
Mdh2	P40926	Me1	P48163	Mfap5	Q13361
Mfge8	Q08431	Mif	P14174	Minpp1	Q9UNW1
Mmp12	P39900	Mmp19	Q99542	Mmp2	P08253
Mmp3	P08254	Mrc2	Q9UBG0	Msn	P26038
Mt2	P02798	Myh9	F1MQ37	Myl12a	Q6ZWQ9
Myl6	P60661	Naglu	P54802	Ncl	P09405

Input	UniProt Id	Input	UniProt Id	Input	UniProt Id
Zyx	Q9N675				

Input	Ensembl Id	Input	Ensembl Id	Input	Ensembl Id
Acta2	ENSG00000107796	Actb	ENST00000331789	Anxa2	ENSG00000182718
Apoe	ENSG00000130203	B2m	ENSG00000166710	C1qc	ENSGALP00000007599
Calr	ENSG00000179218	Ccl2	ENSG00000108691	Ccn1	ENSG00000145386
Ccn2	ENSG00000118523	Cfl1	ENSG00000172757	Cnn2	ENSG00000064666
Col1a1	ENSG00000108821	Col1a2	ENSG00000164692	Csf1	ENSG00000184371
Csrp1	ENSG00000159176	Cxcl12	ENSG00000107562	F3	ENSG00000117525
Fn1	ENSG00000115414	Fscn1	ENSG00000075618	Gsto1	ENSG00000148834
Hdgf	ENSG00000143321	Hmox1	ENSG00000100292	Hnrnpa2b1	ENSG00000122566
Hsp90aa1	ENSG00000080824	Hsp90b1	ENSG00000166598	Hspa5	ENSG00000044574
Hspa8	ENSG00000109971	Hspd1	ENSG00000144381	Hyou1	ENSG00000149428
Ifi30	ENSG00000216490	Igfbp3	ENSG00000146674	Igfbp7	ENSG00000163453
Itgb1	ENSG00000150093	Lcn2	ENSG00000148346	Lcp1	ENSG00000136167
Lgals3	ENSG00000131981	Lmna	ENSG00000160789	Lpl	ENSG00000175445
Me1	ENSG00000065833	Mif	ENSG00000240972	Mmp2	ENSG00000087245
Mmp3	ENSG00000149968	Msn	ENSG00000147065	Mt2	ENSG00000125148
Pdia6	ENSG00000143870	Pf4	ENSG00000163737	Ppia	ENSG00000196262
Psmb8	ENSG00000204264	Rbbp4	ENSG00000162521	Rplp0	ENSG00000089157
Serpinb2	ENSG00000197632	Serpine1	ENSG00000106366	Serpinh1	ENSG00000149257
Sod2	ENSG00000112096	Spp1	ENSG00000118785	Stmn1	ENSG00000117632
Tcp1	ENSG00000120438	Tf	ENSG00000117525	Thbs1	ENSG00000137801
Timp1	ENSG00000102265	Tln1	ENSG00000137076	Tpp1	ENSG00000166340
Txnrd1	ENSG00000198431	Vcam1	ENSG00000162692	Vim	ENSG00000026025

Interactors (307)

Input	UniProt Id	Interacts with	Input	UniProt Id	Interacts with
Actb	P60709	P13569	Actg1	P63261	P60709
Actr2	A7MB62	Q95107	Actr3	Q99JY9	P34152
Adam15	Q13444	P12931	Adam9	Q13443	Q99962
Ahnak	Q09666-2	Q5SY16	Ahsg	P12763	P17931
Akap12	Q02952	P00533	Alb	P02768	P02768
Aldoa	P05064	P63101	Anxa2	EBI-8785901, P07355	Q8NBP7
Apoe	P02649	P02649	Apoh	P02749	P08519
Arg1	P05089	P03372	Arhgdia	P52565	P60953
Arpc1b	O15143	O00401	Arpc3	O15145	P48552
Atic	P31939	P00533	Atox1	O00244	P35670
Atp6ap2	O75787	Q15904	Axl	P30530	P19174
B2m	P61769	P61769	B4galt1	P08037	P29752
Bag3	O95817	Q00613	Basp1	Q91XV3	Q62108
Bmp1	P13497-2	P07585	Bmper	Q9W2H2	P18824
C3	P01024-PRO_0000005911, P01024-PRO_0000005908, P01024	P08603, P05156	Calr	P27797	P30101
Calu	O43852	P04049	Cap1	Q13114	Q99558
Capg	P40121	Q8IUQ4	Ccl2	P13500	P13501
Ccn1	P20248	Q9H211	Ccn2	P29279	P02751
Ccn5	O76076	P49639	Cct2	P78371	Q8IWZ6

Input	UniProt Id	Interacts with	Input	UniProt Id	Interacts with
Cct4	P50991	Q13882	Cct5	P48643	Q13418
Cct7	P42943	P26309	Cd14	EBI-11177705, EBI-11177518	O43474
Cdh2	P15116	P18031	Cfl1	P23528	P60709
Cfl2	Q549N0, Q9Y281	P60709	Cltc	Q00610	O14920
Clu	P10909	P30101	Col1a1	P02452	P02751
Col3a1	P02461-1	P09486	Col4a1	P08120	O46339
Col5a1	P20908	P01033, P08253	Colec12	Q5KU26	Q9NV70
Coro1c	Q9ULV4	P19320	Cotl1	Q14019	P09917
Csf1	P07141	P05480	Csrp1	P21291	Q16790
Ctsa	P10619	P08670	Ctsb	P07858	P02760
Cxcl12	P48061	P78556, P13501, O15444, Q99616	Dag1	Q14118	Q14118
Dcn	P07585	P02751	Ddx39b	Q13838	P82979, Q9Y3Y2
Des	P48675	P42930	Dld	O08749	Q9CQV8
Dnpep	Q19087	O17761	Dstn	P60981	P60709
Ecm1	Q16610	Q9NQ94	Eef1a1	P68104	Q9NYA1
Eef1b	P24534	P26641	Eef1g	P26641, P26641-2	P29692, P24534
Eef2	P13639	Q16539	Efemp1	Q12805	P24592
Efemp2	O95967	Q9UBX5, P15502	Eif4a1	P60843	Q6NZJ6
Eif5a	P63242	P63101	Emilin1	Q9Y6C2-2, Q9Y6C2	Q00994
Eno1	P06733	P03496	Erp29	P30040	Q969S0
Ezr	P15311, P31977	Q63155	F3	P13726	P08709
Fbln1	P23142	P02647	Fbln2	P98095	Q9H2F3
Fbln5	Q9UBX5	P15502	Fbn1	P35555	P15502
Fermt2	Q96AC1	P35222	Fkbp1a	P62942	P36896
Flna	P21333	O14786	Flnb	O75369	Q13233
Flnc	Q14315	O75923	Fmod	P13605	P08603
Fn1	P02751	P07585	Fscn1	Q16658	P54278
Fst	P19883	P10599	Fstl1	Q12841	P08571
G3bp1	Q13283	Q04637	Ganab	Q14697	P04578
Gapdh	P04406	P00558	Gdi2	P50395	Q8BMD2
Gpc4	O75487	Q9NRD5	Grn	P28799	P98160
Gsn	P06396	P32121	Gsto1	P78417	P04792
Gstp1	P09211	P22735	H4c1	P62805	P33992, P25205, P49736
H4c11	P62805	P33992, P25205, P49736	H4c12	P62805	P33992, P25205, P49736
H4c14	P62805	P33992, P25205, P49736	H4c2	P62805	P33992, P25205, P49736
H4c3	P62805	P33992, P25205, P49736	H4c4	P62805	P33992, P25205, P49736
H4c6	P62805	P33992, P25205, P49736	H4c8	P62805	P33992, P25205, P49736
H4c9	P62805	P33992, P25205, P49736	Hdgf	P51858	Q07955
Hexb	P07686	P06865	Hk1	P16615-1	P13569
Hmox1	P09601	Q9NUX5	Hnrnpa1	P09651	P22626
Hnrnpa2b1	P22626	P67809	Hnrnpa3	P51991	P09651
Hnrnpab	Q7ZYE9	P26599	Hnrnpk	P61978	P12931
Hp	P00738	P02647	Hprt1	P00492	Q96G04
Hsp90aa1	P07900	P12931	Hsp90ab1	P08238	O14757

Input	UniProt Id	Interacts with	Input	UniProt Id	Interacts with
Hsp90b1	P14625	P11021	Hspa4	P34932	P11142
Hspa5	P11021	P13569	Hspa8	P11142	Q99558
Hspd1	P10809	Q16822	Hspe1	P61604	P19320
Hspg2	P98160-PRO_0000391621, P98160-PRO_0000391622, P98160	P35968	Htra1	Q92743	P10636-8
Hyou1	Q9Y4L1	P11021	Ifi30	P13284	P22735
Igfbp3	P17936	Q86XT9	Igfbp4	P22692	P05019
Igfbp6	P24592	P24592	Inhba	P08476	P21674
Iqgap1	P46940	P60709	Isyna1	Q9NPH2	Q9NUX5
Itgb1	P05556	P78536	Itm2b	Q9Y287	P04578
Kpnb1	Q14974	Q99558	Krt1	P04264	P19320
Krt76	Q01546	O43559	Krt79	Q5XKE5	A1L190
Lama4	Q16363	P04637	Lamc1	P02468	P10493
Lcn2	P80188	P07237	Ldha	P00338	P11142
Ldhb	P07195	P19320	Lgals1	P09382	P35968
Lgals3	P17931	P09237	Lgals3bp	Q08380	Q9BZR8
Lmna	P48678	P31750	Lox	P28300	Q9UBX5, P15502
Lrp1	Q07954	P02649	Lum	P51884	P50281
Lxn	Q9BS40	Q9UQK1	Mdh1	P40925	P00533
Mdh2	P40926	P19320	Me1	P48163	P48163
Mif	P14174	P14174	Mmp2	P08253	P16035
Mrc2	Q9UBG0	P02454	Msn	P26038	P16070
Mtpn	P62775	Q5XI32	Myh9	P35579	O00255
Myl12a	P19105	Q14164	Myl6	P60660	P19320, 18348
Naca	P38879	P15731	Nap1l1	P55209	P52333
Ncl	P19338	Q00987	Nedd4	P46934-4	Q9HAU4
Nedd8	Q15843	Q8TBC4, P61081	Nid2	Q8IV28	P10745
Nme1	P15531	P10911	Npc2	P61916	16113, O15118
Nucb1	Q02818	P10823	Ostf1	Q92882	P42858
P4hb	P07237	P01137	Pabpc1	P11940	Q92900
Pafah1b2	P68402	P43034	Pcbp1	Q15365	P11940
Pcolce	Q15113	P18828	Pcsk9	Q8NBP7	P08253
Pdcd6ip	Q8WUM4	P12931	Pdia3	P30101	P30304
Pdia6	Q15084	P11021	Pdlim1	O00151	P14136
Pebp1	P30086	P16050	Pf4	P02776-PRO_0000351217, P02776	P78556, P13501, O15444, Q99616, P48061
Pfn1	P07737	P60709	Pgam1	P18669	P12004
Pgk1	P00558, P00558-1	O15379	Pkm	P14618-1	Q16665
Pla1a	EBI-3954042	P78527	Pld3	Q8IV08	P19838
Plod1	Q02809	P55795	Plod3	O60568	Q9UMD9
Ppia	P62937	O00267	Ppib	P23284	P40855
Ppic	P45877	Q8N9N5	Ppid	Q08752	P63244
Ppt1	P53043	P02829	Prdx2	P32119	P10599
Prdx6	P30041	P21796	Prkcsh	P14314	P04578
Prnp	P10279, P04156	P04156	Psma1	P25786	P21673
Psma2	P25787	Q9Y5K5	Psma4	P25789	Q16665
Psma5	P28066	Q9Y5K5	Psma6	P60900	Q9Y5K5
Psma7	O14818	Q16665	Psmb2	P49721	Q9Y5K5
Psmb3	P49720	Q9Y5K5	Psmb4	P28070	Q9Y5K5

Input	UniProt Id	Interacts with	Input	UniProt Id	Interacts with
Psmb5	P28074	Q9Y5K5	Psmb6	P28072	Q9Y5K5
Psmb8	P28062-2	Q13114	Psme1	Q06323	Q9UL46
Ptx3	P26022	P09038	Rack1	P63244	Q14694
Rad23b	P54727	Q92681	Rbbp4	Q09028	P46531
Rcn1	Q15293	Q969Y2	Rcn3	Q96D15	Q9UMX1
Rdx	P35241	P10911	Rnh1	P13489	P03950
Rpl10a	P62906	Q99558	Rpl12	P30050	P27824
Rpl24	P83731	Q99558	Rpl4	P36578	Q99558
Rpl5	P47962	P23804	Rpl8	P62917	Q99558
Rplp0	P05388	P03372	Rplp1	P05386	Q9GZQ8
Rps12	P25398	P03372	Rps14	P62263	Q9Y3D8
Rps8	P62241	Q15843	Rpsa	P08865	P03372
Rsu1	Q15404	Q13418	S100a11	P31949	P04271
S100a4	P26447	Q00987	S100a6	P06703	Q00987
Sdcbp	O00560	P24592	Sdf4	Q9BRK5	Q13418
Septin11	Q8C1B7	Q62108	Septin2	B7ZR20	Q6P5F9
Serpinc1	P01008	P01008	Serpine1	P05121	Q8NBQ5
Serpinf1	P36955	Q9P2X3	Serping1	E9PK97	Q9BRI3
Serpinh1	P50454	P03372	Slc9a3r1	O14745	P13569
Sod2	P04179	P04179	Spp1	P10451	P16070
Sptbn1	Q01082	Q13813	Ssc5d	A1L4H1	Q13526
Stip1	P31948	P07900	Stmn1	P16949	P46527
Sytl4	Q96C24	Q86TI0	Tcp1	P17987	Q13882
Tf	P02787	O00501	Tgm2	P21980	P02751
Thbs1	P07996-PRO_0000035842	P16671	Thbs2	Q03350	Q07954
Timp1	P01033	P14780	Timp2	P16035	P08253
Tinagl1	Q9GZM7	O95429	Tkt	Q16832	P29353
Tln1	Q9Y490, P26039	P05556	Tmsb4x	P20065	Q8WWQ8
Tpi1	P60174	P12004	Tpm4	P67936	Q9NRD5
Tpp1	Q96AP0	Q92900	Tpt1	P13693	P60709
Trim28	Q62318	P12813	Ttr	P02766	Q15109
Tubb4b	P68371	Q5S007	Tubb5	P04350	P22736
Txn	P10599	P19883	Txnrd1	Q16881	Q03135
U2af2	P26368	P82979	Uba1	P22314	Q9UNE7
Ube2n	P61088	P98170	Vasn	Q6EMK4	P22735
Vat1	P54219-3	Q9Y5Y5	Vcam1	P19320	P40429
Vcan	P13611	P14780	Vcl	Q64727	Q8VI36
Vcp	P55072	O96017	Vegfd	O43915	Q15836
Vim	P08670	P11021	Wdr1	O75083	P62993
Ybx1	P67809	Q99697	Ywhab	P35213	Q9JLT6
Ywhae	P62258	Q92934	Ywhag	P61981	Q92934
Ywhah	Q04917	Q92934	Ywhaq	P27348	Q92934
Ywhaz	P63104	Q92934	Zyx	Q15942	P02751

Input	ChEBI Id	Interacts with	Input	ChEBI Id	Interacts with
Actb	P60709	18348	Actg1	P63261	18348
Cfl1	P23528	18348	Cltc	Q00610	16618
Dstn	P60981	16618	Ezr	P15311	18348
Flna	P21333	18348	Flnb	O75369	18348
Flnc	Q14315	18348	Igfbp3	P17936	16336
Iqgap1	P46940	16618	Itgb1	P05556	16618

Input	ChEBI Id	Interacts with
Kpnb1	Q14974	18348
Lrp1	Q07954	29108
Myh9	P35579	18348
Niban2	Q96TA1	17283
Pkm	P14618-1	18319
Rdx	P35241	16851
Sptbn1	Q01082	16618
Trim28	Q13263	18348

Input	ChEBI Id	Interacts with
Ldha	P00338	18348
Msn	P26038	18348
Myl6	P60660	18348
Npc2	P61916	16113
Rack1	P63244	18348
Septin2	Q15019	18348
Tln1	Q9Y490	18348

7. Identifiers not found

These 38 identifiers were not found neither mapped to any entity in Reactome.

Aebp1	Angptl2	Anxa3	C1s1	Ccdc80	Cd248	Cpq	Cpxm1
Ctla2a	Dpp3	Fkbp10	Fkbp2	Fndc1	Gm20431	Gm7324	Gm9780
Hars1	Heg1	Hint1	Man1a	Marcksl1	Metrnl	Oaf	Olfml2b
Pdgfrl	Pdia4	Prl7c1	Ptms	Rexo2	Rnase4	Sbsn	Serpina3n
Srpx	Srpx2	Svep1	Tagln	Tgfbr3	Twf1		

vWAT-MSC secretome from HFD mice

Pathway Analysis Report

Table of Contents

1. Introduction
2. Properties
3. Genome-wide overview
4. Most significant pathways
5. Pathways details
6. Identifiers found
7. Identifiers not found

1. Introduction

Reactome is a curated database of pathways and reactions in human biology. Reactions can be considered as pathway 'steps'. Reactome defines a 'reaction' as any event in biology that changes the state of a biological molecule. Binding, activation, translocation, degradation and classical biochemical events involving a catalyst are all reactions. Information in the database is authored by expert biologists, entered and maintained by Reactome's team of curators and editorial staff. Reactome content frequently cross-references other resources e.g. NCBI, Ensembl, UniProt, KEGG (Gene and Compound), ChEBI, PubMed and GO. Orthologous reactions inferred from annotation for Homo sapiens are available for 17 non-human species including mouse, rat, chicken, puffer fish, worm, fly, yeast, rice, and Arabidopsis. Pathways are represented by simple diagrams following an SBGN-like format.

Reactome's annotated data describe reactions possible if all annotated proteins and small molecules were present and active simultaneously in a cell. By overlaying an experimental dataset on these annotations, a user can perform a pathway over-representation analysis. By overlaying quantitative expression data or time series, a user can visualize the extent of change in affected pathways and its progression. A binomial test is used to calculate the probability shown for each result, and the p-values are corrected for the multiple testing (Benjamini–Hochberg procedure) that arises from evaluating the submitted list of identifiers against every pathway.

To learn more about our Pathway Analysis, please have a look at our relevant publications:

Fabregat A, Sidiropoulos K, Garapati P, Gillespie M, Hausmann K, Haw R, … D'Eustachio P (2016). The reactome pathway knowledgebase. Nucleic Acids Research, 44(D1), D481–D487. https://doi.org/10.1093/nar/gkv1351.

Fabregat A, Sidiropoulos K, Viteri G, Forner O, Marin-Garcia P, Arnau V, … Hermjakob H (2017). Reactome pathway analysis: a high-performance in-memory approach. BMC Bioinformatics, 18.

2. Properties

- This is an **overrepresentation** analysis: A statistical (hypergeometric distribution) test that determines whether certain Reactome pathways are over-represented (enriched) in the submitted data. It answers the question 'Does my list contain more proteins for pathway X than would be expected by chance?' This test produces a probability score, which is corrected for false discovery rate using the Benjamani-Hochberg method.
- 335 out of 354 identifiers in the sample were found in Reactome, where 8330 pathways were hit by at least one of them.
- IntAct interactors were included to increase the analysis background. This greatly increases the size of Reactome pathways, which maximises the chances of matching your submitted identifiers to the expanded pathway, but will include interactors that have not undergone manual curation by Reactome and may include interactors that have no biological significance, or unexplained relevance.
- This report is filtered to show only results for species 'Mus musculus' and resource 'all resources'.
- The unique ID for this analysis (token) is MjAyMDAzMjQxMjU5NDFfMzIwMg%3D%3D. This ID is valid for at least 7 days in Reactome's server. Use it to access Reactome services with your data.

3. Genome-wide overview

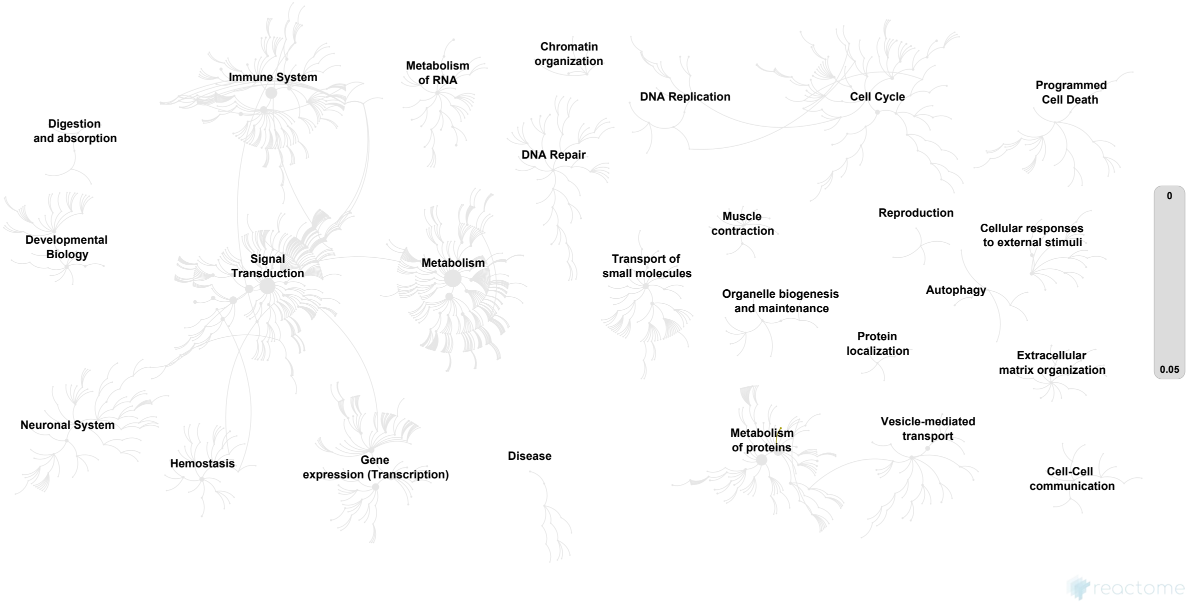

This figure shows a genome-wide overview of the results of your pathway analysis. Reactome pathways are arranged in a hierarchy. The center of each of the circular "bursts" is the root of one top-level pathway, for example "DNA Repair". Each step away from the center represents the next level lower in the pathway hierarchy. The color code denotes over-representation of that pathway in your input dataset. Light grey signifies pathways which are not significantly over-represented.

4. Most significant pathways

The following table shows the 25 most relevant pathways sorted by p-value.

Pathway name	Entities				Reactions	
	found	ratio	p-value	FDR*	found	ratio
Post-translational protein phosphorylation	30 / 114	0.009	0.044	1	1 / 1	1.28e-04
Chk1/Chk2(Cds1) mediated inactivation of Cyclin B:Cdk1 complex	6 / 14	0.001	0.051	1	1 / 4	5.13e-04
Regulation of ornithine decarboxylase (ODC)	15 / 50	0.004	0.054	1	1 / 4	5.13e-04
HSF1 activation	5 / 11	9.09e-04	0.058	1	5 / 6	7.69e-04
Cross-presentation of soluble exogenous antigens (endosomes)	15 / 51	0.004	0.062	1	1 / 4	5.13e-04
Regulation of RUNX2 expression and activity	15 / 52	0.004	0.07	1	1 / 5	6.41e-04
Regulation of Insulin-like Growth Factor (IGF) transport and uptake by Insulin-like Growth Factor Binding Proteins (IGFBPs)	30 / 120	0.01	0.073	1	3 / 6	7.69e-04
Ubiquitin-dependent degradation of Cyclin D	15 / 53	0.004	0.079	1	1 / 3	3.84e-04
p53-Independent DNA Damage Response	15 / 53	0.004	0.079	1	1 / 3	3.84e-04
Ubiquitin Mediated Degradation of Phosphorylated Cdc25A	15 / 53	0.004	0.079	1	1 / 3	3.84e-04
p53-Independent G1/S DNA damage checkpoint	15 / 53	0.004	0.079	1	1 / 3	3.84e-04
NCAM1 interactions	8 / 24	0.002	0.086	1	1 / 3	3.84e-04
Autodegradation of the E3 ubiquitin ligase COP1	15 / 54	0.004	0.089	1	1 / 5	6.41e-04
Crosslinking of collagen fibrils	8 / 25	0.002	0.102	1	13 / 13	0.002
Collagen degradation	16 / 62	0.005	0.128	1	15 / 20	0.003
Activation of C3 and C5	3 / 7	5.79e-04	0.145	1	3 / 3	3.84e-04
CDT1 association with the CDC6:ORC:origin complex	15 / 59	0.005	0.149	1	1 / 3	3.84e-04
Degradation of AXIN	15 / 59	0.005	0.149	1	2 / 8	0.001
Dectin-1 mediated noncanonical NF-kB signaling	15 / 59	0.005	0.149	1	1 / 8	0.001
NIK-->noncanonical NF-kB signaling	15 / 59	0.005	0.149	1	1 / 9	0.001
AUF1 (hnRNP D0) binds and destabilizes mRNA	17 / 64	0.005	0.154	1	3 / 4	5.13e-04

Pathway name	Entities				Reactions	
	found	ratio	p-value	FDR*	found	ratio
Assembly of collagen fibrils and other multimeric structures	17 / 69	0.006	0.158	1	23 / 26	0.003
Degradation of GLI1 by the proteasome	16 / 61	0.005	0.178	1	2 / 5	6.41e-04
Anchoring fibril formation	5 / 16	0.001	0.184	1	2 / 4	5.13e-04
FBXL7 down-regulates AURKA during mitotic entry and in early mitosis	16 / 62	0.005	0.193	1	3 / 6	7.69e-04

* False Discovery Rate

7. Regulation of Insulin-like Growth Factor (IGF) transport and uptake by Insulin-like Growth Factor Binding Proteins (IGFBPs) (R-MMU-381426)

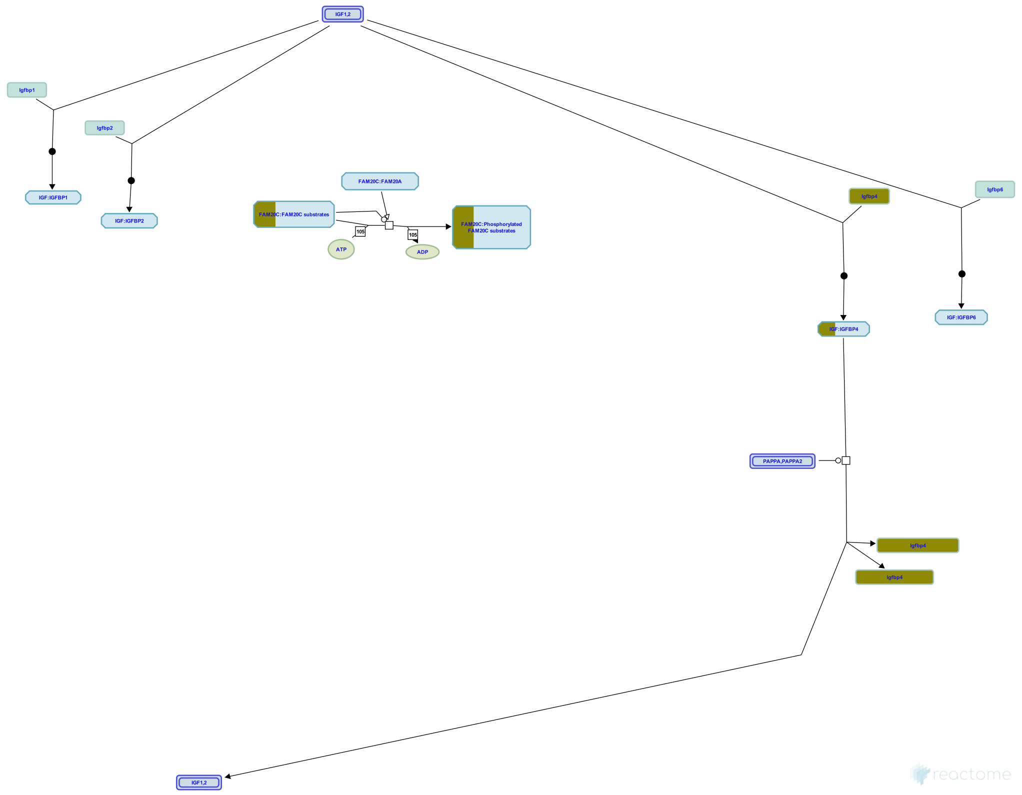

Cellular compartments: extracellular region.

Inferred from: Regulation of Insulin-like Growth Factor (IGF) transport and uptake by Insulin-like Growth Factor Binding Proteins (IGFBPs).

This event has been computationally inferred from an event that has been demonstrated in another species.

The inference is based on the homology mapping from PANTHER. Briefly, reactions for which all involved PhysicalEntities (in input, output and catalyst) have a mapped orthologue/paralogue (for complexes at least 75% of components must have a mapping) are inferred to the other species. High level events are also inferred for these events to allow for easier navigation.

More details and caveats of the event inference in Reactome. For details on PANTHER see also: http://www.pantherdb.org/about.jsp

References

Edit history

Date	Action	Author
2020-03-08	Created	Cook J

Entities found in this pathway (30)

Input	UniProt Id	Input	UniProt Id	Input	UniProt Id
Alb	P07724	Apoe	P08226	C3	P01027

Input	UniProt Id	Input	UniProt Id	Input	UniProt Id
C4b	P01029	Calu	O35887	Ccn1	P18406
Cdh2	P15116	Csf1	P07141	Cst3	P21460
Fbn1	Q61554	Fstl1	Q62356	Gas6	Q61592
Hsp90b1	P08113	Igfbp4	P47879	Igfbp7	Q61581
Itih2	Q61703	Lamb1	P02469	Lamc1	P02468
Lgals1	P16045	Mfge8	P21956	Nucb1	Q02819
P4hb	P09103	Pdia6	Q922R8	Prss23	Q9D6X6
Qsox1	Q8BND5	Rcn1	Q05186	Serpinc1	P32261
Spp1	P10923	Tf	Q921I1	Timp1	P12032

6. Identifiers found

Below is a list of the input identifiers that have been found or mapped to an equivalent element in Reactome, classified by resource.

Entities (335)

Input	UniProt Id	Input	UniProt Id	Input	UniProt Id
A2m	P01023	Acta2	P62737	Actb	P60711
Actg1	P63259	Actn1	P12814	Actr3	F1P679
Adam9	Q13443	Adss2	P30520	Ahcy	P23526
Ak2	P54819	Akr1a1	P14550	Akr1b1	P15121
Alb	P02768	Aldoa	P04075	Anxa1	P07150
Anxa2	P07355	Anxa5	P08758	Anxa6	P08133
Apex1	P28352	Apoe	P02649	Apoh	P02749
Arcn1	Q5XJY5	Arhgdia	F1PL93	Arhgdib	P52566
Arpc1b	E2RMT4	Arpc2	F1PAG6	Arpc4	E2QWU0
Arpc5	O15511	Atic	P31939	Atox1	Q9TT99
Atp5f1b	J9NT37	Atp6ap1	Q15904	B2m	P61769
Bgn	P21810	Blvrb	P30043	Bmp1	P13497
C1qa	E1BSP0	C1qb	F1NH19	C1qc	P02747
C1ra	Q8CG16	C3	P01024	C4b	P01029
Calr	P27797	Calu	O43852	Cap1	Q01518
Capg	Q9BPX3	Cbx3	Q13185	Ccl7	Q03366
Ccl9	Q5FVN3	Ccn1	O00622	Ccn2	P29279
Cd14	P08571	Cdh11	P55288	Cdh2	P19022
Cenpl	Q8N0S6	Cfl1	P23528	Cfp	P27918
Ckb	P12277	Cltb	P09497	Cmpk1	P30085
Cndp2	Q96KP4	Cnn2	Q99439	Col1a1	P02452
Col1a2	P08123	Col2a1	P02458	Col3a1	P02461
Col4a1	P02462	Col4a2	P08572	Col5a2	P05997
Col6a1	P12109	Col6a2	P12110	Col6a3	P12111
Cotl1	Q14019	Cpe	P16870	Creg1	O75629
Csf1	P09603	Csf1r	A7Z067	Csrp1	P21291
Cst3	P01034	Cstb	P04080	Ctsa	P10619
Ctsb	P07858	Ctsd	P07339	Ctsl	P07711
Ctss	P25774	Ctsz	Q9UBR2	Cycs	P99999
Dag1	Q14118	Dbi	P07108	Ddx39b	Q13838
Dld	P09622	Dpysl2	P47942	Dpysl3	Q62952
Ecm1	Q16610	Eef1a1	P62630	Eef1b	P24534
Eef1d	P29692	Eef1g	P26641	Eef2	P13639
Efemp2	O95967	Eif5a	P63241	Eno1	P06733
Fabp4	P04117	Fabp5	Q01469	Fah	P16930
Fam3c	Q92520	Fbln1	P23142	Fbln2	P98095
Fbln5	Q9UBX5	Fbn1	P35555	Fkbp1a	P62942
Flnb	A0A0G2JXT8	Flnc	D3ZHA0	Fscn1	Q16658
Fstl1	Q12841	Fth1	P02794	Ftl1	P02793
Gapdh	P04406	Gas6	Q14393	Gdi2	P50395
Glo1	Q04760	Glud1	P26443	Gm20390	E9PZF0

Input	UniProt Id	Input	UniProt Id	Input	UniProt Id
Gm2a	P17900	Got1	P17174	Got2	P00505
Gpc1	P35052	Gpi	P06744	Grn	P28799
Grpel1	Q9HAV7	Gsn	P06396	Gsr	P47791
Gstm2	F6Q751	Gstp1	P09211	Gzme	P08884
H2-D1	P14427	H3f3a	P84243	H4c1	P62805
H4c11	P62805	H4c12	P62805	H4c14	P62805
H4c2	P62805	H4c3	P62805	H4c4	P62805
H4c6	P62805	H4c8	P62805	H4c9	P62805
H4f16	P62806	Hba	P01966	Hdgf	P51858
Hist1h4m	P62806	Hmgb1	P09429	Hmox1	P09601
Hnrnpa1	P04256	Hnrnpa2b1	P22626	Hnrnpf	Q794E4
Hnrnpu	Q6IMY8	Hp	P00738	Hprt1	P00492
Hsp90aa1	P82995	Hsp90ab1	P34058	Hsp90b1	P14625
Hspa4	O88600	Hspa5	P11021	Hspa8	P11142
Htra1	Q92743	Igfbp4	P22692	Igfbp7	Q16270
Iqgap1	P46940	Itgb1	P05556	Itgb2	P05107
Itih2	P19823	Itm2b	Q9Y287	Kpnb1	Q14974
Krt1	P04264	Krt2	G3MZ71	Krt5	F1SGG6
Krt77	G3MYU2	Krt78	A6QNX5	Krt79	Q148H7
Lamb1	P07942	Lamc1	P11047	Lcp1	P13796
Ldha	P00340	Ldhb	P00337	Lgals1	P09382
Lgals3	P17931	Lgals3bp	Q08380	Lgmn	Q9R0J8
Lmna	P48678-3	Lox	P28300	Loxl2	Q9Y4K0
Loxl3	P58215	Lpl	P11151	Lrp1	E1BGJ0
Lum	P51884	Lyz2	P08905	Man2b1	O00754
Manf	P55145	Marcks	A0A287BRL8	Mdh1	P40925
Mdh2	P40926	Mfge8	Q08431	Mif	P14174
Mmp12	P39900	Mmp2	P08253	Msn	Q9W002
Mt2	P02798	Mydgf	Q969H8	Myh9	F1MQ37
Myl12a	Q6ZWQ9	Myl6	Q60605	Ncl	P09405
Nedd8	Q71UE8	Nid1	P14543	Nid2	Q14112
Nme1	P15531	Npc2	P61916	Npepps	P55786
Npm1	P13084	Nucb1	Q02818	Ogn	P20774
P4hb	P07237	Pdap1	Q13442	Pdcd6ip	Q8WUM4
Pdia3	F6WRR4	Pdia6	Q15084	Pdxk	O00764
Pea15	Q15121	Pebp1	P30086	Pfn1	P07737
Pgam1	P18669	Pgd	P52209	Pgk1	P00558
Pgls	O95336	Pgm1	P36871	Pkm	P14618
Pla2g7	Q5RHM0	Plg	P00747	Plod1	Q02809
Plod3	O60568	Pnp	P00491	Ppia	P62937
Ppib	P23284	Prdx1	P35700	Prdx2	Q61171
Prdx5	P99029	Prdx6	P30041	Prss1	Q9Z1R9
Prss23	O95084	Psap	P07602	Psma1	P25786
Psma2	P25787	Psma3	P25788	Psma4	P25789
Psma5	P28066	Psma6	P60900	Psma7	O14818
Psmb1	P20618	Psmb2	P49721	Psmb3	P49720
Psmb4	P28070	Psmb5	P28074	Psmb6	P28072
Psmb8	P28062	Psme1	Q06323	Ptgr1	Q14914
Ptx3	P26022	Pygl	P06737	Qsox1	O00391

Input	UniProt Id	Input	UniProt Id	Input	UniProt Id
Rad23b	Q4KMA2	Rbbp4	Q09028	Rcn1	Q15293
Rdx	P35241	Rpl12	P30050	Rpl19	P84098
Rpl5	P46777	Rpl8	P62917	Rplp1	P05386
Rplp2	P05387	Rps12	P25398	Rps14	P62263
Rps21	P63220	Rps28	P62857	Rps5	P46782
Rps8	P62241	Rpsa	P08865	Rsu1	D4A8F2
S100a11	P31949	Sdcbp	O00560	Serpinb6a	Q6P9U0
Serpinc1	P01008	Serpine1	P05121	Serpine2	A0A287AYW8
Serping1	P05155	Serpinh1	P50454	Sfpq	A0A0G2K8K0
Sod1	P00441	Sod2	P09671	Sod3	O09164
Spp1	P10451	Sptbn1	Q62261	Stip1	P31948
Tagln2	P37802	Taldo1	P37837	Tcn2	P20062
Tf	P02787	Tgfbi	Q15582	Thbs1	P07996
Thbs2	P35442	Timp1	P01033	Timp2	P16035
Tkt	P29401	Tln1	Q9Y490	Tmsb4x	P62328
Tpi1	P60174	Tpm4	Q6IRU2	Tubb4b	P68371
Tubb5	P07437	Txn	P10639	Txnrd1	Q16881
Uba1	Q02053	Ube2n	P61088	Vat1	Q99536
Vcl	P18206	Vcp	P55072	Vim	P02543
Wdr1	O75083	Ybx1	P62960	Ywhab	Q5XGC8
Ywhae	P62260	Ywhag	Q0V9W8	Ywhah	Q28DR3
Ywhaq	Q5BL40	Ywhaz	Q6P4Z5		

Input	Ensembl Id	Input	Ensembl Id	Input	Ensembl Id
Acta2	ENSG00000107796	Actb	ENST00000331789	Anxa1	ENSG00000135046
Anxa2	ENSG00000182718	Apoe	ENSG00000130203	Atp5f1b	ENSG00000110955
B2m	ENSG00000166710	C1qc	ENSGALP00000007599	Calr	ENSG00000179218
Ccn1	ENSG00000145386	Ccn2	ENSG00000118523	Cfl1	ENSG00000172757
Cnn2	ENSG00000064666	Col1a1	ENSG00000108821	Col1a2	ENSG00000164692
Csf1	ENSG00000184371	Csf1r	ENSG00000182578	Csrp1	ENSG00000159176
Ctsd	ENSG00000117984	Cycs	ENSG00000172115	Fabp4	ENSG00000170323
Fscn1	ENSG00000075618	Fth1	ENST00000273550	Hdgf	ENSG00000143321
Hmox1	ENSG00000100292	Hnrnpa2b1	ENSG00000122566	Hnrnpf	ENSG00000169813
Hsp90aa1	ENSG00000080824	Hsp90b1	ENSG00000166598	Hspa5	ENSG00000044574
Hspa8	ENSG00000109971	Igfbp7	ENSG00000163453	Itgb1	ENSG00000150093
Itgb2	ENSG00000160255	Lcp1	ENSG00000136167	Lgals3	ENSG00000131981
Lmna	ENSG00000160789	Lpl	ENSG00000175445	Mif	ENSG00000240972
Mmp2	ENSG00000087245	Msn	ENSG00000147065	Mt2	ENSG00000125148
Mydgf	ENSG00000074842	Pdia6	ENSG00000143870	Ppia	ENSG00000196262
Psap	ENSG00000122852, ENSG00000185303	Psmb8	ENSG00000204264	Rbbp4	ENSG00000162521
Serpine1	ENSG00000106366	Serpinh1	ENSG00000149257	Sod1	ENSG00000142168
Sod2	ENSG00000112096	Spp1	ENSG00000118785	Taldo1	ENSG00000177156
Tf	ENSG00000117525	Thbs1	ENSG00000137801	Timp1	ENSG00000102265
Tln1	ENSG00000137076	Txnrd1	ENSG00000198431	Vim	ENSG00000026025

Interactors (258)

Input	UniProt Id	Interacts with	Input	UniProt Id	Interacts with
Actb	P60709	P13569	Actg1	P63261	P23528
Actn1	P12814	P12931	Actr3	Q99JY9	P34152

Input	UniProt Id	Interacts with	Input	UniProt Id	Interacts with
Adam9	Q13443	Q99962	Ahcy	P23526	Q8IWL3
Ahnak	Q09666-2	Q5SY16	Ahnak2	Q8IVF2-3	P24593
Akap12	Q02952	P00533	Alb	P02768	P02768
Aldoa	P05064	P63101	Anxa1	P04083	Q13546
Anxa2	EBI-8785901, P07355	Q8NBP7	Anxa6	P14824	P63101
Apex1	P27695	Q09472	Apoe	P02649	P02649
Apoh	P02749	P08519	Arhgdia	P52565	P60953
Arpc1b	O15143	O00401	Arpc4	P59998	O00401
Arpc5	O15511	P00533	Atic	P31939	P00533
Atox1	O00244	P35670	Atp5f1b	P06576	P05919
Atp6ap1	Q15904	P27824	B2m	P61769	P61769
Basp1	Q91XV3	Q62108	Bmp1	P13497-2	P07585
C3	P01024-PRO_0000005911, P01024-PRO_0000005908, P01024	P08603, P05156	Calr	P27797	P04792
Calu	O43852	P04049	Cap1	Q13114	Q99558
Capg	P40121	Q8IUQ4	Caprin1	Q14444	P03372
Cbx3	Q13185	Q14739	Ccn1	P20248	Q9H211
Ccn2	P29279	P02751	Cd14	EBI-11177705, EBI-11177518	O43474
Cdh2	P15116	P18031	Cenpl	P38265	P14136
Cfl1	P45592	P63102	Cfl2	Q549N0	P23528
Cfp	P27918	O75344	Col1a1	P02452	P02751
Col3a1	P02461-1	P09486	Col4a1	P08120	O46339
Coro1c	Q9ULV4	P19320	Cotl1	Q14019	P09917
Csf1	P07141	P05480	Csf1r	P07333	Q8CIH5
Csrp1	P21291	Q16790	Ctsa	P10619	P08670
Ctsb	P07858	P02760	Ctsd	P07339	P04578
Cycs	P99999	Q9Y4K3	Dag1	Q14118	Q14118
Dbn1	Q16643	P60484	Ddx39b	Q13838	P82979, Q9Y3Y2
Dld	O08749	Q9CQV8	Dnpep	Q19087	O17761
Dstn	P60981	P60709	Ecm1	Q16610	Q9NQ94
Eef1a1	P68104	Q00987	Eef1b	P24534	P26641
Eef1d	P29692	P26641, P29692	Eef1g	P26641, P26641-2	P29692, P24534
Eef2	P58252	P63101	Efemp2	O95967	Q9UBX5, P15502
Eif5a	P63242	P63101	Eno1	P06733	P03496
Erp29	P30040	Q969S0	Fabp4	P15090	P08670
Fah	P16930	P00533	Fam3c	Q92520	Q15223
Fbln1	P23142	P02647	Fbln2	P98095	Q9H2F3
Fbln5	Q9UBX5	P15502	Fbn1	P35555	P15502
Fkbp1a	P62942	P36896	Flnb	O75369	Q13233
Flnc	Q14315	O75923	Fscn1	Q16658	P54278
Fstl1	Q12841	P08571	Fth1	P02794	P04792
Gapdh	P04406	P00558	Gdi2	P50395	Q8BMD2
Got1	P17174	P00533	Grn	P28799	P98160
Grpel1	Q9HAV7	P07902	Gsn	P06396	P32121
Gstp1	P09211	P22735	H3f3a	P84243	P49736
H4c1	P62805	P33992, P25205, P49736	H4c11	P62805	P33992, P25205, P49736
H4c12	P62805	P33992, P25205, P49736	H4c14	P62805	P33992, P25205, P49736

Input	UniProt Id	Interacts with	Input	UniProt Id	Interacts with
H4c2	P62805	P33992, P25205, P49736	H4c3	P62805	P33992, P25205, P49736
H4c4	P62805	P33992, P25205, P49736	H4c6	P62805	P33992, P25205, P49736
H4c8	P62805	P33992, P25205, P49736	H4c9	P62805	P33992, P25205, P49736
Hdgf	P51858	Q07955	Hmgb1	P63158	P13405
Hmox1	P09601	Q9NUX5	Hnrnpa1	P49312	Q3U0V1
Hnrnpa2b1	P22626	P09651	Hnrnpf	P52597	O43347
Hnrnpu	Q00839	Q9NZI8	Hp	P00738	P02647
Hprt1	P00492	Q96G04	Hsp90aa1	P07900	Q99558
Hsp90ab1	P08238	O14757	Hsp90b1	P14625	P11021
Hspa4	P34932	P11142	Hspa5	P11021	P13569
Hspa8	P11142	Q99558	Hspe1	P61604	P19320
Htra1	Q92743	P10636-8	Igfbp4	P22692	P05019
Iqgap1	B5DFH1	Q5M824	Itgb1	P05556	P78536
Itgb2	P05107	P00519	Itm2b	Q9Y287	P04578
Kpnb1	Q14974	Q99558	Krt1	P04264	P19320
Krt79	Q5XKE5	A1L190	Lamc1	P02468	P10493
Lasp1	Q14847-2	Q8TDS5	Ldha	P00338	P11142
Ldhb	P07195	P19320	Lgals1	P09382	P35968
Lgals3	P17931	P09237	Lgals3bp	Q08380	Q9BZR8
Lmna	P48678	P31750	Lox	P28300	Q9UBX5, P15502
Lrp1	Q07954	P02649	Lum	P51884	P50281
Mdh1	P40925	P00533	Mdh2	P40926	P19320
Mif	P14174	P14174	Mmp2	P08253	P16035
Msn	P26038	P16070	Mtpn	P62775	Q5XI32
Myh9	P35579	O00255	Myl12a	P19105	Q14164
Myl6	P60660-2	P46940	Naca	P38879	P15731
Ncl	P19338	Q00987	Nedd8	Q15843	Q8TBC4, P61081
Nid2	Q8IV28	P10745	Nme1	P15531	P10911
Npc2	P61916	16113, O15118	Npm1	P06748	Q8N726
Nucb1	Q02818	P10823	P4hb	P07237	P01137
Pdcd5	O14737	Q9NRD5	Pdcd6ip	Q8WUM4	P17931
Pdia3	P30101	P30304	Pdia6	Q15084	P11021
Pdlim1	O00151	P14136	Pea15	Q15121	P28482
Pebp1	P30086	P16050	Pfn1	P07737	O08816
Pgam1	P18669	P12004	Pgk1	P00558, P00558-1	O15379
Pkm	P14618-1	Q16665	Plod1	Q02809	P55795
Plod3	O60568	Q9UMD9	Pls3	Q9NRY6	P49639
Ppia	P62937	O00267	Ppib	P23284	P40855
Ppic	P45877	Q8N9N5	Prdx1	Q06830	P04156
Prdx2	P32119	P10599	Prdx5	P30044	P00441
Prdx6	P30041	P21796	Psap	Q8IWL2	Q9UGM3
Psma1	P25786	P21673	Psma2	P25787	Q9Y5K5
Psma3	P25788	Q12846	Psma4	P25789	Q16665
Psma5	P28066	Q9Y5K5	Psma6	P60900	Q9Y5K5
Psma7	O14818	Q16665	Psmb1	P20618	Q9Y5K5
Psmb2	P49721	Q9Y5K5	Psmb3	P49720	Q9Y5K5
Psmb4	P28070	Q9Y5K5	Psmb5	P28074	Q9Y5K5
Psmb6	P28072	Q9Y5K5	Psmb8	P28062-2	Q13114

Input	UniProt Id	Interacts with	Input	UniProt Id	Interacts with
Psme1	Q06323	Q9UL46	Ptk7	Q13308	P35222
Ptx3	P26022	P09038	Rad23b	P54727	P53350
Rbbp4	Q09028	P46531	Rcn1	Q15293	Q969Y2
Rcn3	Q96D15	Q9UMX1	Rdx	P35241	P10911
Rnh1	P13489	P03950	Rpl12	P30050	P27824
Rpl19	P84098	Q5S007	Rpl5	P47962	P23804
Rpl8	P62917	Q99558	Rplp1	P05386	Q9GZQ8
Rps12	P25398	P03372	Rps14	P62263	Q9Y3D8
Rps8	P62241	Q15843	Rpsa	P08865	P03372
Rsu1	Q15404	P48059	S100a11	P31949	P04271
S100a4	P26447	Q00987	S100a6	P06703	Q00987
Sdcbp	O00560	Q5VTY9	Serbp1	Q8NC51	P67809
Serpinc1	P01008	P01008	Serpine1	P05121	Q8NBQ5
Serpinf1	P36955	Q9P2X3	Serping1	E9PK97	Q9BRI3
Serpinh1	P50454	P03372	Sfpq	P23246	Q13882
Sod1	P00441	P29692	Sod2	P04179	P04179
Spp1	P10451	P16070	Sptbn1	Q01082	Q13813
Stip1	P31948	P07900	Tf	P02787	O00501
Thbs1	P07996-PRO_0000035842	P16671	Thbs2	Q03350	Q07954
Timp1	P01033	P14780	Timp2	P16035	P08253
Tkt	Q16832	P29353	Tln1	Q9Y490, P26039	P05556
Tmsb10	P63313	Q9NUX5	Tmsb4x	P20065	Q8WWQ8
Tpi1	P60174	P12004	Tpm4	P67936	Q9NRD5
Tpt1	P13693	P29692	Tubb4b	P68371	Q5S007
Tubb5	P04350	P22736	Txn	P10599	P19883
Txnrd1	Q16881	Q03135	Uba1	P22314	Q9UNE7
Ube2n	P61088	Q9UNE7	Vat1	P54219-3	Q9Y5Y5
Vcl	Q64727	Q8VI36	Vcp	P55072	O96017
Vim	P08670	P11021	Wdr1	O75083	P62993
Ybx1	P67809	Q99697	Ywhab	P35213	Q9JLT6
Ywhae	P62258	P30304	Ywhag	P61981	P30304, O14757
Ywhah	Q04917	P30304	Ywhaq	P27348	P30304
Ywhaz	P63104	P30304			

Input	ChEBI Id	Interacts with	Input	ChEBI Id	Interacts with
Actb	P60709	16851	Actg1	P63261	16851
Actn1	P12814	16851	Cfl1	P23528	16851
Dstn	P60981	16618	Flnb	O75369	16851
Flnc	Q14315	16851	Iqgap1	P46940	16618
Itgb1	P05556	16618	Kpnb1	Q14974	16851
Ldha	P00338	16851	Lrp1	Q07954	29108
Msn	P26038	16851	Myh9	P35579	16851
Myl6	P60660	16851	Niban2	Q96TA1	17283
Npc2	P61916	16113	Pkm	P14618-1	18319
Rdx	P35241	16851	Sptbn1	Q01082	16618
Tln1	Q9Y490	18348			

7. Identifiers not found

These 19 identifiers were not found neither mapped to any entity in Reactome.

Abracl	Aebp1	Anxa3	Cnn3	Cpq	Dkk3	Dpp3	Fkbp10
Fkbp2	Gm7324	Map4	Pdia4	Prl7c1	Ptms	Rexo2	Rnase4
Tagln	Twf1	Ufm1					

reactome

sWAT-MSC secretome from HFD mice
Pathway Analysis Report

Table of Contents

1. Introduction
2. Properties
3. Genome-wide overview
4. Most significant pathways
5. Pathways details
6. Identifiers found
7. Identifiers not found

1. Introduction

Reactome is a curated database of pathways and reactions in human biology. Reactions can be considered as pathway 'steps'. Reactome defines a 'reaction' as any event in biology that changes the state of a biological molecule. Binding, activation, translocation, degradation and classical biochemical events involving a catalyst are all reactions. Information in the database is authored by expert biologists, entered and maintained by Reactome's team of curators and editorial staff. Reactome content frequently cross-references other resources e.g. NCBI, Ensembl, UniProt, KEGG (Gene and Compound), ChEBI, PubMed and GO. Orthologous reactions inferred from annotation for Homo sapiens are available for 17 non-human species including mouse, rat, chicken, puffer fish, worm, fly, yeast, rice, and Arabidopsis. Pathways are represented by simple diagrams following an SBGN-like format.

Reactome's annotated data describe reactions possible if all annotated proteins and small molecules were present and active simultaneously in a cell. By overlaying an experimental dataset on these annotations, a user can perform a pathway over-representation analysis. By overlaying quantitative expression data or time series, a user can visualize the extent of change in affected pathways and its progression. A binomial test is used to calculate the probability shown for each result, and the p-values are corrected for the multiple testing (Benjamini–Hochberg procedure) that arises from evaluating the submitted list of identifiers against every pathway.

To learn more about our Pathway Analysis, please have a look at our relevant publications:

Fabregat A, Sidiropoulos K, Garapati P, Gillespie M, Hausmann K, Haw R, … D'Eustachio P (2016). The reactome pathway knowledgebase. Nucleic Acids Research, 44(D1), D481–D487. https://doi.org/10.1093/nar/gkv1351.

Fabregat A, Sidiropoulos K, Viteri G, Forner O, Marin-Garcia P, Arnau V, … Hermjakob H (2017). Reactome pathway analysis: a high-performance in-memory approach. BMC Bioinformatics, 18.

2. Properties

- This is an **overrepresentation** analysis: A statistical (hypergeometric distribution) test that determines whether certain Reactome pathways are over-represented (enriched) in the submitted data. It answers the question 'Does my list contain more proteins for pathway X than would be expected by chance?' This test produces a probability score, which is corrected for false discovery rate using the Benjamani-Hochberg method.
- 431 out of 474 identifiers in the sample were found in Reactome, where 9779 pathways were hit by at least one of them.
- IntAct interactors were included to increase the analysis background. This greatly increases the size of Reactome pathways, which maximises the chances of matching your submitted identifiers to the expanded pathway, but will include interactors that have not undergone manual curation by Reactome and may include interactors that have no biological significance, or unexplained relevance.
- This report is filtered to show only results for species 'Mus musculus' and resource 'all resources'.
- The unique ID for this analysis (token) is MjAyMDAzMjQxMzA0MDFfMzIwNA%3D%3D. This ID is valid for at least 7 days in Reactome's server. Use it to access Reactome services with your data.

3. Genome-wide overview

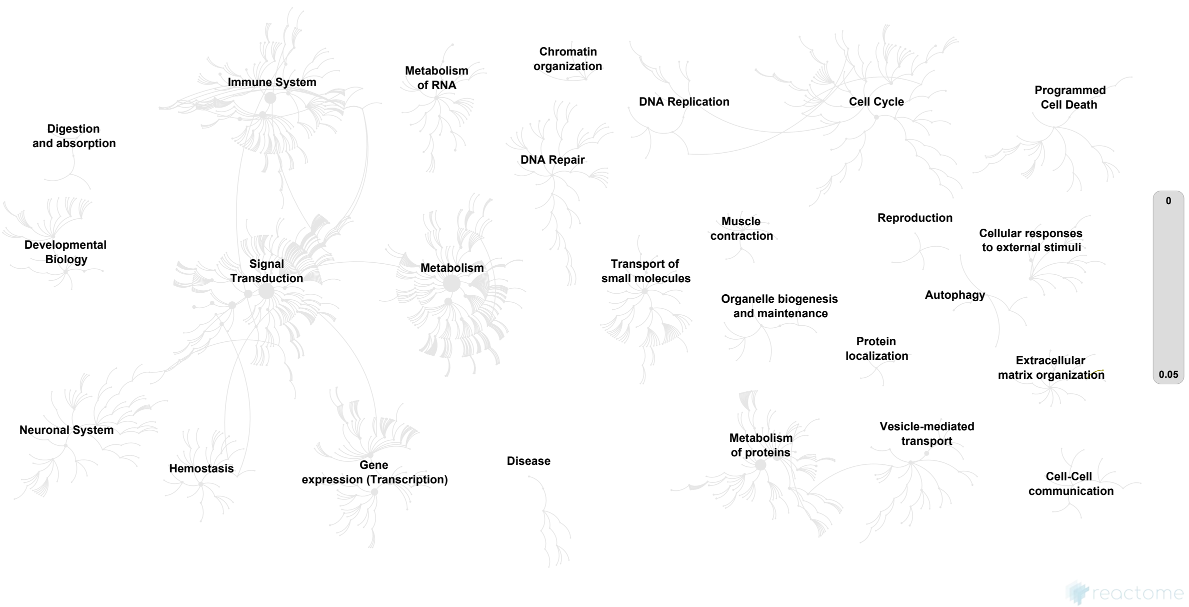

This figure shows a genome-wide overview of the results of your pathway analysis. Reactome pathways are arranged in a hierarchy. The center of each of the circular "bursts" is the root of one top-level pathway, for example "DNA Repair". Each step away from the center represents the next level lower in the pathway hierarchy. The color code denotes over-representation of that pathway in your input dataset. Light grey signifies pathways which are not significantly over-represented.

4. Most significant pathways

The following table shows the 25 most relevant pathways sorted by p-value.

Pathway name	Entities				Reactions	
	found	ratio	p-value	FDR*	found	ratio
Crosslinking of collagen fibrils	11 / 25	0.002	0.047	1	13 / 13	0.002
Antagonism of Activin by Follistatin	3 / 4	3.31e-04	0.076	1	2 / 2	2.56e-04
Laminin interactions	11 / 29	0.002	0.105	1	7 / 8	0.001
Chk1/Chk2(Cds1) mediated inactivation of Cyclin B:Cdk1 complex	6 / 14	0.001	0.132	1	1 / 4	5.13e-04
HSF1 activation	5 / 11	9.09e-04	0.136	1	5 / 6	7.69e-04
Regulation of Insulin-like Growth Factor (IGF) transport and uptake by Insulin-like Growth Factor Binding Proteins (IGFBPs)	35 / 120	0.01	0.169	1	6 / 6	7.69e-04
Cross-presentation of soluble exogenous antigens (endosomes)	16 / 51	0.004	0.191	1	4 / 4	5.13e-04
AUF1 (hnRNP D0) binds and destabilizes mRNA	20 / 64	0.005	0.229	1	3 / 4	5.13e-04
Dissolution of Fibrin Clot	6 / 17	0.001	0.24	1	17 / 18	0.002
Post-translational protein phosphorylation	32 / 114	0.009	0.24	1	1 / 1	1.28e-04
Regulation of ornithine decarboxylase (ODC)	15 / 50	0.004	0.249	1	1 / 4	5.13e-04
Assembly of collagen fibrils and other multimeric structures	20 / 69	0.006	0.253	1	20 / 26	0.003
Association of TriC/CCT with target proteins during biosynthesis	4 / 11	9.09e-04	0.284	1	1 / 1	1.28e-04
Elastic fibre formation	13 / 44	0.004	0.285	1	13 / 16	0.002
Regulation of RUNX2 expression and activity	15 / 52	0.004	0.296	1	1 / 5	6.41e-04
Ubiquitin-dependent degradation of Cyclin D	15 / 53	0.004	0.321	1	1 / 3	3.84e-04
p53-Independent G1/S DNA damage checkpoint	15 / 53	0.004	0.321	1	1 / 3	3.84e-04
p53-Independent DNA Damage Response	15 / 53	0.004	0.321	1	1 / 3	3.84e-04
Ubiquitin Mediated Degradation of Phosphorylated Cdc25A	15 / 53	0.004	0.321	1	1 / 3	3.84e-04
Autodegradation of the E3 ubiquitin ligase COP1	15 / 54	0.004	0.346	1	1 / 5	6.41e-04
Anchoring fibril formation	5 / 16	0.001	0.354	1	2 / 4	5.13e-04
NCAM1 interactions	7 / 24	0.002	0.373	1	1 / 3	3.84e-04

Pathway name	Entities				Reactions	
	found	ratio	p-value	FDR*	found	ratio
Transport of gamma-carboxylated protein precursors from the endoplasmic reticulum to the Golgi apparatus	3 / 9	7.44e-04	0.378	1	3 / 9	0.001
Removal of aminoterminal propeptides from gamma-carboxylated proteins	3 / 10	8.27e-04	0.442	1	3 / 9	0.001
Collagen formation	27 / 106	0.009	0.442	1	47 / 76	0.01

* False Discovery Rate

6. Regulation of Insulin-like Growth Factor (IGF) transport and uptake by Insulin-like Growth Factor Binding Proteins (IGFBPs) (R-MMU-381426)

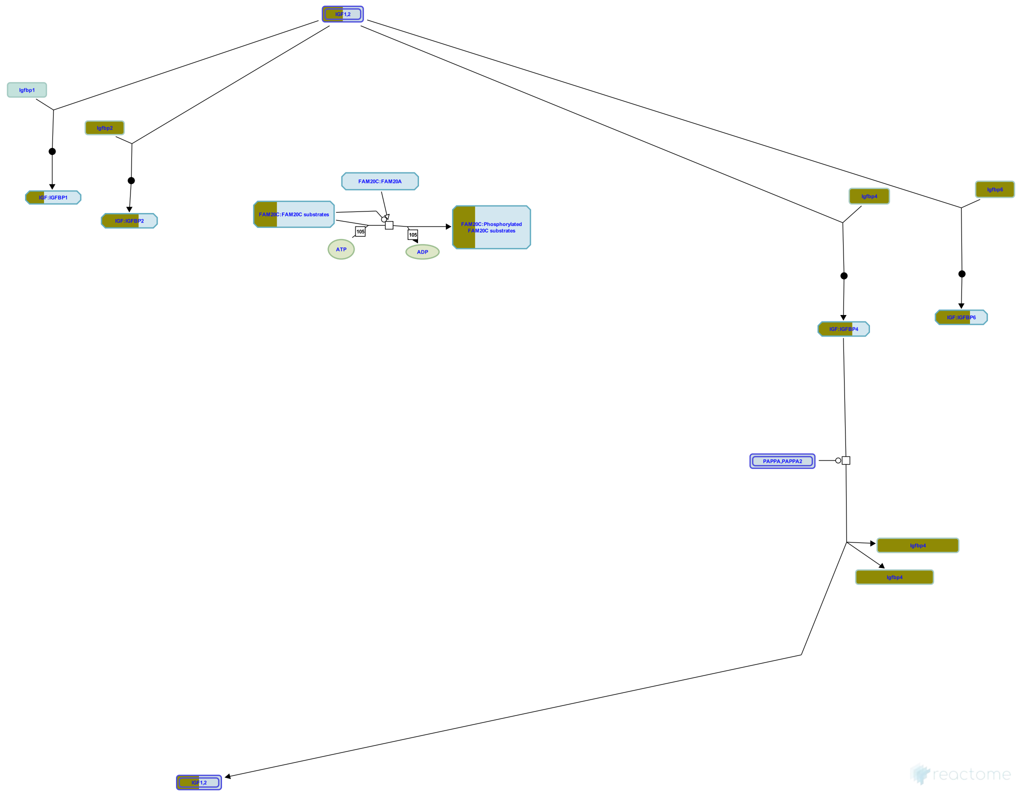

Cellular compartments: extracellular region.

Inferred from: Regulation of Insulin-like Growth Factor (IGF) transport and uptake by Insulin-like Growth Factor Binding Proteins (IGFBPs).

This event has been computationally inferred from an event that has been demonstrated in another species.

The inference is based on the homology mapping from PANTHER. Briefly, reactions for which all involved PhysicalEntities (in input, output and catalyst) have a mapped orthologue/paralogue (for complexes at least 75% of components must have a mapping) are inferred to the other species. High level events are also inferred for these events to allow for easier navigation.

More details and caveats of the event inference in Reactome. For details on PANTHER see also: http://www.pantherdb.org/about.jsp

References

Edit history

Date	Action	Author
2020-03-08	Created	Cook J

Entities found in this pathway (35)

Input	UniProt Id	Input	UniProt Id	Input	UniProt Id
Ahsg	P29699	Alb	P07724	C3	P01027

Input	UniProt Id	Input	UniProt Id	Input	UniProt Id
Calu	O35887	Ccn1	P18406	Cdh2	P15116
Csf1	P07141	Cst3	P21460	F5	O88783
Fbn1	Q61554	Fstl1	Q62356	Gas6	Q61592
Hsp90b1	P08113	Igf2	P09535	Igfbp2	P47877
Igfbp4	P47879	Igfbp6	P47880	Igfbp7	Q61581
Itih2	Q61703	Lamb1	P02469	Lamb2	Q61292
Lamc1	P02468	Lgals1	P16045	Mfge8	P21956
Nucb1	Q02819	P4hb	P09103	Pcsk9	Q80W65
Pdia6	Q922R8	Prkcsh	O08795	Qsox1	Q8BND5
Rcn1	Q05186	Serpinc1	P32261	Tf	Q921I1
Timp1	P12032	Vcan	Q62059		

6. Identifiers found

Below is a list of the input identifiers that have been found or mapped to an equivalent element in Reactome, classified by resource.

Entities (431)

Input	UniProt Id	Input	UniProt Id	Input	UniProt Id
A2m	P01023	Acta2	P62739	Actb	P60709
Actg1	P63259	Actn1	P12814	Actn4	O43707
Actr2	P61160	Actr3	F1P679	Adam12	O43184
Adam9	Q13443	Adamts1	Q9UHI8	Adamts5	Q9UNA0
Adss2	P30520	Ahsg	P02765	Aimp1	Q12904
Ak1	P00568	Ak2	P54819	Akr1a1	P14550
Akr1b1	P15121	Alb	P02768	Aldoa	P04075
Anxa1	P04083	Anxa2	Q07936	Anxa5	P08758
Anxa6	P08133	Apoh	P02749	Arhgdia	F1PL93
Arhgdib	P52566	Arpc1b	E2RMT4	Arpc2	F1PAG6
Arpc4	E2QWU0	Arpc5	O15511	Asah1	Q13510
Asl	P04424	Atic	P31939	Atox1	Q9TT99
Atp5f1b	J9NT37	Axl	F1PGV4	B2m	P61769
B4galt1	P15291	Bag3	Q5U2U8	Banf1	O75531
Bgn	P21810	Blvrb	P30043	Bmp1	P13497
C1ra	Q8CG16	C3	P01024	Calr	P27797
Calu	O43852	Cand1	Q86VP6	Canx	P35564
Cap1	Q01518	Capg	Q9BPX3	Capza1	A4FUA8
Capza2	Q5E997	Ccl2	P13500	Ccl7	Q03366
Ccl8	Q09141	Ccn1	O00622	Ccn2	P29279
Cct2	P78371	Cct3	P49368	Cct4	P50991
Cct5	P48643	Cdc37	Q16543	Cdc42	P60953
Cdh11	P55288	Cdh2	P19022	Cfl1	P23528
Ckb	P12277	Cltc	Q00610	Cmpk1	P30085
Cndp2	Q96KP4	Cnn2	Q99439	Col1a1	P02452
Col1a2	P08123	Col3a1	P02461	Col4a1	P02462
Col4a2	P08572	Col5a2	P05997	Col6a1	P12109
Col6a2	P12110	Col6a3	P12111	Colec12	Q5KU26
Cotl1	Q14019	Cox6b1	P14854	Cpe	P16870
Creg1	O75629	Crk	A0A287A2R9	Crlf1	F1ML91
Csf1	P09603	Csrp1	P21291	Cst3	P01034
Cst6	P04080	Cstb	P04080	Ctsa	P10619
Ctsb	P07858	Ctsh	P09668	Ctsl	P07711
Ctss	P25774	Ctsz	Q9UBR2	Cxcl5	P50228
Cycs	P99999	Dag1	Q14118	Dbi	P07108
Dcn	P07585	Ddah1	P56965	Dld	P09622
Dpysl2	P47942	Ecm1	Q16610	Eef1a1	P62630
Eef1b	P24534	Eef1g	P26641	Eef2	P13639
Efemp1	Q12805	Efemp2	O95967	Efhd2	F1SUW2
Egfr	A0A286ZV23	Ehd1	Q9WVK4	Eif2s1	P05198
Eif5a	P63241	Emilin1	Q9Y6C2	Emilin2	Q9BXX0

Input	UniProt Id	Input	UniProt Id	Input	UniProt Id
Eno1	P06733	Ero1a	E2RNW5	Erp44	Q9BS26
Ext1	Q16394	Ext2	Q93063	F2	J9NSF9
F3	P13726	F5	P12259	Fabp5	Q01469
Fah	P16930	Fam3c	Q92520	Fbln1	P23142
Fbln2	P98095	Fbln5	Q9UBX5	Fbn1	P35555
Fdps	Q920E5	Fgfr1	P11362	Fh	P07954
Fhl2	Q14192	Fkbp1a	P62942	Flna	P21333
Flnb	A0A0G2JXT8	Flnc	D3ZHA0	Fmod	Q06828
Fscn1	Q16658	Fst	P47931	Fstl1	Q12841
Fth1	P02794	Ftl1	P02793	G6pdx	Q00612
Galnt2	Q10471	Gapdh	P04406	Gars1	P41250
Gas6	Q14393	Gclm	P48507	Gdi1	P31150
Gdi2	P50395	Glb1	P16278	Glo1	J9NRV6
Gm20390	E9PZF0	Gm2a	P17900	Gmfb	F6WBZ2
Got1	P17174	Got2	P00505	Gpc1	P35052
Gpi	P06744	Grn	P28799	Grpel1	Q9HAV7
Gsn	P06396	Gsr	P00390-2	Gstm1	A4IFG0, Q9N0V4
Gstm2	F6Q751	Gsto1	P78417	Gstp1	P09211
H4c1	P62805	H4c11	P62805	H4c12	P62805
H4c14	P62805	H4c2	P62805	H4c3	P62805
H4c4	P62805	H4c6	P62805	H4c8	P62805
H4c9	P62805	H4f16	P62804	Hexa	P06865
Hexb	P07686	Hist1h2bq	G3V8B3	Hist1h4m	P62804
Hmox1	P09601	Hnrnpa1	F6XNZ3	Hnrnpa2b1	P22626
Hnrnpk	P61979	Hprt1	P00492	Hsp90aa1	P82995
Hsp90ab1	P34058	Hsp90b1	P14625	Hspa1a	P0DMV8
Hspa4	O88600	Hspa5	P11021	Hspa8	P11142
Hspb1	P14602	Hspd1	P63038	Hspg2	P98160
Htra1	Q92743	Hyou1	Q9Y4L1	Idh1	O75874
Igf2	P01344	Igfbp2	P47877	Igfbp4	P22692
Igfbp6	P47880	Igfbp7	Q16270	Inhba	Q04998
Inhbb	Q04999	Iqgap1	P46940	Itga5	P08648
Itgb1	P05556	Itih2	P19823	Itm2b	Q9Y287
Kars1	Q15046	Kpnb1	Q14974	Krt1	P04264
Krt10	E1C6Q9	Krt2	G3MZ71	Krt77	G3MYU2
Krt78	A6QNX5	Lama2	P24043	Lama4	Q16363
Lamb1	P07942	Lamb2	P11047, P55268	Lamc1	P11047
Lap3	Q01532	Lcp1	P13796	Ldha	P00340
Lgals1	P09382	Lgals3	P17931	Lgals3bp	Q08380
Lgmn	Q9R0J8	Lmna	P48678-3	Lox	P28300
Loxl1	Q08397	Loxl2	Q9Y4K0	Loxl3	P58215
Lrp1	E1BGJ0	Lta4h	P09960	Lum	P51884
Lyz2	P08905	Man2a1	Q16706	Man2b1	O00754
Mapk1	P28482	Mapre1	Q61166	Marcks	A0A287BRL8
Mdh1	P40925	Mdh2	P40926	Mfap5	Q13361
Mfge8	Q08431	Mif	P14174	Minpp1	Q9UNW1
Mmp12	P39900	Mmp2	P08253	Mmp3	P08254
Mrc2	Q9UBG0	Msn	Q9W002	Mt1	O19000
Mt2	P02798	Mtap	Q13126	Myh9	F1MQ37

Input	UniProt Id	Input	UniProt Id	Input	UniProt Id
Myl12a	Q6ZWQ9	Myl9	Q1LZF9	Naglu	P54802
Naxe	Q8NCW5	Ncl	P09405	Nedd8	Q71UE8
Neo1	F7B3P5	Nid1	P14543	Nid2	Q14112
Nme1	P15531	Npepps	P55786	Npm1	P13084
Nrp1	A4III3	Ntf3	P20783	Nucb1	Q02818
Ogn	P20774	Ostf1	Q92882	P4ha2	O15460
P4hb	P07237	Pabpc1	P29341	Pafah1b1	P43034
Pcolce	Q15113	Pcsk9	Q8NBP7	Pdap1	Q13442
Pdia3	P27773	Pdia6	Q15084	Pea15	Q15121
Pebp1	P30086	Pfn1	P07737	Pgam1	P18669
Pgd	P52209	Pgk1	P00558	Pgls	O95336
Pgm1	P36871	Pgm2	Q96G03	Pgp	A6NDG6
Pkm	P14618	Pla1a	Q53H76	Plau	P29598
Pld3	Q9VIF2	Plg	P00747	Plod1	Q02809
Plod2	O00469	Plod3	O60568	Ppia	P62937
Ppib	P23284	Ppid	F1RTY6	Ppp2r1a	P30153
Prdx2	Q61171	Prdx5	P99029	Prdx6	P30041
Prkcsh	P14314	Prnp	P04156	Pros1	P07225
Psma1	P25786	Psma2	P25787	Psma3	P25788
Psma4	P25789	Psma5	P28066	Psma6	P60900
Psma7	O14818	Psmb1	P20618	Psmb2	P49721
Psmb3	P49720	Psmb4	P28070	Psmb5	P28074
Psmb6	P28072	Psmb7	Q99436	Psmc2	P35998
Ptgr1	Q14914	Ptpa	P9WIA1	Ptx3	P26022
Pxdn	Q92626	Pygl	P06737	Qsox1	O00391
Rab7a	P51149	Rac1	P63000	Rack1	P63243
Rad23b	P54728	Ran	P62828	Rcn1	Q15293
Rdx	E9PT65	Rpl12	P30050	Rpl19	P84098
Rpl23a	P62750	Rpl24	P83731	Rpl30	P62888
Rpl4	P36578	Rpl5	P46777	Rpl7a	P62424
Rplp0	P05388	Rplp1	P05386	Rplp2	P05387
Rps28	P62857	Rps5	P46782	Rps8	P62241
Rpsa	P08865	Rsu1	D4A8F2	S100a10	P05943
S100a11	P31949	Sdcbp	O00560	Sema7a	F1NIZ9
Serpinb6a	Q6P9U0	Serpinc1	P01008	Serpine1	P05121
Serpine2	P07092	Serping1	P05155	Serpinh1	P50454
Slit3	F1RR92	Sod2	P09671	Sod3	O09164
Spon2	Q9BUD6	Sptbn1	Q62261	Stip1	P31948
Tagln2	P37802	Taldo1	P37837	Tars1	P26639
Tcn2	P20062	Tf	P02787	Thbs1	P07996
Thbs2	P35442	Thy1	P04216	Timp1	P01033
Timp2	P16035	Tkt	P29401	Tln1	Q9Y490
Tmpo	P42167-1	Tmsb4x	P62328	Tpi1	P60174
Tpm4	Q6IRU2	Tpp1	Q96AP0	Tubb4b	P68371
Tubb5	P04350	Tubb6	Q9BUF5	Txn	P08629
Txnrd1	Q16881	Uap1	Q16222	Uba1	Q02053
Ube2n	P61088	Ugp2	Q91ZJ5	Usp14	Q5U2N2
Vapa	Q9P0L0	Vat1	Q99536	Vcam1	A7MBB0
Vcan	P13611	Vcl	P18206	Vcp	P55072

Input	UniProt Id	Input	UniProt Id	Input	UniProt Id
Vegfa	P15692	Vegfd	O43915	Vim	P02543
Wdr1	O75083	Xdh	F1S3Y7	Xpnpep1	Q54G06
Ybx1	P62960	Ywhab	F1SDR7	Ywhae	P62260
Ywhag	F2Z4Z1	Ywhah	F2Z4Y1	Ywhaq	F1SA98
Ywhaz	F2Z558	Zyx	Q9N675		

Input	Ensembl Id	Input	Ensembl Id	Input	Ensembl Id
Acta2	ENSG00000107796	Actb	ENST00000331789	Anxa1	ENSG00000135046
Anxa2	ENSG00000182718	Atp5f1b	ENSG00000110955	B2m	ENSG00000166710
Calr	ENSG00000179218	Capza1	ENSG00000116489	Ccl2	ENSG00000108691
Ccn1	ENSG00000145386	Ccn2	ENSG00000118523	Cdc42	ENSG00000070831
Cfl1	ENSG00000172757	Cnn2	ENSG00000064666	Col1a1	ENSG00000108821
Col1a2	ENSG00000164692	Csf1	ENSG00000184371	Csrp1	ENSG00000159176
Cycs	ENSG00000172115	Egfr	ENSG00000146648	F3	ENSG00000117525
Fdps	ENSG00000160752	Fhl2	ENSG00000115641	Fscn1	ENSG00000075618
Fth1	ENST00000273550	Gsto1	ENSG00000148834	Hmox1	ENSG00000100292
Hnrnpa2b1	ENSG00000122566	Hsp90aa1	ENSG00000080824	Hsp90b1	ENSG00000166598
Hspa1a	ENSG00000204389, ENSG00000215328, ENSG00000234475, ENSG00000235941, ENSG00000237724	Hspa5	ENSG00000044574	Hspa8	ENSG00000109971
Hspb1	ENSG00000106211	Hspd1	ENSG00000144381	Hyou1	ENSG00000149428
Igf2	ENST00000337883, ENST00000381406	Igfbp7	ENSG00000163453	Itga5	ENSG00000161638
Itgb1	ENSG00000150093	Lcp1	ENSG00000136167	Lgals3	ENSG00000131981
Lmna	ENSG00000160789	Mif	ENSG00000240972	Mmp2	ENSG00000087245
Mmp3	ENSG00000149968	Msn	ENSG00000147065	Mt2	ENSG00000125148
Mtap	ENSG00000099810	Myl9	ENSG00000101335	Pdia6	ENSG00000143870
Ppia	ENSG00000196262	Rplp0	ENSG00000089157	Serpine1	ENSG00000106366
Serpinh1	ENSG00000149257	Sod2	ENSG00000112096	Taldo1	ENSG00000177156
Tf	ENSG00000117525	Thbs1	ENSG00000137801	Timp1	ENSG00000102265
Tln1	ENSG00000137076	Tpp1	ENSG00000166340	Txnrd1	ENSG00000198431
Vapa	ENST00000340541	Vcam1	ENSG00000162692	Vegfa	ENSG00000112715
Vim	ENSG00000026025				

Interactors (336)

Input	UniProt Id	Interacts with	Input	UniProt Id	Interacts with
Actb	P60709	P13569	Actg1	P63261	P23528
Actn1	P12814	P12931	Actn4	O43707	Q07157
Actr2	A7MB62	Q95107	Actr3	Q99JY9	P34152
Adam12	O43184-2	O95633	Adam9	Q13443	Q99962
Ahnak	Q09666	P00533	Ahsg	P12763	P17931
Aimp1	Q12904-2	Q96CV9	Akap12	Q02952	P00533
Alb	P02768	P02768	Aldoa	P05064	P63101
Anxa1	P04083	Q13546	Anxa2	EBI-8785901, P07355	Q8NBP7
Anxa6	P14824	P63101	Apoh	P02749	P08519
Arhgdia	P52565	P60953	Arpc1b	O15143	O00401
Arpc4	P59998	O00401	Arpc5	O15511	P00533
Atic	P31939	P00533	Atox1	O00244	P35670
Atp5f1b	P06576	P05919	Axl	P30530	P19174

Input	UniProt Id	Interacts with	Input	UniProt Id	Interacts with
B2m	P61769	P61769	B4galt1	P08037	P29752
Bag3	O95817	Q00613	Banf1	O75531	O75531
Basp1	Q91XV3	Q62108	Bmp1	P13497-2	P07585
Bmper	Q9W2H2	P18824	C3	P01024-PRO_0000005911, P01024-PRO_0000005908, P01024	P08603, P05156
Calr	P27797	P30101	Calu	O43852	P04049
Cand1	Q86VP6	P62877, Q13616	Canx	P27824	P13569
Cap1	Q13114	Q99558	Capg	P40121	Q8IUQ4
Capza1	P47753, P13127	Q9Y5K6	Capza2	P47755	P52907
Ccl2	P13500	P13501	Ccn1	P20248	Q9H211
Ccn2	P29279	P02751	Ccn3	P48745	O43559
Ccn4	O95388	P49639	Ccn5	O76076	P49639
Cct2	P78371	Q8IWZ6	Cct3	P49368	Q13882
Cct4	P50991	Q13882	Cct5	P48643	P17987
Cdc37	Q16543	Q99558	Cdc42	P60953	Q13177
Cdh2	P15116	P18031	Cfl1	P23528	P53667
Cfl2	Q549N0	P23528	Cltc	Q00610	P11142
Col1a1	P02452	P02751	Col3a1	P02461-1	P09486
Col4a1	P08120	O46339	Colec12	Q5KU26	Q9NV70
Coro1c	Q9ULV4	P19320	Cotl1	Q14019	P09917
Crk	P46108	P16234	Csf1	P07141	P05480
Csrp1	P21291	Q16790	Cst6	Q15828	O95070
Ctsa	P10619	P08670	Ctsb	P07858	P02760
Cuta	O60888	P22736	Cycs	P99999	Q9Y4K3
Dag1	Q14118	Q14118	Dcn	P07585	P02751
Dld	O08749	Q9CQV8	Dnpep	Q19087	O17761
Dstn	P60981	P60709	Ecm1	Q16610	Q9NQ94
Eef1a1	P68104	Q9NYA1	Eef1b	P24534	P26641
Eef1g	P26641, P26641-2	P29692, P24534	Eef2	P58252	P63101
Efemp1	Q12805	P24592	Efemp2	O95967	Q9UBX5, P15502
Egfr	P00533	P11021	Ehd1	Q9H4M9	16618, Q9H1K0
Eif2s1	P05198	Q9Z2B5	Eif5a	P63242	P63101
Emilin1	Q9Y6C2-2, Q9Y6C2	Q00994	Eno1	P06733	P03496
Ero1a	Q96HE7	P07237	Erp29	P30040	Q969S0
F3	P13726	P08709	Fah	P16930	P00533
Fam3c	Q92520	Q15223	Fbln1	P23142	P02647
Fbln2	P98095	Q9H2F3	Fbln5	Q9UBX5	P15502
Fbn1	P35555	P15502	Fdps	P14324	Q9BRI3
Fgfr1	P11362	P23352	Fh	P07954	P21673
Fhl1	Q13642	P04156	Fhl2	O35115	Q91V26
Fkbp1a	P62942	P36896	Flna	P21333	O14786
Flnb	O75369	Q13233	Flnc	Q14315	O75923
Fmod	P13605	P08603	Fscn1	Q16658	P54278
Fst	P19883	P10599	Fstl1	Q12841	P08571
Fth1	P02794	P04792	Galnt2	Q10471	Q9HC36
Gapdh	P04406	P00558	Gdi1	P31150	P51149
Gdi2	P50395	Q8BMD2	Glb1	P16278	P08670
Got1	P17174	P00533	Grn	P28799	P98160

Input	UniProt Id	Interacts with	Input	UniProt Id	Interacts with
Grpel1	Q9HAV7	P07902	Gsn	P06396	P32121
Gsto1	P78417	P04792	Gstp1	P09211	P22735
H4c1	P62805	P33992, P25205, P49736	H4c11	P62805	P33992, P25205, P49736
H4c12	P62805	P33992, P25205, P49736	H4c14	P62805	P33992, P25205, P49736
H4c2	P62805	P33992, P25205, P49736	H4c3	P62805	P33992, P25205, P49736
H4c4	P62805	P33992, P25205, P49736	H4c6	P62805	P33992, P25205, P49736
H4c8	P62805	P33992, P25205, P49736	H4c9	P62805	P33992, P25205, P49736
Hexb	P07686	P06865	Hmox1	P09601	Q9NUX5
Hnrnpa1	P09651	P22626	Hnrnpa2b1	P22626	P67809
Hnrnpk	P61978	P12931	Hprt1	P00492	Q96G04
Hsp90aa1	P07900	Q15185	Hsp90ab1	P08238	O14757
Hsp90b1	P14625	P11021	Hspa1a	P0DMV8	P13569
Hspa4	P34932	P11142	Hspa5	P11021	P13569
Hspa8	P11142	Q99558	Hspb1	P04792	P31749
Hspd1	P10809	Q16822	Hspe1	P61604	P19320
Hspg2	P98160-PRO_0000391621, P98160-PRO_0000391622, P98160	P35968	Htra1	Q92743	P10636-8
Hyou1	Q9Y4L1	P11021	Igfbp4	P22692	P05019
Igfbp6	P24592	P24592	Inhba	P08476	P21674
Iqgap1	P46940	P60709	Itga5	P08648	P02751
Itgb1	P05556	P78536	Itm2b	Q9Y287	P04578
Kpnb1	Q14974	Q99558	Krt1	P04264	P19320
Krt10	P13645	P19320	Lama4	Q16363	P04637
Lamc1	P02468	P10493	Lap3	P28838	Q13185
Lasp1	Q14847-2	Q8TDS5	Ldha	P00338	P11142
Lgals1	P09382	P35968	Lgals3	P16110	Q9Z0P7
Lgals3bp	Q08380	Q9BZR8	Lmna	P02545	Q14739
Lox	P28300	Q9UBX5, P15502	Lrp1	Q07954	P02649
Lum	P51884	P50281	Mapk1	P63086	Q63155
Mapre1	Q15691	P11021	Mdh1	P40925	P00533
Mdh2	P40926	P19320	Mif	P14174	P14174
Mmp2	P08253	P16035	Mrc2	Q9UBG0	P02454
Msn	P26038	P16070	Mtap	Q9V813	Q7KNM2
Mtpn	P62775	Q5XI32	Myh9	P35579	O00255
Myl12a	P19105	Q14164	Myl9	P24844-2	Q99757
Naca	P38879	P15731	Nbl1	Q61477	P12023
Ncl	P19338	Q00987	Nedd8	Q15843	P68104
Nid2	Q8IV28	P10745	Nme1	P15531	P10911
Npm1	P06748	Q8N726	Nrp1	P97333	P08648
Ntf3	P20783	P07174	Nucb1	Q02818	P10823
Ostf1	Q92882	P42858	P4ha2	O15460	P07237
P4hb	P07237	Q6PJG9	Pabpc1	P11940	Q92900
Pafah1b1	P43034	P07900	Pcolce	Q15113	P18828
Pcsk9	Q8NBP7	P08253	Pdcd5	O14737	Q9NRD5

Input	UniProt Id	Interacts with	Input	UniProt Id	Interacts with
Pdia3	P30101	P30304	Pdia6	Q15084	P11021
Pdlim1	O00151	P14136	Pea15	Q15121	P28482
Pebp1	P30086	P16050	Pfn1	P07737	P60709
Pgam1	P18669	P12004	Pgk1	P00558, P00558-1	O15379
Pgm2	Q96G03	P04792	Pkm	P14618-1	Q16665
Pla1a	EBI-3954042	P78527	Pld3	Q8IV08	P19838
Plod1	Q02809	P55795	Plod3	O60568	Q9UMD9
Ppia	P62937	O00267	Ppib	P23284	P40855
Ppic	P45877	Q8N9N5	Ppid	Q08752	P63244
Ppp2r1a	P30153	P13569	Prdx2	P32119	P10599
Prdx5	P30044	P00441	Prdx6	P30041	Q7KZN9
Prkcsh	P14314	P04578	Prnp	P10279, P04156	P04156
Psma1	P25786	P21673	Psma2	P25787	Q9Y5K5
Psma3	P25788	Q12846	Psma4	P25789	Q16665
Psma5	P28066	Q9Y5K5	Psma6	P60900	Q9Y5K5
Psma7	O14818	Q16665	Psmb1	P20618	Q9HAP6
Psmb2	P49721	Q9Y5K5	Psmb3	P49720	Q9Y5K5
Psmb4	P28070	Q9Y5K5	Psmb5	P28074	Q9Y5K5
Psmb6	P28072	Q9Y5K5	Psmb7	Q99436	Q9Y5K5
Psmc2	P35998	P38936	Ptk7	Q13308	P35222
Ptpa	P18433	P12931	Ptx3	P26022	P09038
Rac1	P63000	Q13177	Rack1	P63244	Q14694
Rad23b	P54727	P53350	Ran	P62826	P47813
Rcn1	Q15293	Q969Y2	Rcn3	Q96D15	Q9UMX1
Rdx	P35241	P10911	Rnh1	P13489	P03950
Rpl12	P30050	P27824	Rpl19	P84098	Q5S007
Rpl23a	P62750	Q5S007	Rpl24	P83731	Q99558
Rpl30	P62888	Q5S007	Rpl4	P36578	Q99558
Rpl5	P47962	P23804	Rpl7a	P62424	Q99558
Rplp0	P05388	P03372	Rplp1	P05386	Q9GZQ8
Rps8	P62241	Q15843	Rpsa	P08865	P03372
Rsu1	Q15404	P48059	S100a10	P60903	P07355
S100a11	P31949	P04271	S100a4	P26447	Q00987
S100a6	P06703	Q02790	Sdcbp	O00560	Q5VTY9
Sdf4	Q9BRK5	Q13418	Sema7a	O75326	O60486
Septin11	Q9NVA2	Q16181	Serbp1	Q8NC51	P67809
Serpinc1	P01008	P01008	Serpine1	P05121	Q8NBQ5
Serpinf1	P36955	Q9P2X3	Serping1	E9PK97	Q9BRI3
Serpinh1	P50454	P03372	Sh3bgrl	O75368	P00533
Sod2	P04179	P04179	Sptbn1	Q01082	Q13813
Stip1	P31948	P53041	Tars1	P26639	P19320
Tf	P02787	O00501	Tgm2	P21980	P02751
Thbs1	P07996-PRO_0000035842	P16671	Thbs2	Q03350	Q07954
Timp1	P01033	P14780	Timp2	P16035	P08253
Tkt	Q16832	P29353	Tln1	Q9Y490, P26039	P05556
Tmpo	P42167	P27824	Tmsb10	P63313	Q9NUX5
Tmsb4x	P20065	Q8WWQ8	Tpi1	P60174	P12004
Tpm4	P67936	Q9NRD5	Tpp1	Q96AP0	Q92900
Tpt1	P13693	P29692	Tubb4b	P68371	Q5S007

Input	UniProt Id	Interacts with	Input	UniProt Id	Interacts with
Tubb5	P04350	P22736	Tubb6	Q922F4	Q62108
Txn	P10599	P19883	Txnrd1	Q16881	Q03135
Uba1	P22314	Q9UNE7	Ube2n	P61088	P98170
Ugp2	Q16851	O76003	Usp14	P54578	Q9Y5K5
Vapa	Q9WV55	Q9QXM1	Vasn	Q6EMK4	P22735
Vat1	P54219-3	Q9Y5Y5	Vcam1	P19320	Q14160
Vcan	P13611	P14780	Vcl	Q64727	Q8VI36
Vcp	P55072	O96017	Vegfa	EBI-2895491	Q16665
Vegfd	O43915	Q15836	Vim	P08670	P11021
Wdr1	O75083	P62993	Xpnpep1	Q9NQW7-3	P69892
Ybx1	P67809	Q9NZI8, Q9Y6M1	Ywhab	P35213	Q9JLT6
Ywhae	P62258	P30304	Ywhag	P61981	P30304, O14757
Ywhah	Q04917	P30304	Ywhaq	P27348	P30304
Ywhaz	P63101	P63017	Zyx	Q15942	P02751

Input	ChEBI Id	Interacts with	Input	ChEBI Id	Interacts with
Actb	P60709	18348	Actg1	P63261	18348
Actn1	P12814	18348	Actn4	O43707	16851
Cand1	Q86VP6	18348	Capza1	P52907	18348
Cdc42	P60953	15996	Cfl1	P23528	18348
Cltc	Q00610	16618	Dstn	P60981	16618
Ehd1	Q9H4M9	16618	Flna	P21333	18348
Flnb	O75369	18348	Flnc	Q14315	18348
Iqgap1	P46940	16618	Itgb1	P05556	16618
Kpnb1	Q14974	18348	Ldha	P00338	18348
Lrp1	Q07954	29108	Msn	P26038	18348
Myh9	P35579	18348	Neo1	Q92859	28424
Niban2	Q96TA1	17283	Pkm	P14618-1	18319
Rab7a	P51149	18348	Rac1	P63000	15996
Rack1	P63244	18348	Ran	P62826	17552, 15996
Rdx	P35241	16851	S100a13	P97352	29036
Sptbn1	Q01082	16618	Tln1	Q9Y490	18348

7. Identifiers not found

These 43 identifiers were not found neither mapped to any entity in Reactome.

Aebp1	Angptl2	Anxa3	Ccdc80	Cd248	Clic4	Cpq	Cpxm1
Crip1	Ctla2a	Dkk3	Dpp3	Fkbp10	Fkbp2	GaskbB	Glipr2
Hars1	Lpp	Man1a	Map4	Metrnl	Mroh6	Oaf	Olfml3
Palld	Pcdhgc3	Pdia4	Postn	Prl2c3	Prl7c1	Ptms	Rars1
Rexo2	Rnase4	Srpx2	Svep1	Tagln	Tgfbr3	Twf1	Txndc12
Txnl1	Uap1l1	Ufm1					

reactome

BM-MSC secretome from HFD mice
Pathway Analysis Report

Table of Contents

1. Introduction
2. Properties
3. Genome-wide overview
4. Most significant pathways
5. Pathways details
6. Identifiers found
7. Identifiers not found

1. Introduction

Reactome is a curated database of pathways and reactions in human biology. Reactions can be considered as pathway 'steps'. Reactome defines a 'reaction' as any event in biology that changes the state of a biological molecule. Binding, activation, translocation, degradation and classical biochemical events involving a catalyst are all reactions. Information in the database is authored by expert biologists, entered and maintained by Reactome’s team of curators and editorial staff. Reactome content frequently cross-references other resources e.g. NCBI, Ensembl, UniProt, KEGG (Gene and Compound), ChEBI, PubMed and GO. Orthologous reactions inferred from annotation for Homo sapiens are available for 17 non-human species including mouse, rat, chicken, puffer fish, worm, fly, yeast, rice, and Arabidopsis. Pathways are represented by simple diagrams following an SBGN-like format.

Reactome's annotated data describe reactions possible if all annotated proteins and small molecules were present and active simultaneously in a cell. By overlaying an experimental dataset on these annotations, a user can perform a pathway over-representation analysis. By overlaying quantitative expression data or time series, a user can visualize the extent of change in affected pathways and its progression. A binomial test is used to calculate the probability shown for each result, and the p-values are corrected for the multiple testing (Benjamini–Hochberg procedure) that arises from evaluating the submitted list of identifiers against every pathway.

To learn more about our Pathway Analysis, please have a look at our relevant publications:

Fabregat A, Sidiropoulos K, Garapati P, Gillespie M, Hausmann K, Haw R, … D’Eustachio P (2016). The reactome pathway knowledgebase. Nucleic Acids Research, 44(D1), D481–D487. https://doi.org/10.1093/nar/gkv1351.

Fabregat A, Sidiropoulos K, Viteri G, Forner O, Marin-Garcia P, Arnau V, … Hermjakob H (2017). Reactome pathway analysis: a high-performance in-memory approach. BMC Bioinformatics, 18.

2. Properties

- This is an **overrepresentation** analysis: A statistical (hypergeometric distribution) test that determines whether certain Reactome pathways are over-represented (enriched) in the submitted data. It answers the question ‘Does my list contain more proteins for pathway X than would be expected by chance?’ This test produces a probability score, which is corrected for false discovery rate using the Benjamani-Hochberg method.
- 380 out of 413 identifiers in the sample were found in Reactome, where 8320 pathways were hit by at least one of them.
- IntAct interactors were included to increase the analysis background. This greatly increases the size of Reactome pathways, which maximises the chances of matching your submitted identifiers to the expanded pathway, but will include interactors that have not undergone manual curation by Reactome and may include interactors that have no biological significance, or unexplained relevance.
- This report is filtered to show only results for species 'Mus musculus' and resource 'all resources'.
- The unique ID for this analysis (token) is MjAyMDAzMjQxMzA3NDhfMzIwNw%3D%3D. This ID is valid for at least 7 days in Reactome’s server. Use it to access Reactome services with your data.

3. Genome-wide overview

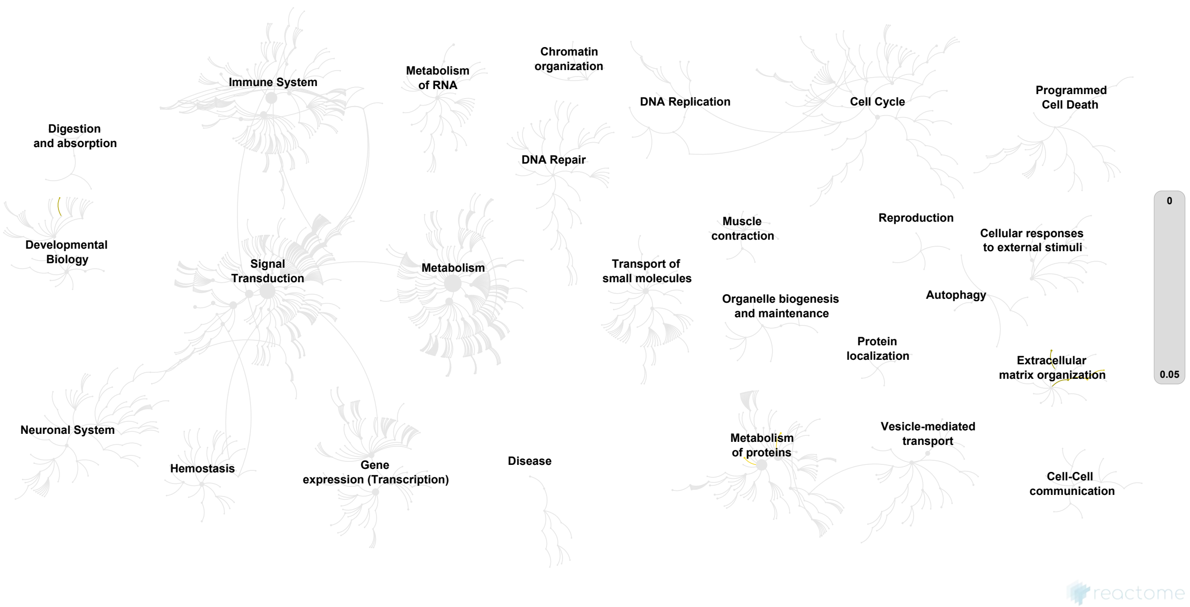

This figure shows a genome-wide overview of the results of your pathway analysis. Reactome pathways are arranged in a hierarchy. The center of each of the circular "bursts" is the root of one top-level pathway, for example "DNA Repair". Each step away from the center represents the next level lower in the pathway hierarchy. The color code denotes over-representation of that pathway in your input dataset. Light grey signifies pathways which are not significantly over-represented.

4. Most significant pathways

The following table shows the 25 most relevant pathways sorted by p-value.

Pathway name	Entities				Reactions	
	found	ratio	p-value	FDR*	found	ratio
Regulation of Insulin-like Growth Factor (IGF) transport and uptake by Insulin-like Growth Factor Binding Proteins (IGFBPs)	41 / 120	0.01	0.002	1	5 / 6	7.69e-04
Post-translational protein phosphorylation	39 / 114	0.009	0.002	1	1 / 1	1.28e-04
Assembly of collagen fibrils and other multimeric structures	25 / 69	0.006	0.006	1	23 / 26	0.003
Crosslinking of collagen fibrils	11 / 25	0.002	0.017	1	13 / 13	0.002
NCAM1 interactions	10 / 24	0.002	0.031	1	1 / 3	3.84e-04
Collagen degradation	20 / 62	0.005	0.039	1	15 / 20	0.003
Collagen formation	31 / 106	0.009	0.04	1	55 / 76	0.01
Antagonism of Activin by Follistatin	3 / 4	3.31e-04	0.052	1	2 / 2	2.56e-04
Activation of C3 and C5	4 / 7	5.79e-04	0.06	1	3 / 3	3.84e-04
Collagen chain trimerization	14 / 43	0.004	0.07	1	10 / 27	0.003
Chk1/Chk2(Cds1) mediated inactivation of Cyclin B:Cdk1 complex	6 / 14	0.001	0.075	1	1 / 4	5.13e-04
Collagen biosynthesis and modifying enzymes	22 / 76	0.006	0.079	1	32 / 50	0.006
HSF1 activation	5 / 11	9.09e-04	0.082	1	5 / 6	7.69e-04
Laminin interactions	10 / 29	0.002	0.085	1	6 / 8	0.001
ECM proteoglycans	18 / 57	0.005	0.092	1	8 / 15	0.002
Dissolution of Fibrin Clot	6 / 17	0.001	0.146	1	17 / 18	0.002
Alternative complement activation	3 / 7	5.79e-04	0.179	1	9 / 9	0.001
RUNX1 regulates transcription of genes involved in differentiation of keratinocytes	2 / 4	3.31e-04	0.202	1	2 / 2	2.56e-04
Trafficking and processing of endosomal TLR	6 / 19	0.002	0.206	1	4 / 6	7.69e-04
Elastic fibre formation	12 / 44	0.004	0.21	1	11 / 16	0.002
Degradation of the extracellular matrix	38 / 150	0.012	0.212	1	58 / 78	0.01
Anchoring fibril formation	5 / 16	0.001	0.241	1	2 / 4	5.13e-04
Non-integrin membrane-ECM interactions	12 / 42	0.003	0.261	1	5 / 9	0.001

Pathway name	Entities				Reactions	
	found	ratio	p-value	FDR*	found	ratio
Transport of gamma-carboxylated protein precursors from the endoplasmic reticulum to the Golgi apparatus	3 / 9	7.44e-04	0.288	1	3 / 9	0.001
Platelet degranulation	30 / 131	0.011	0.319	1	5 / 8	0.001

* False Discovery Rate

5. Pathways details

For every pathway of the most significant pathways, we present its diagram, as well as a short summary, its bibliography and the list of inputs found in it.

1. Regulation of Insulin-like Growth Factor (IGF) transport and uptake by Insulin-like Growth Factor Binding Proteins (IGFBPs) (R-MMU-381426)

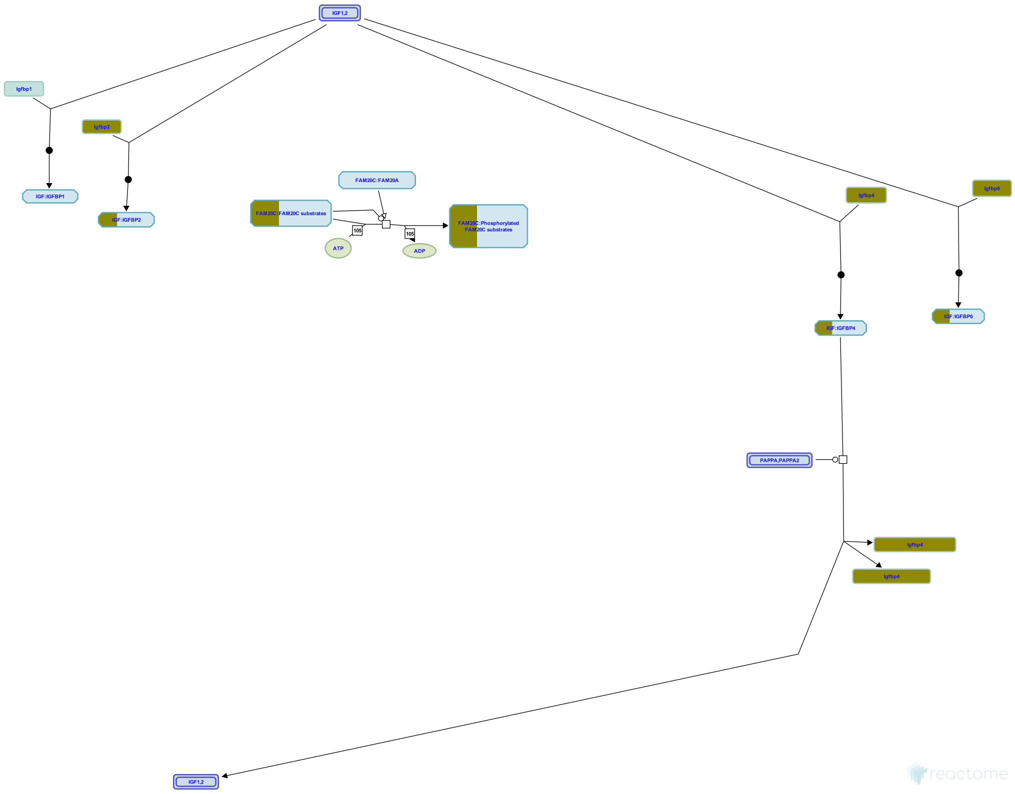

Cellular compartments: extracellular region.

Inferred from: Regulation of Insulin-like Growth Factor (IGF) transport and uptake by Insulin-like Growth Factor Binding Proteins (IGFBPs).

This event has been computationally inferred from an event that has been demonstrated in another species.

The inference is based on the homology mapping from PANTHER. Briefly, reactions for which all involved PhysicalEntities (in input, output and catalyst) have a mapped orthologue/paralogue (for complexes at least 75% of components must have a mapping) are inferred to the other species. High level events are also inferred for these events to allow for easier navigation.

More details and caveats of the event inference in Reactome. For details on PANTHER see also: http://www.pantherdb.org/about.jsp

References

Edit history

Date	Action	Author
2020-03-08	Created	Cook J

Entities found in this pathway (41)

Input	UniProt Id	Input	UniProt Id	Input	UniProt Id
Adam10	O35598	Ahsg	P29699	Alb	P07724
Aplp2	Q06335	Apoe	P08226	C3	P01027
C4b	P01029	Calu	O35887	Ccn1	P18406
Cdh2	P15116	Csf1	P07141	Cst3	P21460
F5	O88783	Fbn1	Q61554	Fn1	P11276
Fstl1	Q62356	Fstl3	Q9EQC7	Gas6	Q61592
Hsp90b1	P08113	Igfbp2	P47877	Igfbp3	P47878
Igfbp4	P47879	Igfbp6	P47880	Igfbp7	Q61581
Itih2	Q61703	Lamb1	P02469	Lamb2	Q61292
Lamc1	P02468	Lgals1	P16045	Mfge8	P21956
Nucb1	Q02819	P4hb	P09103	Pcsk9	Q80W65
Pdia6	Q922R8	Prkcsh	O08795	Prss23	Q9D6X6
Rcn1	Q05186	Serpinc1	P32261	Spp1	P10923
Tf	Q921I1	Timp1	P12032		

25. Platelet degranulation (R-MMU-114608)

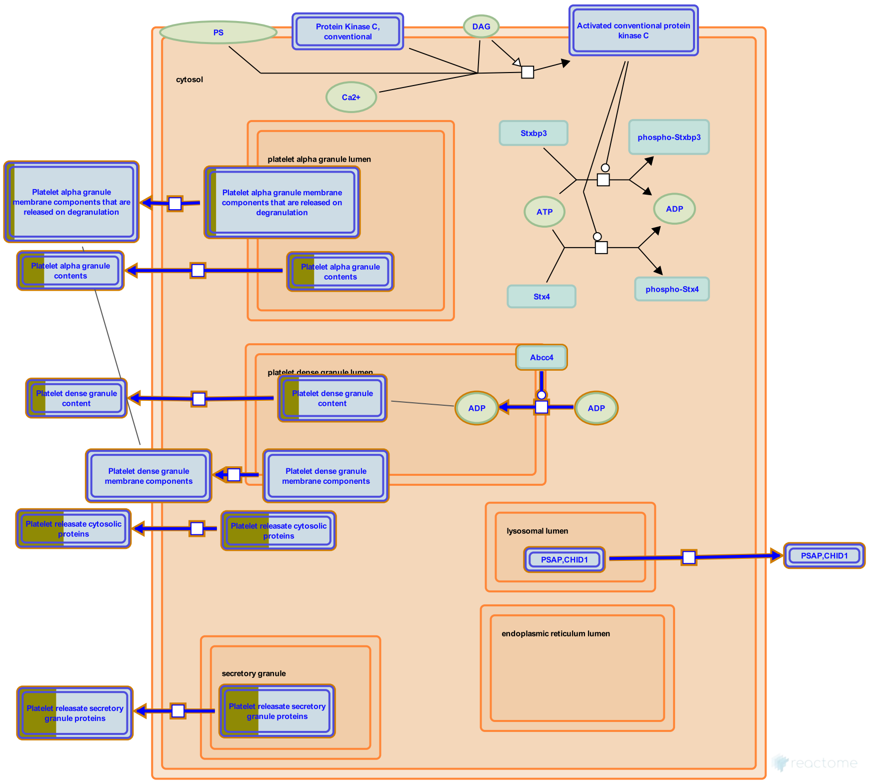

Inferred from: Platelet degranulation .

This event has been computationally inferred from an event that has been demonstrated in another species.

The inference is based on the homology mapping from PANTHER. Briefly, reactions for which all involved PhysicalEntities (in input, output and catalyst) have a mapped orthologue/paralogue (for complexes at least 75% of components must have a mapping) are inferred to the other species. High level events are also inferred for these events to allow for easier navigation.

More details and caveats of the event inference in Reactome. For details on PANTHER see also: http://www.pantherdb.org/about.jsp

References

Edit history

Date	Action	Author
2020-03-08	Created	Cook J

Entities found in this pathway (30)

Input	UniProt Id	Input	UniProt Id	Input	UniProt Id
A2m	Q6GQT1	Actg1	P63260	Ahsg	P29699
Alb	P07724	Aldoa	P05064	Anxa5	P48036
Aplp2	Q06335	Apoh	Q01339	Ecm1	Q61508
F5	O88783	Fam3c	Q91VU0	Fn1	P11276
Gas6	Q61592	Islr	Q6GU68	Lgals3bp	Q07797

Input	UniProt Id	Input	UniProt Id	Input	UniProt Id
Manf	Q9CXI5	Pf4	Q9Z126	Plg	P20918
Pros1	Q08761	Serpine1	P22777	Serping1	P97290
Tagln2	Q9WVA4	Tf	Q921I1	Thbs1	P35441
Timp1	P12032	Tln1	P26039	Tmsb4x	P20065
Vcl	Q64727	Vegfd	P97946	Wdr1	O88342

6. Identifiers found

Below is a list of the input identifiers that have been found or mapped to an equivalent element in Reactome, classified by resource.

Entities (380)

Input	UniProt Id	Input	UniProt Id	Input	UniProt Id
A2m	P01023	Acta2	P62737	Actb	P60709
Actg1	P63261	Actr2	P61160	Actr3	F1P679
Adam10	O14672	Adam9	Q13443	Adamts1	Q9UHI8
Adamts5	Q9UNA0	Adm	F7AC43	Adss2	P30520
Ahsg	P02765	Akr1a1	P14550	Akr1b1	P15121
Alb	P02768	Aldoa	P04075	Anxa1	P07150
Anxa2	P07355	Anxa5	P08758	Anxa6	P08133
Aplp2	Q06481	Apoe	P02649	Apoh	P02749
Arg1	P05089	Arhgdia	F1PL93	Arhgdib	P52566
Arpc1b	E2RMT4	Arpc2	F1PAG6	Arpc3	E2R985
Asah1	Q13510	Atox1	Q9TT99	Atp5f1b	J9NT37
Atp6ap1	Q15904	Atp6ap2	O75787	Axl	F1PGV4
B2m	P61769	B4galt1	P15291	Bag3	Q5U2U8
Bgn	P21810	Blvrb	P30043	Bmp1	P13497
C1qa	E1BSP0	C1qb	F1NH19	C1qc	Q02105
C1ra	Q8CG16	C3	P01024	C4b	P01029
Calr	P27797	Calu	O43852	Cant1	Q8WVQ1
Cap1	Q01518	Capg	Q9BPX3	Ccl2	P13500
Ccl6	Q68FP3	Ccl7	Q03366	Ccl8	Q09141
Ccl9	Q5FVN3	Ccn1	O00622	Ccn2	P29279
Cd14	P08571	Cdh2	P19022	Cfh	P06909
Cfl1	P23528	Cfp	P27918	Ckb	P12277
Cmpk1	P30085	Cndp2	Q96KP4	Cnn2	Q99439
Col12a1	Q99715	Col14a1	Q05707	Col1a1	P02452
Col1a2	P08123	Col2a1	P02458	Col3a1	P02461
Col4a1	P02462	Col4a2	P08572	Col5a1	P20908
Col5a2	P05997	Col5a3	P25940	Col6a1	P12109
Col6a2	P12110	Col6a3	P12111	Colec12	Q5KU26
Comp	P49747	Cotl1	Q14019	Cpe	P16870
Creg1	O75629	Crlf1	R4GH98	Csf1	P09603
Csf1r	A7Z067	Csrp1	P21291	Cst3	P01034
Cstb	P04080	Ctsa	P10619	Ctsb	P07858
Ctsc	P53634	Ctsd	P07339	Ctsh	P09668
Ctsk	P43235	Ctsl	P07711	Ctss	P25774
Ctsz	Q9UBR2	Cxcl1	P09341	Cxcl16	Q8BSU2
Cxcl5	P50228	Dag1	Q14118	Dbi	P07108
Dcn	P07585	Dld	P09622	Dpysl2	P47942
Ecm1	Q16610	Eef1a1	P68104	Eef1b	O70251
Eef1g	Q9D8N0	Eef2	P13639	Efemp1	Q12805
Ehd1	Q641Z6	Emilin1	Q9Y6C2	Emilin2	Q9BXX0
Eno1	P06733	Ero1a	E2RNW5	Erp44	Q9BS26

Input	UniProt Id	Input	UniProt Id	Input	UniProt Id
Esd	P10768	Ext2	Q93063	Ezr	P15311
F2	P00734	F5	P12259	Fabp5	Q01469
Fam3c	Q92520	Fbln1	P23142	Fbln2	P98095
Fbln5	Q9UBX5	Fbn1	P35555	Fkbp1a	P62942
Flnb	A0A0G2JXT8	Flnc	E2RP26	Fmod	Q06828
Fn1	P02751	Fscn1	Q16658	Fst	P47931
Fstl1	Q12841	Fstl3	O95633	Fth1	P02794
Ftl1	P02793	Galnt2	Q10471	Gapdh	P04406
Gas6	Q14393	Gdi2	P50395	Gm20390	E9PZF0
Gm20547	B8JJN0	Gm2a	P17900	Got1	P17174
Got2	P00505	Gpc1	P35052	Gpi	P06744
Grn	P28799	Gsn	P06396	Gsr	P00390-2
Gstp1	P09211	Gusb	P08236	Gzme	P08884
H4c1	P62805	H4c11	P62805	H4c12	P62805
H4c14	P62805	H4c2	P62805	H4c3	P62805
H4c4	P62805	H4c6	P62805	H4c8	P62805
H4c9	P62805	H4f16	P62804	Hexb	P07686
Hist1h4m	P62804	Hmox1	P09601	Hnrnpa1	F6XNZ3
Hnrnpa2b1	P22626	Hnrnpu	Q6IMY8	Hp	P00738
Hprt1	P00492	Hsp90aa1	P07900	Hsp90ab1	P08238
Hsp90b1	P14625	Hspa4	O88600	Hspa5	P11021
Hspa8	P11142	Hspd1	P63038	Hspg2	P98160
Htra1	Q92743	Icam1	P13597	Idh1	O75874
Igfbp2	P47877	Igfbp3	P17936	Igfbp4	P22692
Igfbp6	P47880	Igfbp7	Q16270	Inhba	Q04998
Iqgap1	P46940	Islr	O14498	Itgb2	P05107
Itih2	P19823	Itm2b	Q9Y287	Kpnb1	Q14974
Krt1	P04264	Krt2	P35908	Krt5	F1SGG6
Krt76	Q01546	Krt77	Q7Z794	Krt78	Q8N1N4
Krt79	Q5XKE5	Lama2	P24043	Lama4	Q16363
Lamb1	P07942	Lamb2	P11047, P55268	Lamc1	P11047
Lcp1	P13796	Ldha	P00340	Ldhb	P00337
Lgals1	P09382	Lgals3	P17931	Lgals3bp	Q08380
Lgmn	Q9R0J8	Lipa	Q9Z0M5	Lmna	P48678-3
Lox	P28300	Loxl1	Q08397	Loxl2	Q9Y4K0
Loxl3	P58215	Lpl	P11151	Lrp1	E1BGJ0
Lta4h	P09960	Ltbp2	Q14767	Lum	P51884
Lyz2	P08905	Man2a1	Q16706	Man2b1	O00754
Manf	P55145	Marcks	A0A287BRL8	Mdh1	P40925
Mdh2	P40926	Mfap5	Q13361	Mfge8	Q08431
Mif	P14174	Minpp1	Q9UNW1	Mmp12	P39900
Mmp19	Q99542	Mmp2	P08253	Mmp3	P08254
Mrc1	P22897	Mrc2	Q9UBG0	Msn	Q9W002
Mt2	P02798	Mtap	Q13126	Mydgf	Q969H8
Myh9	F1MQ37	Myl12a	Q6ZWQ9	Naglu	P54802
Ncl	P09405	Nedd8	Q71UE8	Nid1	P14543
Nid2	Q14112	Nme1	P15531	Npc2	P61916
Npm1	P13084	Nrp1	A4III3	Nucb1	Q02818
Ogn	P20774	P4hb	P07237	Pafah1b2	P68402

Input	UniProt Id	Input	UniProt Id	Input	UniProt Id
Pcolce	Q15113	Pcsk9	Q8NBP7	Pdcd6ip	Q8WUM4
Pdia3	F6WRR4	Pdia6	Q15084	Pebp1	P30086
Pf4	P02776	Pfn1	P07737	Pgam1	P18669
Pgd	P52209	Pgk1	P00558	Pgls	O95336
Pgm1	P36871	Pkm	P14618	Pla2g7	Q5RHM0
Plau	P00749	Pld3	Q9VIF2	Plg	P00747
Plod1	Q02809	Plod2	O00469	Plod3	O60568
Ppia	P62937	Ppib	P23284	Prdx2	Q61171
Prdx6	P30041	Prkcsh	P14314	Prnp	P04156
Pros1	P07225	Prss23	O95084	Psma1	P25786
Psma2	P25787	Psma3	P25788	Psma4	P25789
Psma5	P28066	Psma6	P60900	Psma7	O14818
Psmb2	P49721	Psmb3	P49720	Psmb6	P28072
Ptx3	P26022	Pvr	Q5U334	Pxdn	Q92626
Qpct	Q16769	Rcn1	Q15293	Rdx	P35241
Rnaset2b	C0HKG6	Rpl12	P30050	Rpl4	P36578
Rplp1	P05386	Rplp2	P05387	Rps12	P25398
Rps14	P62263	Rps28	P62857	Rps8	P62241
Rsu1	E2R9R1	S100a11	P31949	Saa3	Q2HXZ9
Sat2	Q96QD8	Sdcbp	O00560	Sema7a	F1NIZ9
Serpinb2	P29524	Serpinb6a	Q6P9U0	Serpinc1	P01008
Serpine1	P05121	Serpine2	P07092	Serping1	P05155
Serpinh1	P50454	Slit3	F1RR92	Sod2	P09671
Sod3	O09164	Spon2	Q9BUD6	Spp1	P10451
Sptbn1	Q62261	Ssc5d	A1L4H1	Stip1	P31948
Tagln2	P37802	Taldo1	P37837	Tcn2	P20062
Tf	P02787	Tgfbi	Q15582	Thbs1	P07996
Thbs2	P35442	Timp1	P01033	Timp2	P16035
Tkt	P29401	Tln1	Q9Y490	Tmsb4x	P62328
Tpi1	P60174	Tpm4	Q6IRU2	Tpp1	Q96AP0
Tubb5	P07437	Txn	P08629	Txnrd1	Q16881
Ube2l3	F6W7B6	Ube2n	P61088	Vat1	Q99536
Vcam1	P29533	Vcl	P18206	Vcp	P55072
Vegfd	O43915	Vim	P02543	Wdr1	O75083
Xdh	F1S3Y7	Ybx1	P62960	Ywhab	Q5XGC8
Ywhae	P62260	Ywhag	Q0V9W8	Ywhah	Q28DR3
Ywhaq	Q5BL40	Ywhaz	Q6P4Z5		

Input	Ensembl Id	Input	Ensembl Id	Input	Ensembl Id
Acta2	ENSG00000107796	Actb	ENST00000331789	Anxa1	ENSG00000135046
Anxa2	ENSG00000182718	Apoe	ENSG00000130203	Atp5f1b	ENSG00000110955
B2m	ENSG00000166710	C1qc	ENSGALP00000007599	Calr	ENSG00000179218
Ccl2	ENSG00000108691	Ccn1	ENSG00000145386	Ccn2	ENSG00000118523
Cfl1	ENSG00000172757	Cnn2	ENSG00000064666	Col1a1	ENSG00000108821
Col1a2	ENSG00000164692	Csf1	ENSG00000184371	Csf1r	ENSG00000182578
Csrp1	ENSG00000159176	Ctsd	ENSG00000117984	Cxcl1	ENSG00000163739
Fn1	ENSG00000115414	Fscn1	ENSG00000075618	Fth1	ENST00000273550
Hmox1	ENSG00000100292	Hnrnpa2b1	ENSG00000122566	Hsp90aa1	ENSG00000080824
Hsp90b1	ENSG00000166598	Hspa5	ENSG00000044574	Hspa8	ENSG00000109971

Input	UniProt Id	Interacts with
Ecm1	Q16610	Q9NQ94
Eef1b	P24534	P26641
Eef2	P13639	Q16539
Ehd1	Q9H4M9	Q9H1K0
Eno1	P06733	P03496
Erp29	P30040	Q969S0
Fam3c	Q92520	Q15223
Fbln2	P98095	Q9H2F3
Fbn1	P35555	P15502
Flnb	O75369	Q13233
Fmod	P13605	P08603
Fscn1	Q16658	P54278
Fstl1	Q12841	P08571
Galnt2	Q10471	Q9HC36
Gdi2	P50395	Q8BMD2
Grn	P28799	P98160
Gstp1	P09211	P22735
H4c11	P62805	P33992, P25205, P49736
H4c14	P62805	P33992, P25205, P49736
H4c3	P62805	P33992, P25205, P49736
H4c6	P62805	P33992, P25205, P49736
H4c9	P62805	P33992, P25205, P49736
Hmox1	P09601	Q9NUX5
Hnrnpa2b1	P22626	P67809
Hnrnpu	Q00839	Q9NZI8
Hprt1	P00492	Q96G04
Hsp90ab1	P08238	O14757
Hspa4	P34932	P11142
Hspa8	P11142	Q99558
Hspe1	P61604	P19320
Htra1	Q92743	P10636-8
Igfbp3	P17936	Q86XT9
Igfbp6	P24592	P24592
Iqgap1	B5DFH1	Q5M824
Itm2b	Q9Y287	P04578
Krt1	P04264	P19320
Krt79	Q5XKE5	A1L190
Lamc1	P02468	P10493
Ldhb	P07195	P19320
Lgals3	P17931	P09237
Lmna	P48678	P31750
Lrp1	Q07954	P02649
Mdh1	P40925	P00533
Mif	P14174	P14174
Mrc1	P25588	P32485

Input	UniProt Id	Interacts with
Eef1a1	P68105	Q06124
Eef1g	P26641, P26641-2	P29692, P24534
Efemp1	Q12805	P24592
Emilin1	Q9Y6C2-2, Q9Y6C2	Q00994
Ero1a	Q96HE7	P07237
Ezr	P15311, P31977	Q63155
Fbln1	P23142	P02647
Fbln5	Q9UBX5	P15502
Fkbp1a	P62942	P36896
Flnc	Q14315	O75923
Fn1	P02751	P07585
Fst	P19883	P10599
Fth1	P02794	P04792
Gapdh	P04406	P00558
Got1	P17174	P00533
Gsn	P06396	P32121, P49407
H4c1	P62805	P33992, P25205, P49736
H4c12	P62805	P33992, P25205, P49736
H4c2	P62805	P33992, P25205, P49736
H4c4	P62805	P33992, P25205, P49736
H4c8	P62805	P33992, P25205, P49736
Hexb	P07686	P06865
Hnrnpa1	P09651	P22626
Hnrnpab	Q7ZYE9	P26599
Hp	P00738	P02647
Hsp90aa1	P07900	P12931
Hsp90b1	P14625	P11021
Hspa5	P11021	P13569
Hspd1	P10809	Q16822
Hspg2	P98160-PRO_0000391621, P98160-PRO_0000391622, P98160	P35968
Icam1	EBI-6726290, EBI-10052614	Q04206
Igfbp4	P22692	P05019
Inhba	P08476	P21674
Itgb2	P05107	P00519
Kpnb1	Q14974	Q9H1C4
Krt76	Q01546	O43559
Lama4	Q16363	P04637
Ldha	P00338	P11142
Lgals1	P09382	P35968
Lgals3bp	Q08380	Q9BZR8
Lox	P28300	Q9UBX5, P15502
Lum	P51884	P50281
Mdh2	P40926	P19320
Mmp2	P08253	P16035
Mrc2	Q9UBG0	P02454

Input	UniProt Id	Interacts with	Input	UniProt Id	Interacts with
Msn	P26038	P16070	Mtap	Q9V813	Q7KNM2
Myh9	P35579	O00255	Myl12a	P19105	Q14164
Naca	P38879	P15731	Nap1l1	P55209	P52333
Ncl	P19338	P35579	Nedd8	Q15843	Q8TBC4, P61081
Nid2	Q8IV28	P10745	Nme1	P15531	P10911
Npc2	P61916	16113, O15118	Npm1	P06748	Q8N726
Nrp1	P97333	P08648	Nucb1	Q02818	P10823
P4hb	P07237	P01137	Pafah1b2	P68402	P43034
Pcolce	Q15113	P18828	Pcsk9	Q8NBP7	P08253
Pdcd6ip	Q8WUM4	P12931	Pdia3	P30101	P30304
Pdia6	Q15084	P11021	Pdlim1	O00151	P14136
Pebp1	P30086	P16050	Pf4	P02776-PRO_0000351217, P02776	P78556, P13501, O15444, Q99616, P48061
Pfn1	P07737	O08816	Pgam1	P18669	P12004
Pgk1	P00558, P00558-1	O15379	Pkm	P14618-1	Q16665
Pld3	Q8IV08	P19838	Plod1	Q02809	P55795
Plod3	O60568	Q9UMD9	Pls3	Q9NRY6	P49639
Ppia	P62937	O00267	Ppib	P23284	P40855
Ppic	P45877	Q8N9N5	Prdx2	P32119	P10599
Prdx6	P30041	Q7KZN9	Prkcsh	P14314	P04578
Prnp	P10279, P04156	P04156	Psma1	P25786	P21673
Psma2	P25787	Q9Y5K5	Psma3	P25788	Q12846
Psma4	P25789	Q16665	Psma5	P28066	Q9Y5K5
Psma6	P60900	Q9Y5K5	Psma7	O14818	Q16665
Psmb2	P49721	Q9Y5K5	Psmb3	P49720	Q9Y5K5
Psmb6	P28072	Q9Y5K5	Ptx3	P26022	P09038
Pvr	P15151	P11686	Rcn1	Q15293	Q969Y2
Rcn3	Q96D15	Q9UMX1	Rdx	P35241	P10911
Rnh1	P13489	P03950	Rpl12	P30050	P27824
Rpl4	P36578	Q99558	Rplp1	P05386	Q9GZQ8
Rps12	P25398	P03372	Rps14	P62263	Q9Y3D8
Rps8	P62241	Q15843	Rsu1	Q15404	Q13418
S100a11	P31949	P04271	S100a4	P26447	P35579
S100a6	P06703	Q02790	Sat2	Q96F10	P21673
Sdcbp	O00560	P24592	Sdf4	Q9BRK5	Q13418
Sema7a	O75326	O60486	Serbp1	Q8NC51	P67809
Serpinc1	P01008	P01008	Serpine1	P05121	Q8NBQ5
Serpinf1	P36955	Q9P2X3	Serping1	E9PK97	Q9BRI3
Serpinh1	P50454	P03372	Sod2	P04179	P04179
Spp1	P10451	P16070	Sptbn1	Q01082	Q13813
Ssc5d	A1L4H1	Q13526	Stip1	P31948	P19320
Tf	P02787	O00501	Thbs1	P07996-PRO_0000035842	P16671
Thbs2	Q03350	Q07954	Timp1	P01033	P14780
Timp2	P16035	P08253	Tinagl1	Q9GZM7	O95429
Tkt	Q16832	P29353	Tln1	Q9Y490	P19320, 18348
Tmsb10	P63313	Q9NUX5	Tmsb4x	P20065	Q8WWQ8
Tpi1	P60174	P12004	Tpm4	P67936	Q9NRD5
Tpp1	Q96AP0	O43768	Tpt1	P13693	P29692
Tubb5	P04350	P22736	Txn	P10599	P19883

Input	UniProt Id	Interacts with
Txnrd1	Q16881	Q03135
Ube2n	P61088	P98170
Vat1	P54219-3	Q9Y5Y5
Vcl	Q64727, P12003	P26039
Vegfd	O43915	Q15836
Wdr1	O75083	P62993
Ywhab	P35213	Q9JLT6
Ywhag	P61981	Q92934
Ywhaq	P27348	Q92934

Input	UniProt Id	Interacts with
Ube2l3	P68036	P50876
Vasn	Q6EMK4	P22735
Vcam1	P19320	P06733
Vcp	P55072	O96017
Vim	P08670	P11021
Ybx1	P67809	Q99697
Ywhae	P62258	Q92934
Ywhah	Q04917	Q92934
Ywhaz	P63104	Q92934

Input	ChEBI Id	Interacts with
Actb	P60709	18348
Cfh	P08603	18132
Dstn	P60981	16618
Ezr	P15311	18348
Flnc	Q14315	18348
Iqgap1	P46940	16618
Ldha	P00338	18348
Msn	P26038	18348
Niban2	Q96TA1	17283
Pkm	P14618-1	18319
Sptbn1	Q01082	16618

Input	ChEBI Id	Interacts with
Actg1	P63261	18348
Cfl1	P23528	18348
Ehd1	Q9H4M9	16618
Flnb	O75369	18348
Igfbp3	P17936	16336
Kpnb1	Q14974	18348
Lrp1	Q07954	29108
Myh9	P35579	18348
Npc2	P61916	16113
Rdx	P35241	16851
Tln1	Q9Y490	18348

7. Identifiers not found

These 33 identifiers were not found neither mapped to any entity in Reactome.

Adck5	Aebp1	Angptl2	Anxa3	C1s1	Ccdc80	Cd248	Cpxm1
Ctla2a	Dkk3	Fkbp10	Fndc1	GaskbB	Hars1	Hint1	Man1a
Metrnl	Mroh6	Oaf	Olfml2b	Pdgfrl	Pdia4	Prl7c1	Ptms
Rars1	Rnase4	Rps18-ps6	Sbsn	Srpx	Srpx2	Svep1	Tagln
Tgfbr3							
