# Supplementary material for: A comparative study on normal and obese mice indicates that the secretome of mesenchymal stromal cells is influenced by tissue environment and physiopathological conditions
Source: Cell Commun Signal. 2020 Jul 29;18:118. doi: 10.1186/s12964-020-00614-w (PMC7388533; doi:10.1186/s12964-020-00614-w)
Supplement: Supplementary file 4 — Additional file 3. GO analysis carried out with PANTHER. The list shows ontology terms overrepresented in the secretomes of vWAT-MSCs, sWAT-MSCs, and BM-MSCs taken from ND- and HFD-treated mice. Ontology terms were classified as: cellular components, protein classes, molecular functions, biological processes, and pathways. [file 12964_2020_614_MOESM4_ESM.pdf]

Analysis Summary: Please report in publication

vWAT-MSC secretome from ND mice

Analysis Type: PANTHER Overrepresentation Test (Released 20190711)

Annotation Version and Release Date: PANTHER version 15 Released 2020-02-14

Analyzed List: Client Text Box Input (Mus musculus) Change

Reference List: Mus musculus (all genes in database) Change

Annotation Data Set: PANTHER GO-Slim Cellular Component

Test Type: (•) Fisher's Exact ( ) Binomial

Correction: (•) Calculate False Discovery Rate ( ) Use the Bonferroni correction for multiple testing ( ) No correction

Results

	Reference list	Client Text Box Input
Uniquely Mapped IDS:	22265 out of 22265	361 out of 363
Unmapped IDs:	0	0
Multiple mapping information:	0	5

Export Table XML with user input ids JSON with user input ids View: -- Please select a chart to display --

Displaying only results for FDR P < 0.05, click here to display all results

	Mus musculus (REF)	Client Text Box Input (Hierarchy NEW!)					
PANTHER GO-Slim Cellular Component	#	#	expected	Fold Enrichment	+/-	raw P value	FDR
proteasome core complex, alpha-subunit complex	8	7	.13	53.67	+	1.49E-09	7.03E-08
↳proteasome complex	39	13	.64	20.45	+	1.36E-12	1.18E-10
↳endopeptidase complex	39	13	.64	20.45	+	1.36E-12	1.41E-10
↳peptidase complex	55	13	.90	14.50	+	5.33E-11	3.08E-09
↳intracellular part	6691	150	109.09	1.38	+	6.41E-06	1.33E-04
↳intracellular	6721	151	109.58	1.38	+	5.01E-06	1.08E-04
Arp2/3 protein complex	9	6	.15	40.89	+	7.25E-08	2.09E-06
↳cytoskeletal part	486	19	7.92	2.40	+	8.27E-04	1.02E-02
↳cytoskeleton	586	21	9.55	2.20	+	1.41E-03	1.70E-02
↳intracellular non-membrane-bounded organelle	1344	42	21.91	1.92	+	8.78E-05	1.47E-03
↳non-membrane-bounded organelle	1344	42	21.91	1.92	+	8.78E-05	1.43E-03
↳actin cytoskeleton	160	17	2.61	6.52	+	4.32E-09	1.61E-07
collagen trimer	17	4	.28	14.43	+	3.14E-04	4.54E-03
cytosolic small ribosomal subunit	40	7	.65	10.73	+	9.34E-06	1.87E-04
↳cytosolic part	162	20	2.64	7.57	+	1.58E-11	1.02E-09
↳cytoplasmic part	2902	86	47.31	1.82	+	4.63E-08	1.42E-06

↳cytoplasm	4183	138	68.20	2.02	+	2.34E-17	6.09E-15
↳cytosol	753	52	12.28	4.24	+	9.56E-18	4.97E-15
↳cytosolic ribosome	104	17	1.70	10.03	+	1.03E-11	7.67E-10
↳ribosome	151	17	2.46	6.91	+	1.94E-09	8.41E-08
↳ribonucleoprotein complex	463	21	7.55	2.78	+	4.10E-05	7.35E-04
↳small ribosomal subunit	57	7	.93	7.53	+	7.31E-05	1.27E-03
↳ribosomal subunit	143	17	2.33	7.29	+	9.11E-10	4.74E-08
collagen-containing extracellular matrix	66	11	1.08	10.22	+	4.04E-08	1.31E-06
↳extracellular matrix	177	18	2.89	6.24	+	2.85E-09	1.14E-07
↳extracellular region part	1253	59	20.43	2.89	+	6.00E-13	7.80E-11
↳extracellular region	1253	59	20.43	2.89	+	6.00E-13	1.04E-10
cytosolic large ribosomal subunit	64	10	1.04	9.58	+	2.88E-07	7.13E-06
↳large ribosomal subunit	86	10	1.40	7.13	+	3.31E-06	7.82E-05
actin filament	70	9	1.14	7.89	+	4.88E-06	1.10E-04
↳polymeric cytoskeletal fiber	246	11	4.01	2.74	+	3.06E-03	3.62E-02
↳supramolecular fiber	298	15	4.86	3.09	+	1.77E-04	2.80E-03
↳supramolecular polymer	301	15	4.91	3.06	+	1.97E-04	3.01E-03
↳supramolecular complex	301	15	4.91	3.06	+	1.97E-04	2.92E-03
extracellular space	1169	49	19.06	2.57	+	4.59E-09	1.59E-07
Unclassified	12713	156	207.27	.75	-	1.03E-07	2.68E-06
integral component of plasma membrane	770	2	12.55	.16	-	6.31E-04	8.20E-03
↳integral component of membrane	1114	5	18.16	.28	-	5.29E-04	7.05E-03
↳intrinsic component of membrane	1141	5	18.60	.27	-	3.84E-04	5.39E-03
↳membrane part	2301	14	37.51	.37	-	1.02E-05	1.97E-04
↳membrane	3585	35	58.45	.60	-	5.11E-04	7.00E-03
↳intrinsic component of plasma membrane	777	2	12.67	.16	-	6.51E-04	8.26E-03
↳plasma membrane part	1622	8	26.44	.30	-	3.46E-05	6.42E-04

© Copyright 2020 Paul Thomas All Rights Reserved.

Analysis Summary: Please report in publication

sWAT-MSC secretome from ND mice

Analysis Type: PANTHER Overrepresentation Test (Released 20190711)

Annotation Version and Release Date: PANTHER version 15 Released 2020-02-14

Analyzed List: Client Text Box Input (Mus musculus) Change

Reference List: Mus musculus (all genes in database) Change

Annotation Data Set: PANTHER GO-Slim Cellular Component

Test Type: (•) Fisher's Exact ( ) Binomial

Correction: Calculate False Discovery Rate Use the Bonferroni correction for multiple testing No correction

Results

	Reference list	Client Text Box Input
Uniquely Mapped IDS:	22265 out of 22265	572 out of 569
Unmapped IDs:	0	1
Multiple mapping information:	0	15

Export Table XML with user input ids JSON with user input ids View: -- Please select a chart to display --

Displaying only results for FDR P < 0.05, click here to display all results

	Mus musculus (REF)	Client Text Box Input (Hierarchy NEW!)					
PANTHER GO-Slim Cellular Component	#	#	expected	Fold Enrichment	+/-	raw P value	FDR
proteasome core complex, alpha-subunit complex	8	7	.20	34.24	+	3.11E-08	8.09E-07
↳proteasome complex	39	16	1.00	16.05	+	2.17E-13	1.26E-11
↳endopeptidase complex	39	16	1.00	16.05	+	2.17E-13	1.41E-11
↳peptidase complex	55	16	1.41	11.38	+	1.62E-11	7.01E-10
↳intracellular part	6691	208	170.99	1.22	+	1.02E-03	1.02E-02
↳intracellular	6721	209	171.76	1.22	+	1.03E-03	1.01E-02
Arp2/3 protein complex	9	4	.23	17.39	+	2.28E-04	2.76E-03
↳cytoskeletal part	486	31	12.42	2.50	+	8.92E-06	1.66E-04
↳cytoskeleton	586	32	14.98	2.14	+	1.09E-04	1.57E-03
↳intracellular non-membrane-bounded organelle	1344	55	34.35	1.60	+	9.72E-04	1.01E-02
↳non-membrane-bounded organelle	1344	55	34.35	1.60	+	9.72E-04	9.91E-03
↳actin cytoskeleton	160	20	4.09	4.89	+	2.64E-08	7.24E-07
chaperonin-containing T-complex	10	4	.26	15.65	+	3.13E-04	3.62E-03
↳cytosolic part	162	27	4.14	6.52	+	2.45E-13	1.27E-11
↳cytoplasmic part	2902	128	74.16	1.73	+	1.27E-09	4.13E-08
↳cytoplasm	4183	190	106.90	1.78	+	3.64E-16	3.16E-14

↳cytosol	753	65	19.24	3.38	+	1.35E-16	1.40E-14
collagen trimer	17	6	.43	13.81	+	1.64E-05	2.84E-04
COPI vesicle coat	9	3	.23	13.04	+	2.86E-03	2.52E-02
↳membrane	3585	63	91.62	.69	-	9.31E-04	9.88E-03
↳COPI-coated vesicle	9	3	.23	13.04	+	2.86E-03	2.57E-02
↳membrane coat	36	5	.92	5.43	+	3.37E-03	2.88E-02
↳coated membrane	36	5	.92	5.43	+	3.37E-03	2.83E-02
↳membrane part	2301	31	58.80	.53	-	5.86E-05	8.97E-04
cytosolic small ribosomal subunit	40	12	1.02	11.74	+	4.25E-09	1.23E-07
↳cytosolic ribosome	104	21	2.66	7.90	+	4.74E-12	2.24E-10
↳ribosome	151	21	3.86	5.44	+	2.16E-09	6.60E-08
↳ribonucleoprotein complex	463	26	11.83	2.20	+	3.19E-04	3.60E-03
↳small ribosomal subunit	57	12	1.46	8.24	+	1.24E-07	3.06E-06
↳ribosomal subunit	143	21	3.65	5.75	+	8.95E-10	3.58E-08
basement membrane	27	6	.69	8.70	+	1.46E-04	2.05E-03
↳collagen-containing extracellular matrix	66	23	1.69	13.64	+	2.16E-17	2.80E-15
↳extracellular matrix	177	36	4.52	7.96	+	8.16E-20	1.42E-17
↳extracellular region part	1253	106	32.02	3.31	+	2.76E-26	7.19E-24
↳extracellular region	1253	106	32.02	3.31	+	2.76E-26	1.44E-23
cortical actin cytoskeleton	38	6	.97	6.18	+	7.36E-04	7.98E-03
↳cortical cytoskeleton	42	6	1.07	5.59	+	1.18E-03	1.13E-02
↳cell cortex part	91	8	2.33	3.44	+	3.33E-03	2.89E-02
↳cell cortex	121	9	3.09	2.91	+	5.34E-03	4.27E-02
actin filament	70	11	1.79	6.15	+	5.23E-06	1.05E-04
↳polymeric cytoskeletal fiber	246	19	6.29	3.02	+	4.06E-05	6.40E-04
↳supramolecular fiber	298	24	7.62	3.15	+	2.09E-06	4.36E-05
↳supramolecular polymer	301	25	7.69	3.25	+	7.65E-07	1.73E-05
↳supramolecular complex	301	25	7.69	3.25	+	7.65E-07	1.66E-05
cytosolic large ribosomal subunit	64	9	1.64	5.50	+	8.17E-05	1.21E-03
↳large ribosomal subunit	86	9	2.20	4.10	+	6.10E-04	6.75E-03
adherens junction	93	8	2.38	3.37	+	3.77E-03	3.11E-02
↳anchoring junction	102	8	2.61	3.07	+	6.30E-03	4.96E-02
extracellular space	1169	82	29.87	2.74	+	8.35E-16	6.20E-14
Unclassified	12713	251	324.89	.77	-	9.62E-10	3.57E-08
integral component of plasma membrane	770	5	19.68	.25	-	1.51E-04	2.06E-03
↳integral component of membrane	1114	8	28.47	.28	-	1.18E-05	2.11E-04
↳intrinsic component of membrane	1141	8	29.16	.27	-	6.02E-06	1.16E-04
↳intrinsic component of plasma membrane	777	5	19.86	.25	-	1.52E-04	2.03E-03
↳plasma membrane part	1622	17	41.45	.41	-	2.30E-05	3.75E-04
nucleoplasm	479	3	12.24	.25	-	4.36E-03	3.54E-02
↳nuclear lumen	868	5	22.18	.23	-	2.06E-05	3.45E-04
↳nuclear part	1243	7	31.77	.22	-	1.83E-07	4.34E-06
↳nucleus	2974	51	76.00	.67	-	1.70E-03	1.58E-02
↳intracellular organelle lumen	951	8					

			24.30	.33	-	1.90E-04	2.41E-03
↳organelle lumen	951	8	24.30	.33	-	1.90E-04	2.36E-03
↳membrane-enclosed lumen	951	8	24.30	.33	-	1.90E-04	2.47E-03
chromosomal part	364	1	9.30	.11	-	1.88E-03	1.72E-02
↳chromosome	376	1	9.61	.10	-	1.29E-03	1.22E-02
transferase complex	444	1	11.35	.09	-	3.00E-04	3.55E-03

© Copyright 2020 Paul Thomas All Rights Reserved.

Analysis Summary: Please report in publication

BM-MSC secretome from ND mice

Analysis Type: PANTHER Overrepresentation Test (Released 20190711)

Annotation Version and Release Date: PANTHER version 15 Released 2020-02-14

Analyzed List: Client Text Box Input (Mus musculus) Change

Reference List: Mus musculus (all genes in database) Change

Annotation Data Set: PANTHER GO-Slim Cellular Component

Test Type: Fisher's Exact Binomial

Correction: Calculate False Discovery Rate Use the Bonferroni correction for multiple testing No correction

Results

	Reference list	Client Text Box Input
Uniquely Mapped IDS:	22265 out of 22265	482 out of 482
Unmapped IDs:	0	3
Multiple mapping information:	0	9

Export Table XML with user input ids JSON with user input ids View: -- Please select a chart to display --

Displaying only results for FDR P < 0.05, click here to display all results

	Mus musculus (REF)	Client Text Box Input (Hierarchy NEW!)					
PANTHER GO-Slim Cellular Component	#	#	expected	Fold Enrichment	+/-	raw P value	FDR
proteasome core complex, alpha-subunit complex	8	6	.17	34.64	+	2.28E-07	7.91E-06
↳proteasome complex	39	12	.84	14.21	+	5.37E-10	3.49E-08
↳endopeptidase complex	39	12	.84	14.21	+	5.37E-10	3.99E-08
↳peptidase complex	55	12	1.19	10.08	+	1.49E-08	7.05E-07
Arp2/3 protein complex	9	4	.19	20.53	+	1.22E-04	1.87E-03
↳cytoskeletal part	486	24	10.52	2.28	+	2.70E-04	3.80E-03
↳cytoskeleton	586	24	12.69	1.89	+	3.85E-03	3.64E-02
↳actin cytoskeleton	160	19	3.46	5.49	+	9.47E-09	4.92E-07
collagen trimer	17	7	.37	19.02	+	4.65E-07	1.51E-05
chaperonin-containing T-complex	10	3	.22	13.86	+	2.31E-03	2.31E-02
↳cytosolic part	162	20	3.51	5.70	+	2.10E-09	1.21E-07
↳cytoplasmic part	2902	96	62.82	1.53	+	2.99E-05	5.75E-04
↳cytoplasm	4183	140	90.55	1.55	+	7.55E-08	3.02E-06
↳cytosol	753	49	16.30	3.01	+	3.34E-11	2.90E-09

basement membrane	27	6	.58	10.27	+	5.98E-05	9.71E-04
↳collagen-containing extracellular matrix	66	22	1.43	15.40	+	7.74E-18	8.05E-16
↳extracellular matrix	177	34	3.83	8.87	+	2.66E-20	3.46E-18
↳extracellular region part	1253	107	27.13	3.94	+	3.61E-33	9.39E-31
↳extracellular region	1253	107	27.13	3.94	+	3.61E-33	1.88E-30
cytosolic small ribosomal subunit	40	7	.87	8.08	+	5.55E-05	9.31E-04
↳cytosolic ribosome	104	15	2.25	6.66	+	3.46E-08	1.50E-06
↳ribosome	151	15	3.27	4.59	+	2.69E-06	7.36E-05
↳ribonucleoprotein complex	463	22	10.02	2.19	+	1.06E-03	1.26E-02
↳small ribosomal subunit	57	7	1.23	5.67	+	4.02E-04	5.23E-03
↳ribosomal subunit	143	15	3.10	4.85	+	1.44E-06	4.17E-05
actin filament	70	12	1.52	7.92	+	1.55E-07	5.74E-06
↳polymeric cytoskeletal fiber	246	14	5.33	2.63	+	1.39E-03	1.48E-02
↳supramolecular fiber	298	19	6.45	2.95	+	5.22E-05	9.36E-04
↳supramolecular polymer	301	20	6.52	3.07	+	1.92E-05	3.99E-04
↳supramolecular complex	301	20	6.52	3.07	+	1.92E-05	3.84E-04
cortical actin cytoskeleton	38	6	.82	7.29	+	3.13E-04	4.17E-03
↳cortical cytoskeleton	42	6	.91	6.60	+	5.07E-04	6.42E-03
↳cell cortex part	91	9	1.97	4.57	+	2.76E-04	3.78E-03
↳cell cortex	121	9	2.62	3.44	+	1.83E-03	1.87E-02
cytosolic large ribosomal subunit	64	8	1.39	5.77	+	1.40E-04	2.08E-03
↳large ribosomal subunit	86	8	1.86	4.30	+	8.68E-04	1.05E-02
lysosome	114	9	2.47	3.65	+	1.25E-03	1.44E-02
↳lytic vacuole	115	9	2.49	3.62	+	1.32E-03	1.46E-02
↳intracellular membrane-bounded organelle	4662	73	100.92	.72	-	1.77E-03	1.84E-02
↳membrane-bounded organelle	4721	76	102.20	.74	-	3.30E-03	3.24E-02
extracellular space	1169	83	25.31	3.28	+	1.11E-20	1.92E-18
Unclassified	12713	226	275.22	.82	-	9.47E-06	2.34E-04
nuclear lumen	868	7	18.79	.37	-	3.69E-03	3.55E-02
↳nuclear part	1243	10	26.91	.37	-	2.54E-04	3.67E-03
↳nucleus	2974	41	64.38	.64	-	1.37E-03	1.48E-02
integral component of plasma membrane	770	2	16.67	.12	-	1.77E-05	3.83E-04
↳integral component of membrane	1114	7	24.12	.29	-	6.87E-05	1.08E-03
↳intrinsic component of membrane	1141	7	24.70	.28	-	5.09E-05	9.46E-04
↳membrane part	2301	20	49.81	.40	-	1.31E-06	4.01E-05
↳membrane	3585	46	77.61	.59	-	5.30E-05	9.19E-04
↳intrinsic component of plasma membrane	777	2	16.82	.12	-	1.19E-05	2.69E-04
↳plasma membrane part	1622	12	35.11	.34	-	6.91E-06	1.80E-04
↳plasma membrane	3243	45	70.21	.64	-	8.17E-04	1.01E-02
transferase complex	444	1	9.61	.10	-	1.30E-03	1.47E-02
organelle envelope	283	0	6.13	< 0.01	-	5.28E-03	4.81E-02
↳envelope	283	0	6.13	< 0.01	-	5.28E-03	4.90E-02

© Copyright 2020 Paul Thomas All Rights Reserved.

Analysis Summary: Please report in publication vWAT-MSC secretome from ND mice

Analysis Type: PANTHER Overrepresentation Test (Released 20190711)

Annotation Version and Release Date: PANTHER version 15 Released 2020-02-14

Analyzed List: Client Text Box Input (Mus musculus) Change

Reference List: Mus musculus (all genes in database) Change

Annotation Data Set: PANTHER Protein Class

Test Type: (•) Fisher's Exact ( ) Binomial

Correction: (•) Calculate False Discovery Rate ( ) Use the Bonferroni correction for multiple testing ( ) No correction

Results

	Reference list	Client Text Box Input
Uniquely Mapped IDS:	22265 out of 22265	361 out of 363
Unmapped IDs:	0	0
Multiple mapping information:	0	5

Export Table XML with user input ids JSON with user input ids View: -- Please select a chart to display --

Displaying only results for FDR P < 0.05, click here to display all results

	Mus musculus (REF)	Client Text Box Input (Hierarchy NEW!)					
PANTHER Protein Class	#	#	expected	Fold Enrichment	+/-	raw P value	FDR
Hsp90 family chaperone	4	3	.07	46.00	+	1.37E-04	1.40E-03
↳chaperone	74	8	1.21	6.63	+	5.15E-05	5.58E-04
non-motor actin binding protein	93	17	1.52	11.21	+	2.12E-12	8.25E-11
↳actin or actin-binding cytoskeletal protein	236	33	3.85	8.58	+	1.04E-19	2.03E-17
↳cytoskeletal protein	492	36	8.02	4.49	+	3.03E-13	1.48E-11
peroxidase	23	4	.37	10.67	+	8.54E-04	7.24E-03
↳oxidoreductase	418	24	6.81	3.52	+	2.37E-07	4.20E-06
↳metabolite interconversion enzyme	1428	50	23.28	2.15	+	5.29E-07	7.94E-06
actin and actin related protein	30	5	.49	10.22	+	2.26E-04	2.20E-03
calmodulin-related	62	8	1.01	7.91	+	1.61E-05	1.85E-04
↳calcium-binding protein	79	13	1.29	10.09	+	2.68E-09	6.54E-08
extracellular matrix structural protein	73	9	1.19	7.56	+	6.66E-06	8.12E-05
↳extracellular matrix protein	161	14	2.62	5.33	+	9.63E-07	1.34E-05

protease inhibitor	157	18	2.56	7.03	+	4.95E-10	1.61E-08
↳protein-binding activity modulator	632	28	10.30	2.72	+	3.30E-06	4.29E-05
ribosomal protein	159	16	2.59	6.17	+	2.50E-08	5.42E-07
↳translational protein	288	23	4.70	4.90	+	1.43E-09	3.99E-08
reductase	51	5	.83	6.01	+	2.02E-03	1.46E-02
dehydrogenase	119	8	1.94	4.12	+	1.04E-03	8.44E-03
translation factor	95	6	1.55	3.87	+	5.78E-03	3.76E-02
Unclassified	12347	131	201.30	.65	-	2.46E-13	1.60E-11
immunoglobulin	389	0	6.34	< 0.01	-	3.42E-03	2.30E-02
↳defense/immunity protein	600	1	9.78	.10	-	1.35E-03	1.02E-02
C2H2 zinc finger transcription factor	424	0	6.91	< 0.01	-	2.46E-03	1.71E-02
↳zinc finger transcription factor	502	0	8.18	< 0.01	-	4.68E-04	4.15E-03
↳DNA-binding transcription factor	970	0	15.81	< 0.01	-	2.52E-07	4.10E-06
↳gene-specific transcriptional regulator	1054	0	17.18	< 0.01	-	4.67E-08	9.11E-07
G-protein coupled receptor	568	0	9.26	< 0.01	-	2.33E-04	2.16E-03
↳transmembrane signal receptor	712	2	11.61	.17	-	1.26E-03	9.79E-03

© Copyright 2020 Paul Thomas All Rights Reserved.

Analysis Summary: Please report in publication sWAT-MSC secretome from ND mice

Analysis Type: PANTHER Overrepresentation Test (Released 20190711)

Annotation Version and Release Date: PANTHER version 15 Released 2020-02-14

Analyzed List: Client Text Box Input (Mus musculus) Change

Reference List: Mus musculus (all genes in database) Change

Annotation Data Set: PANTHER Protein Class

Test Type: Fisher's Exact Binomial

Correction: Calculate False Discovery Rate Use the Bonferroni correction for multiple testing No correction

Results

	Reference list	Client Text Box Input
Uniquely Mapped IDS:	22265 out of 22265	572 out of 569
Unmapped IDs:	0	1
Multiple mapping information:	0	15

Export Table XML with user input ids JSON with user input ids View: -- Please select a chart to display --

Displaying only results for FDR P < 0.05, click here to display all results

	Mus musculus (REF)	Client Text Box Input (Hierarchy NEW!)					
PANTHER Protein Class	#	#	expected	Fold Enrichment	+/-	raw P value	FDR
Hsp90 family chaperone	4	3	.10	29.35	+	5.00E-04	3.36E-03
↳chaperone	74	14	1.89	7.40	+	3.59E-08	5.39E-07
chaperonin	13	7	.33	21.07	+	3.36E-07	4.68E-06
tubulin	20	6	.51	11.74	+	3.51E-05	3.11E-04
↳cytoskeletal protein	492	49	12.57	3.90	+	6.68E-15	3.26E-13
extracellular matrix structural protein	73	18	1.87	9.65	+	9.20E-12	2.99E-10
↳extracellular matrix protein	161	28	4.11	6.81	+	3.45E-14	1.35E-12
non-motor actin binding protein	93	17	2.38	7.15	+	1.97E-09	3.19E-08
↳actin or actin-binding cytoskeletal protein	236	37	6.03	6.13	+	4.91E-17	3.19E-15
aminoacyl-tRNA synthetase	34	6	.87	6.91	+	4.35E-04	3.03E-03
↳translational protein	288	32	7.36	4.35	+	2.91E-11	7.09E-10
actin and actin related protein	30	5	.77	6.52	+	1.65E-03	9.47E-03
calmodulin-related	62	10	1.58	6.31	+	1.14E-05	1.11E-04

↳calcium-binding protein	79	16	2.02	7.93	+	1.61E-09	2.85E-08
protease inhibitor	157	24	4.01	5.98	+	2.66E-11	7.41E-10
↳protein-binding activity modulator	632	37	16.15	2.29	+	6.64E-06	7.19E-05
actin binding motor protein	27	4	.69	5.80	+	7.04E-03	3.71E-02
ribosomal protein	159	17	4.06	4.18	+	2.11E-06	2.42E-05
growth factor	90	9	2.30	3.91	+	8.24E-04	5.18E-03
↳intercellular signal molecule	343	20	8.77	2.28	+	9.51E-04	5.80E-03
translation initiation factor	60	6	1.53	3.91	+	5.99E-03	3.24E-02
↳translation factor	95	9	2.43	3.71	+	1.17E-03	6.93E-03
metalloprotease	167	13	4.27	3.05	+	6.06E-04	3.94E-03
↳protease	597	37	15.26	2.43	+	1.83E-06	2.23E-05
oxidoreductase	418	25	10.68	2.34	+	1.53E-04	1.15E-03
↳metabolite interconversion enzyme	1428	64	36.49	1.75	+	2.16E-05	2.01E-04
serine protease	229	13	5.85	2.22	+	1.01E-02	4.94E-02
Unclassified	12347	203	315.54	.64	-	1.06E-20	2.06E-18
nucleic acid binding protein	858	9	21.93	.41	-	2.60E-03	1.45E-02
transporter	687	7	17.56	.40	-	8.71E-03	4.35E-02
immunoglobulin	389	0	9.94	< 0.01	-	9.86E-05	8.01E-04
↳defense/immunity protein	600	3	15.33	.20	-	2.89E-04	2.09E-03
helix-turn-helix transcription factor	213	0	5.44	< 0.01	-	7.28E-03	3.74E-02
↳DNA-binding transcription factor	970	1	24.79	.04	-	6.29E-10	1.36E-08
↳gene-specific transcriptional regulator	1054	2	26.94	.07	-	9.66E-10	1.88E-08
C2H2 zinc finger transcription factor	424	0	10.84	< 0.01	-	4.67E-05	3.96E-04
↳zinc finger transcription factor	502	0	12.83	< 0.01	-	6.64E-06	6.81E-05
G-protein coupled receptor	568	0	14.52	< 0.01	-	8.46E-07	1.10E-05
↳transmembrane signal receptor	712	4	18.20	.22	-	1.29E-04	1.01E-03

© Copyright 2020 Paul Thomas All Rights Reserved.

Analysis Summary: Please report in publication BM-MSC secretome from ND mice

Analysis Type: PANTHER Overrepresentation Test (Released 20190711)

Annotation Version and Release Date: PANTHER version 15 Released 2020-02-14

Analyzed List: Client Text Box Input (Mus musculus) Change

Reference List: Mus musculus (all genes in database) Change

Annotation Data Set: PANTHER Protein Class

Test Type: (•) Fisher's Exact ( ) Binomial

Correction: (•) Calculate False Discovery Rate ( ) Use the Bonferroni correction for multiple testing ( ) No correction

Results

	Reference list	Client Text Box Input
Uniquely Mapped IDS:	22265 out of 22265	482 out of 482
Unmapped IDs:	0	3
Multiple mapping information:	0	9

Export Table XML with user input ids JSON with user input ids View: -- Please select a chart to display --

Displaying only results for FDR P < 0.05, click here to display all results

	Mus musculus (REF)	Client Text Box Input (Hierarchy NEW!)					
PANTHER Protein Class	#	#	expected	Fold Enrichment	+/-	raw P value	FDR
Hsp90 family chaperone	4	3	.09	34.64	+	3.11E-04	2.16E-03
↳chaperone	74	11	1.60	6.87	+	1.79E-06	2.69E-05
chaperonin	13	6	.28	21.32	+	1.88E-06	2.62E-05
extracellular matrix structural protein	73	18	1.58	11.39	+	6.15E-13	2.00E-11
↳extracellular matrix protein	161	30	3.49	8.61	+	9.84E-18	1.92E-15
actin and actin related protein	30	5	.65	7.70	+	8.04E-04	5.06E-03
↳actin or actin-binding cytoskeletal protein	236	34	5.11	6.65	+	7.02E-17	6.85E-15
↳cytoskeletal protein	492	39	10.65	3.66	+	1.83E-11	5.09E-10
protease inhibitor	157	26	3.40	7.65	+	1.83E-14	1.19E-12
↳protein-binding activity modulator	632	31	13.68	2.27	+	4.62E-05	4.29E-04
non-motor actin binding protein	93	15	2.01	7.45	+	9.05E-09	2.21E-07
actin binding motor protein	27	4	.58	6.84	+	3.98E-03	2.28E-02
calmodulin-related	62	9	1.34	6.71	+	1.86E-05	1.91E-04

↳calcium-binding protein	79	13	1.71	7.60	+	7.20E-08	1.28E-06
cytokine	77	8	1.67	4.80	+	4.43E-04	2.98E-03
↳intercellular signal molecule	343	19	7.43	2.56	+	2.91E-04	2.27E-03
metalloprotease	167	13	3.62	3.60	+	1.28E-04	1.09E-03
↳protease	597	34	12.92	2.63	+	7.40E-07	1.20E-05
ribosomal protein	159	12	3.44	3.49	+	3.01E-04	2.26E-03
↳translational protein	288	17	6.23	2.73	+	3.01E-04	2.18E-03
dehydrogenase	119	8	2.58	3.11	+	5.73E-03	3.10E-02
↳oxidoreductase	418	26	9.05	2.87	+	3.47E-06	4.51E-05
↳metabolite interconversion enzyme	1428	57	30.91	1.84	+	1.43E-05	1.55E-04
cysteine protease	136	9	2.94	3.06	+	3.83E-03	2.26E-02
serine protease	229	12	4.96	2.42	+	5.55E-03	3.09E-02
Unclassified	12347	184	267.29	.69	-	5.58E-14	2.72E-12
RNA binding protein	615	4	13.31	.30	-	6.44E-03	3.40E-02
↳nucleic acid binding protein	858	6	18.57	.32	-	1.52E-03	9.26E-03
transporter	687	1	14.87	.07	-	1.01E-05	1.16E-04
immunoglobulin	389	0	8.42	< 0.01	-	4.90E-04	3.19E-03
↳defense/immunity protein	600	1	12.99	.08	-	6.91E-05	6.13E-04
C2H2 zinc finger transcription factor	424	0	9.18	< 0.01	-	2.22E-04	1.80E-03
↳zinc finger transcription factor	502	0	10.87	< 0.01	-	2.97E-05	2.89E-04
↳DNA-binding transcription factor	970	1	21.00	.05	-	3.28E-08	7.10E-07
↳gene-specific transcriptional regulator	1054	2	22.82	.09	-	6.70E-08	1.31E-06
G-protein coupled receptor	568	0	12.30	< 0.01	-	9.44E-06	1.15E-04
non-receptor serine/threonine protein kinase	256	0	5.54	< 0.01	-	7.40E-03	3.80E-02

© Copyright 2020 Paul Thomas All Rights Reserved.

Analysis Summary: Please report in publication vWAT-MSC secretome from ND mice

Analysis Type: PANTHER Overrepresentation Test (Released 20190711)

Annotation Version and Release Date: PANTHER version 15 Released 2020-02-14

Analyzed List: Client Text Box Input (Mus musculus) Change

Reference List: Mus musculus (all genes in database) Change

Annotation Data Set: PANTHER GO-Slim Molecular Function

Test Type: (•) Fisher's Exact ( ) Binomial

Correction: Calculate False Discovery Rate Use the Bonferroni correction for multiple testing No correction

Results

	Reference list	Client Text Box Input
Uniquely Mapped IDS:	22265 out of 22265	361 out of 363
Unmapped IDs:	0	0
Multiple mapping information:	0	5

Export Table XML with user input ids JSON with user input ids View: -- Please select a chart to display --

Displaying only results for FDR P < 0.05, click here to display all results

	Mus musculus (REF)	Client Text Box Input (Hierarchy NEW!)					
PANTHER GO-Slim Molecular Function	#	#	expected	Fold Enrichment	+/-	raw P value	FDR
extracellular matrix binding	10	4	.16	24.53	+	5.74E-05	7.26E-04
↳binding	4859	128	79.22	1.62	+	6.64E-09	2.35E-07
actin filament binding	84	17	1.37	12.41	+	5.00E-13	4.42E-11
↳actin binding	129	21	2.10	9.98	+	3.93E-14	4.18E-12
↳cytoskeletal protein binding	340	26	5.54	4.69	+	2.83E-10	1.50E-08
↳protein binding	2575	79	41.98	1.88	+	5.77E-08	1.70E-06
↳protein-containing complex binding	283	30	4.61	6.50	+	4.77E-15	6.33E-13
unfolded protein binding	45	9	.73	12.27	+	1.79E-07	4.76E-06
oxidoreductase activity, acting on peroxide as acceptor	36	6	.59	10.22	+	5.27E-05	6.82E-04
↳oxidoreductase activity	443	24	7.22	3.32	+	6.38E-07	1.36E-05
↳catalytic activity	3715	114	60.57	1.88	+	9.08E-12	6.89E-10
isomerase activity	85	14	1.39	10.10	+	6.46E-10	2.64E-08
ATP binding	32	5	.52	9.58	+	2.95E-04	3.20E-03
↳purine ribonucleotide binding	167	12	2.72	4.41	+	3.41E-05	4.77E-04
↳ribonucleotide binding	173	12	2.82	4.25	+	4.71E-05	6.25E-04
↳nucleotide binding	220	14	3.59	3.90	+	2.76E-05	3.96E-04
↳nucleoside phosphate binding	220	14	3.59	3.90	+	2.76E-05	4.07E-04
↳heterocyclic compound binding	1638	42	26.71	1.57	+	4.43E-03	3.74E-02
↳organic cyclic compound binding	1680	43	27.39	1.57	+	3.67E-03	3.19E-02

↳small molecule binding	358	18	5.84	3.08	+	4.15E-05	5.65E-04
↳carbohydrate derivative binding	242	17	3.95	4.31	+	1.10E-06	2.16E-05
↳purine nucleotide binding	179	12	2.92	4.11	+	6.42E-05	7.93E-04
↳purine ribonucleoside triphosphate binding	142	8	2.32	3.46	+	2.97E-03	2.63E-02
↳anion binding	473	19	7.71	2.46	+	4.14E-04	4.40E-03
↳ion binding	711	34	11.59	2.93	+	5.06E-08	1.68E-06
↳drug binding	117	11	1.91	5.77	+	7.10E-06	1.11E-04
integrin binding	28	4	.46	8.76	+	1.64E-03	1.62E-02
↳cell adhesion molecule binding	70	7	1.14	6.13	+	2.37E-04	2.68E-03
structural constituent of ribosome	128	16	2.09	7.67	+	1.48E-09	5.61E-08
↳structural molecule activity	218	22	3.55	6.19	+	5.54E-11	3.27E-09
metalloendopeptidase activity	42	5	.68	7.30	+	9.12E-04	9.49E-03
↳endopeptidase activity	355	37	5.79	6.39	+	4.36E-18	2.31E-15
↳peptidase activity, acting on L-amino acid peptides	454	39	7.40	5.27	+	2.20E-16	5.84E-14
↳peptidase activity	463	39	7.55	5.17	+	4.03E-16	7.14E-14
↳hydrolase activity	1631	65	26.59	2.44	+	5.25E-11	3.49E-09
↳catalytic activity, acting on a protein	1447	53	23.59	2.25	+	5.52E-08	1.72E-06
↳metallopeptidase activity	75	6	1.22	4.91	+	1.93E-03	1.80E-02
endopeptidase inhibitor activity	108	12	1.76	6.82	+	5.40E-07	1.25E-05
↳peptidase inhibitor activity	108	12	1.76	6.82	+	5.40E-07	1.20E-05
↳peptidase regulator activity	114	13	1.86	6.99	+	1.38E-07	3.85E-06
↳enzyme regulator activity	389	21	6.34	3.31	+	3.40E-06	5.48E-05
↳molecular function regulator	728	26	11.87	2.19	+	2.87E-04	3.18E-03
↳enzyme inhibitor activity	149	14	2.43	5.76	+	4.07E-07	9.81E-06
protease binding	119	13	1.94	6.70	+	2.17E-07	5.50E-06
↳enzyme binding	618	22	10.08	2.18	+	1.04E-03	1.05E-02
serine-type endopeptidase activity	148	12	2.41	4.97	+	1.11E-05	1.69E-04
↳serine hydrolase activity	152	13	2.48	5.25	+	2.79E-06	4.77E-05
↳hydrolase activity, acting on acid phosphorus-nitrogen bonds	152	13	2.48	5.25	+	2.79E-06	4.63E-05
transition metal ion binding	87	6	1.42	4.23	+	3.87E-03	3.31E-02
↳metal ion binding	219	16	3.57	4.48	+	1.41E-06	2.58E-05
↳cation binding	314	16	5.12	3.13	+	9.51E-05	1.12E-03
calcium ion binding	119	8	1.94	4.12	+	1.04E-03	1.06E-02
RNA binding	488	18	7.96	2.26	+	1.73E-03	1.67E-02
Unclassified	13264	156	216.25	.72	-	2.99E-10	1.32E-08
transmembrane transporter activity	701	2	11.43	.17	-	1.86E-03	1.76E-02
sequence-specific DNA binding	538	1	8.77	.11	-	2.70E-03	2.43E-02
G protein-coupled receptor activity	405	0	6.60	< 0.01	-	2.26E-03	2.07E-02
↳transmembrane signaling receptor activity	1182	2	19.27	.10	-	1.17E-06	2.22E-05
↳signaling receptor activity	1282	3	20.90	.14	-	1.80E-06	3.19E-05
↳molecular transducer activity	1349	3	21.99	.14	-	8.96E-07	1.83E-05
DNA-binding transcription factor activity, RNA polymerase II-specific	366	0	5.97	< 0.01	-	5.08E-03	4.22E-02
↳DNA-binding transcription factor activity	618	0	10.08	< 0.01	-	6.45E-05	7.78E-04
↳transcription regulator activity	772	1	12.59	.08	-	9.98E-05	1.15E-03
protein serine/threonine kinase activity	374	0	6.10	< 0.01	-	5.31E-03	4.34E-02

© Copyright 2020 Paul Thomas All Rights Reserved.

Analysis Summary: Please report in publication

sWAT-MSC secretome from ND mice

Analysis Type: PANTHER Overrepresentation Test (Released 20190711)

Annotation Version and Release Date: PANTHER version 15 Released 2020-02-14

Analyzed List: Client Text Box Input (Mus musculus) Change

Reference List: Mus musculus (all genes in database) Change

Annotation Data Set: PANTHER GO-Slim Molecular Function

Test Type: (•) Fisher's Exact ( ) Binomial

Correction: Calculate False Discovery Rate Use the Bonferroni correction for multiple testing No correction

Results

	Reference list	Client Text Box Input
Uniquely Mapped IDS:	22265 out of 22265	572 out of 569
Unmapped IDs:	0	1
Multiple mapping information:	0	15

Export Table XML with user input ids JSON with user input ids View: -- Please select a chart to display --

Displaying only results for FDR P < 0.05, click here to display all results

	Mus musculus (REF)	Client Text Box Input (Hierarchy NEW!)					
PANTHER GO-Slim Molecular Function	#	#	expected	Fold Enrichment	+/-	raw P value	FDR
extracellular matrix structural constituent	11	5	.28	17.79	+	3.28E-05	3.79E-04
↳structural molecule activity	218	34	5.57	6.10	+	1.08E-15	1.43E-13
extracellular matrix binding	10	4	.26	15.65	+	3.13E-04	2.60E-03
↳binding	4859	185	124.18	1.49	+	6.10E-09	1.71E-07
integrin binding	28	10	.72	13.98	+	2.15E-08	5.72E-07
↳cell adhesion molecule binding	70	15	1.79	8.39	+	2.66E-09	7.86E-08
↳protein binding	2575	122	65.81	1.85	+	4.13E-11	1.99E-09
↳protein-containing complex binding	283	37	7.23	5.12	+	8.74E-15	9.28E-13
unfolded protein binding	45	14	1.15	12.17	+	1.44E-10	5.89E-09
heparin binding	27	6	.69	8.70	+	1.46E-04	1.29E-03
↳glycosaminoglycan binding	42	7	1.07	6.52	+	1.99E-04	1.71E-03
↳carbohydrate derivative binding	242	27	6.18	4.37	+	9.60E-10	3.00E-08
↳anion binding	473	31	12.09	2.56	+	6.83E-06	9.07E-05
↳ion binding	711	53	18.17	2.92	+	2.00E-11	1.06E-09
ATP binding	32	7	.82	8.56	+	4.42E-05	4.79E-04
↳purine ribonucleotide binding	167	20	4.27	4.69	+	5.03E-08	1.16E-06
↳ribonucleotide binding	173	20	4.42	4.52	+	8.53E-08	1.81E-06
↳nucleotide binding	220	22	5.62	3.91	+	2.00E-07	3.66E-06
↳nucleoside phosphate binding	220	22	5.62	3.91	+	2.00E-07	3.79E-06
↳small molecule binding	358	24	9.15	2.62	+	3.64E-05	4.11E-04
↳purine nucleotide binding	179	20	4.57	4.37	+	1.41E-07	2.78E-06
↳purine ribonucleoside triphosphate binding	142	16	3.63	4.41	+	2.26E-06	3.42E-05
↳drug binding	117	13	2.99	4.35	+	2.28E-05	2.88E-04
copper ion binding	19	4	.49	8.24	+	2.32E-03	1.54E-02
↳transition metal ion binding	87	9	2.22	4.05	+	6.59E-04	5.00E-03
↳metal ion binding							

	219	20	5.60	3.57	+	2.60E-06	3.73E-05
↳cation binding	314	22	8.02	2.74	+	4.13E-05	4.57E-04
actin filament binding	84	17	2.15	7.92	+	5.01E-10	1.77E-08
↳actin binding	129	23	3.30	6.98	+	4.34E-12	2.56E-10
↳cytoskeletal protein binding	340	29	8.69	3.34	+	5.93E-08	1.31E-06
rRNA binding	25	5	.64	7.83	+	8.03E-04	5.92E-03
heat shock protein binding	26	5	.66	7.53	+	9.38E-04	6.82E-03
isomerase activity	85	15	2.17	6.91	+	2.66E-08	6.73E-07
↳catalytic activity	3715	163	94.94	1.72	+	3.03E-12	2.01E-10
carboxypeptidase activity	30	5	.77	6.52	+	1.65E-03	1.12E-02
↳peptidase activity, acting on L-amino acid peptides	454	52	11.60	4.48	+	4.54E-18	2.41E-15
↳peptidase activity	463	52	11.83	4.39	+	9.71E-18	2.58E-15
↳hydrolase activity	1631	87	41.68	2.09	+	2.65E-10	1.00E-08
↳catalytic activity, acting on a protein	1447	81	36.98	2.19	+	1.26E-10	5.58E-09
structural constituent of ribosome	128	20	3.27	6.11	+	8.62E-10	2.86E-08
growth factor activity	26	4	.66	6.02	+	6.25E-03	3.91E-02
↳receptor ligand activity	231	16	5.90	2.71	+	5.04E-04	3.93E-03
↳signaling receptor activator activity	232	16	5.93	2.70	+	5.26E-04	4.05E-03
↳receptor regulator activity	255	18	6.52	2.76	+	1.85E-04	1.61E-03
↳molecular function regulator	728	42	18.60	2.26	+	2.57E-06	3.79E-05
metalloendopeptidase activity	42	6	1.07	5.59	+	1.18E-03	8.33E-03
↳endopeptidase activity	355	45	9.07	4.96	+	2.87E-17	5.08E-15
↳metallopeptidase activity	75	10	1.92	5.22	+	4.99E-05	5.10E-04
endopeptidase inhibitor activity	108	12	2.76	4.35	+	4.67E-05	4.96E-04
↳peptidase inhibitor activity	108	12	2.76	4.35	+	4.67E-05	4.87E-04
↳peptidase regulator activity	114	12	2.91	4.12	+	7.54E-05	7.28E-04
↳enzyme regulator activity	389	23	9.94	2.31	+	3.27E-04	2.63E-03
↳enzyme inhibitor activity	149	14	3.81	3.68	+	6.12E-05	6.13E-04
protease binding	119	12	3.04	3.95	+	1.10E-04	1.02E-03
translation regulator activity	63	6	1.61	3.73	+	7.42E-03	4.53E-02
oxidoreductase activity, acting on the CH-OH group of donors, NAD or NADP as acceptor	76	7	1.94	3.60	+	4.67E-03	2.99E-02
↳oxidoreductase activity	443	28	11.32	2.47	+	2.89E-05	3.57E-04
ligase activity	88	8	2.25	3.56	+	2.75E-03	1.80E-02
serine-type endopeptidase activity	148	13	3.78	3.44	+	2.06E-04	1.74E-03
↳serine hydrolase activity	152	16	3.88	4.12	+	5.03E-06	7.02E-05
↳hydrolase activity, acting on acid phosphorus-nitrogen bonds	152	16	3.88	4.12	+	5.03E-06	6.84E-05
GTP binding	110	9	2.81	3.20	+	2.98E-03	1.93E-02
cytokine receptor binding	122	9	3.12	2.89	+	5.61E-03	3.54E-02
Unclassified	13264	255	338.97	.75	-	2.62E-12	2.32E-10
ion transmembrane transporter activity	581	5	14.85	.34	-	6.62E-03	4.09E-02
↳transmembrane transporter activity	701	5	17.91	.28	-	7.82E-04	5.85E-03
↳transporter activity	782	7	19.98	.35	-	1.53E-03	1.05E-02
protein serine/threonine kinase activity	374	1	9.56	.10	-	1.29E-03	9.01E-03
↳protein kinase activity	451	2	11.53	.17	-	1.89E-03	1.27E-02
↳phosphotransferase activity, alcohol group as acceptor	534	3	13.65	.22	-	1.12E-03	8.05E-03
G protein-coupled receptor activity	405	0	10.35	< 0.01	-	6.69E-05	6.58E-04
↳transmembrane signaling receptor activity	1182	5	30.21	.17	-	2.71E-08	6.53E-07
↳signaling receptor activity	1282	9	32.76	.27	-	1.30E-06	2.10E-05
↳molecular transducer activity	1349	9	34.47	.26	-	3.49E-07	5.98E-06
DNA-binding transcription factor activity, RNA polymerase II-specific	366	0	9.35	< 0.01	-	1.43E-04	1.31E-03
↳DNA-binding transcription factor activity	618	0	15.79	< 0.01	-	2.62E-07	4.63E-06
↳transcription regulator activity	772	1	19.73	.05	-	1.04E-07	2.12E-06
RNA polymerase II regulatory region sequence-specific DNA binding	344	0	8.79	< 0.01	-	3.23E-04	2.64E-03
↳transcription regulatory region sequence-specific DNA binding	372	0	9.51	< 0.01	-	1.45E-04	1.30E-03
↳sequence-specific double-stranded DNA binding	395	0	10.09	< 0.01	-	1.05E-04	9.92E-04

↳sequence-specific DNA binding	538	0	13.75	< 0.01	-	1.87E-06	2.92E-05
↳DNA binding	803	2	20.52	.10	-	4.30E-07	7.13E-06
↳double-stranded DNA binding	444	0	11.35	< 0.01	-	1.99E-05	2.58E-04
↳transcription regulatory region DNA binding	428	0	10.94	< 0.01	-	2.96E-05	3.57E-04
↳regulatory region nucleic acid binding	428	0	10.94	< 0.01	-	2.96E-05	3.49E-04
↳RNA polymerase II regulatory region DNA binding	349	0	8.92	< 0.01	-	3.39E-04	2.69E-03

© Copyright 2020 Paul Thomas All Rights Reserved.

Analysis Summary: Please report in publication

BM-MSC secretome from ND mice

Analysis Type: PANTHER Overrepresentation Test (Released 20190711)

Annotation Version and Release Date: PANTHER version 15 Released 2020-02-14

Analyzed List: Client Text Box Input (Mus musculus) Change

Reference List: Mus musculus (all genes in database) Change

Annotation Data Set: PANTHER GO-Slim Molecular Function

Test Type: (•) Fisher's Exact ( ) Binomial

Correction: (•) Calculate False Discovery Rate ( ) Use the Bonferroni correction for multiple testing ( ) No correction

Results

	Reference list	Client Text Box Input
Uniquely Mapped IDS:	22265 out of 22265	482 out of 482
Unmapped IDs:	0	3
Multiple mapping information:	0	9

Export Table XML with user input ids JSON with user input ids View: -- Please select a chart to display --

Displaying only results for FDR P < 0.05, click here to display all results

	Mus musculus (REF)	Client Text Box Input (Hierarchy NEW!)					
PANTHER GO-Slim Molecular Function	#	#	expected	Fold Enrichment	+/-	raw P value	FDR
extracellular matrix binding	10	5	.22	23.10	+	1.05E-05	1.51E-04
↳binding	4859	157	105.19	1.49	+	8.08E-08	2.68E-06
extracellular matrix structural constituent	11	5	.24	21.00	+	1.51E-05	1.95E-04
↳structural molecule activity	218	24	4.72	5.09	+	4.31E-10	3.82E-08
integrin binding	28	8	.61	13.20	+	6.89E-07	1.52E-05
↳cell adhesion molecule binding	70	9	1.52	5.94	+	4.42E-05	5.22E-04
↳protein binding	2575	103	55.74	1.85	+	1.28E-09	9.72E-08
↳protein-containing complex binding	283	32	6.13	5.22	+	2.60E-13	3.45E-11
unfolded protein binding	45	11	.97	11.29	+	2.18E-08	8.25E-07
rRNA binding	25	5	.54	9.24	+	3.85E-04	3.65E-03
heparin binding	27	5	.58	8.55	+	5.25E-04	4.89E-03
↳glycosaminoglycan binding	42	5	.91	5.50	+	3.08E-03	2.13E-02
↳carbohydrate derivative binding	242	17	5.24	3.24	+	4.19E-05	5.05E-04
↳ion binding	711	36	15.39	2.34	+	6.08E-06	9.23E-05
metalloendopeptidase activity	42	7	.91	7.70	+	7.31E-05	8.43E-04
↳endopeptidase activity	355	46	7.69	5.99	+	8.97E-21	4.76E-18
↳peptidase activity, acting on L-amino acid peptides	454	51	9.83	5.19	+	1.86E-20	4.95E-18
↳peptidase activity	463	51	10.02	5.09	+	4.05E-20	7.17E-18
↳hydrolase activity	1631	73	35.31	2.07	+	7.35E-09	3.90E-07
↳catalytic activity	3715	134	80.42	1.67	+	1.79E-09	1.19E-07
↳catalytic activity, acting on a protein	1447	73	31.33	2.33	+	5.86E-11	6.23E-09
↳metallopeptidase activity	75	11	1.62	6.77	+	2.02E-06	3.83E-05
actin filament binding	84	14	1.82	7.70	+	1.96E-08	9.46E-07
↳actin binding	129	18	2.79	6.45	+	2.37E-09	1.40E-07
↳cytoskeletal protein binding	340	22	7.36	2.99	+	1.10E-05	1.53E-04
isomerase activity	85	14	1.84	7.61	+	2.24E-08	7.92E-07

ATP binding	32	5	.69	7.22	+	1.04E-03	8.51E-03
↳purine ribonucleotide binding	167	10	3.62	2.77	+	4.62E-03	2.95E-02
↳ribonucleotide binding	173	10	3.75	2.67	+	5.82E-03	3.59E-02
↳nucleotide binding	220	12	4.76	2.52	+	4.11E-03	2.66E-02
↳nucleoside phosphate binding	220	12	4.76	2.52	+	4.11E-03	2.70E-02
↳purine nucleotide binding	179	10	3.88	2.58	+	7.24E-03	4.32E-02
↳drug binding	117	10	2.53	3.95	+	3.81E-04	3.68E-03
carboxypeptidase activity	30	4	.65	6.16	+	5.58E-03	3.53E-02
growth factor binding	38	5	.82	6.08	+	2.07E-03	1.51E-02
endopeptidase inhibitor activity	108	14	2.34	5.99	+	3.23E-07	8.17E-06
↳peptidase inhibitor activity	108	14	2.34	5.99	+	3.23E-07	7.80E-06
↳peptidase regulator activity	114	15	2.47	6.08	+	1.03E-07	3.22E-06
↳enzyme regulator activity	389	20	8.42	2.37	+	6.50E-04	5.95E-03
↳molecular function regulator	728	35	15.76	2.22	+	2.76E-05	3.41E-04
↳enzyme inhibitor activity	149	15	3.23	4.65	+	2.31E-06	3.96E-05
protease binding	119	15	2.58	5.82	+	1.71E-07	4.54E-06
↳enzyme binding	618	24	13.38	1.79	+	7.53E-03	4.44E-02
structural constituent of ribosome	128	14	2.77	5.05	+	2.06E-06	3.77E-05
serine-type endopeptidase activity	148	15	3.20	4.68	+	2.14E-06	3.79E-05
↳serine hydrolase activity	152	17	3.29	5.17	+	1.26E-07	3.70E-06
↳hydrolase activity, acting on acid phosphorus-nitrogen bonds	152	17	3.29	5.17	+	1.26E-07	3.51E-06
oxidoreductase activity, acting on the CH-OH group of donors, NAD or NADP as acceptor	76	7	1.65	4.25	+	1.91E-03	1.43E-02
↳oxidoreductase activity	443	28	9.59	2.92	+	1.09E-06	2.31E-05
cysteine-type endopeptidase activity	93	8	2.01	3.97	+	1.39E-03	1.07E-02
transition metal ion binding	87	7	1.88	3.72	+	3.86E-03	2.59E-02
↳metal ion binding	219	18	4.74	3.80	+	3.41E-06	5.48E-05
↳cation binding	314	18	6.80	2.65	+	2.83E-04	2.89E-03
cytokine activity	116	9	2.51	3.58	+	1.40E-03	1.06E-02
↳receptor ligand activity	231	14	5.00	2.80	+	7.88E-04	6.75E-03
↳signaling receptor activator activity	232	14	5.02	2.79	+	8.20E-04	6.91E-03
↳receptor regulator activity	255	14	5.52	2.54	+	1.91E-03	1.41E-02
calcium ion binding	119	8	2.58	3.11	+	5.73E-03	3.58E-02
cytokine receptor binding	122	8	2.64	3.03	+	6.57E-03	3.97E-02
Unclassified	13264	225	287.14	.78	-	2.08E-08	8.49E-07
G protein-coupled receptor activity	405	0	8.77	< 0.01	-	3.26E-04	3.21E-03
↳transmembrane signaling receptor activity	1182	5	25.59	.20	-	1.27E-06	2.59E-05
↳signaling receptor activity	1282	7	27.75	.25	-	3.61E-06	5.65E-05
↳molecular transducer activity	1349	7	29.20	.24	-	1.32E-06	2.60E-05
ion channel activity	292	0	6.32	< 0.01	-	3.37E-03	2.29E-02
↳inorganic molecular entity transmembrane transporter activity	550	0	11.91	< 0.01	-	1.40E-05	1.86E-04
↳transmembrane transporter activity	701	0	15.18	< 0.01	-	3.77E-07	8.70E-06
↳transporter activity	782	2	16.93	.12	-	1.19E-05	1.62E-04
↳ion transmembrane transporter activity	581	0	12.58	< 0.01	-	6.16E-06	9.09E-05
↳channel activity	308	0	6.67	< 0.01	-	2.26E-03	1.60E-02
↳passive transmembrane transporter activity	308	0	6.67	< 0.01	-	2.26E-03	1.62E-02
metal ion transmembrane transporter activity	310	0	6.71	< 0.01	-	2.27E-03	1.59E-02
↳inorganic cation transmembrane transporter activity	395	0	8.55	< 0.01	-	3.16E-04	3.16E-03
↳cation transmembrane transporter activity	424	0	9.18	< 0.01	-	2.22E-04	2.45E-03
DNA-binding transcription factor activity, RNA polymerase II-specific	366	0	7.92	< 0.01	-	7.04E-04	6.33E-03
↳DNA-binding transcription factor activity	618	0	13.38	< 0.01	-	2.80E-06	4.65E-05
↳transcription regulator activity	772	2	16.71	.12	-	1.79E-05	2.26E-04
RNA polymerase II regulatory region sequence-specific DNA binding	344	0	7.45	< 0.01	-	1.03E-03	8.54E-03
↳transcription regulatory region sequence-specific DNA binding	372	0	8.05	< 0.01	-	7.33E-04	6.49E-03
↳sequence-specific double-stranded DNA binding	395	1	8.55	.12	-	4.00E-03	2.65E-02
↳sequence-specific DNA binding	538	1	11.65	.09	-	2.05E-04	2.32E-03

↳DNA binding	803	4	17.38	.23	-	2.50E-04	2.61E-03
↳double-stranded DNA binding	444	1	9.61	.10	-	1.30E-03	1.03E-02
↳transcription regulatory region DNA binding	428	0	9.27	< 0.01	-	2.30E-04	2.49E-03
↳regulatory region nucleic acid binding	428	0	9.27	< 0.01	-	2.30E-04	2.44E-03
↳RNA polymerase II regulatory region DNA binding	349	0	7.56	< 0.01	-	1.05E-03	8.43E-03
monovalent inorganic cation transmembrane transporter activity	261	0	5.65	< 0.01	-	7.59E-03	4.43E-02
protein serine/threonine kinase activity	374	0	8.10	< 0.01	-	7.47E-04	6.50E-03
↳protein kinase activity	451	1	9.76	.10	-	1.34E-03	1.05E-02
↳phosphotransferase activity, alcohol group as acceptor	534	3	11.56	.26	-	5.85E-03	3.57E-02

© Copyright 2020 Paul Thomas All Rights Reserved.

GENEONTOLOGY Unifying Biology   PANTHER Classification System

LOGIN REGISTER CONTACT US

Home About PANTHER Data PANTHER Tools PANTHER Services Workspace Downloads Help/Tutorial

PANTHER 15.0 released!

Analysis Summary: Please report in publication

vWAT-MSC secretome from ND mice

Analysis Type: PANTHER Overrepresentation Test (Released 20190711)

Annotation Version and Release Date: PANTHER version 15 Released 2020-02-14

Analyzed List: Client Text Box Input (Mus musculus) Change

Reference List: Mus musculus (all genes in database) Change

Annotation Data Set: PANTHER GO-Slim Biological Process

Test Type: (•) Fisher's Exact ( ) Binomial

Correction: (•) Calculate False Discovery Rate ( ) Use the Bonferroni correction for multiple testing ( ) No correction

Results

	Reference list	Client Text Box Input
Uniquely Mapped IDS:	22265 out of 22265	361 out of 363
Unmapped IDs:	0	0
Multiple mapping information:	0	5

Export Table XML with user input ids JSON with user input ids View: -- Please select a chart to display --

Displaying only results for FDR P < 0.05, click here to display all results

	Mus musculus (REF)	Client Text Box Input (Hierarchy NEW!)					
PANTHER GO-Slim Biological Process	#	#	expected	Fold Enrichment	+/-	raw P value	FDR
tissue remodeling	3	2	.05	40.89	+	2.49E-03	3.51E-02
response to reactive oxygen species	11	5	.18	27.88	+	3.90E-06	1.55E-04
↳response to oxidative stress	25	9	.41	22.08	+	2.35E-09	3.46E-07
cellular response to oxidative stress	16	6	.26	23.00	+	9.82E-07	5.48E-05
↳cellular process	7925	180	129.21	1.39	+	7.15E-08	7.03E-06
↳cellular response to stress	444	16	7.24	2.21	+	3.85E-03	4.85E-02
glucose 6-phosphate metabolic process	9	3	.15	20.45	+	8.09E-04	1.40E-02
↳organophosphate metabolic process	312	15	5.09	2.95	+	2.84E-04	5.86E-03
↳cellular metabolic process	4357	109	71.03	1.53	+	2.77E-06	1.30E-04
↳metabolic process	4857	115	79.19	1.45	+	1.67E-05	5.40E-04
↳organic substance metabolic process	4680	106	76.30	1.39	+	2.71E-04	5.82E-03
Arp2/3 complex-mediated actin nucleation	18	6	.29	20.45	+	1.72E-06	9.13E-05
↳positive regulation of actin filament polymerization	34	6	.55	10.82	+	3.96E-05	1.09E-03
↳regulation of actin filament polymerization	71	10	1.16	8.64	+	6.82E-07	4.40E-05
↳regulation of protein polymerization	75	10	1.22	8.18	+	1.07E-06	5.83E-05
↳regulation of protein complex assembly	94	10	1.53	6.53	+	6.84E-06	2.48E-04
↳regulation of cellular component organization	396	17	6.46	2.63	+	4.03E-04	8.01E-03
↳regulation of cellular component biogenesis	140	10	2.28	4.38	+	1.60E-04	3.63E-03
↳regulation of actin polymerization or depolymerization	73	11	1.19	9.24	+	1.02E-07	8.74E-06
↳regulation of actin filament organization	81	11	1.32	8.33	+	2.62E-07	2.00E-05
↳regulation of actin cytoskeleton organization	106	12	1.73	6.94	+	4.50E-07	3.32E-05
↳regulation of actin filament-based process	107	12	1.74	6.88	+	4.93E-07	3.39E-05
↳regulation of cytoskeleton organization	146	12	2.38	5.04	+	9.79E-06	3.37E-04

↳regulation of organelle organization	264	15	4.30	3.49	+	4.95E-05	1.31E-03
↳regulation of supramolecular fiber organization	90	11	1.47	7.50	+	6.82E-07	4.27E-05
↳regulation of actin filament length	73	11	1.19	9.24	+	1.02E-07	9.12E-06
↳regulation of cellular component size	108	12	1.76	6.82	+	5.40E-07	3.60E-05
↳regulation of anatomical structure size	120	13	1.96	6.64	+	2.37E-07	1.89E-05
↳regulation of biological quality	905	28	14.75	1.90	+	1.87E-03	2.80E-02
↳cellular component organization	2396	61	39.06	1.56	+	6.14E-04	1.12E-02
↳cellular component organization or biogenesis	2526	65	41.18	1.58	+	2.38E-04	5.16E-03
↳positive regulation of supramolecular fiber organization	48	8	.78	10.22	+	2.95E-06	1.32E-04
↳positive regulation of cellular component organization	136	11	2.22	4.96	+	2.63E-05	7.77E-04
↳positive regulation of cytoskeleton organization	49	8	.80	10.01	+	3.38E-06	1.42E-04
↳positive regulation of organelle organization	79	9	1.29	6.99	+	1.19E-05	4.04E-04
↳positive regulation of protein polymerization	38	6	.62	9.68	+	6.90E-05	1.72E-03
↳positive regulation of protein complex assembly	43	6	.70	8.56	+	1.28E-04	3.07E-03
↳positive regulation of cellular component biogenesis	64	7	1.04	6.71	+	1.42E-04	3.30E-03
↳actin filament organization	159	21	2.59	8.10	+	1.51E-12	6.23E-10
↳actin cytoskeleton organization	238	23	3.88	5.93	+	4.47E-11	1.15E-08
↳cytoskeleton organization	560	29	9.13	3.18	+	1.05E-07	8.67E-06
↳actin filament-based process	246	23	4.01	5.73	+	8.21E-11	1.69E-08
↳supramolecular fiber organization	236	25	3.85	6.50	+	9.82E-13	5.07E-10
collagen fibril organization	11	3	.18	16.73	+	1.31E-03	2.04E-02
↳extracellular matrix organization	56	7	.91	7.67	+	6.60E-05	1.68E-03
↳extracellular structure organization	63	7	1.03	6.82	+	1.30E-04	3.05E-03
↳protein-containing complex subunit organization	481	28	7.84	3.57	+	1.73E-08	2.10E-06
glycolytic process	20	5	.33	15.33	+	4.22E-05	1.14E-03
↳generation of precursor metabolites and energy	103	11	1.68	6.55	+	2.29E-06	1.10E-04
↳nucleotide phosphorylation	20	5	.33	15.33	+	4.22E-05	1.13E-03
↳organic substance catabolic process	703	43	11.46	3.75	+	4.16E-13	2.86E-10
↳catabolic process	888	51	14.48	3.52	+	2.26E-14	4.66E-11
↳carbohydrate metabolic process	133	9	2.17	4.15	+	4.90E-04	9.54E-03
↳primary metabolic process	4458	99	72.68	1.36	+	9.44E-04	1.57E-02
↳carboxylic acid metabolic process	331	14	5.40	2.59	+	1.47E-03	2.22E-02
↳oxoacid metabolic process	342	14	5.58	2.51	+	1.96E-03	2.89E-02
↳organic acid metabolic process	355	14	5.79	2.42	+	2.72E-03	3.74E-02
↳small molecule metabolic process	587	24	9.57	2.51	+	5.72E-05	1.49E-03
↳purine ribonucleotide metabolic process	130	9	2.12	4.25	+	4.19E-04	8.24E-03
↳purine nucleotide metabolic process	138	9	2.25	4.00	+	6.30E-04	1.13E-02
↳purine-containing compound metabolic process	152	10	2.48	4.04	+	2.98E-04	6.04E-03
↳nitrogen compound metabolic process	4212	97	68.67	1.41	+	3.35E-04	6.70E-03
↳organonitrogen compound metabolic process	2527	82	41.20	1.99	+	2.05E-09	3.26E-07
↳nucleotide metabolic process	163	9	2.66	3.39	+	1.90E-03	2.82E-02
↳nucleoside phosphate metabolic process	168	9	2.74	3.29	+	2.31E-03	3.30E-02
↳nucleobase-containing small molecule metabolic process	202	12	3.29	3.64	+	1.88E-04	4.17E-03
↳ribonucleotide metabolic process	135	9	2.20	4.09	+	5.43E-04	1.01E-02
↳ribose phosphate metabolic process	143	9	2.33	3.86	+	8.00E-04	1.40E-02
↳ATP metabolic process	56	7	.91	7.67	+	6.60E-05	1.66E-03
reactive oxygen species metabolic process	40	7	.65	10.73	+	9.34E-06	3.27E-04
ribosomal large subunit assembly	23	4	.37	10.67	+	8.54E-04	1.43E-02
↳ribonucleoprotein complex assembly	110	8	1.79	4.46	+	6.42E-04	1.14E-02
↳cellular protein-containing complex assembly	367	20	5.98	3.34	+	5.10E-06	1.91E-04
↳protein-containing complex assembly	394	20	6.42	3.11	+	1.37E-05	4.57E-04

↳cellular component assembly	734	29	11.97	2.42	+	2.13E-05	6.77E-04
↳cellular component biogenesis	867	33	14.14	2.33	+	1.55E-05	5.07E-04
↳ribonucleoprotein complex subunit organization	115	8	1.87	4.27	+	8.44E-04	1.44E-02
↳ribonucleoprotein complex biogenesis	238	12	3.88	3.09	+	7.63E-04	1.35E-02
↳ribosomal large subunit biogenesis	63	6	1.03	5.84	+	8.38E-04	1.44E-02
↳ribosome biogenesis	176	11	2.87	3.83	+	2.29E-04	5.03E-03
response to toxic substance	32	5	.52	9.58	+	2.95E-04	6.04E-03
glucose metabolic process	26	4	.42	9.44	+	1.28E-03	2.04E-02
↳hexose metabolic process	36	4	.59	6.82	+	3.78E-03	4.78E-02
chaperone-mediated protein folding	26	4	.42	9.44	+	1.28E-03	2.02E-02
↳protein folding	81	17	1.32	12.87	+	2.98E-13	3.08E-10
actin filament polymerization	74	10	1.21	8.29	+	9.60E-07	5.51E-05
↳protein polymerization	89	10	1.45	6.89	+	4.38E-06	1.67E-04
↳actin polymerization or depolymerization	79	12	1.29	9.32	+	2.45E-08	2.66E-06
actin filament bundle assembly	31	4	.51	7.91	+	2.30E-03	3.32E-02
↳actin filament bundle organization	33	4	.54	7.43	+	2.83E-03	3.85E-02
negative regulation of endopeptidase activity	82	10	1.34	7.48	+	2.24E-06	1.10E-04
↳negative regulation of peptidase activity	87	10	1.42	7.05	+	3.64E-06	1.47E-04
↳negative regulation of hydrolase activity	102	11	1.66	6.61	+	2.10E-06	1.08E-04
↳regulation of hydrolase activity	287	13	4.68	2.78	+	1.20E-03	1.91E-02
↳negative regulation of catalytic activity	169	11	2.76	3.99	+	1.64E-04	3.69E-03
↳negative regulation of molecular function	196	11	3.20	3.44	+	5.44E-04	1.00E-02
↳regulation of peptidase activity	112	12	1.83	6.57	+	7.71E-07	4.68E-05
↳regulation of proteolysis	155	13	2.53	5.14	+	3.40E-06	1.41E-04
↳negative regulation of proteolysis	88	10	1.43	6.97	+	3.99E-06	1.56E-04
↳negative regulation of cellular protein metabolic process	164	11	2.67	4.11	+	1.28E-04	3.04E-03
↳negative regulation of protein metabolic process	168	12	2.74	4.38	+	3.61E-05	1.01E-03
↳regulation of endopeptidase activity	107	12	1.74	6.88	+	4.93E-07	3.51E-05
carbohydrate biosynthetic process	43	5	.70	7.13	+	1.00E-03	1.62E-02
maturation of SSU-rRNA	35	4	.57	7.01	+	3.44E-03	4.41E-02
↳nucleic acid metabolic process	1911	16	31.16	.51	-	3.14E-03	4.24E-02
↳ribosomal small subunit biogenesis	51	5	.83	6.01	+	2.02E-03	2.93E-02
developmental growth	51	5	.83	6.01	+	2.02E-03	2.96E-02
↳growth	55	5	.90	5.58	+	2.74E-03	3.75E-02
response to inorganic substance	54	5	.88	5.68	+	2.55E-03	3.57E-02
response to endoplasmic reticulum stress	65	6	1.06	5.66	+	9.74E-04	1.61E-02
proteasome-mediated ubiquitin-dependent protein catabolic process	194	17	3.16	5.37	+	5.96E-08	6.15E-06
↳proteasomal protein catabolic process	205	18	3.34	5.39	+	2.36E-08	2.70E-06
↳proteolysis involved in cellular protein catabolic process	331	24	5.40	4.45	+	3.73E-09	5.13E-07
↳proteolysis	693	39	11.30	3.45	+	5.91E-11	1.35E-08
↳protein metabolic process	2115	62	34.48	1.80	+	8.60E-06	3.06E-04
↳cellular protein catabolic process	333	24	5.43	4.42	+	4.16E-09	5.37E-07
↳cellular macromolecule catabolic process	429	25	6.99	3.57	+	1.01E-07	9.50E-06
↳macromolecule catabolic process	463	29	7.55	3.84	+	2.03E-09	3.49E-07
↳cellular catabolic process	765	42	12.47	3.37	+	2.13E-11	6.29E-09
↳protein catabolic process	355	27	5.79	4.66	+	1.42E-10	2.67E-08
↳organonitrogen compound catabolic process	457	32	7.45	4.29	+	1.98E-11	6.81E-09
↳cellular protein metabolic process	1711	56	27.90	2.01	+	9.03E-07	5.32E-05
↳ubiquitin-dependent protein catabolic process	276	17	4.50	3.78	+	5.82E-06	2.14E-04
↳modification-dependent protein catabolic process	285	18	4.65	3.87	+	2.22E-06	1.12E-04
↳modification-dependent macromolecule catabolic process	290	18	4.73	3.81	+	2.80E-06	1.28E-04

negative regulation of cellular component organization	88	7	1.43	4.88	+	8.51E-04	1.44E-02
regulation of cell migration	79	6	1.29	4.66	+	2.47E-03	3.51E-02
↳regulation of cell motility	86	6	1.40	4.28	+	3.67E-03	4.67E-02
drug metabolic process	189	13	3.08	4.22	+	2.49E-05	7.56E-04
translational elongation	216	14	3.52	3.98	+	2.28E-05	7.12E-04
↳translation	216	14	3.52	3.98	+	2.28E-05	7.02E-04
↳peptide biosynthetic process	219	14	3.57	3.92	+	2.63E-05	7.87E-04
↳organonitrogen compound biosynthetic process	589	24	9.60	2.50	+	6.01E-05	1.55E-03
↳amide biosynthetic process	252	14	4.11	3.41	+	1.10E-04	2.67E-03
↳cellular amide metabolic process	369	16	6.02	2.66	+	5.35E-04	1.00E-02
↳peptide metabolic process	286	16	4.66	3.43	+	3.33E-05	9.41E-04
small molecule catabolic process	108	7	1.76	3.98	+	2.57E-03	3.56E-02
oxidation-reduction process	220	14	3.59	3.90	+	2.76E-05	8.01E-04
cofactor metabolic process	168	10	2.74	3.65	+	6.27E-04	1.14E-02
small molecule biosynthetic process	155	9	2.53	3.56	+	1.37E-03	2.12E-02
cell migration	255	11	4.16	2.65	+	3.97E-03	4.97E-02
↳cell motility	285	12	4.65	2.58	+	3.24E-03	4.31E-02
↳localization of cell	285	12	4.65	2.58	+	3.24E-03	4.34E-02
Unclassified	12808	164	208.82	.79	-	2.97E-06	1.31E-04
macromolecule modification	1237	8	20.17	.40	-	3.41E-03	4.43E-02
regulation of transcription by RNA polymerase II	745	1	12.15	.08	-	1.43E-04	3.29E-03
↳regulation of transcription, DNA-templated	1117	5	18.21	.27	-	5.34E-04	1.03E-02
↳regulation of gene expression	1368	9	22.30	.40	-	1.83E-03	2.76E-02
↳regulation of nucleic acid-templated transcription	1117	5	18.21	.27	-	5.34E-04	1.02E-02
↳regulation of RNA biosynthetic process	1117	5	18.21	.27	-	5.34E-04	1.01E-02
↳regulation of RNA metabolic process	1222	8	19.92	.40	-	3.37E-03	4.42E-02
↳regulation of nucleobase-containing compound metabolic process	1263	8	20.59	.39	-	2.56E-03	3.57E-02
↳regulation of cellular biosynthetic process	1234	8	20.12	.40	-	3.39E-03	4.43E-02
↳regulation of biosynthetic process	1240	8	20.22	.40	-	3.43E-03	4.43E-02
↳regulation of macromolecule biosynthetic process	1223	7	19.94	.35	-	1.45E-03	2.21E-02
↳regulation of cellular macromolecule biosynthetic process	1216	7	19.83	.35	-	1.43E-03	2.20E-02
transcription by RNA polymerase II	772	1	12.59	.08	-	9.98E-05	2.45E-03
↳transcription, DNA-templated	1159	5	18.90	.26	-	2.71E-04	5.71E-03
↳nucleic acid-templated transcription	1159	5	18.90	.26	-	2.71E-04	5.77E-03
↳RNA biosynthetic process	1166	5	19.01	.26	-	2.76E-04	5.75E-03
G protein-coupled receptor signaling pathway	682	0	11.12	< 0.01	-	3.08E-05	8.83E-04
↳signal transduction	2229	20	36.34	.55	-	3.35E-03	4.43E-02
↳signaling	2365	20	38.56	.52	-	9.91E-04	1.62E-02
↳cell communication	2367	20	38.59	.52	-	9.92E-04	1.61E-02

© Copyright 2020 Paul Thomas All Rights Reserved.

Analysis Summary: Please report in publication

sWAT-MSC secretome from ND mice

Analysis Type: PANTHER Overrepresentation Test (Released 20190711)

Annotation Version and Release Date: PANTHER version 15 Released 2020-02-14

Analyzed List: Client Text Box Input (Mus musculus) Change

Reference List: Mus musculus (all genes in database) Change

Annotation Data Set: PANTHER GO-Slim Biological Process

Test Type: Fisher's Exact Binomial

Correction: Calculate False Discovery Rate Use the Bonferroni correction for multiple testing No correction

Results

	Reference list	Client Text Box Input
Uniquely Mapped IDS:	22265 out of 22265	572 out of 569
Unmapped IDs:	0	1
Multiple mapping information:	0	15

Export Table XML with user input ids JSON with user input ids View: -- Please select a chart to display --

Displaying only results for FDR P < 0.05, click here to display all results

	Mus musculus (REF)	Client Text Box Input (Hierarchy NEW!)					
PANTHER GO-Slim Biological Process	#	#	expected	Fold Enrichment	+/-	raw P value	FDR
tissue remodeling	3	2	.08	26.09	+	5.90E-03	4.93E-02
positive chemotaxis	7	4	.18	22.36	+	1.10E-04	2.26E-03
↳chemotaxis	186	14	4.75	2.95	+	5.18E-04	7.28E-03
↳response to chemical	915	42	23.38	1.80	+	4.12E-04	6.20E-03
↳taxis	186	14	4.75	2.95	+	5.18E-04	7.33E-03
↳locomotion	356	32	9.10	3.52	+	3.82E-09	3.58E-07
sprouting angiogenesis	7	4	.18	22.36	+	1.10E-04	2.24E-03
↳angiogenesis	38	6	.97	6.18	+	7.36E-04	9.38E-03
↳anatomical structure morphogenesis	450	29	11.50	2.52	+	1.66E-05	4.44E-04
↳anatomical structure development	1076	44	27.50	1.60	+	3.00E-03	2.81E-02
↳blood vessel morphogenesis	39	6	1.00	6.02	+	8.32E-04	1.05E-02
↳tube morphogenesis	54	7	1.38	5.07	+	7.83E-04	9.91E-03
↳tube development	57	7	1.46	4.81	+	1.05E-03	1.24E-02
↳multicellular organism development	828	39	21.16	1.84	+	4.87E-04	6.98E-03
↳blood vessel development	42	6	1.07	5.59	+	1.18E-03	1.38E-02
↳vasculature development	45	6	1.15	5.22	+	1.62E-03	1.83E-02
↳system development	737	35	18.83	1.86	+	8.58E-04	1.07E-02
↳cardiovascular system development	46	6	1.18	5.10	+	1.80E-03	1.97E-02
↳circulatory system development	92	9	2.35	3.83	+	9.51E-04	1.15E-02
collagen fibril organization	11	5	.28	17.79	+	3.28E-05	8.27E-04
↳supramolecular fiber organization	236	28	6.03	4.64	+	1.30E-10	3.36E-08
↳cellular component organization	2396	92	61.23	1.50	+	1.25E-04	2.49E-03
↳cellular process	7925	276	202.53	1.36	+	4.83E-10	7.67E-08
↳cellular component organization or biogenesis	2526	96	64.55	1.49	+	1.05E-04	2.19E-03
↳extracellular matrix organization	56	15	1.43	10.48	+	1.87E-10	4.28E-08

↳extracellular structure organization	63	15	1.61	9.32	+	7.60E-10	9.80E-08
↳protein-containing complex subunit organization	481	33	12.29	2.68	+	8.15E-07	3.66E-05
IMP metabolic process	7	3	.18	16.77	+	1.62E-03	1.84E-02
↳small molecule metabolic process	587	29	15.00	1.93	+	1.39E-03	1.60E-02
↳metabolic process	4857	164	124.12	1.32	+	1.16E-04	2.32E-03
↳nucleobase-containing compound metabolic process	2112	32	53.97	.59	-	1.30E-03	1.52E-02
↳heterocycle metabolic process	2151	34	54.97	.62	-	2.36E-03	2.37E-02
↳cellular metabolic process	4357	149	111.35	1.34	+	1.47E-04	2.79E-03
↳organic cyclic compound metabolic process	2218	35	56.68	.62	-	1.70E-03	1.89E-02
↳organic substance metabolic process	4680	155	119.60	1.30	+	4.87E-04	6.93E-03
↳nitrogen compound metabolic process	4212	144	107.64	1.34	+	2.29E-04	3.87E-03
↳cellular aromatic compound metabolic process	2163	35	55.28	.63	-	3.13E-03	2.91E-02
↳organonitrogen compound metabolic process	2527	132	64.58	2.04	+	4.81E-15	9.92E-12
response to hypoxia	12	5	.31	16.30	+	4.56E-05	1.06E-03
↳response to abiotic stimulus	86	8	2.20	3.64	+	2.41E-03	2.41E-02
positive regulation of epithelial cell proliferation	8	3	.20	14.67	+	2.19E-03	2.28E-02
positive regulation of leukocyte chemotaxis	8	3	.20	14.67	+	2.19E-03	2.27E-02
↳positive regulation of leukocyte migration	11	3	.28	10.67	+	4.56E-03	4.01E-02
↳regulation of leukocyte migration	17	5	.43	11.51	+	1.75E-04	3.17E-03
↳regulation of cell migration	79	9	2.02	4.46	+	3.46E-04	5.41E-03
↳regulation of cell motility	86	9	2.20	4.10	+	6.10E-04	8.13E-03
↳regulation of locomotion	91	9	2.33	3.87	+	8.86E-04	1.09E-02
↳regulation of cellular component movement	93	9	2.38	3.79	+	1.02E-03	1.22E-02
↳positive regulation of cell migration	43	6	1.10	5.46	+	1.31E-03	1.52E-02
↳positive regulation of cell motility	47	6	1.20	5.00	+	1.98E-03	2.13E-02
↳positive regulation of cellular component movement	48	6	1.23	4.89	+	2.18E-03	2.30E-02
↳positive regulation of locomotion	48	6	1.23	4.89	+	2.18E-03	2.29E-02
↳positive regulation of chemotaxis	9	3	.23	13.04	+	2.86E-03	2.70E-02
positive regulation of angiogenesis	11	4	.28	14.23	+	4.18E-04	6.26E-03
↳regulation of developmental process	265	15	6.77	2.21	+	5.51E-03	4.68E-02
↳positive regulation of developmental process	109	9	2.79	3.23	+	2.81E-03	2.70E-02
↳regulation of multicellular organismal development	198	13	5.06	2.57	+	2.55E-03	2.50E-02
actin filament depolymerization	11	4	.28	14.23	+	4.18E-04	6.21E-03
↳actin polymerization or depolymerization	79	13	2.02	6.44	+	4.60E-07	2.21E-05
↳actin filament organization	159	20	4.06	4.92	+	2.40E-08	1.84E-06
↳actin cytoskeleton organization	238	24	6.08	3.95	+	4.84E-08	3.22E-06
↳cytoskeleton organization	560	38	14.31	2.66	+	1.54E-07	7.94E-06
↳actin filament-based process	246	25	6.29	3.98	+	2.21E-08	1.76E-06
response to reactive oxygen species	11	4	.28	14.23	+	4.18E-04	6.17E-03
↳response to oxidative stress	25	7	.64	10.96	+	1.13E-05	3.14E-04
bone morphogenesis	14	5	.36	13.98	+	8.22E-05	1.77E-03
↳bone development	21	5	.54	9.32	+	4.02E-04	6.15E-03
↳animal organ development	349	19	8.92	2.13	+	3.29E-03	3.05E-02
↳animal organ morphogenesis	104	10	2.66	3.76	+	5.71E-04	7.91E-03
glucose 6-phosphate metabolic process	9	3	.23	13.04	+	2.86E-03	2.71E-02
cellular response to oxidative stress	16	5	.41	12.23	+	1.38E-04	2.69E-03
↳cellular response to chemical stimulus	641	33	16.38	2.01	+	2.31E-04	3.87E-03
endothelial cell proliferation	10	3	.26	11.74	+	3.65E-03	3.32E-02
↳epithelial cell proliferation	11	3	.28	10.67	+	4.56E-03	4.03E-02
chondrocyte differentiation	17	5	.43	11.51	+	1.75E-04	3.19E-03
↳cartilage development	18	5	.46	10.87	+	2.19E-04	3.77E-03
↳connective tissue development	18	5	.46	10.87	+	2.19E-04	3.80E-03
↳tissue development	179	15	4.57	3.28	+	1.12E-04	2.27E-03
protein stabilization	11	3	.28	10.67	+	4.56E-03	3.99E-02

glycolytic process	20	5	.51	9.78	+	3.32E-04	5.27E-03
↳generation of precursor metabolites and energy	103	11	2.63	4.18	+	1.33E-04	2.61E-03
↳nucleotide phosphorylation	20	5	.51	9.78	+	3.32E-04	5.23E-03
↳organic substance catabolic process	703	48	17.97	2.67	+	2.80E-09	2.75E-07
↳catabolic process	888	54	22.69	2.38	+	1.30E-08	1.07E-06
↳carboxylic acid metabolic process	331	21	8.46	2.48	+	3.54E-04	5.49E-03
↳oxoacid metabolic process	342	21	8.74	2.40	+	4.36E-04	6.39E-03
↳organic acid metabolic process	355	21	9.07	2.31	+	6.07E-04	8.14E-03
↳ATP metabolic process	56	6	1.43	4.19	+	4.40E-03	3.91E-02
Arp2/3 complex-mediated actin nucleation	18	4	.46	8.70	+	1.95E-03	2.11E-02
↳regulation of actin filament polymerization	71	11	1.81	6.06	+	5.90E-06	1.93E-04
↳regulation of protein polymerization	75	11	1.92	5.74	+	9.43E-06	2.74E-04
↳regulation of protein complex assembly	94	12	2.40	5.00	+	1.34E-05	3.63E-04
↳regulation of cellular component organization	396	21	10.12	2.08	+	2.24E-03	2.32E-02
↳regulation of cellular component biogenesis	140	14	3.58	3.91	+	3.27E-05	8.34E-04
↳regulation of actin polymerization or depolymerization	73	12	1.87	6.43	+	1.28E-06	5.41E-05
↳regulation of actin filament organization	81	12	2.07	5.80	+	3.39E-06	1.21E-04
↳regulation of actin cytoskeleton organization	106	13	2.71	4.80	+	8.72E-06	2.65E-04
↳regulation of actin filament-based process	107	14	2.73	5.12	+	1.99E-06	8.06E-05
↳regulation of cytoskeleton organization	146	15	3.73	4.02	+	1.29E-05	3.54E-04
↳regulation of organelle organization	264	16	6.75	2.37	+	2.70E-03	2.60E-02
↳regulation of supramolecular fiber organization	90	12	2.30	5.22	+	8.98E-06	2.69E-04
↳regulation of actin filament length	73	12	1.87	6.43	+	1.28E-06	5.52E-05
↳regulation of cellular component size	108	13	2.76	4.71	+	1.05E-05	2.96E-04
↳regulation of anatomical structure size	120	14	3.07	4.57	+	6.69E-06	2.09E-04
↳positive regulation of supramolecular fiber organization	48	6	1.23	4.89	+	2.18E-03	2.31E-02
↳positive regulation of cellular component organization	136	10	3.48	2.88	+	3.67E-03	3.33E-02
↳positive regulation of cytoskeleton organization	49	6	1.25	4.79	+	2.40E-03	2.41E-02
↳positive regulation of organelle organization	79	7	2.02	3.47	+	5.67E-03	4.77E-02
negative regulation of actin filament polymerization	23	5	.59	8.51	+	5.77E-04	7.84E-03
ribosomal large subunit assembly	23	5	.59	8.51	+	5.77E-04	7.78E-03
↳ribonucleoprotein complex assembly	110	10	2.81	3.56	+	8.53E-04	1.07E-02
↳cellular protein-containing complex assembly	367	24	9.38	2.56	+	5.26E-05	1.21E-03
↳protein-containing complex assembly	394	25	10.07	2.48	+	8.16E-05	1.77E-03
↳cellular component assembly	734	37	18.76	1.97	+	1.44E-04	2.78E-03
↳cellular component biogenesis	867	41	22.16	1.85	+	2.83E-04	4.59E-03
↳ribonucleoprotein complex subunit organization	115	10	2.94	3.40	+	1.17E-03	1.38E-02
↳ribosome biogenesis	176	13	4.50	2.89	+	9.54E-04	1.14E-02
chaperone-mediated protein folding	26	5	.66	7.53	+	9.38E-04	1.14E-02
↳protein folding	81	22	2.07	10.63	+	8.23E-15	8.49E-12
endoplasmic reticulum unfolded protein response	22	4	.56	7.11	+	3.69E-03	3.32E-02
↳response to endoplasmic reticulum stress	65	8	1.66	4.82	+	4.59E-04	6.67E-03
↳cellular response to unfolded protein	29	6	.74	8.10	+	2.05E-04	3.58E-03
↳response to unfolded protein	29	6	.74	8.10	+	2.05E-04	3.61E-03
↳response to topologically incorrect protein	41	7	1.05	6.68	+	1.75E-04	3.22E-03
↳response to organic substance	558	30	14.26	2.10	+	2.29E-04	3.90E-03
↳cellular response to topologically incorrect protein	41	7	1.05	6.68	+	1.75E-04	3.25E-03
↳cellular response to organic substance	488	27	12.47	2.16	+	2.83E-04	4.57E-03
regulation of cellular response to growth factor stimulus	30	5	.77	6.52	+	1.65E-03	1.84E-02
granulocyte chemotaxis	38	6	.97	6.18	+	7.36E-04	9.44E-03
↳myeloid leukocyte migration	48	10	1.23	8.15	+	1.52E-06	6.27E-05
↳leukocyte migration	57	10	1.46	6.86	+	5.90E-06	1.90E-04
↳cell migration	255	28	6.52	4.30	+	6.51E-10	8.96E-08

↳cell motility	285	29	7.28	3.98	+	1.61E-09	1.66E-07
↳movement of cell or subcellular component	549	35	14.03	2.49	+	2.16E-06	8.59E-05
↳localization of cell	285	29	7.28	3.98	+	1.61E-09	1.75E-07
↳leukocyte chemotaxis	47	9	1.20	7.49	+	9.28E-06	2.74E-04
↳cell chemotaxis	81	9	2.07	4.35	+	4.09E-04	6.21E-03
response to toxic substance	32	5	.82	6.11	+	2.13E-03	2.26E-02
actin filament polymerization	74	11	1.89	5.82	+	8.41E-06	2.59E-04
↳protein polymerization	89	11	2.27	4.84	+	3.99E-05	9.47E-04
negative regulation of cell death	54	8	1.38	5.80	+	1.47E-04	2.80E-03
↳regulation of cell death	123	10	3.14	3.18	+	1.86E-03	2.03E-02
glutathione metabolic process	35	5	.89	5.59	+	3.02E-03	2.82E-02
↳peptide metabolic process	286	35	7.31	4.79	+	2.72E-13	1.87E-10
↳cellular amide metabolic process	369	36	9.43	3.82	+	4.95E-11	1.70E-08
↳cellular modified amino acid metabolic process	67	8	1.71	4.67	+	5.51E-04	7.69E-03
muscle contraction	43	6	1.10	5.46	+	1.31E-03	1.51E-02
↳muscle system process	45	6	1.15	5.22	+	1.62E-03	1.82E-02
developmental growth	51	7	1.30	5.37	+	5.75E-04	7.92E-03
↳growth	55	7	1.41	4.98	+	8.64E-04	1.07E-02
ribosomal small subunit biogenesis	51	7	1.30	5.37	+	5.75E-04	7.86E-03
negative regulation of endopeptidase activity	82	11	2.10	5.25	+	2.01E-05	5.32E-04
↳negative regulation of peptidase activity	87	11	2.22	4.95	+	3.30E-05	8.21E-04
↳negative regulation of hydrolase activity	102	12	2.61	4.60	+	2.80E-05	7.23E-04
↳regulation of hydrolase activity	287	16	7.33	2.18	+	4.55E-03	4.03E-02
↳negative regulation of catalytic activity	169	12	4.32	2.78	+	2.01E-03	2.15E-02
↳negative regulation of molecular function	196	13	5.01	2.60	+	2.35E-03	2.38E-02
↳regulation of peptidase activity	112	12	2.86	4.19	+	6.45E-05	1.45E-03
↳regulation of proteolysis	155	14	3.96	3.53	+	9.05E-05	1.93E-03
↳regulation of protein metabolic process	581	29	14.85	1.95	+	8.87E-04	1.08E-02
↳negative regulation of proteolysis	88	11	2.25	4.89	+	3.63E-05	8.72E-04
↳negative regulation of cellular protein metabolic process	164	12	4.19	2.86	+	1.59E-03	1.81E-02
↳negative regulation of protein metabolic process	168	13	4.29	3.03	+	6.38E-04	8.44E-03
↳regulation of cellular protein metabolic process	533	26	13.62	1.91	+	2.27E-03	2.33E-02
↳regulation of endopeptidase activity	107	12	2.73	4.39	+	4.30E-05	1.01E-03
reactive oxygen species metabolic process	40	5	1.02	4.89	+	5.07E-03	4.34E-02
translational elongation	216	26	5.52	4.71	+	4.53E-10	8.50E-08
↳translation	216	26	5.52	4.71	+	4.53E-10	7.79E-08
↳cellular protein metabolic process	1711	94	43.73	2.15	+	8.18E-12	4.22E-09
↳protein metabolic process	2115	105	54.05	1.94	+	9.97E-11	2.94E-08
↳peptide biosynthetic process	219	28	5.60	5.00	+	2.70E-11	1.11E-08
↳organonitrogen compound biosynthetic process	589	40	15.05	2.66	+	7.06E-08	4.28E-06
↳amide biosynthetic process	252	28	6.44	4.35	+	5.10E-10	7.52E-08
cellular response to growth factor stimulus	90	10	2.30	4.35	+	1.98E-04	3.52E-03
↳response to growth factor	90	10	2.30	4.35	+	1.98E-04	3.55E-03
response to inorganic substance	54	6	1.38	4.35	+	3.74E-03	3.35E-02
proteasome-mediated ubiquitin-dependent protein catabolic process	194	20	4.96	4.03	+	4.59E-07	2.26E-05
↳proteasomal protein catabolic process	205	20	5.24	3.82	+	1.02E-06	4.47E-05
↳proteolysis involved in cellular protein catabolic process	331	30	8.46	3.55	+	9.91E-09	8.89E-07
↳proteolysis	693	50	17.71	2.82	+	2.22E-10	4.58E-08
↳cellular protein catabolic process	333	30	8.51	3.53	+	1.12E-08	9.67E-07
↳cellular macromolecule catabolic process	429	31	10.96	2.83	+	6.32E-07	2.97E-05
↳macromolecule catabolic process	463	34	11.83	2.87	+	1.27E-07	6.89E-06
↳cellular catabolic process	765	43	19.55	2.20	+	4.60E-06	1.58E-04
↳protein catabolic process	355	33	9.07	3.64	+	9.98E-10	1.14E-07
↳organonitrogen compound catabolic process	457	38	11.68	3.25	+	9.87E-10	1.20E-07

↳ubiquitin-dependent protein catabolic process	276	22	7.05	3.12	+	6.45E-06	2.05E-04
↳modification-dependent protein catabolic process	285	23	7.28	3.16	+	3.30E-06	1.20E-04
↳modification-dependent macromolecule catabolic process	290	23	7.41	3.10	+	4.32E-06	1.51E-04
peptidyl-lysine modification	91	8	2.33	3.44	+	3.33E-03	3.07E-02
cellular amino acid metabolic process	123	10	3.14	3.18	+	1.86E-03	2.02E-02
cell morphogenesis involved in differentiation	181	14	4.63	3.03	+	4.02E-04	6.19E-03
↳cell morphogenesis	255	19	6.52	2.92	+	6.37E-05	1.45E-03
↳cellular component morphogenesis	281	20	7.18	2.79	+	7.46E-05	1.64E-03
↳cell development	397	20	10.15	1.97	+	6.00E-03	4.97E-02
oxidation-reduction process	220	17	5.62	3.02	+	1.01E-04	2.14E-03
cell adhesion	288	19	7.36	2.58	+	2.79E-04	4.60E-03
↳biological adhesion	288	19	7.36	2.58	+	2.79E-04	4.56E-03
enzyme linked receptor protein signaling pathway	213	14	5.44	2.57	+	1.76E-03	1.94E-02
cell death	186	12	4.75	2.52	+	4.19E-03	3.74E-02
Unclassified	12808	262	327.32	.80	-	5.80E-08	3.63E-06
regulation of transcription by RNA polymerase II	745	5	19.04	.26	-	2.92E-04	4.68E-03
↳regulation of transcription, DNA-templated	1117	5	28.55	.18	-	1.14E-07	6.70E-06
↳regulation of gene expression	1368	12	34.96	.34	-	1.02E-05	2.94E-04
↳regulation of nucleic acid-templated transcription	1117	5	28.55	.18	-	1.14E-07	6.51E-06
↳regulation of RNA biosynthetic process	1117	5	28.55	.18	-	1.14E-07	6.33E-06
↳regulation of RNA metabolic process	1222	7	31.23	.22	-	2.57E-07	1.30E-05
↳regulation of nucleobase-containing compound metabolic process	1263	7	32.28	.22	-	1.32E-07	6.97E-06
↳regulation of cellular biosynthetic process	1234	11	31.54	.35	-	3.34E-05	8.22E-04
↳regulation of biosynthetic process	1240	11	31.69	.35	-	3.37E-05	8.18E-04
↳regulation of macromolecule biosynthetic process	1223	9	31.25	.29	-	4.85E-06	1.61E-04
↳regulation of cellular macromolecule biosynthetic process	1216	9	31.08	.29	-	4.75E-06	1.61E-04
transcription by RNA polymerase II	772	5	19.73	.25	-	1.51E-04	2.83E-03
↳transcription, DNA-templated	1159	5	29.62	.17	-	3.85E-08	2.74E-06
↳nucleic acid-templated transcription	1159	5	29.62	.17	-	3.85E-08	2.84E-06
↳RNA biosynthetic process	1166	5	29.80	.17	-	3.88E-08	2.67E-06
↳nucleobase-containing compound biosynthetic process	1376	11	35.16	.31	-	2.90E-06	1.07E-04
↳aromatic compound biosynthetic process	1399	11	35.75	.31	-	2.18E-06	8.32E-05
↳organic cyclic compound biosynthetic process	1433	11	36.62	.30	-	8.06E-07	3.70E-05
↳heterocycle biosynthetic process	1399	11	35.75	.31	-	2.18E-06	8.48E-05
↳RNA metabolic process	1673	18	42.75	.42	-	2.15E-05	5.62E-04
↳nucleic acid metabolic process	1911	20	48.84	.41	-	2.70E-06	1.01E-04
G protein-coupled receptor signaling pathway	682	4	17.43	.23	-	2.53E-04	4.22E-03
positive regulation of transcription, DNA-templated	421	2	10.76	.19	-	2.54E-03	2.51E-02
↳positive regulation of nucleic acid-templated transcription	421	2	10.76	.19	-	2.54E-03	2.52E-02
↳positive regulation of RNA biosynthetic process	421	2	10.76	.19	-	2.54E-03	2.50E-02
↳positive regulation of nucleobase-containing compound metabolic process	461	3	11.78	.25	-	5.98E-03	4.98E-02
cellular lipid metabolic process	317	1	8.10	.12	-	5.63E-03	4.76E-02
lymphocyte mediated immunity	232	0	5.93	< 0.01	-	4.93E-03	4.28E-02
↳leukocyte mediated immunity	235	0	6.01	< 0.01	-	5.00E-03	4.32E-02
antigen receptor-mediated signaling pathway	236	0	6.03	< 0.01	-	5.03E-03	4.32E-02
↳immune response-activating cell surface receptor signaling pathway	248	0	6.34	< 0.01	-	3.34E-03	3.06E-02
↳immune response-regulating cell surface receptor signaling pathway	250	0	6.39	< 0.01	-	3.37E-03	3.07E-02
↳immune response-regulating signaling pathway	269	0	6.87	< 0.01	-	2.33E-03	2.37E-02
↳immune response-activating signal transduction	267	0	6.82	< 0.01	-	2.29E-03	2.34E-02
negative regulation of transcription, DNA-templated	305	0	7.79	< 0.01	-	6.88E-04	8.98E-03
↳negative regulation of nucleic acid-templated transcription	305	0	7.79	< 0.01	-	6.88E-04	9.04E-03
↳negative regulation of RNA biosynthetic process	305	0	7.79	< 0.01	-	6.88E-04	8.93E-03
↳negative regulation of RNA metabolic process	316	0	8.08	< 0.01	-	7.25E-04	9.35E-03
↳negative regulation of nucleobase-containing compound metabolic process	329	0	8.41	< 0.01	-	4.79E-04	6.92E-03

↳negative regulation of cellular biosynthetic process	354	1	9.05	.11	-	2.82E-03	2.68E-02
↳negative regulation of biosynthetic process	354	1	9.05	.11	-	2.82E-03	2.69E-02
↳negative regulation of macromolecule biosynthetic process	345	1	8.82	.11	-	2.69E-03	2.62E-02
↳negative regulation of gene expression	409	0	10.45	< 0.01	-	6.95E-05	1.54E-03
↳negative regulation of cellular macromolecule biosynthetic process	345	1	8.82	.11	-	2.69E-03	2.61E-02
second-messenger-mediated signaling	228	0	5.83	< 0.01	-	4.91E-03	4.28E-02
sensory perception	242	0	6.18	< 0.01	-	5.31E-03	4.53E-02
↳nervous system process	320	1	8.18	.12	-	5.72E-03	4.80E-02

© Copyright 2020 Paul Thomas All Rights Reserved.

Analysis Summary: Please report in publication

BM-MSC secretome from ND mice

Analysis Type: PANTHER Overrepresentation Test (Released 20190711)

Annotation Version and Release Date: PANTHER version 15 Released 2020-02-14

Analyzed List: Client Text Box Input (Mus musculus) Change

Reference List: Mus musculus (all genes in database) Change

Annotation Data Set: PANTHER GO-Slim Biological Process

Test Type: (•) Fisher's Exact ( ) Binomial

Correction: (•) Calculate False Discovery Rate ( ) Use the Bonferroni correction for multiple testing ( ) No correction

Results

	Reference list	Client Text Box Input
Uniquely Mapped IDS:	22265 out of 22265	482 out of 482
Unmapped IDs:	0	3
Multiple mapping information:	0	9

Export Table XML with user input ids JSON with user input ids View: -- Please select a chart to display --

Displaying only results for FDR P < 0.05, click here to display all results

	Mus musculus (REF)	Client Text Box Input (Hierarchy NEW!)					
PANTHER GO-Slim Biological Process	#	#	expected	Fold Enrichment	+/-	raw P value	FDR
collagen fibril organization	11	6	.24	25.20	+	8.91E-07	6.57E-05
↳supramolecular fiber organization	236	28	5.11	5.48	+	2.93E-12	6.05E-09
↳cellular process	7925	222	171.56	1.29	+	3.02E-06	1.45E-04
↳extracellular matrix organization	56	14	1.21	11.55	+	1.99E-10	5.14E-08
↳extracellular structure organization	63	14	1.36	10.27	+	7.57E-10	1.56E-07
↳protein-containing complex subunit organization	481	29	10.41	2.79	+	1.71E-06	9.52E-05
glucose 6-phosphate metabolic process	9	3	.19	15.40	+	1.80E-03	2.80E-02
↳cellular metabolic process	4357	124	94.32	1.31	+	1.16E-03	1.97E-02
↳metabolic process	4857	136	105.15	1.29	+	1.22E-03	2.07E-02
glycolytic process	20	6	.43	13.86	+	1.41E-05	5.01E-04
↳generation of precursor metabolites and energy	103	10	2.23	4.48	+	1.47E-04	3.53E-03
↳nucleotide phosphorylation	20	6	.43	13.86	+	1.41E-05	4.93E-04
↳organic substance catabolic process	703	47	15.22	3.09	+	3.68E-11	1.27E-08
↳catabolic process	888	54	19.22	2.81	+	3.44E-11	1.42E-08
↳carbohydrate metabolic process	133	9	2.88	3.13	+	3.34E-03	4.62E-02
↳purine-containing compound metabolic process	152	10	3.29	3.04	+	2.45E-03	3.59E-02
↳cellular aromatic compound metabolic process	2163	27	46.83	.58	-	1.71E-03	2.70E-02
↳nitrogen compound metabolic process	4212	118	91.18	1.29	+	2.74E-03	3.88E-02
↳organic cyclic compound metabolic process	2218	27	48.02	.56	-	8.66E-04	1.57E-02
↳organonitrogen compound metabolic process	2527	107	54.71	1.96	+	2.73E-11	1.41E-08
↳heterocycle metabolic process	2151	27	46.57	.58	-	1.71E-03	2.71E-02
↳nucleobase-containing compound metabolic process	2112	26	45.72	.57	-	1.53E-03	2.49E-02
↳ATP metabolic process	56	6	1.21	4.95	+	1.98E-03	3.05E-02
Arp2/3 complex-mediated actin nucleation	18	5	.39	12.83	+	1.03E-04	2.58E-03

↳positive regulation of actin filament polymerization	34	5	.74	6.79	+	1.33E-03	2.23E-02
↳regulation of actin filament polymerization	71	9	1.54	5.86	+	4.89E-05	1.33E-03
↳regulation of protein polymerization	75	9	1.62	5.54	+	7.20E-05	1.86E-03
↳regulation of protein complex assembly	94	9	2.03	4.42	+	3.45E-04	6.84E-03
↳regulation of cellular component biogenesis	140	10	3.03	3.30	+	1.39E-03	2.29E-02
↳regulation of actin polymerization or depolymerization	73	10	1.58	6.33	+	1.01E-05	3.95E-04
↳regulation of actin filament organization	81	10	1.75	5.70	+	2.30E-05	7.21E-04
↳regulation of actin cytoskeleton organization	106	10	2.29	4.36	+	1.83E-04	4.24E-03
↳regulation of actin filament-based process	107	10	2.32	4.32	+	1.96E-04	4.45E-03
↳regulation of cytoskeleton organization	146	11	3.16	3.48	+	5.38E-04	1.03E-02
↳regulation of supramolecular fiber organization	90	10	1.95	5.13	+	5.24E-05	1.40E-03
↳regulation of actin filament length	73	10	1.58	6.33	+	1.01E-05	4.03E-04
↳regulation of cellular component size	108	10	2.34	4.28	+	2.10E-04	4.62E-03
↳regulation of anatomical structure size	120	11	2.60	4.23	+	1.12E-04	2.74E-03
↳positive regulation of supramolecular fiber organization	48	7	1.04	6.74	+	1.55E-04	3.67E-03
↳positive regulation of cytoskeleton organization	49	7	1.06	6.60	+	1.74E-04	4.07E-03
↳positive regulation of organelle organization	79	7	1.71	4.09	+	2.34E-03	3.55E-02
↳positive regulation of protein polymerization	38	5	.82	6.08	+	2.07E-03	3.17E-02
↳positive regulation of protein complex assembly	43	5	.93	5.37	+	3.38E-03	4.60E-02
↳positive regulation of cellular component biogenesis	64	6	1.39	4.33	+	3.66E-03	4.82E-02
↳actin filament organization	159	18	3.44	5.23	+	4.53E-08	5.84E-06
↳actin cytoskeleton organization	238	20	5.15	3.88	+	7.12E-07	5.44E-05
↳cytoskeleton organization	560	26	12.12	2.14	+	3.96E-04	7.72E-03
↳actin filament-based process	246	20	5.33	3.76	+	1.15E-06	7.18E-05
response to hypoxia	12	3	.26	11.55	+	3.56E-03	4.74E-02
response to oxidative stress	25	5	.54	9.24	+	3.85E-04	7.56E-03
granulocyte chemotaxis	38	7	.82	8.51	+	4.15E-05	1.17E-03
↳myeloid leukocyte migration	48	8	1.04	7.70	+	2.23E-05	7.09E-04
↳leukocyte migration	57	8	1.23	6.48	+	6.71E-05	1.75E-03
↳cell migration	255	20	5.52	3.62	+	1.92E-06	1.04E-04
↳cell motility	285	21	6.17	3.40	+	2.69E-06	1.35E-04
↳movement of cell or subcellular component	549	26	11.88	2.19	+	3.18E-04	6.37E-03
↳locomotion	356	23	7.71	2.98	+	7.12E-06	3.06E-04
↳localization of cell	285	21	6.17	3.40	+	2.69E-06	1.39E-04
↳leukocyte chemotaxis	47	8	1.02	7.86	+	1.95E-05	6.28E-04
↳cell chemotaxis	81	8	1.75	4.56	+	6.04E-04	1.12E-02
↳cellular response to chemical stimulus	641	26	13.88	1.87	+	3.62E-03	4.79E-02
nucleobase metabolic process	23	4	.50	8.03	+	2.37E-03	3.55E-02
ribosomal large subunit assembly	23	4	.50	8.03	+	2.37E-03	3.52E-02
↳ribonucleoprotein complex assembly	110	8	2.38	3.36	+	3.68E-03	4.81E-02
↳cellular protein-containing complex assembly	367	19	7.94	2.39	+	8.79E-04	1.58E-02
↳protein-containing complex assembly	394	19	8.53	2.23	+	1.55E-03	2.49E-02
chemokine-mediated signaling pathway	35	6	.76	7.92	+	2.10E-04	4.67E-03
↳cellular response to chemokine	36	6	.78	7.70	+	2.41E-04	5.02E-03
↳response to chemokine	36	6	.78	7.70	+	2.41E-04	4.97E-03
↳response to cytokine	162	10	3.51	2.85	+	3.77E-03	4.90E-02
↳response to organic substance	558	25	12.08	2.07	+	1.06E-03	1.84E-02
glucose metabolic process	26	4	.56	7.11	+	3.53E-03	4.76E-02
chaperone-mediated protein folding	26	4	.56	7.11	+	3.53E-03	4.73E-02
↳protein folding	81	17	1.75	9.69	+	2.50E-11	1.72E-08
negative regulation of endopeptidase activity	82	12	1.78	6.76	+	7.08E-07	5.62E-05
↳negative regulation of peptidase activity	87	12	1.88	6.37	+	1.25E-06	7.56E-05

↳negative regulation of hydrolase activity	102	12	2.21	5.43	+	5.59E-06	2.51E-04
↳regulation of hydrolase activity	287	17	6.21	2.74	+	2.90E-04	5.93E-03
↳negative regulation of catalytic activity	169	12	3.66	3.28	+	5.03E-04	9.71E-03
↳negative regulation of molecular function	196	13	4.24	3.06	+	5.48E-04	1.03E-02
↳regulation of peptidase activity	112	13	2.42	5.36	+	2.62E-06	1.39E-04
↳regulation of proteolysis	155	14	3.36	4.17	+	1.56E-05	5.21E-04
↳regulation of protein metabolic process	581	27	12.58	2.15	+	2.97E-04	6.02E-03
↳negative regulation of proteolysis	88	12	1.91	6.30	+	1.39E-06	8.19E-05
↳negative regulation of cellular protein metabolic process	164	13	3.55	3.66	+	1.08E-04	2.69E-03
↳negative regulation of protein metabolic process	168	14	3.64	3.85	+	3.58E-05	1.06E-03
↳regulation of cellular protein metabolic process	533	24	11.54	2.08	+	1.39E-03	2.28E-02
↳regulation of endopeptidase activity	107	13	2.32	5.61	+	1.65E-06	9.45E-05
cellular response to topologically incorrect protein	41	5	.89	5.63	+	2.80E-03	3.94E-02
↳response to topologically incorrect protein	41	5	.89	5.63	+	2.80E-03	3.91E-02
actin filament polymerization	74	9	1.60	5.62	+	6.55E-05	1.73E-03
↳protein polymerization	89	9	1.93	4.67	+	2.37E-04	5.00E-03
↳actin polymerization or depolymerization	79	11	1.71	6.43	+	3.18E-06	1.49E-04
muscle contraction	43	5	.93	5.37	+	3.38E-03	4.66E-02
carbohydrate biosynthetic process	43	5	.93	5.37	+	3.38E-03	4.63E-02
negative regulation of cell death	54	6	1.17	5.13	+	1.67E-03	2.67E-02
response to endoplasmic reticulum stress	65	7	1.41	4.97	+	8.25E-04	1.51E-02
proteasome-mediated ubiquitin-dependent protein catabolic process	194	16	4.20	3.81	+	1.14E-05	4.35E-04
↳proteasomal protein catabolic process	205	17	4.44	3.83	+	5.69E-06	2.50E-04
↳proteolysis involved in cellular protein catabolic process	331	28	7.17	3.91	+	3.74E-09	5.94E-07
↳proteolysis	693	47	15.00	3.13	+	2.33E-11	2.40E-08
↳protein metabolic process	2115	89	45.79	1.94	+	3.02E-09	5.20E-07
↳cellular protein catabolic process	333	28	7.21	3.88	+	4.23E-09	6.23E-07
↳cellular macromolecule catabolic process	429	29	9.29	3.12	+	1.90E-07	2.18E-05
↳macromolecule catabolic process	463	32	10.02	3.19	+	2.70E-08	3.72E-06
↳cellular catabolic process	765	42	16.56	2.54	+	1.14E-07	1.39E-05
↳protein catabolic process	355	31	7.69	4.03	+	2.61E-10	5.99E-08
↳organonitrogen compound catabolic process	457	36	9.89	3.64	+	1.32E-10	3.90E-08
↳cellular protein metabolic process	1711	77	37.04	2.08	+	1.98E-09	3.71E-07
↳ubiquitin-dependent protein catabolic process	276	17	5.97	2.85	+	1.88E-04	4.32E-03
↳modification-dependent protein catabolic process	285	18	6.17	2.92	+	9.14E-05	2.33E-03
↳modification-dependent macromolecule catabolic process	290	18	6.28	2.87	+	1.12E-04	2.73E-03
animal organ morphogenesis	104	8	2.25	3.55	+	2.67E-03	3.85E-02
translational elongation	216	16	4.68	3.42	+	3.88E-05	1.13E-03
↳translation	216	16	4.68	3.42	+	3.88E-05	1.11E-03
↳peptide biosynthetic process	219	16	4.74	3.37	+	4.52E-05	1.26E-03
↳amide biosynthetic process	252	16	5.46	2.93	+	2.09E-04	4.70E-03
↳cellular amide metabolic process	369	23	7.99	2.88	+	1.23E-05	4.53E-04
↳peptide metabolic process	286	23	6.19	3.71	+	2.18E-07	2.36E-05
oxidation-reduction process	220	16	4.76	3.36	+	4.76E-05	1.31E-03
tissue development	179	11	3.88	2.84	+	2.52E-03	3.66E-02
enzyme linked receptor protein signaling pathway	213	13	4.61	2.82	+	1.13E-03	1.94E-02
Unclassified	12808	231	277.27	.83	-	3.27E-05	9.93E-04
organic substance transport	801	6	17.34	.35	-	2.68E-03	3.84E-02
↳transport	1822	20	39.44	.51	-	6.91E-04	1.27E-02
↳establishment of localization	1847	20	39.98	.50	-	5.41E-04	1.02E-02
G protein-coupled receptor signaling pathway	682	4	14.76	.27	-	1.77E-03	2.77E-02
transcription by RNA polymerase II	772	2	16.71	.12	-	1.79E-05	5.87E-04
↳transcription, DNA-templated	1159	4	25.09	.16	-	3.24E-07	3.19E-05

↳nucleic acid-templated transcription	1159	4	25.09	.16	-	3.24E-07	3.35E-05
↳RNA biosynthetic process	1166	4	25.24	.16	-	3.30E-07	3.09E-05
↳nucleobase-containing compound biosynthetic process	1376	7	29.79	.23	-	9.68E-07	6.89E-05
↳aromatic compound biosynthetic process	1399	7	30.29	.23	-	4.71E-07	3.89E-05
↳organic cyclic compound biosynthetic process	1433	7	31.02	.23	-	3.44E-07	3.09E-05
↳heterocycle biosynthetic process	1399	7	30.29	.23	-	4.71E-07	4.05E-05
↳RNA metabolic process	1673	13	36.22	.36	-	9.44E-06	3.98E-04
↳nucleic acid metabolic process	1911	15	41.37	.36	-	2.83E-06	1.39E-04
negative regulation of gene expression	409	1	8.85	.11	-	2.72E-03	3.88E-02
↳regulation of gene expression	1368	9	29.61	.30	-	1.16E-05	4.36E-04
cellular lipid metabolic process	317	0	6.86	< 0.01	-	2.36E-03	3.56E-02
positive regulation of transcription by RNA polymerase II	345	0	7.47	< 0.01	-	1.03E-03	1.80E-02
↳regulation of transcription by RNA polymerase II	745	2	16.13	.12	-	2.48E-05	7.65E-04
↳regulation of transcription, DNA-templated	1117	4	24.18	.17	-	1.02E-06	7.01E-05
↳regulation of nucleic acid-templated transcription	1117	4	24.18	.17	-	1.02E-06	6.78E-05
↳regulation of RNA biosynthetic process	1117	4	24.18	.17	-	1.02E-06	6.57E-05
↳regulation of RNA metabolic process	1222	7	26.45	.26	-	1.41E-05	4.86E-04
↳regulation of nucleobase-containing compound metabolic process	1263	7	27.34	.26	-	5.05E-06	2.31E-04
↳regulation of cellular biosynthetic process	1234	7	26.71	.26	-	9.87E-06	4.08E-04
↳regulation of biosynthetic process	1240	7	26.84	.26	-	1.01E-05	4.09E-04
↳regulation of macromolecule biosynthetic process	1223	7	26.48	.26	-	1.42E-05	4.81E-04
↳regulation of cellular macromolecule biosynthetic process	1216	7	26.32	.27	-	1.38E-05	4.99E-04
↳positive regulation of transcription, DNA-templated	421	0	9.11	< 0.01	-	2.17E-04	4.67E-03
↳positive regulation of nucleic acid-templated transcription	421	0	9.11	< 0.01	-	2.17E-04	4.72E-03
↳positive regulation of RNA biosynthetic process	421	0	9.11	< 0.01	-	2.17E-04	4.62E-03
↳positive regulation of cellular biosynthetic process	458	1	9.91	.10	-	8.96E-04	1.60E-02
↳positive regulation of biosynthetic process	461	1	9.98	.10	-	8.98E-04	1.58E-02
↳positive regulation of macromolecule biosynthetic process	451	1	9.76	.10	-	1.34E-03	2.23E-02
nervous system process	320	0	6.93	< 0.01	-	2.43E-03	3.58E-02

© Copyright 2020 Paul Thomas All Rights Reserved.

Analysis Summary: Please report in publication vWAT-MSC secretome from ND mice

Analysis Type: PANTHER Overrepresentation Test (Released 20190711)

Annotation Version and Release Date: PANTHER version 15 Released 2020-02-14

Analyzed List: Client Text Box Input (Mus musculus) Change

Reference List: Mus musculus (all genes in database) Change

Annotation Data Set: PANTHER Pathways

Test Type: (•) Fisher's Exact ( ) Binomial

Correction: (•) Calculate False Discovery Rate ( ) Use the Bonferroni correction for multiple testing ( ) No correction

Results

	Reference list	Client Text Box Input
Uniquely Mapped IDS:	22265 out of 22265	361 out of 363
Unmapped IDs:	0	0
Multiple mapping information:	0	5

Export Table XML with user input ids JSON with user input ids View: -- Please select a chart to display --

Displaying only results for FDR P < 0.05, click here to display all results

	Mus musculus (REF)	Client Text Box Input					
PANTHER Pathways	#	#	expected	Fold Enrichment	+/-	raw P value	FDR
Asparagine and aspartate biosynthesis	4	2	.07	30.67	+	3.69E-03	4.08E-02
Pentose phosphate pathway	9	4	.15	27.26	+	4.15E-05	9.85E-04
ATP synthesis	7	3	.11	26.29	+	4.52E-04	9.38E-03
Glycolysis	26	8	.42	18.87	+	5.12E-08	1.70E-06
Cytoskeletal regulation by Rho GTPase	79	15	1.29	11.65	+	2.65E-11	1.47E-09
Parkinson disease	95	18	1.55	11.62	+	2.75E-13	2.28E-11
De novo purine biosynthesis	29	4	.47	8.46	+	1.85E-03	2.55E-02
Integrin signalling pathway	190	21	3.10	6.78	+	3.25E-11	1.35E-09
Blood coagulation	51	5	.83	6.01	+	2.02E-03	2.58E-02
Huntington disease	150	12	2.45	4.91	+	1.26E-05	3.49E-04
FGF signaling pathway	120	8	1.96	4.09	+	1.09E-03	2.02E-02
Alzheimer disease-presenilin pathway	125	8	2.04	3.93	+	1.40E-03	2.32E-02
EGF receptor signaling pathway	136	8	2.22	3.61	+	2.31E-03	2.74E-02
Inflammation mediated by chemokine and cytokine signaling pathway	258	12	4.21	2.85	+	1.48E-03	2.23E-02
Unclassified	19669	256	320.68	.80	-	1.51E-19	2.51E-17

© Copyright 2020 Paul Thomas All Rights Reserved.

Analysis Summary: Please report in publication sWAT-MSC secretome from ND mice

Analysis Type: PANTHER Overrepresentation Test (Released 20190711)

Annotation Version and Release Date: PANTHER version 15 Released 2020-02-14

Analyzed List: Client Text Box Input (Mus musculus) Change

Reference List: Mus musculus (all genes in database) Change

Annotation Data Set: PANTHER Pathways

Test Type: (•) Fisher's Exact ( ) Binomial

Correction: (•) Calculate False Discovery Rate ( ) Use the Bonferroni correction for multiple testing ( ) No correction

Results

	Reference list	Client Text Box Input
Uniquely Mapped IDS:	22265 out of 22265	572 out of 569
Unmapped IDs:	0	1
Multiple mapping information:	0	15

Export Table XML with user input ids JSON with user input ids View: -- Please select a chart to display --

Displaying only results for FDR P < 0.05, click here to display all results

	Mus musculus (REF)	Client Text Box Input					
PANTHER Pathways	#	#	expected	Fold Enrichment	+/-	raw P value	FDR
Pentose phosphate pathway	9	4	.23	17.39	+	2.28E-04	4.21E-03
Purine metabolism	8	3	.20	14.67	+	2.19E-03	2.79E-02
Adenine and hypoxanthine salvage pathway	8	3	.20	14.67	+	2.19E-03	2.59E-02
Glycolysis	26	8	.66	12.04	+	1.45E-06	4.82E-05
Parkinson disease	95	21	2.43	8.65	+	1.05E-12	5.80E-11
Cytoskeletal regulation by Rho GTPase	79	16	2.02	7.93	+	1.61E-09	6.67E-08
Axon guidance mediated by semaphorins	23	4	.59	6.81	+	4.25E-03	4.41E-02
Integrin signalling pathway	190	33	4.86	6.80	+	1.78E-16	1.47E-14
De novo purine biosynthesis	29	5	.74	6.75	+	1.44E-03	2.00E-02
Axon guidance mediated by Slit/Robo	24	4	.61	6.52	+	4.86E-03	4.74E-02
Blood coagulation	51	8	1.30	6.14	+	1.03E-04	2.13E-03
Huntington disease	150	16	3.83	4.17	+	4.30E-06	1.19E-04
FGF signaling pathway	120	11	3.07	3.59	+	4.48E-04	7.43E-03
CCKR signaling map	159	12	4.06	2.95	+	1.24E-03	1.88E-02
Inflammation mediated by chemokine and cytokine signaling pathway	258	19	6.59	2.88	+	7.37E-05	1.75E-03
EGF receptor signaling pathway	136	10	3.48	2.88	+	3.67E-03	4.07E-02
Unclassified	19669	411	502.66	.82	-	1.02E-24	1.70E-22

© Copyright 2020 Paul Thomas All Rights Reserved.

PANTHER 15.0 released!

Analysis Summary: Please report in publication

BM-MSC secretome from ND mice

Analysis Type: PANTHER Overrepresentation Test (Released 20190711)

Annotation Version and Release Date: PANTHER version 15 Released 2020-02-14

Analyzed List: Client Text Box Input (Mus musculus) Change

Reference List: Mus musculus (all genes in database) Change

Annotation Data Set: PANTHER Pathways

Test Type: (•) Fisher's Exact ( ) Binomial

Correction: (•) Calculate False Discovery Rate ( ) Use the Bonferroni correction for multiple testing ( ) No correction

Results

	Reference list	Client Text Box Input
Uniquely Mapped IDS:	22265 out of 22265	482 out of 482
Unmapped IDs:	0	3
Multiple mapping information:	0	9

Export Table XML with user input ids JSON with user input ids View: -- Please select a chart to display --

Displaying only results for FDR P < 0.05, click here to display all results

	Mus musculus (REF)	Client Text Box Input					
PANTHER Pathways	#	#	expected	Fold Enrichment	+/-	raw P value	FDR
Pentose phosphate pathway	9	4	.19	20.53	+	1.22E-04	2.54E-03
Glycolysis	26	9	.56	15.99	+	3.46E-08	1.44E-06
Plasminogen activating cascade	17	5	.37	13.59	+	8.18E-05	1.94E-03
Pyruvate metabolism	12	3	.26	11.55	+	3.56E-03	4.54E-02
Blood coagulation	51	10	1.10	9.06	+	5.70E-07	1.58E-05
Parkinson disease	95	17	2.06	8.27	+	2.23E-10	1.24E-08
De novo purine biosynthesis	29	5	.63	7.96	+	7.01E-04	1.29E-02
Cytoskeletal regulation by Rho GTPase	79	12	1.71	7.02	+	4.95E-07	1.64E-05
Integrin signalling pathway	190	27	4.11	6.56	+	1.56E-13	1.29E-11
Alzheimer disease-presenilin pathway	125	9	2.71	3.33	+	2.26E-03	3.12E-02
Huntington disease	150	10	3.25	3.08	+	2.24E-03	3.38E-02
Inflammation mediated by chemokine and cytokine signaling pathway	258	14	5.59	2.51	+	2.12E-03	3.51E-02
Unclassified	19669	346	425.80	.81	-	2.74E-22	4.54E-20

© Copyright 2020 Paul Thomas All Rights Reserved.

PANTHER 15.0 released!

Analysis Summary: Please report in publication

vWAT-MSC secretome from HFD mice

Analysis Type: PANTHER Overrepresentation Test (Released 20190711)

Annotation Version and Release Date: PANTHER version 15 Released 2020-02-14

Analyzed List: Client Text Box Input (Mus musculus) Change

Reference List: Mus musculus (all genes in database) Change

Annotation Data Set: PANTHER GO-Slim Cellular Component

Test Type: (•) Fisher's Exact ( ) Binomial

Correction: (•) Calculate False Discovery Rate ( ) Use the Bonferroni correction for multiple testing ( ) No correction

Results

	Reference list	Client Text Box Input
Uniquely Mapped IDS:	22265 out of 22265	377 out of 378
Unmapped IDs:	0	3
Multiple mapping information:	0	10

Export Table XML with user input ids JSON with user input ids View: -- Please select a chart to display --

Displaying only results for FDR P < 0.05, click here to display all results

	Mus musculus (REF)	Client Text Box Input (Hierarchy NEW!)					
PANTHER GO-Slim Cellular Component	#	#	expected	Fold Enrichment	+/-	raw P value	FDR
proteasome core complex, alpha-subunit complex	8	7	.14	51.54	+	1.96E-09	7.84E-08
↳proteasome complex	39	14	.66	21.14	+	1.37E-13	1.78E-11
↳endopeptidase complex	39	14	.66	21.14	+	1.37E-13	2.37E-11
↳peptidase complex	55	14	.93	14.99	+	6.92E-12	3.60E-10
↳intracellular part	6691	144	113.60	1.27	+	1.02E-03	1.33E-02
↳intracellular	6721	145	114.10	1.27	+	8.46E-04	1.16E-02
Arp2/3 protein complex	9	4	.15	26.18	+	4.85E-05	7.88E-04
↳cytoskeletal part	486	22	8.25	2.67	+	5.04E-05	7.94E-04
↳cytoskeleton	586	22	9.95	2.21	+	9.84E-04	1.31E-02
↳actin cytoskeleton	160	19	2.72	6.99	+	1.84E-10	8.72E-09
collagen trimer	17	7	.29	24.25	+	9.26E-08	2.68E-06
collagen-containing extracellular matrix	66	15	1.12	13.39	+	4.95E-12	2.86E-10
↳extracellular matrix	177	22	3.00	7.32	+	2.91E-12	1.89E-10
	1253	71	21.27	3.34	+	1.42E-18	3.68E-16

↳extracellular region part							
↳extracellular region	1253	71	21.27	3.34	+	1.42E-18	7.36E-16
actin filament	70	13	1.19	10.94	+	1.17E-09	5.08E-08
↳polymeric cytoskeletal fiber	246	16	4.18	3.83	+	9.46E-06	2.14E-04
↳supramolecular fiber	298	21	5.06	4.15	+	1.11E-07	3.04E-06
↳supramolecular polymer	301	22	5.11	4.31	+	3.01E-08	1.12E-06
↳supramolecular complex	301	22	5.11	4.31	+	3.01E-08	1.04E-06
cytosolic small ribosomal subunit	40	7	.68	10.31	+	1.21E-05	2.33E-04
↳cytosolic part	162	14	2.75	5.09	+	1.65E-06	4.08E-05
↳cytoplasmic part	2902	80	49.27	1.62	+	1.49E-05	2.77E-04
↳cytoplasm	4183	132	71.02	1.86	+	2.39E-13	2.49E-11
↳cytosol	753	46	12.78	3.60	+	2.63E-13	2.28E-11
↳cytosolic ribosome	104	12	1.77	6.80	+	5.69E-07	1.48E-05
↳ribosome	151	12	2.56	4.68	+	1.99E-05	3.57E-04
↳small ribosomal subunit	57	7	.97	7.23	+	9.37E-05	1.39E-03
↳ribosomal subunit	143	12	2.43	4.94	+	1.20E-05	2.40E-04
cortical actin cytoskeleton	38	5	.65	7.75	+	7.22E-04	1.01E-02
↳cortical cytoskeleton	42	5	.71	7.01	+	1.09E-03	1.38E-02
↳cell cortex part	91	7	1.54	4.53	+	1.29E-03	1.59E-02
extracellular space	1169	58	19.85	2.92	+	6.88E-13	5.11E-11
Unclassified	12713	162	215.83	.75	-	4.26E-08	1.30E-06
integral component of plasma membrane	770	1	13.07	.08	-	4.42E-05	7.67E-04
↳integral component of membrane	1114	3	18.91	.16	-	1.14E-05	2.38E-04
↳intrinsic component of membrane	1141	3	19.37	.15	-	8.00E-06	1.89E-04
↳membrane part	2301	15	39.06	.38	-	1.03E-05	2.23E-04
↳membrane	3585	37	60.86	.61	-	6.46E-04	9.33E-03
↳intrinsic component of plasma membrane	777	1	13.19	.08	-	4.46E-05	7.48E-04
↳plasma membrane part	1622	9	27.54	.33	-	5.04E-05	7.71E-04

© Copyright 2020 Paul Thomas All Rights Reserved.

Analysis Summary: Please report in publication ? sWAT-MSC secretome from HFD mice

Analysis Type: PANTHER Overrepresentation Test (Released 20190711)

Annotation Version and Release Date: PANTHER version 15 Released 2020-02-14

Analyzed List: Client Text Box Input (Mus musculus) Change

Reference List: Mus musculus (all genes in database) Change

Annotation Data Set: PANTHER GO-Slim Cellular Component ?

Test Type: (•) Fisher's Exact ( ) Binomial

Correction: (•) Calculate False Discovery Rate ( ) Use the Bonferroni correction for multiple testing ? ( ) No correction

Results ?

	Reference list	Client Text Box Input
Uniquely Mapped IDS:	22265 out of 22265	506 out of 505
Unmapped IDs:	0	4
Multiple mapping information:	0	8

Export Table | XML with user input ids | JSON with user input ids View: -- Please select a chart to display --

Displaying only results for FDR P < 0.05, click here to display all results

	Mus musculus (REF)	Client Text Box Input (Hierarchy NEW! ?)					
PANTHER GO-Slim Cellular Component	#	#	expected	Fold Enrichment	+/-	raw P value	FDR
proteasome core complex, alpha-subunit complex	8	7	.18	38.58	+	1.40E-08	5.19E-07
↳proteasome complex	39	15	.88	16.96	+	4.92E-13	3.20E-11
↳endopeptidase complex	39	15	.88	16.96	+	4.92E-13	3.65E-11
↳peptidase complex	55	15	1.25	12.02	+	2.96E-11	1.54E-09
↳intracellular part	6691	185	151.76	1.22	+	1.69E-03	1.80E-02
↳intracellular	6721	186	152.44	1.22	+	1.70E-03	1.77E-02
Arp2/3 protein complex	9	5	.20	24.49	+	8.93E-06	1.86E-04
↳cytoskeletal part	486	29	11.02	2.63	+	5.04E-06	1.19E-04
↳cytoskeleton	586	30	13.29	2.26	+	6.75E-05	9.48E-04
↳intracellular non-membrane-bounded organelle	1344	48	30.48	1.57	+	2.50E-03	2.45E-02
↳non-membrane-bounded organelle	1344	48	30.48	1.57	+	2.50E-03	2.40E-02
↳actin cytoskeleton	160	23	3.63	6.34	+	2.05E-11	1.18E-09
collagen trimer	17	6	.39	15.56	+	8.46E-06	1.83E-04
actin cortical patch	15	5	.34	14.70	+	6.19E-05	8.94E-04
↳cortical actin cytoskeleton	38	8	.86	9.28	+	6.89E-06	1.56E-04
↳cortical cytoskeleton							

	42	8	.95	8.40	+	1.31E-05	2.44E-04
↳cell cortex part	91	9	2.06	4.36	+	3.85E-04	4.66E-03
↳cell cortex	121	10	2.74	3.64	+	6.94E-04	8.02E-03
↳cytoplasmic part	2902	110	65.82	1.67	+	8.46E-08	2.75E-06
↳cytoplasm	4183	170	94.88	1.79	+	4.78E-15	4.97E-13
chaperonin-containing T-complex	10	3	.23	13.23	+	2.63E-03	2.48E-02
↳cytosolic part	162	18	3.67	4.90	+	1.16E-07	3.18E-06
↳cytosol	753	50	17.08	2.93	+	5.47E-11	2.59E-09
basement membrane	27	6	.61	9.80	+	7.69E-05	1.05E-03
↳collagen-containing extracellular matrix	66	20	1.50	13.36	+	2.70E-15	3.51E-13
↳extracellular matrix	177	34	4.01	8.47	+	1.11E-19	1.93E-17
↳extracellular region part	1253	98	28.42	3.45	+	7.16E-26	1.86E-23
↳extracellular region	1253	98	28.42	3.45	+	7.16E-26	3.72E-23
actomyosin	18	4	.41	9.80	+	1.27E-03	1.38E-02
actin filament	70	13	1.59	8.19	+	3.46E-08	1.20E-06
↳polymeric cytoskeletal fiber	246	18	5.58	3.23	+	2.76E-05	4.34E-04
↳supramolecular fiber	298	24	6.76	3.55	+	2.66E-07	6.92E-06
↳supramolecular polymer	301	25	6.83	3.66	+	8.69E-08	2.66E-06
↳supramolecular complex	301	25	6.83	3.66	+	8.69E-08	2.51E-06
focal adhesion	38	6	.86	6.96	+	3.99E-04	4.71E-03
↳adherens junction	93	8	2.11	3.79	+	1.85E-03	1.88E-02
↳anchoring junction	102	8	2.31	3.46	+	3.15E-03	2.88E-02
↳cell junction	121	9	2.74	3.28	+	2.49E-03	2.49E-02
cytosolic large ribosomal subunit	64	9	1.45	6.20	+	3.33E-05	5.09E-04
↳cytosolic ribosome	104	14	2.36	5.94	+	3.69E-07	9.14E-06
↳ribosome	151	14	3.42	4.09	+	1.98E-05	3.43E-04
↳large ribosomal subunit	86	9	1.95	4.61	+	2.62E-04	3.25E-03
↳ribosomal subunit	143	14	3.24	4.32	+	1.12E-05	2.17E-04
cytosolic small ribosomal subunit	40	5	.91	5.51	+	3.09E-03	2.87E-02
extracellular space	1169	74	26.51	2.79	+	7.64E-15	6.63E-13
Unclassified	12713	223	288.35	.77	-	8.83E-09	3.83E-07
nuclear lumen	868	5	19.69	.25	-	1.49E-04	1.98E-03
↳nuclear part	1243	8	28.19	.28	-	1.10E-05	2.20E-04
↳intracellular organelle lumen	951	9	21.57	.42	-	3.40E-03	3.00E-02
↳organelle lumen	951	9	21.57	.42	-	3.40E-03	2.95E-02
↳membrane-enclosed lumen	951	9	21.57	.42	-	3.40E-03	3.05E-02
integral component of plasma membrane	770	4	17.46	.23	-	2.54E-04	3.22E-03
↳integral component of membrane	1114	7	25.27	.28	-	2.65E-05	4.30E-04
↳intrinsic component of membrane	1141	7	25.88	.27	-	1.93E-05	3.46E-04
↳membrane part	2301	25	52.19	.48	-	2.08E-05	3.48E-04
↳membrane	3585	55	81.31	.68	-	1.12E-03	1.23E-02
↳intrinsic component of plasma membrane	777	4	17.62	.23	-	1.78E-04	2.32E-03
↳plasma membrane part	1622	15	36.79	.41	-	5.38E-05	8.00E-04
transferase complex	444	1	10.07	.10	-	9.06E-04	1.02E-02

© Copyright 2020 Paul Thomas All Rights Reserved.

Analysis Summary: Please report in publication BM-MSC secretome from HFD mice

Analysis Type: PANTHER Overrepresentation Test (Released 20190711)

Annotation Version and Release Date: PANTHER version 15 Released 2020-02-14

Analyzed List: Client Text Box Input (Mus musculus) Change

Reference List: Mus musculus (all genes in database) Change

Annotation Data Set: PANTHER GO-Slim Cellular Component

Test Type: Fisher's Exact Binomial

Correction: Calculate False Discovery Rate Use the Bonferroni correction for multiple testing No correction

Results

	Reference list	Client Text Box Input
Uniquely Mapped IDS:	22265 out of 22265	438 out of 438
Unmapped IDs:	0	3
Multiple mapping information:	0	9

Export Table XML with user input ids JSON with user input ids View: -- Please select a chart to display --

Displaying only results for FDR P < 0.05, click here to display all results

	Mus musculus (REF)	Client Text Box Input (Hierarchy NEW!)					
PANTHER GO-Slim Cellular Component	#	#	expected	Fold Enrichment	+/-	raw P value	FDR
proteasome core complex, alpha-subunit complex	8	7	.16	44.48	+	5.34E-09	4.63E-07
↳proteasome complex	39	10	.77	13.03	+	2.71E-08	1.56E-06
↳endopeptidase complex	39	10	.77	13.03	+	2.71E-08	1.76E-06
↳peptidase complex	55	10	1.08	9.24	+	4.48E-07	1.79E-05
Arp2/3 protein complex	9	5	.18	28.24	+	4.53E-06	1.24E-04
↳cytoskeletal part	486	20	9.56	2.09	+	2.64E-03	3.27E-02
↳actin cytoskeleton	160	18	3.15	5.72	+	1.18E-08	8.79E-07
collagen trimer	17	7	.33	20.93	+	2.47E-07	1.17E-05
basement membrane	27	6	.53	11.30	+	3.55E-05	6.83E-04
↳collagen-containing extracellular matrix	66	22	1.30	16.94	+	1.06E-18	1.10E-16
↳extracellular matrix	177	35	3.48	10.05	+	1.47E-22	1.91E-20
↳extracellular region part	1253	107	24.65	4.34	+	4.63E-37	1.20E-34
↳extracellular region	1253	107	24.65	4.34	+	4.63E-37	2.41E-34
actin filament	70	11	1.38	7.99	+	4.38E-07	1.90E-05

↳polymeric cytoskeletal fiber	246	13	4.84	2.69	+	1.66E-03	2.16E-02
↳supramolecular fiber	298	18	5.86	3.07	+	4.74E-05	8.81E-04
↳supramolecular polymer	301	19	5.92	3.21	+	1.66E-05	3.92E-04
↳supramolecular complex	301	19	5.92	3.21	+	1.66E-05	3.75E-04
cortical actin cytoskeleton	38	5	.75	6.69	+	1.38E-03	1.83E-02
↳cortical cytoskeleton	42	5	.83	6.05	+	2.06E-03	2.61E-02
↳cytoplasm	4183	118	82.29	1.43	+	3.47E-05	6.94E-04
lysosome	114	11	2.24	4.90	+	3.14E-05	6.80E-04
↳lytic vacuole	115	11	2.26	4.86	+	3.38E-05	7.03E-04
↳intracellular membrane-bounded organelle	4662	60	91.71	.65	-	1.35E-04	2.35E-03
↳membrane-bounded organelle	4721	62	92.87	.67	-	2.34E-04	3.80E-03
extracellular space	1169	85	23.00	3.70	+	1.22E-24	2.11E-22
cytosol	753	36	14.81	2.43	+	2.01E-06	7.47E-05
Unclassified	12713	201	250.09	.80	-	3.42E-06	1.11E-04
nuclear lumen	868	4	17.08	.23	-	3.53E-04	5.41E-03
↳nuclear part	1243	5	24.45	.20	-	3.57E-06	1.09E-04
↳nucleus	2974	29	58.50	.50	-	1.21E-05	3.01E-04
↳intracellular organelle lumen	951	6	18.71	.32	-	1.07E-03	1.50E-02
↳organelle lumen	951	6	18.71	.32	-	1.07E-03	1.46E-02
↳membrane-enclosed lumen	951	6	18.71	.32	-	1.07E-03	1.54E-02
integral component of plasma membrane	770	3	15.15	.20	-	4.24E-04	6.30E-03
↳integral component of membrane	1114	4	21.91	.18	-	5.57E-06	1.45E-04
↳intrinsic component of membrane	1141	4	22.45	.18	-	4.01E-06	1.16E-04
↳membrane part	2301	15	45.27	.33	-	1.53E-07	7.95E-06
↳membrane	3585	43	70.52	.61	-	2.15E-04	3.61E-03
↳intrinsic component of plasma membrane	777	3	15.29	.20	-	2.85E-04	4.49E-03
↳plasma membrane part	1622	12	31.91	.38	-	7.48E-05	1.34E-03
↳plasma membrane	3243	43	63.80	.67	-	3.95E-03	4.56E-02
cytoplasmic vesicle	448	1	8.81	.11	-	2.72E-03	3.21E-02
↳intracellular vesicle	448	1	8.81	.11	-	2.72E-03	3.28E-02

© Copyright 2020 Paul Thomas All Rights Reserved.

Analysis Summary: Please report in publication vWAT-MSC secretome from HFD mice

Analysis Type: PANTHER Overrepresentation Test (Released 20190711)

Annotation Version and Release Date: PANTHER version 15 Released 2020-02-14

Analyzed List: Client Text Box Input (Mus musculus) Change

Reference List: Mus musculus (all genes in database) Change

Annotation Data Set: PANTHER Protein Class

Test Type: (•) Fisher's Exact ( ) Binomial

Correction: (•) Calculate False Discovery Rate ( ) Use the Bonferroni correction for multiple testing ( ) No correction

Results

	Reference list	Client Text Box Input
Uniquely Mapped IDS:	22265 out of 22265	377 out of 378
Unmapped IDs:	0	3
Multiple mapping information:	0	10

Export Table XML with user input ids JSON with user input ids View: -- Please select a chart to display --

Displaying only results for FDR P < 0.05, click here to display all results

	Mus musculus (REF)	Client Text Box Input (Hierarchy NEW!)					
PANTHER Protein Class	#	#	expected	Fold Enrichment	+/-	raw P value	FDR
Hsp90 family chaperone	4	3	.07	44.18	+	1.54E-04	1.87E-03
↳chaperone	74	7	1.26	5.57	+	4.12E-04	4.01E-03
extracellular matrix structural protein	73	16	1.24	12.91	+	1.55E-12	6.03E-11
↳extracellular matrix protein	161	22	2.73	8.05	+	5.26E-13	2.56E-11
translation elongation factor	15	3	.25	11.78	+	3.13E-03	2.10E-02
↳translational protein	288	16	4.89	3.27	+	5.79E-05	8.06E-04
non-motor actin binding protein	93	18	1.58	11.40	+	3.92E-13	2.55E-11
↳actin or actin-binding cytoskeletal protein	236	35	4.01	8.74	+	5.13E-21	1.00E-18
↳cytoskeletal protein	492	38	8.35	4.55	+	4.67E-14	4.55E-12
actin binding motor protein	27	4	.46	8.73	+	1.68E-03	1.37E-02
actin and actin related protein	30	4	.51	7.85	+	2.39E-03	1.72E-02
calmodulin-related	62	8	1.05	7.60	+	2.14E-05	3.22E-04
↳calcium-binding protein	79	13	1.34	9.69	+	4.33E-09	1.05E-07

protease inhibitor	157	17	2.67	6.38	+	6.03E-09	1.31E-07
↳protein-binding activity modulator	632	22	10.73	2.05	+	1.76E-03	1.37E-02
reductase	51	5	.87	5.77	+	2.40E-03	1.67E-02
↳oxidoreductase	418	23	7.10	3.24	+	1.68E-06	3.28E-05
↳metabolite interconversion enzyme	1428	43	24.24	1.77	+	3.17E-04	3.25E-03
ribosomal protein	159	11	2.70	4.07	+	1.41E-04	1.83E-03
dehydrogenase	119	7	2.02	3.46	+	5.29E-03	3.33E-02
protease	597	22	10.14	2.17	+	1.09E-03	9.67E-03
Unclassified	12347	144	209.62	.69	-	2.30E-11	7.49E-10
immunoglobulin	389	0	6.60	< 0.01	-	2.26E-03	1.70E-02
↳defense/immunity protein	600	2	10.19	.20	-	5.23E-03	3.40E-02
C2H2 zinc finger transcription factor	424	0	7.20	< 0.01	-	1.59E-03	1.35E-02
↳zinc finger transcription factor	502	0	8.52	< 0.01	-	3.15E-04	3.41E-03
↳DNA-binding transcription factor	970	1	16.47	.06	-	2.12E-06	3.75E-05
↳gene-specific transcriptional regulator	1054	2	17.89	.11	-	5.64E-06	9.17E-05
G-protein coupled receptor	568	0	9.64	< 0.01	-	1.56E-04	1.79E-03
↳transmembrane signal receptor	712	2	12.09	.17	-	8.92E-04	8.29E-03

© Copyright 2020 Paul Thomas All Rights Reserved.

Analysis Summary: Please report in publication sWAT-MSC secretome from HFD mice

Analysis Type: PANTHER Overrepresentation Test (Released 20190711)

Annotation Version and Release Date: PANTHER version 15 Released 2020-02-14

Analyzed List: Client Text Box Input (Mus musculus) Change

Reference List: Mus musculus (all genes in database) Change

Annotation Data Set: PANTHER Protein Class

Test Type: Fisher's Exact Binomial

Correction: Calculate False Discovery Rate Use the Bonferroni correction for multiple testing No correction

Results

	Reference list	Client Text Box Input
Uniquely Mapped IDS:	22265 out of 22265	506 out of 505
Unmapped IDs:	0	4
Multiple mapping information:	0	8

Export Table XML with user input ids JSON with user input ids View: -- Please select a chart to display --

Displaying only results for FDR P < 0.05, click here to display all results

	Mus musculus (REF)	Client Text Box Input (Hierarchy NEW!)					
PANTHER Protein Class	#	#	expected	Fold Enrichment	+/-	raw P value	FDR
Hsp90 family chaperone	4	3	.09	33.07	+	3.55E-04	2.47E-03
↳chaperone	74	13	1.68	7.75	+	6.18E-08	1.00E-06
chaperonin	13	5	.29	16.96	+	3.55E-05	3.30E-04
extracellular matrix structural protein	73	16	1.66	9.66	+	1.08E-10	3.00E-09
↳extracellular matrix protein	161	27	3.65	7.39	+	1.32E-14	6.42E-13
calmodulin-related	62	12	1.41	8.53	+	7.84E-08	1.18E-06
↳calcium-binding protein	79	18	1.79	10.05	+	4.16E-12	1.35E-10
non-motor actin binding protein	93	17	2.11	8.06	+	3.36E-10	8.19E-09
↳actin or actin-binding cytoskeletal protein	236	37	5.35	6.91	+	1.08E-18	2.10E-16
↳cytoskeletal protein	492	42	11.16	3.76	+	1.40E-12	5.47E-11
actin and actin related protein	30	5	.68	7.35	+	9.86E-04	6.20E-03
actin binding motor protein	27	4	.61	6.53	+	4.68E-03	2.47E-02
aminoacyl-tRNA synthetase	34	5	.77	6.48	+	1.63E-03	8.81E-03

↳translational protein	288	21	6.53	3.21	+	6.33E-06	8.23E-05
protease inhibitor	157	20	3.56	5.62	+	2.81E-09	6.08E-08
↳protein-binding activity modulator	632	33	14.33	2.30	+	2.04E-05	1.99E-04
reductase	51	6	1.16	5.19	+	1.61E-03	8.97E-03
↳oxidoreductase	418	26	9.48	2.74	+	7.83E-06	9.54E-05
↳metabolite interconversion enzyme	1428	59	32.39	1.82	+	1.49E-05	1.62E-04
growth factor	90	10	2.04	4.90	+	7.66E-05	6.49E-04
↳intercellular signal molecule	343	21	7.78	2.70	+	7.16E-05	6.34E-04
metalloprotease	167	13	3.79	3.43	+	2.00E-04	1.56E-03
↳protease	597	32	13.54	2.36	+	1.38E-05	1.58E-04
ribosomal protein	159	11	3.61	3.05	+	1.50E-03	8.58E-03
cysteine protease	136	9	3.08	2.92	+	5.14E-03	2.64E-02
Unclassified	12347	190	280.05	.68	-	1.96E-15	1.27E-13
transporter	687	4	15.58	.26	-	9.31E-04	6.05E-03
RNA binding protein	615	3	13.95	.22	-	1.18E-03	6.99E-03
↳nucleic acid binding protein	858	5	19.46	.26	-	2.10E-04	1.58E-03
immunoglobulin	389	0	8.82	< 0.01	-	3.31E-04	2.39E-03
↳defense/immunity protein	600	3	13.61	.22	-	1.12E-03	6.82E-03
C2H2 zinc finger transcription factor	424	0	9.62	< 0.01	-	1.52E-04	1.23E-03
↳zinc finger transcription factor	502	0	11.39	< 0.01	-	2.01E-05	2.06E-04
↳DNA-binding transcription factor	970	1	22.00	.05	-	9.59E-09	1.87E-07
↳gene-specific transcriptional regulator	1054	2	23.91	.08	-	2.06E-08	3.66E-07
G-protein coupled receptor	568	0	12.88	< 0.01	-	4.11E-06	5.72E-05
↳transmembrane signal receptor	712	4	16.15	.25	-	6.78E-04	4.56E-03

© Copyright 2020 Paul Thomas All Rights Reserved.

Analysis Summary: Please report in publication BM-MSC secretome from HFD mice

Analysis Type: PANTHER Overrepresentation Test (Released 20190711)

Annotation Version and Release Date: PANTHER version 15 Released 2020-02-14

Analyzed List: Client Text Box Input (Mus musculus) Change

Reference List: Mus musculus (all genes in database) Change

Annotation Data Set: PANTHER Protein Class

Test Type: (•) Fisher's Exact ( ) Binomial

Correction: (•) Calculate False Discovery Rate ( ) Use the Bonferroni correction for multiple testing ( ) No correction

Results

	Reference list	Client Text Box Input
Uniquely Mapped IDS:	22265 out of 22265	438 out of 438
Unmapped IDs:	0	3
Multiple mapping information:	0	9

Export Table XML with user input ids JSON with user input ids View: -- Please select a chart to display --

Displaying only results for FDR P < 0.05, click here to display all results

	Mus musculus (REF)	Client Text Box Input (Hierarchy NEW!)					
PANTHER Protein Class	#	#	expected	Fold Enrichment	+/-	raw P value	FDR
Hsp90 family chaperone	4	3	.08	38.12	+	2.36E-04	2.09E-03
↳chaperone	74	7	1.46	4.81	+	9.66E-04	6.73E-03
extracellular matrix structural protein	73	20	1.44	13.93	+	9.68E-16	6.29E-14
↳extracellular matrix protein	161	30	3.17	9.47	+	7.26E-19	1.42E-16
chemokine	15	4	.30	13.56	+	4.21E-04	3.42E-03
↳cytokine	77	11	1.51	7.26	+	1.03E-06	1.54E-05
↳intercellular signal molecule	343	22	6.75	3.26	+	2.83E-06	3.95E-05
actin and actin related protein	30	5	.59	8.47	+	5.26E-04	3.95E-03
↳actin or actin-binding cytoskeletal protein	236	32	4.64	6.89	+	1.91E-16	1.86E-14
↳cytoskeletal protein	492	34	9.68	3.51	+	9.07E-10	2.21E-08
non-motor actin binding protein	93	15	1.83	8.20	+	2.56E-09	5.55E-08
protease inhibitor	157	24	3.09	7.77	+	1.17E-13	5.70E-12
↳protein-binding activity modulator	632	29	12.43	2.33	+	4.08E-05	4.42E-04

actin binding motor protein	27	4	.53	7.53	+	2.85E-03	1.73E-02
calmodulin-related	62	9	1.22	7.38	+	8.82E-06	1.15E-04
↳calcium-binding protein	79	15	1.55	9.65	+	3.48E-10	9.69E-09
cysteine protease	136	11	2.68	4.11	+	1.37E-04	1.27E-03
↳protease	597	34	11.74	2.90	+	8.29E-08	1.47E-06
↳protein modifying enzyme	1204	38	23.69	1.60	+	5.44E-03	3.03E-02
serine protease	229	14	4.50	3.11	+	2.88E-04	2.44E-03
oxidoreductase	418	23	8.22	2.80	+	1.80E-05	2.20E-04
↳metabolite interconversion enzyme	1428	46	28.09	1.64	+	1.58E-03	9.96E-03
metalloprotease	167	9	3.29	2.74	+	7.34E-03	3.97E-02
Unclassified	12347	168	242.89	.69	-	1.30E-12	5.05E-11
transporter	687	4	13.51	.30	-	4.59E-03	2.63E-02
RNA binding protein	615	2	12.10	.17	-	9.00E-04	6.50E-03
↳nucleic acid binding protein	858	3	16.88	.18	-	7.06E-05	7.24E-04
immunoglobulin	389	0	7.65	< 0.01	-	1.09E-03	7.33E-03
↳defense/immunity protein	600	2	11.80	.17	-	1.31E-03	8.49E-03
C2H2 zinc finger transcription factor	424	0	8.34	< 0.01	-	4.81E-04	3.75E-03
↳zinc finger transcription factor	502	0	9.88	< 0.01	-	9.88E-05	9.63E-04
↳DNA-binding transcription factor	970	1	19.08	.05	-	1.41E-07	2.29E-06
↳gene-specific transcriptional regulator	1054	1	20.73	.05	-	2.94E-08	5.73E-07
G-protein coupled receptor	568	0	11.17	< 0.01	-	3.17E-05	3.63E-04
↳transmembrane signal receptor	712	4	14.01	.29	-	3.36E-03	1.99E-02

© Copyright 2020 Paul Thomas All Rights Reserved.

Analysis Summary: Please report in publication

vWAT-MSC secretome from HFD mice

Analysis Type: PANTHER Overrepresentation Test (Released 20190711)

Annotation Version and Release Date: PANTHER version 15 Released 2020-02-14

Analyzed List: Client Text Box Input (Mus musculus) Change

Reference List: Mus musculus (all genes in database) Change

Annotation Data Set: PANTHER GO-Slim Molecular Function

Test Type: (•) Fisher's Exact ( ) Binomial

Correction: Calculate False Discovery Rate Use the Bonferroni correction for multiple testing No correction

Results

	Reference list	Client Text Box Input
Uniquely Mapped IDS:	22265 out of 22265	377 out of 378
Unmapped IDs:	0	3
Multiple mapping information:	0	10

Export Table XML with user input ids JSON with user input ids View: -- Please select a chart to display --

Displaying only results for FDR P < 0.05, click here to display all results

	Mus musculus (REF)	Client Text Box Input (Hierarchy NEW!)					
PANTHER GO-Slim Molecular Function	#	#	expected	Fold Enrichment	+/-	raw P value	FDR
extracellular matrix binding	10	3	.17	17.67	+	1.17E-03	1.19E-02
↳binding	4859	127	82.49	1.54	+	1.71E-07	5.05E-06
extracellular matrix structural constituent	11	3	.19	16.06	+	1.47E-03	1.44E-02
↳structural molecule activity	218	21	3.70	5.67	+	6.90E-10	4.07E-08
unfolded protein binding	45	10	.76	13.09	+	2.24E-08	7.92E-07
↳protein binding	2575	84	43.72	1.92	+	6.26E-09	2.56E-07
actin filament binding	84	18	1.43	12.62	+	8.56E-14	7.58E-12
↳actin binding	129	23	2.19	10.50	+	9.68E-16	1.28E-13
↳cytoskeletal protein binding	340	27	5.77	4.68	+	1.42E-10	1.08E-08
↳protein-containing complex binding	283	29	4.80	6.04	+	8.52E-14	9.05E-12
rRNA binding	25	4	.42	9.42	+	1.30E-03	1.31E-02
copper ion binding	19	3	.32	9.30	+	5.62E-03	4.52E-02
↳transition metal ion binding	87	7	1.48	4.74	+	1.01E-03	1.05E-02
↳metal ion binding	219	17	3.72	4.57	+	5.22E-07	1.39E-05
↳cation binding	314	17	5.33	3.19	+	4.64E-05	6.66E-04
↳ion binding	711	35	12.07	2.90	+	4.36E-08	1.45E-06
ATP binding	32	5	.54	9.20	+	3.55E-04	4.09E-03
↳purine ribonucleotide binding	167	11	2.84	3.88	+	2.10E-04	2.60E-03
↳ribonucleotide binding	173	11	2.94	3.75	+	2.80E-04	3.38E-03

↳nucleotide binding	220	14	3.74	3.75	+	4.25E-05	6.45E-04
↳nucleoside phosphate binding	220	14	3.74	3.75	+	4.25E-05	6.64E-04
↳small molecule binding	358	18	6.08	2.96	+	6.93E-05	9.43E-04
↳carbohydrate derivative binding	242	16	4.11	3.89	+	7.79E-06	1.53E-04
↳purine nucleotide binding	179	12	3.04	3.95	+	9.36E-05	1.24E-03
↳purine ribonucleoside triphosphate binding	142	8	2.41	3.32	+	3.78E-03	3.29E-02
↳anion binding	473	18	8.03	2.24	+	1.88E-03	1.82E-02
↳drug binding	117	10	1.99	5.03	+	5.52E-05	7.72E-04
integrin binding	28	4	.48	8.41	+	1.90E-03	1.80E-02
↳cell adhesion molecule binding	70	7	1.19	5.89	+	3.01E-04	3.56E-03
isomerase activity	85	12	1.44	8.32	+	7.84E-08	2.45E-06
↳catalytic activity	3715	114	63.07	1.81	+	1.66E-10	1.10E-08
oxidoreductase activity, acting on peroxide as acceptor	36	4	.61	6.54	+	4.35E-03	3.66E-02
↳oxidoreductase activity	443	24	7.52	3.19	+	1.29E-06	2.99E-05
structural constituent of ribosome	128	12	2.17	5.52	+	4.22E-06	9.33E-05
metallopeptidase activity	75	7	1.27	5.50	+	4.44E-04	5.01E-03
↳peptidase activity, acting on L-amino acid peptides	454	41	7.71	5.32	+	2.91E-17	7.73E-15
↳peptidase activity	463	41	7.86	5.22	+	5.50E-17	9.73E-15
↳hydrolase activity	1631	62	27.69	2.24	+	4.49E-09	2.38E-07
↳catalytic activity, acting on a protein	1447	57	24.57	2.32	+	6.94E-09	2.63E-07
endopeptidase inhibitor activity	108	10	1.83	5.45	+	2.94E-05	4.88E-04
↳peptidase inhibitor activity	108	10	1.83	5.45	+	2.94E-05	4.73E-04
↳peptidase regulator activity	114	11	1.94	5.68	+	8.23E-06	1.56E-04
↳enzyme regulator activity	389	18	6.60	2.73	+	1.86E-04	2.36E-03
↳molecular function regulator	728	26	12.36	2.10	+	6.77E-04	7.33E-03
↳enzyme inhibitor activity	149	12	2.53	4.74	+	1.76E-05	3.01E-04
protease binding	119	11	2.02	5.44	+	1.20E-05	2.19E-04
↳enzyme binding	618	21	10.49	2.00	+	4.00E-03	3.43E-02
serine-type endopeptidase activity	148	12	2.51	4.78	+	1.65E-05	2.93E-04
↳serine hydrolase activity	152	13	2.58	5.04	+	4.30E-06	9.14E-05
↳hydrolase activity, acting on acid phosphorus-nitrogen bonds	152	13	2.58	5.04	+	4.30E-06	8.79E-05
↳endopeptidase activity	355	37	6.03	6.14	+	1.63E-17	8.67E-15
calcium ion binding	119	7	2.02	3.46	+	5.29E-03	4.32E-02
Unclassified	13264	168	225.19	.75	-	4.94E-09	2.39E-07
DNA binding	803	4	13.63	.29	-	4.60E-03	3.82E-02
inorganic molecular entity transmembrane transporter activity	550	1	9.34	.11	-	1.93E-03	1.80E-02
↳transmembrane transporter activity	701	1	11.90	.08	-	1.38E-04	1.78E-03
ion transmembrane transporter activity	581	1	9.86	.10	-	8.91E-04	9.46E-03
G protein-coupled receptor activity	405	0	6.88	< 0.01	-	2.41E-03	2.21E-02
↳transmembrane signaling receptor activity	1182	2	20.07	.10	-	5.55E-07	1.40E-05
↳signaling receptor activity	1282	3	21.76	.14	-	8.76E-07	2.11E-05
↳molecular transducer activity	1349	3	22.90	.13	-	2.85E-07	7.97E-06
DNA-binding transcription factor activity, RNA polymerase II-specific	366	0	6.21	< 0.01	-	3.36E-03	3.02E-02
↳DNA-binding transcription factor activity	618	0	10.49	< 0.01	-	4.35E-05	6.42E-04
↳transcription regulator activity	772	2	13.11	.15	-	4.63E-04	5.12E-03
protein serine/threonine kinase activity	374	0	6.35	< 0.01	-	3.42E-03	3.03E-02

© Copyright 2020 Paul Thomas All Rights Reserved.

Analysis Summary: Please report in publication

sWAT-MSC secretome from HFD mice

Analysis Type: PANTHER Overrepresentation Test (Released 20190711)

Annotation Version and Release Date: PANTHER version 15 Released 2020-02-14

Analyzed List: Client Text Box Input (Mus musculus) Change

Reference List: Mus musculus (all genes in database) Change

Annotation Data Set: PANTHER GO-Slim Molecular Function

Test Type: ◉ Fisher's Exact ○ Binomial

Correction: ◉ Calculate False Discovery Rate ○ Use the Bonferroni correction for multiple testing ○ No correction

Results

	Reference list	Client Text Box Input
Uniquely Mapped IDS:	22265 out of 22265	506 out of 505
Unmapped IDs:	0	4
Multiple mapping information:	0	8

Export Table XML with user input ids JSON with user input ids View: -- Please select a chart to display --

Displaying only results for FDR P < 0.05, click here to display all results

	Mus musculus (REF)	Client Text Box Input (Hierarchy NEW!)					
PANTHER GO-Slim Molecular Function	#	#	expected	Fold Enrichment	+/-	raw P value	FDR
extracellular matrix structural constituent	11	5	.25	20.04	+	1.88E-05	2.62E-04
↳structural molecule activity	218	24	4.94	4.85	+	1.07E-09	3.80E-08
extracellular matrix binding	10	4	.23	17.64	+	2.01E-04	1.77E-03
↳binding	4859	170	110.21	1.54	+	1.81E-09	5.65E-08
integrin binding	28	11	.64	17.32	+	5.48E-10	2.24E-08
↳cell adhesion molecule binding	70	14	1.59	8.82	+	4.47E-09	1.32E-07
↳protein binding	2575	118	58.40	2.02	+	2.52E-13	2.23E-11
↳protein-containing complex binding	283	41	6.42	6.39	+	1.77E-19	9.42E-17
disulfide oxidoreductase activity	9	3	.20	14.70	+	2.05E-03	1.42E-02
↳oxidoreductase activity	443	27	10.05	2.69	+	7.44E-06	1.20E-04
↳catalytic activity	3715	146	84.26	1.73	+	1.63E-11	8.68E-10
unfolded protein binding	45	14	1.02	13.72	+	3.11E-11	1.38E-09
heparin binding	27	7	.61	11.43	+	8.10E-06	1.27E-04
↳glycosaminoglycan binding	42	8	.95	8.40	+	1.31E-05	1.88E-04
↳carbohydrate derivative binding	242	24	5.49	4.37	+	7.08E-09	1.88E-07
↳anion binding	473	29	10.73	2.70	+	3.07E-06	5.62E-05
↳ion binding	711	49	16.13	3.04	+	2.56E-11	1.24E-09
actin filament binding	84	19	1.91	9.97	+	1.17E-12	8.91E-11
↳actin binding	129	24	2.93	8.20	+	5.37E-14	5.70E-12
↳cytoskeletal protein binding	340	30	7.71	3.89	+	1.20E-09	3.97E-08

heat shock protein binding	26	5	.59	8.48	+	5.55E-04	4.15E-03
isomerase activity	85	16	1.93	8.30	+	7.65E-10	2.90E-08
2-oxoglutarate-dependent dioxygenase activity	16	3	.36	8.27	+	8.07E-03	4.87E-02
ATP binding	32	6	.73	8.27	+	1.75E-04	1.57E-03
↳purine ribonucleotide binding	167	16	3.79	4.22	+	3.48E-06	6.16E-05
↳ribonucleotide binding	173	16	3.92	4.08	+	5.27E-06	9.03E-05
↳nucleotide binding	220	20	4.99	4.01	+	4.61E-07	1.02E-05
↳nucleoside phosphate binding	220	20	4.99	4.01	+	4.61E-07	1.07E-05
↳small molecule binding	358	22	8.12	2.71	+	4.60E-05	5.43E-04
↳purine nucleotide binding	179	17	4.06	4.19	+	1.93E-06	3.94E-05
↳purine ribonucleoside triphosphate binding	142	12	3.22	3.73	+	1.73E-04	1.58E-03
↳drug binding	117	11	2.65	4.15	+	1.35E-04	1.33E-03
rRNA binding	25	4	.57	7.05	+	3.66E-03	2.43E-02
carboxypeptidase activity	30	4	.68	5.88	+	6.55E-03	4.09E-02
↳peptidase activity, acting on L-amino acid peptides	454	46	10.30	4.47	+	3.41E-16	6.03E-14
↳peptidase activity	463	46	10.50	4.38	+	6.71E-16	8.90E-14
↳hydrolase activity	1631	75	36.99	2.03	+	1.05E-08	2.66E-07
↳catalytic activity, acting on a protein	1447	70	32.82	2.13	+	6.39E-09	1.79E-07
growth factor binding	38	5	.86	5.80	+	2.53E-03	1.72E-02
metalloendopeptidase activity	42	5	.95	5.25	+	3.75E-03	2.46E-02
↳endopeptidase activity	355	41	8.05	5.09	+	2.53E-16	6.72E-14
↳metallopeptidase activity	75	9	1.70	5.29	+	1.02E-04	1.06E-03
endopeptidase inhibitor activity	108	11	2.45	4.49	+	7.00E-05	7.58E-04
↳peptidase inhibitor activity	108	11	2.45	4.49	+	7.00E-05	7.43E-04
↳peptidase regulator activity	114	11	2.59	4.25	+	1.09E-04	1.09E-03
↳enzyme regulator activity	389	22	8.82	2.49	+	2.35E-04	1.98E-03
↳molecular function regulator	728	39	16.51	2.36	+	1.98E-06	3.90E-05
↳enzyme inhibitor activity	149	13	3.38	3.85	+	6.94E-05	7.84E-04
structural constituent of ribosome	128	12	2.90	4.13	+	6.95E-05	7.69E-04
protease binding	119	11	2.70	4.08	+	1.55E-04	1.50E-03
↳enzyme binding	618	25	14.02	1.78	+	6.28E-03	3.97E-02
transition metal ion binding	87	8	1.97	4.05	+	1.25E-03	8.82E-03
↳metal ion binding	219	20	4.97	4.03	+	4.32E-07	1.04E-05
↳cation binding	314	20	7.12	2.81	+	6.36E-05	7.34E-04
serine-type endopeptidase activity	148	12	3.36	3.57	+	2.47E-04	2.05E-03
↳serine hydrolase activity	152	14	3.45	4.06	+	2.12E-05	2.88E-04
↳hydrolase activity, acting on acid phosphorus-nitrogen bonds	152	14	3.45	4.06	+	2.12E-05	2.81E-04
cysteine-type endopeptidase activity	93	7	2.11	3.32	+	6.90E-03	4.26E-02
calcium ion binding	119	8	2.70	2.96	+	7.46E-03	4.55E-02
receptor ligand activity	231	15	5.24	2.86	+	4.22E-04	3.35E-03
↳signaling receptor activator activity	232	15	5.26	2.85	+	4.41E-04	3.44E-03
↳receptor regulator activity	255	16	5.78	2.77	+	3.93E-04	3.16E-03
Unclassified	13264	223	300.85	.74	-	6.26E-12	4.15E-10
protein serine/threonine kinase activity	374	1	8.48	.12	-	3.93E-03	2.55E-02
G protein-coupled receptor activity	405	0	9.19	< 0.01	-	2.21E-04	1.89E-03
↳transmembrane signaling receptor activity	1182	6	26.81	.22	-	2.26E-06	4.28E-05
↳signaling receptor activity	1282	9	29.08	.31	-	2.27E-05	2.94E-04
↳molecular transducer activity	1349	9	30.60	.29	-	6.26E-06	1.04E-04

DNA-binding transcription factor activity, RNA polymerase II-specific	366	0	8.30	< 0.01	-	4.72E-04	3.63E-03
↳DNA-binding transcription factor activity	618	0	14.02	< 0.01	-	1.25E-06	2.65E-05
↳transcription regulator activity	772	2	17.51	.11	-	8.58E-06	1.30E-04
RNA polymerase II regulatory region sequence-specific DNA binding	344	0	7.80	< 0.01	-	6.92E-04	5.03E-03
↳transcription regulatory region sequence-specific DNA binding	372	0	8.44	< 0.01	-	4.91E-04	3.73E-03
↳sequence-specific double-stranded DNA binding	395	0	8.96	< 0.01	-	2.13E-04	1.85E-03
↳sequence-specific DNA binding	538	0	12.20	< 0.01	-	9.18E-06	1.35E-04
↳DNA binding	803	3	18.21	.16	-	2.51E-05	3.18E-04
↳double-stranded DNA binding	444	0	10.07	< 0.01	-	1.05E-04	1.08E-03
↳transcription regulatory region DNA binding	428	0	9.71	< 0.01	-	1.58E-04	1.50E-03
↳regulatory region nucleic acid binding	428	0	9.71	< 0.01	-	1.58E-04	1.47E-03
↳RNA polymerase II regulatory region DNA binding	349	0	7.92	< 0.01	-	7.00E-04	5.03E-03
monovalent inorganic cation transmembrane transporter activity	261	0	5.92	< 0.01	-	4.97E-03	3.18E-02
↳inorganic cation transmembrane transporter activity	395	1	8.96	.11	-	2.77E-03	1.86E-02
↳inorganic molecular entity transmembrane transporter activity	550	2	12.47	.16	-	6.31E-04	4.66E-03
↳transmembrane transporter activity	701	2	15.90	.13	-	3.71E-05	4.48E-04
↳transporter activity	782	3	17.74	.17	-	3.56E-05	4.40E-04
↳cation transmembrane transporter activity	424	1	9.62	.10	-	1.30E-03	9.08E-03
↳ion transmembrane transporter activity	581	2	13.18	.15	-	3.10E-04	2.53E-03

© Copyright 2020 Paul Thomas All Rights Reserved.

Analysis Summary: Please report in publication

BM-MSC secretome from HFD mice

Analysis Type: PANTHER Overrepresentation Test (Released 20190711)

Annotation Version and Release Date: PANTHER version 15 Released 2020-02-14

Analyzed List: Client Text Box Input (Mus musculus) Change

Reference List: Mus musculus (all genes in database) Change

Annotation Data Set: PANTHER GO-Slim Molecular Function

Test Type: Fisher's Exact / Binomial

Correction: Calculate False Discovery Rate / Use the Bonferroni correction for multiple testing / No correction

Results

	Reference list	Client Text Box Input
Uniquely Mapped IDS:	22265 out of 22265	438 out of 438
Unmapped IDs:	0	3
Multiple mapping information:	0	9

Export Table XML with user input ids JSON with user input ids View: -- Please select a chart to display --

Displaying only results for FDR P < 0.05, click here to display all results

	Mus musculus (REF)	Client Text Box Input (Hierarchy NEW!)					
PANTHER GO-Slim Molecular Function	#	#	expected	Fold Enrichment	+/-	raw P value	FDR
extracellular matrix structural constituent	11	5	.22	23.11	+	9.57E-06	1.45E-04
↳structural molecule activity	218	17	4.29	3.96	+	3.53E-06	7.20E-05
extracellular matrix binding	10	4	.20	20.33	+	1.17E-04	1.25E-03
↳binding	4859	142	95.59	1.49	+	4.69E-07	1.19E-05
integrin binding	28	10	.55	18.15	+	1.88E-09	1.11E-07
↳cell adhesion molecule binding	70	13	1.38	9.44	+	6.68E-09	3.22E-07
↳protein binding	2575	102	50.66	2.01	+	1.13E-11	1.00E-09
↳signaling receptor binding	734	27	14.44	1.87	+	2.76E-03	2.12E-02
↳protein-containing complex binding	283	34	5.57	6.11	+	5.71E-16	7.59E-14
heparin binding	27	6	.53	11.30	+	3.55E-05	4.49E-04
↳glycosaminoglycan binding	42	7	.83	8.47	+	4.05E-05	4.67E-04
↳carbohydrate derivative binding	242	18	4.76	3.78	+	3.40E-06	7.22E-05
↳anion binding	473	21	9.30	2.26	+	7.32E-04	6.47E-03
↳ion binding	711	38	13.99	2.72	+	6.71E-08	2.38E-06
unfolded protein binding	45	10	.89	11.30	+	8.68E-08	2.88E-06
actin filament binding	84	16	1.65	9.68	+	8.58E-11	6.51E-09
↳actin binding	129	21	2.54	8.28	+	1.40E-12	1.48E-10
↳cytoskeletal protein binding	340	23	6.69	3.44	+	7.16E-07	1.73E-05
2-oxoglutarate-dependent dioxygenase activity	16	3	.31	9.53	+	5.49E-03	3.65E-02
↳oxidoreductase activity	443	25	8.71	2.87	+	5.17E-06	9.15E-05
↳catalytic activity	3715	123	73.08	1.68	+	3.44E-09	1.83E-07
metalloendopeptidase activity	42	7	.83	8.47	+	4.05E-05	4.78E-04
↳endopeptidase activity	355	44	6.98	6.30	+	7.83E-21	4.16E-18
↳peptidase activity, acting on L-amino acid peptides	454	48	8.93	5.37	+	5.13E-20	1.36E-17
↳peptidase activity	463	48	9.11	5.27	+	1.08E-19	1.90E-17
↳hydrolase activity	1631	68	32.09	2.12	+	1.11E-08	4.92E-07

↳catalytic activity, acting on a protein	1447	68	28.47	2.39	+	8.81E-11	5.85E-09
↳metallopeptidase activity	75	10	1.48	6.78	+	5.55E-06	9.51E-05
ATP binding	32	5	.63	7.94	+	6.85E-04	6.27E-03
↳purine ribonucleotide binding	167	10	3.29	3.04	+	2.38E-03	1.86E-02
↳ribonucleotide binding	173	10	3.40	2.94	+	3.03E-03	2.26E-02
↳nucleotide binding	220	13	4.33	3.00	+	6.35E-04	5.92E-03
↳nucleoside phosphate binding	220	13	4.33	3.00	+	6.35E-04	6.02E-03
↳purine nucleotide binding	179	10	3.52	2.84	+	3.81E-03	2.77E-02
↳drug binding	117	8	2.30	3.48	+	2.97E-03	2.26E-02
isomerase activity	85	12	1.67	7.18	+	3.71E-07	9.85E-06
carboxypeptidase activity	30	4	.59	6.78	+	4.01E-03	2.76E-02
growth factor binding	38	5	.75	6.69	+	1.38E-03	1.14E-02
endopeptidase inhibitor activity	108	12	2.12	5.65	+	3.68E-06	6.98E-05
↳peptidase inhibitor activity	108	12	2.12	5.65	+	3.68E-06	6.74E-05
↳peptidase regulator activity	114	12	2.24	5.35	+	6.13E-06	1.02E-04
↳enzyme regulator activity	389	18	7.65	2.35	+	1.36E-03	1.15E-02
↳molecular function regulator	728	36	14.32	2.51	+	1.15E-06	2.64E-05
↳enzyme inhibitor activity	149	14	2.93	4.78	+	3.57E-06	7.01E-05
cysteine-type endopeptidase activity	93	10	1.83	5.47	+	3.08E-05	4.08E-04
↳cysteine-type peptidase activity	158	11	3.11	3.54	+	4.59E-04	4.43E-03
protease binding	119	12	2.34	5.13	+	9.16E-06	1.43E-04
cytokine activity	116	11	2.28	4.82	+	3.64E-05	4.49E-04
↳receptor ligand activity	231	16	4.54	3.52	+	2.69E-05	3.76E-04
↳signaling receptor activator activity	232	16	4.56	3.51	+	2.82E-05	3.85E-04
↳receptor regulator activity	255	17	5.02	3.39	+	2.40E-05	3.45E-04
serine-type endopeptidase activity	148	14	2.91	4.81	+	3.32E-06	7.34E-05
↳serine hydrolase activity	152	16	2.99	5.35	+	1.77E-07	5.54E-06
↳hydrolase activity, acting on acid phosphorus-nitrogen bonds	152	16	2.99	5.35	+	1.77E-07	5.23E-06
cytokine receptor binding	122	11	2.40	4.58	+	5.57E-05	6.16E-04
transition metal ion binding	87	7	1.71	4.09	+	2.30E-03	1.82E-02
↳metal ion binding	219	16	4.31	3.71	+	1.46E-05	2.15E-04
↳cation binding	314	17	6.18	2.75	+	2.64E-04	2.75E-03
oxidoreductase activity, acting on the CH-OH group of donors, NAD or NADP as acceptor	76	6	1.50	4.01	+	5.08E-03	3.42E-02
G protein-coupled receptor binding	136	9	2.68	3.36	+	2.06E-03	1.66E-02
Unclassified	13264	203	260.93	.78	-	4.22E-08	1.60E-06
sequence-specific DNA binding	538	2	10.58	.19	-	3.70E-03	2.73E-02
↳DNA binding	803	4	15.80	.25	-	9.44E-04	8.22E-03
↳nucleic acid binding	1293	12	25.44	.47	-	4.80E-03	3.27E-02
cation transmembrane transporter activity	424	1	8.34	.12	-	3.89E-03	2.79E-02
↳ion transmembrane transporter activity	581	1	11.43	.09	-	3.13E-04	3.14E-03
↳transmembrane transporter activity	701	1	13.79	.07	-	3.26E-05	4.22E-04
↳transporter activity	782	3	15.38	.20	-	2.88E-04	2.94E-03
transcription regulatory region DNA binding	428	1	8.42	.12	-	3.91E-03	2.77E-02
↳regulatory region nucleic acid binding	428	1	8.42	.12	-	3.91E-03	2.74E-02
inorganic molecular entity transmembrane transporter activity	550	1	10.82	.09	-	4.30E-04	4.23E-03
G protein-coupled receptor activity	405	0	7.97	< 0.01	-	7.16E-04	6.44E-03
↳transmembrane signaling receptor activity	1182	3	23.25	.13	-	3.23E-07	9.03E-06
↳signaling receptor activity	1282	8	25.22	.32	-	9.72E-05	1.05E-03
↳molecular transducer activity	1349	8	26.54	.30	-	3.79E-05	4.57E-04
DNA-binding transcription factor activity, RNA polymerase II-specific	366	0	7.20	< 0.01	-	1.58E-03	1.29E-02
↳DNA-binding transcription factor activity	618	0	12.16	< 0.01	-	9.09E-06	1.46E-04
↳transcription regulator activity	772	2	15.19	.13	-	5.07E-05	5.73E-04
protein serine/threonine kinase activity	374	0	7.36	< 0.01	-	1.03E-03	8.82E-03

© Copyright 2020 Paul Thomas All Rights Reserved.

Analysis Summary: Please report in publication

vWAT-MSC secretome from HFD mice

Analysis Type: PANTHER Overrepresentation Test (Released 20190711)

Annotation Version and Release Date: PANTHER version 15 Released 2020-02-14

Analyzed List: Client Text Box Input (Mus musculus) Change

Reference List: Mus musculus (all genes in database) Change

Annotation Data Set: PANTHER GO-Slim Biological Process

Test Type: (•) Fisher's Exact ( ) Binomial

Correction: (•) Calculate False Discovery Rate ( ) Use the Bonferroni correction for multiple testing ( ) No correction

Results

	Reference list	Client Text Box Input
Uniquely Mapped IDS:	22265 out of 22265	377 out of 378
Unmapped IDs:	0	3
Multiple mapping information:	0	10

Export Table XML with user input ids JSON with user input ids View: -- Please select a chart to display --

Displaying only results for FDR P < 0.05, click here to display all results

	Mus musculus (REF)	Client Text Box Input (Hierarchy NEW!)					
PANTHER GO-Slim Biological Process	#	#	expected	Fold Enrichment	+/-	raw P value	FDR
tissue remodeling	3	2	.05	39.27	+	2.69E-03	3.70E-02
collagen fibril organization	11	5	.19	26.77	+	4.74E-06	2.08E-04
↳supramolecular fiber organization	236	28	4.01	6.99	+	8.37E-15	1.73E-11
↳cellular component organization	2396	64	40.68	1.57	+	3.14E-04	6.89E-03
↳cellular process	7925	197	134.55	1.46	+	8.54E-11	1.26E-08
↳cellular component organization or biogenesis	2526	66	42.88	1.54	+	4.38E-04	9.12E-03
↳extracellular matrix organization	56	11	.95	11.57	+	1.35E-08	1.47E-06
↳extracellular structure organization	63	11	1.07	10.28	+	3.96E-08	3.89E-06
↳protein-containing complex subunit organization	481	27	8.17	3.31	+	1.43E-07	1.05E-05
response to reactive oxygen species	11	5	.19	26.77	+	4.74E-06	2.04E-04
↳response to oxidative stress	25	9	.42	21.20	+	3.32E-09	4.03E-07
↳response to stress	972	30	16.50	1.82	+	2.17E-03	3.14E-02
cellular response to oxidative stress	16	7	.27	25.77	+	6.66E-08	5.50E-06
↳cellular response to stress	444	19	7.54	2.52	+	3.21E-04	6.97E-03
IMP metabolic process	7	3	.12	25.24	+	5.08E-04	9.70E-03
↳nucleoside monophosphate metabolic process	14	3	.24	12.62	+	2.64E-03	3.68E-02
↳nucleoside phosphate metabolic process	168	9	2.85	3.16	+	3.00E-03	4.11E-02
↳nucleobase-containing small molecule metabolic process	202	11	3.43	3.21	+	9.54E-04	1.55E-02
↳metabolic process	4857	119	82.46	1.44	+	1.85E-05	6.58E-04
↳cellular metabolic process	4357	111	73.97	1.50	+	6.11E-06	2.47E-04
↳organic substance metabolic process	4680	111	79.45	1.40	+	1.68E-04	4.13E-03
↳primary metabolic process	4458	102	75.68	1.35	+	1.19E-03	1.88E-02
↳nitrogen compound metabolic process	4212	103	71.51	1.44	+	9.10E-05	2.38E-03

↳organophosphate metabolic process	312	13	5.30	2.45	+	3.40E-03	4.53E-02
↳purine ribonucleotide metabolic process	130	9	2.21	4.08	+	5.58E-04	1.05E-02
↳purine nucleotide metabolic process	138	9	2.34	3.84	+	8.35E-04	1.39E-02
↳purine-containing compound metabolic process	152	9	2.58	3.49	+	1.58E-03	2.36E-02
↳organonitrogen compound metabolic process	2527	89	42.90	2.07	+	3.60E-11	6.75E-09
↳nucleotide metabolic process	163	9	2.77	3.25	+	2.48E-03	3.51E-02
↳ribonucleotide metabolic process	135	9	2.29	3.93	+	7.20E-04	1.24E-02
↳ribose phosphate metabolic process	143	9	2.43	3.71	+	1.06E-03	1.68E-02
glucose 6-phosphate metabolic process	9	3	.15	19.63	+	9.08E-04	1.49E-02
bone morphogenesis	14	4	.24	16.83	+	1.94E-04	4.66E-03
↳bone development	21	4	.36	11.22	+	7.33E-04	1.25E-02
↳skeletal system development	54	5	.92	5.45	+	3.02E-03	4.10E-02
glycolytic process	20	5	.34	14.73	+	5.09E-05	1.50E-03
↳generation of precursor metabolites and energy	103	11	1.75	6.29	+	3.36E-06	1.61E-04
↳nucleotide phosphorylation	20	5	.34	14.73	+	5.09E-05	1.48E-03
↳organic substance catabolic process	703	46	11.94	3.85	+	2.58E-14	2.67E-11
↳catabolic process	888	52	15.08	3.45	+	3.02E-14	2.08E-11
↳carbohydrate metabolic process	133	8	2.26	3.54	+	2.59E-03	3.63E-02
↳ATP metabolic process	56	6	.95	6.31	+	5.82E-04	1.04E-02
chondrocyte differentiation	17	4	.29	13.86	+	3.65E-04	7.85E-03
↳cartilage development	18	4	.31	13.09	+	4.41E-04	8.92E-03
↳connective tissue development	18	4	.31	13.09	+	4.41E-04	9.01E-03
↳tissue development	179	11	3.04	3.62	+	3.69E-04	7.76E-03
superoxide metabolic process	18	4	.31	13.09	+	4.41E-04	9.10E-03
↳reactive oxygen species metabolic process	40	6	.68	8.84	+	1.11E-04	2.79E-03
Arp2/3 complex-mediated actin nucleation	18	4	.31	13.09	+	4.41E-04	8.83E-03
↳positive regulation of actin filament polymerization	34	4	.58	6.93	+	3.60E-03	4.74E-02
↳regulation of actin filament polymerization	71	10	1.21	8.30	+	9.78E-07	5.77E-05
↳regulation of protein polymerization	75	10	1.27	7.85	+	1.54E-06	7.92E-05
↳regulation of protein complex assembly	94	10	1.60	6.27	+	9.68E-06	3.70E-04
↳regulation of cellular component biogenesis	140	11	2.38	4.63	+	4.85E-05	1.45E-03
↳regulation of actin polymerization or depolymerization	73	11	1.24	8.88	+	1.52E-07	1.04E-05
↳regulation of actin filament organization	81	11	1.38	8.00	+	3.89E-07	2.51E-05
↳regulation of actin cytoskeleton organization	106	11	1.80	6.11	+	4.33E-06	1.99E-04
↳regulation of actin filament-based process	107	12	1.82	6.61	+	7.51E-07	4.56E-05
↳regulation of cytoskeleton organization	146	12	2.48	4.84	+	1.46E-05	5.27E-04
↳regulation of organelle organization	264	13	4.48	2.90	+	8.31E-04	1.40E-02
↳regulation of supramolecular fiber organization	90	11	1.53	7.20	+	1.01E-06	5.62E-05
↳regulation of actin filament length	73	11	1.24	8.88	+	1.52E-07	1.08E-05
↳regulation of cellular component size	108	11	1.83	6.00	+	5.11E-06	2.15E-04
↳regulation of anatomical structure size	120	12	2.04	5.89	+	2.27E-06	1.15E-04
↳regulation of biological quality	905	30	15.36	1.95	+	6.14E-04	1.09E-02
↳positive regulation of supramolecular fiber organization	48	6	.81	7.36	+	2.73E-04	6.20E-03
↳positive regulation of cellular component organization	136	10	2.31	4.33	+	1.77E-04	4.29E-03
↳positive regulation of cytoskeleton organization	49	6	.83	7.21	+	3.02E-04	6.79E-03
↳positive regulation of organelle organization	79	8	1.34	5.96	+	1.04E-04	2.65E-03
↳actin filament organization	159	21	2.70	7.78	+	3.22E-12	1.33E-09
↳actin cytoskeleton organization	238	24	4.04	5.94	+	1.70E-11	3.50E-09
↳cytoskeleton organization	560	30	9.51	3.16	+	7.49E-08	5.95E-06
↳actin filament-based process	246	25	4.18	5.99	+	5.45E-12	1.87E-09
response to toxic substance	32	6	.54	11.04	+	3.66E-05	1.11E-03

developmental growth	51	8	.87	9.24	+	5.91E-06	2.44E-04
↳growth	55	8	.93	8.57	+	9.74E-06	3.65E-04
glucose metabolic process	26	4	.44	9.06	+	1.49E-03	2.27E-02
chaperone-mediated protein folding	26	4	.44	9.06	+	1.49E-03	2.26E-02
↳protein folding	81	15	1.38	10.91	+	6.34E-11	1.09E-08
muscle contraction	43	6	.73	8.22	+	1.59E-04	3.95E-03
↳muscle system process	45	6	.76	7.85	+	1.99E-04	4.71E-03
actin filament polymerization	74	10	1.26	7.96	+	1.38E-06	7.28E-05
↳protein polymerization	89	10	1.51	6.62	+	6.22E-06	2.47E-04
↳cellular protein-containing complex assembly	367	19	6.23	3.05	+	3.00E-05	1.00E-03
↳protein-containing complex assembly	394	19	6.69	2.84	+	7.43E-05	1.97E-03
↳cellular component assembly	734	26	12.46	2.09	+	7.09E-04	1.24E-02
↳cellular component biogenesis	867	28	14.72	1.90	+	1.87E-03	2.75E-02
↳actin polymerization or depolymerization	79	12	1.34	8.95	+	3.80E-08	3.92E-06
actin filament bundle assembly	31	4	.53	7.60	+	2.66E-03	3.68E-02
↳actin filament bundle organization	33	4	.56	7.14	+	3.27E-03	4.38E-02
ribosomal small subunit biogenesis	51	6	.87	6.93	+	3.68E-04	7.83E-03
response to inorganic substance	54	6	.92	6.54	+	4.87E-04	9.40E-03
negative regulation of endopeptidase activity	82	9	1.39	6.46	+	2.15E-05	7.40E-04
↳negative regulation of peptidase activity	87	9	1.48	6.09	+	3.31E-05	1.03E-03
↳negative regulation of hydrolase activity	102	10	1.73	5.77	+	1.86E-05	6.52E-04
↳regulation of hydrolase activity	287	14	4.87	2.87	+	5.80E-04	1.05E-02
↳negative regulation of catalytic activity	169	11	2.87	3.83	+	2.32E-04	5.32E-03
↳negative regulation of molecular function	196	11	3.33	3.31	+	7.55E-04	1.28E-02
↳regulation of peptidase activity	112	11	1.90	5.79	+	7.04E-06	2.74E-04
↳regulation of proteolysis	155	12	2.63	4.56	+	2.54E-05	8.58E-04
↳negative regulation of proteolysis	88	9	1.49	6.02	+	3.59E-05	1.11E-03
↳negative regulation of cellular protein metabolic process	164	10	2.78	3.59	+	7.14E-04	1.24E-02
↳negative regulation of protein metabolic process	168	11	2.85	3.86	+	2.21E-04	5.12E-03
↳regulation of endopeptidase activity	107	11	1.82	6.06	+	4.71E-06	2.11E-04
response to endoplasmic reticulum stress	65	6	1.10	5.44	+	1.20E-03	1.87E-02
transition metal ion homeostasis	55	5	.93	5.35	+	3.25E-03	4.38E-02
proteasome-mediated ubiquitin-dependent protein catabolic process	194	17	3.29	5.16	+	1.06E-07	8.08E-06
↳proteasomal protein catabolic process	205	18	3.48	5.17	+	4.32E-08	4.06E-06
↳proteolysis involved in cellular protein catabolic process	331	26	5.62	4.63	+	3.91E-10	5.37E-08
↳proteolysis	693	41	11.77	3.48	+	1.49E-11	3.84E-09
↳protein metabolic process	2115	68	35.91	1.89	+	4.04E-07	2.53E-05
↳cellular protein catabolic process	333	26	5.65	4.60	+	4.41E-10	5.68E-08
↳cellular macromolecule catabolic process	429	28	7.28	3.84	+	4.00E-09	4.59E-07
↳macromolecule catabolic process	463	32	7.86	4.07	+	7.63E-11	1.21E-08
↳cellular catabolic process	765	44	12.99	3.39	+	6.11E-12	1.80E-09
↳protein catabolic process	355	29	6.03	4.81	+	1.53E-11	3.51E-09
↳organonitrogen compound catabolic process	457	35	7.76	4.51	+	6.20E-13	3.20E-10
↳cellular protein metabolic process	1711	60	29.05	2.07	+	1.77E-07	1.18E-05
↳ubiquitin-dependent protein catabolic process	276	18	4.69	3.84	+	2.54E-06	1.25E-04
↳modification-dependent protein catabolic process	285	19	4.84	3.93	+	9.84E-07	5.64E-05
↳modification-dependent macromolecule catabolic process	290	19	4.92	3.86	+	1.25E-06	6.82E-05
maintenance of location	82	6	1.39	4.31	+	3.57E-03	4.73E-02
oxidation-reduction process	220	15	3.74	4.02	+	1.06E-05	3.91E-04
cofactor metabolic process	168	10	2.85	3.51	+	8.51E-04	1.40E-02
cell migration	255	15	4.33	3.46	+	5.35E-05	1.49E-03
↳cell motility	285	16	4.84	3.31	+	5.15E-05	1.46E-03

↳locomotion	356	16	6.04	2.65	+	5.67E-04	1.06E-02
↳localization of cell	285	16	4.84	3.31	+	5.15E-05	1.48E-03
translational elongation	216	12	3.67	3.27	+	4.80E-04	9.43E-03
↳translation	216	12	3.67	3.27	+	4.80E-04	9.35E-03
↳peptide biosynthetic process	219	12	3.72	3.23	+	5.39E-04	1.02E-02
↳organonitrogen compound biosynthetic process	589	21	10.00	2.10	+	1.87E-03	2.74E-02
↳amide biosynthetic process	252	12	4.28	2.80	+	1.71E-03	2.53E-02
↳cellular amide metabolic process	369	18	6.26	2.87	+	9.98E-05	2.57E-03
↳peptide metabolic process	286	18	4.86	3.71	+	4.06E-06	1.90E-04
Unclassified	12808	164	217.45	.75	-	5.51E-08	4.94E-06
regulation of transcription by RNA polymerase II	745	2	12.65	.16	-	6.49E-04	1.14E-02
↳regulation of transcription, DNA-templated	1117	4	18.96	.21	-	6.13E-05	1.69E-03
↳regulation of gene expression	1368	9	23.22	.39	-	1.04E-03	1.68E-02
↳regulation of nucleic acid-templated transcription	1117	4	18.96	.21	-	6.13E-05	1.66E-03
↳regulation of RNA biosynthetic process	1117	4	18.96	.21	-	6.13E-05	1.64E-03
↳regulation of RNA metabolic process	1222	8	20.75	.39	-	1.91E-03	2.78E-02
↳regulation of nucleobase-containing compound metabolic process	1263	8	21.44	.37	-	1.44E-03	2.22E-02
↳regulation of cellular biosynthetic process	1234	7	20.95	.33	-	5.78E-04	1.07E-02
↳regulation of biosynthetic process	1240	7	21.05	.33	-	5.78E-04	1.06E-02
↳regulation of macromolecule biosynthetic process	1223	6	20.76	.29	-	2.14E-04	5.03E-03
↳regulation of cellular macromolecule biosynthetic process	1216	6	20.64	.29	-	3.07E-04	6.82E-03
transcription by RNA polymerase II	772	2	13.11	.15	-	4.63E-04	9.18E-03
↳transcription, DNA-templated	1159	4	19.68	.20	-	3.04E-05	9.80E-04
↳nucleic acid-templated transcription	1159	4	19.68	.20	-	3.04E-05	9.95E-04
↳RNA biosynthetic process	1166	4	19.80	.20	-	3.06E-05	9.72E-04
↳nucleobase-containing compound biosynthetic process	1376	9	23.36	.39	-	1.05E-03	1.68E-02
↳aromatic compound biosynthetic process	1399	10	23.75	.42	-	2.41E-03	3.43E-02
↳organic cyclic compound biosynthetic process	1433	10	24.33	.41	-	1.34E-03	2.08E-02
↳heterocycle biosynthetic process	1399	10	23.75	.42	-	2.41E-03	3.46E-02
↳nucleic acid metabolic process	1911	16	32.44	.49	-	1.49E-03	2.25E-02

© Copyright 2020 Paul Thomas All Rights Reserved.

Analysis Summary: Please report in publication

sWAT-MSC secretome from HFD mice

Analysis Type: PANTHER Overrepresentation Test (Released 20190711)

Annotation Version and Release Date: PANTHER version 15 Released 2020-02-14

Analyzed List: Client Text Box Input (Mus musculus) Change

Reference List: Mus musculus (all genes in database) Change

Annotation Data Set: PANTHER GO-Slim Biological Process

Test Type: ◉ Fisher's Exact ○ Binomial

Correction: ◉ Calculate False Discovery Rate ○ Use the Bonferroni correction for multiple testing ○ No correction

Results

	Reference list	Client Text Box Input
Uniquely Mapped IDS:	22265 out of 22265	506 out of 505
Unmapped IDs:	0	4
Multiple mapping information:	0	8

Export Table XML with user input ids JSON with user input ids View: -- Please select a chart to display --

Displaying only results for FDR P < 0.05, click here to display all results

	Mus musculus (REF)	Client Text Box Input (Hierarchy NEW!)					
PANTHER GO-Slim Biological Process	#	#	expected	Fold Enrichment	+/-	raw P value	FDR
tissue remodeling	3	2	.07	29.39	+	4.70E-03	4.23E-02
collagen fibril organization	11	5	.25	20.04	+	1.88E-05	4.67E-04
↳supramolecular fiber organization	236	30	5.35	5.60	+	3.10E-13	3.20E-10
↳cellular component organization	2396	82	54.34	1.51	+	2.14E-04	3.62E-03
↳cellular process	7925	251	179.75	1.40	+	1.83E-10	7.54E-08
↳cellular component organization or biogenesis	2526	85	57.29	1.48	+	2.95E-04	4.69E-03
↳extracellular matrix organization	56	13	1.27	10.23	+	3.36E-09	5.33E-07
↳extracellular structure organization	63	13	1.43	9.10	+	1.15E-08	9.90E-07
↳protein-containing complex subunit organization	481	33	10.91	3.02	+	5.70E-08	3.36E-06
positive chemotaxis	7	3	.16	18.90	+	1.16E-03	1.46E-02
↳chemotaxis	186	12	4.22	2.84	+	1.63E-03	1.86E-02
↳response to chemical	915	39	20.75	1.88	+	2.71E-04	4.40E-03
↳taxis	186	12	4.22	2.84	+	1.63E-03	1.87E-02
↳locomotion	356	28	8.07	3.47	+	4.27E-08	2.84E-06
sprouting angiogenesis	7	3	.16	18.90	+	1.16E-03	1.45E-02
↳angiogenesis	38	5	.86	5.80	+	2.53E-03	2.62E-02
↳anatomical structure morphogenesis	450	25	10.21	2.45	+	8.88E-05	1.70E-03
↳blood vessel morphogenesis	39	5	.88	5.65	+	2.80E-03	2.88E-02
↳blood vessel development	42	5	.95	5.25	+	3.75E-03	3.63E-02
↳vasculature development	45	5	1.02	4.90	+	4.90E-03	4.38E-02
↳cardiovascular system development	46	5	1.04	4.79	+	5.34E-03	4.61E-02
IMP metabolic process	7	3	.16	18.90	+	1.16E-03	1.44E-02
↳nucleoside monophosphate metabolic process	14	3	.32	9.45	+	5.85E-03	4.85E-02
↳nucleobase-containing small molecule metabolic process	202	13	4.58	2.84	+	1.08E-03	1.39E-02
↳small molecule metabolic process	587	29	13.31	2.18	+	1.43E-04	2.55E-03

↳metabolic process	4857	141	110.16	1.28	+	1.58E-03	1.83E-02
↳nucleobase-containing compound metabolic process	2112	27	47.90	.56	-	1.13E-03	1.43E-02
↳heterocycle metabolic process	2151	28	48.79	.57	-	1.25E-03	1.54E-02
↳cellular metabolic process	4357	131	98.82	1.33	+	5.47E-04	7.84E-03
↳organic cyclic compound metabolic process	2218	29	50.31	.58	-	1.12E-03	1.42E-02
↳organic substance metabolic process	4680	134	106.15	1.26	+	3.44E-03	3.37E-02
↳nitrogen compound metabolic process	4212	121	95.53	1.27	+	5.84E-03	4.86E-02
↳cellular aromatic compound metabolic process	2163	29	49.06	.59	-	2.17E-03	2.35E-02
↳purine ribonucleotide metabolic process	130	9	2.95	3.05	+	3.90E-03	3.71E-02
↳purine nucleotide metabolic process	138	9	3.13	2.88	+	5.62E-03	4.75E-02
↳purine-containing compound metabolic process	152	10	3.45	2.90	+	3.40E-03	3.35E-02
↳organonitrogen compound metabolic process	2527	110	57.32	1.92	+	4.94E-11	2.55E-08
↳ribonucleotide metabolic process	135	9	3.06	2.94	+	4.92E-03	4.35E-02
positive regulation of epithelial cell proliferation	8	3	.18	16.53	+	1.57E-03	1.83E-02
actin filament depolymerization	11	4	.25	16.03	+	2.69E-04	4.40E-03
↳actin polymerization or depolymerization	79	15	1.79	8.37	+	2.34E-09	4.03E-07
↳actin filament organization	159	23	3.61	6.38	+	1.83E-11	1.26E-08
↳actin cytoskeleton organization	238	26	5.40	4.82	+	2.51E-10	8.62E-08
↳cytoskeleton organization	560	36	12.70	2.83	+	6.54E-08	3.55E-06
↳actin filament-based process	246	26	5.58	4.66	+	4.80E-10	1.24E-07
↳protein-containing complex disassembly	62	6	1.41	4.27	+	3.96E-03	3.75E-02
glucose 6-phosphate metabolic process	9	3	.20	14.70	+	2.05E-03	2.27E-02
response to hypoxia	12	4	.27	14.70	+	3.52E-04	5.42E-03
↳response to stress	972	36	22.05	1.63	+	5.80E-03	4.85E-02
↳response to abiotic stimulus	86	7	1.95	3.59	+	4.65E-03	4.21E-02
Arp2/3 complex-mediated actin nucleation	18	6	.41	14.70	+	1.11E-05	2.97E-04
↳positive regulation of actin filament polymerization	34	6	.77	7.78	+	2.34E-04	3.86E-03
↳regulation of actin filament polymerization	71	13	1.61	8.07	+	4.01E-08	2.76E-06
↳regulation of protein polymerization	75	13	1.70	7.64	+	7.10E-08	3.76E-06
↳regulation of protein complex assembly	94	14	2.13	6.57	+	1.21E-07	5.43E-06
↳regulation of cellular component organization	396	21	8.98	2.34	+	5.35E-04	7.73E-03
↳regulation of cellular component biogenesis	140	15	3.18	4.72	+	1.99E-06	6.84E-05
↳regulation of actin polymerization or depolymerization	73	14	1.66	8.46	+	7.16E-09	8.70E-07
↳regulation of actin filament organization	81	14	1.84	7.62	+	2.30E-08	1.70E-06
↳regulation of actin cytoskeleton organization	106	15	2.40	6.24	+	7.86E-08	3.96E-06
↳regulation of actin filament-based process	107	15	2.43	6.18	+	8.79E-08	4.32E-06
↳regulation of cytoskeleton organization	146	17	3.31	5.13	+	1.42E-07	6.22E-06
↳regulation of organelle organization	264	18	5.99	3.01	+	6.52E-05	1.31E-03
↳regulation of supramolecular fiber organization	90	14	2.04	6.86	+	7.47E-08	3.85E-06
↳regulation of actin filament length	73	14	1.66	8.46	+	7.16E-09	9.24E-07
↳regulation of cellular component size	108	15	2.45	6.12	+	9.80E-08	4.71E-06
↳regulation of anatomical structure size	120	16	2.72	5.88	+	6.13E-08	3.52E-06
↳regulation of biological quality	905	38	20.53	1.85	+	4.03E-04	5.98E-03
↳positive regulation of supramolecular fiber organization	48	8	1.09	7.35	+	3.09E-05	7.16E-04
↳positive regulation of cellular component organization	136	12	3.08	3.89	+	1.19E-04	2.15E-03
↳positive regulation of cytoskeleton organization	49	8	1.11	7.20	+	3.52E-05	8.08E-04
↳positive regulation of organelle organization	79	9	1.79	5.02	+	1.46E-04	2.58E-03
↳positive regulation of protein polymerization	38	6	.86	6.96	+	3.99E-04	5.97E-03
↳positive regulation of protein complex assembly	43	7	.98	7.18	+	1.11E-04	2.02E-03
↳positive regulation of cellular component biogenesis	64	8	1.45	5.51	+	1.91E-04	3.34E-03
cellular response to oxidative stress	16	5	.36	13.78	+	7.98E-05	1.57E-03
↳cellular response to chemical stimulus	641	31	14.54	2.13	+	1.43E-04	2.57E-03
↳cellular response to stress	444	20	10.07	1.99	+	5.73E-03	4.83E-02

↳response to oxidative stress	25	7	.57	12.34	+	5.27E-06	1.65E-04
bone morphogenesis	14	4	.32	12.60	+	5.71E-04	8.08E-03
↳bone development	21	4	.48	8.40	+	2.09E-03	2.28E-02
↳animal organ development	349	17	7.92	2.15	+	3.81E-03	3.66E-02
↳animal organ morphogenesis	104	10	2.36	4.24	+	2.29E-04	3.84E-03
positive regulation of angiogenesis	11	3	.25	12.02	+	3.29E-03	3.33E-02
↳positive regulation of developmental process	109	8	2.47	3.24	+	4.59E-03	4.19E-02
epithelial cell proliferation	11	3	.25	12.02	+	3.29E-03	3.31E-02
protein stabilization	11	3	.25	12.02	+	3.29E-03	3.30E-02
↳regulation of protein stability	14	3	.32	9.45	+	5.85E-03	4.83E-02
response to reactive oxygen species	11	3	.25	12.02	+	3.29E-03	3.28E-02
glycolytic process	20	5	.45	11.02	+	1.94E-04	3.36E-03
↳generation of precursor metabolites and energy	103	12	2.34	5.14	+	9.68E-06	2.70E-04
↳nucleotide phosphorylation	20	5	.45	11.02	+	1.94E-04	3.33E-03
↳organic substance catabolic process	703	46	15.94	2.88	+	5.28E-10	1.21E-07
↳catabolic process	888	51	20.14	2.53	+	4.41E-09	6.06E-07
↳carbohydrate metabolic process	133	9	3.02	2.98	+	4.49E-03	4.14E-02
↳carboxylic acid metabolic process	331	19	7.51	2.53	+	3.37E-04	5.31E-03
↳oxoacid metabolic process	342	19	7.76	2.45	+	7.79E-04	1.06E-02
↳organic acid metabolic process	355	19	8.05	2.36	+	9.67E-04	1.29E-02
↳ATP metabolic process	56	6	1.27	4.72	+	2.48E-03	2.59E-02
chondrocyte differentiation	17	4	.39	10.37	+	1.06E-03	1.38E-02
↳cartilage development	18	4	.41	9.80	+	1.27E-03	1.55E-02
↳connective tissue development	18	4	.41	9.80	+	1.27E-03	1.55E-02
↳tissue development	179	15	4.06	3.69	+	3.03E-05	7.10E-04
regulation of leukocyte migration	17	4	.39	10.37	+	1.06E-03	1.38E-02
↳regulation of cell migration	79	8	1.79	4.46	+	6.98E-04	9.67E-03
↳regulation of cell motility	86	8	1.95	4.10	+	1.16E-03	1.44E-02
↳regulation of locomotion	91	8	2.06	3.88	+	1.63E-03	1.87E-02
↳regulation of cellular component movement	93	8	2.11	3.79	+	1.85E-03	2.07E-02
negative regulation of actin filament polymerization	23	5	.52	9.58	+	3.39E-04	5.30E-03
↳negative regulation of organelle organization	64	6	1.45	4.13	+	4.57E-03	4.19E-02
↳negative regulation of cellular component organization	88	8	2.00	4.01	+	1.33E-03	1.57E-02
↳negative regulation of cellular process	914	35	20.73	1.69	+	3.23E-03	3.28E-02
ribosomal large subunit assembly	23	5	.52	9.58	+	3.39E-04	5.26E-03
↳ribonucleoprotein complex assembly	110	8	2.49	3.21	+	4.83E-03	4.34E-02
↳cellular protein-containing complex assembly	367	24	8.32	2.88	+	8.01E-06	2.40E-04
↳protein-containing complex assembly	394	25	8.94	2.80	+	8.49E-06	2.50E-04
↳cellular component assembly	734	37	16.65	2.22	+	1.45E-05	3.80E-04
↳cellular component biogenesis	867	40	19.66	2.03	+	4.17E-05	9.16E-04
↳ribosomal large subunit biogenesis	63	6	1.43	4.20	+	4.26E-03	3.96E-02
↳ribosome biogenesis	176	11	3.99	2.76	+	3.15E-03	3.22E-02
chaperone-mediated protein folding	26	5	.59	8.48	+	5.55E-04	7.90E-03
↳protein folding	81	21	1.84	11.43	+	7.50E-15	1.55E-11
endoplasmic reticulum unfolded protein response	22	4	.50	8.02	+	2.43E-03	2.54E-02
↳response to endoplasmic reticulum stress	65	8	1.47	5.43	+	2.10E-04	3.59E-03
↳cellular response to unfolded protein	29	6	.66	9.12	+	1.09E-04	2.00E-03
↳response to unfolded protein	29	6	.66	9.12	+	1.09E-04	2.02E-03
↳response to topologically incorrect protein	41	7	.93	7.53	+	8.48E-05	1.64E-03
↳response to organic substance	558	27	12.66	2.13	+	4.83E-04	7.02E-03
↳cellular response to topologically incorrect protein	41	7	.93	7.53	+	8.48E-05	1.65E-03
↳cellular response to organic substance	488	24	11.07	2.17	+	6.35E-04	8.85E-03
actin filament polymerization	74	13	1.68	7.75	+	6.18E-08	3.45E-06
↳protein polymerization	89	13	2.02	6.44	+	4.17E-07	1.63E-05

granulocyte chemotaxis	38	6	.86	6.96	+	3.99E-04	6.01E-03
↳myeloid leukocyte migration	48	9	1.09	8.27	+	4.22E-06	1.38E-04
↳leukocyte migration	57	9	1.29	6.96	+	1.46E-05	3.76E-04
↳cell migration	255	25	5.78	4.32	+	4.25E-09	6.26E-07
↳cell motility	285	26	6.46	4.02	+	8.24E-09	8.51E-07
↳movement of cell or subcellular component	549	31	12.45	2.49	+	8.74E-06	2.54E-04
↳localization of cell	285	26	6.46	4.02	+	8.24E-09	8.95E-07
↳leukocyte chemotaxis	47	8	1.07	7.50	+	2.70E-05	6.40E-04
↳cell chemotaxis	81	8	1.84	4.35	+	8.12E-04	1.10E-02
response to toxic substance	32	5	.73	6.89	+	1.28E-03	1.54E-02
glucose metabolic process	26	4	.59	6.78	+	4.15E-03	3.87E-02
glutathione metabolic process	35	5	.79	6.30	+	1.82E-03	2.06E-02
↳peptide metabolic process	286	26	6.49	4.01	+	8.81E-09	8.66E-07
↳cellular amide metabolic process	369	26	8.37	3.11	+	9.30E-07	3.43E-05
↳cellular modified amino acid metabolic process	67	6	1.52	3.95	+	5.61E-03	4.77E-02
muscle contraction	43	6	.98	6.15	+	7.20E-04	9.84E-03
↳muscle system process	45	6	1.02	5.88	+	8.93E-04	1.20E-02
response to interleukin-1	29	4	.66	6.08	+	5.88E-03	4.83E-02
developmental growth	51	7	1.16	6.05	+	2.86E-04	4.62E-03
↳growth	55	7	1.25	5.61	+	4.34E-04	6.35E-03
negative regulation of endopeptidase activity	82	10	1.86	5.38	+	3.73E-05	8.29E-04
↳negative regulation of peptidase activity	87	10	1.97	5.07	+	5.90E-05	1.24E-03
↳negative regulation of hydrolase activity	102	11	2.31	4.75	+	4.35E-05	9.44E-04
↳regulation of hydrolase activity	287	15	6.51	2.30	+	4.33E-03	4.01E-02
↳negative regulation of catalytic activity	169	11	3.83	2.87	+	2.35E-03	2.51E-02
↳regulation of peptidase activity	112	11	2.54	4.33	+	9.45E-05	1.79E-03
↳regulation of proteolysis	155	13	3.52	3.70	+	1.00E-04	1.89E-03
↳regulation of protein metabolic process	581	26	13.18	1.97	+	1.75E-03	1.99E-02
↳negative regulation of proteolysis	88	10	2.00	5.01	+	6.44E-05	1.32E-03
↳negative regulation of cellular protein metabolic process	164	11	3.72	2.96	+	1.88E-03	2.10E-02
↳negative regulation of protein metabolic process	168	12	3.81	3.15	+	7.15E-04	9.84E-03
↳regulation of cellular protein metabolic process	533	24	12.09	1.99	+	1.95E-03	2.16E-02
↳regulation of endopeptidase activity	107	11	2.43	4.53	+	6.48E-05	1.31E-03
negative regulation of apoptotic process	41	5	.93	5.38	+	3.41E-03	3.35E-02
↳regulation of cell death	123	13	2.79	4.66	+	1.09E-05	3.00E-04
↳negative regulation of programmed cell death	42	5	.95	5.25	+	3.75E-03	3.61E-02
↳negative regulation of cell death	54	11	1.22	8.98	+	1.74E-07	7.18E-06
positive regulation of cell migration	43	5	.98	5.13	+	4.11E-03	3.87E-02
↳positive regulation of cell motility	47	5	1.07	4.69	+	5.80E-03	4.87E-02
carbohydrate biosynthetic process	43	5	.98	5.13	+	4.11E-03	3.85E-02
cell-substrate adhesion	45	5	1.02	4.90	+	4.90E-03	4.36E-02
↳cell adhesion	288	19	6.53	2.91	+	6.23E-05	1.30E-03
↳biological adhesion	288	19	6.53	2.91	+	6.23E-05	1.29E-03
response to inorganic substance	54	6	1.22	4.90	+	2.10E-03	2.28E-02
cellular transition metal ion homeostasis	46	5	1.04	4.79	+	5.34E-03	4.59E-02
↳transition metal ion homeostasis	55	6	1.25	4.81	+	2.29E-03	2.46E-02
cellular response to growth factor stimulus	90	9	2.04	4.41	+	3.58E-04	5.43E-03
↳response to growth factor	90	9	2.04	4.41	+	3.58E-04	5.47E-03
proteasome-mediated ubiquitin-dependent protein catabolic process	194	18	4.40	4.09	+	1.32E-06	4.61E-05
↳proteasomal protein catabolic process	205	18	4.65	3.87	+	2.72E-06	9.20E-05
↳proteolysis involved in cellular protein catabolic process	331	28	7.51	3.73	+	1.01E-08	9.46E-07
↳proteolysis	693	45	15.72	2.86	+	1.03E-09	1.93E-07
↳protein metabolic process	2115	87	47.97	1.81	+	1.06E-07	4.96E-06
↳cellular protein catabolic process	333	28	7.55	3.71	+	1.14E-08	1.02E-06

↳cellular macromolecule catabolic process	429	29	9.73	2.98	+	4.89E-07	1.87E-05
↳macromolecule catabolic process	463	33	10.50	3.14	+	2.45E-08	1.74E-06
↳cellular catabolic process	765	42	17.35	2.42	+	5.16E-07	1.94E-05
↳protein catabolic process	355	31	8.05	3.85	+	7.95E-10	1.64E-07
↳organonitrogen compound catabolic process	457	36	10.37	3.47	+	4.64E-10	1.37E-07
↳cellular protein metabolic process	1711	78	38.81	2.01	+	7.66E-09	8.79E-07
↳ubiquitin-dependent protein catabolic process	276	19	6.26	3.04	+	3.64E-05	8.26E-04
↳modification-dependent protein catabolic process	285	20	6.46	3.09	+	1.76E-05	4.44E-04
↳modification-dependent macromolecule catabolic process	290	20	6.58	3.04	+	2.23E-05	5.35E-04
oxidation-reduction process	220	18	4.99	3.61	+	6.77E-06	2.05E-04
cell death	186	15	4.22	3.56	+	4.56E-05	9.81E-04
translational elongation	216	17	4.90	3.47	+	1.94E-05	4.76E-04
↳translation	216	17	4.90	3.47	+	1.94E-05	4.70E-04
↳peptide biosynthetic process	219	18	4.97	3.62	+	6.38E-06	1.97E-04
↳organonitrogen compound biosynthetic process	589	28	13.36	2.10	+	4.04E-04	5.96E-03
↳amide biosynthetic process	252	18	5.72	3.15	+	3.71E-05	8.31E-04
cellular amino acid metabolic process	123	9	2.79	3.23	+	2.76E-03	2.85E-02
enzyme linked receptor protein signaling pathway	213	14	4.83	2.90	+	5.81E-04	8.16E-03
cell morphogenesis involved in differentiation	181	11	4.11	2.68	+	3.85E-03	3.68E-02
↳cell morphogenesis	255	15	5.78	2.59	+	1.10E-03	1.41E-02
↳cellular component morphogenesis	281	16	6.37	2.51	+	1.05E-03	1.38E-02
Unclassified	12808	231	290.50	.80	-	1.52E-07	6.54E-06
organic substance transport	801	6	18.17	.33	-	2.07E-03	2.27E-02
↳establishment of localization	1847	25	41.89	.60	-	5.21E-03	4.54E-02
ion transport	599	4	13.59	.29	-	4.63E-03	4.21E-02
G protein-coupled receptor signaling pathway	682	3	15.47	.19	-	2.95E-04	4.72E-03
regulation of transcription by RNA polymerase II	745	3	16.90	.18	-	7.18E-05	1.43E-03
↳regulation of transcription, DNA-templated	1117	3	25.34	.12	-	5.36E-08	3.45E-06
↳regulation of gene expression	1368	9	31.03	.29	-	4.54E-06	1.46E-04
↳regulation of nucleic acid-templated transcription	1117	3	25.34	.12	-	5.36E-08	3.35E-06
↳regulation of RNA biosynthetic process	1117	3	25.34	.12	-	5.36E-08	3.25E-06
↳regulation of RNA metabolic process	1222	5	27.72	.18	-	2.22E-07	8.98E-06
↳regulation of nucleobase-containing compound metabolic process	1263	5	28.65	.17	-	1.11E-07	5.10E-06
↳regulation of cellular biosynthetic process	1234	8	27.99	.29	-	1.56E-05	3.97E-04
↳regulation of biosynthetic process	1240	8	28.12	.28	-	1.10E-05	2.99E-04
↳regulation of macromolecule biosynthetic process	1223	6	27.74	.22	-	1.16E-06	4.11E-05
↳regulation of cellular macromolecule biosynthetic process	1216	6	27.58	.22	-	1.13E-06	4.09E-05
transcription by RNA polymerase II	772	3	17.51	.17	-	5.18E-05	1.10E-03
↳transcription, DNA-templated	1159	3	26.29	.11	-	1.65E-08	1.31E-06
↳nucleic acid-templated transcription	1159	3	26.29	.11	-	1.65E-08	1.36E-06
↳RNA biosynthetic process	1166	3	26.45	.11	-	1.68E-08	1.29E-06
↳nucleobase-containing compound biosynthetic process	1376	10	31.21	.32	-	1.25E-05	3.30E-04
↳aromatic compound biosynthetic process	1399	10	31.73	.32	-	9.12E-06	2.58E-04
↳organic cyclic compound biosynthetic process	1433	10	32.50	.31	-	4.84E-06	1.54E-04
↳heterocycle biosynthetic process	1399	10	31.73	.32	-	9.12E-06	2.61E-04
↳RNA metabolic process	1673	13	37.95	.34	-	2.86E-06	9.51E-05
↳nucleic acid metabolic process	1911	14	43.34	.32	-	2.44E-07	9.69E-06
positive regulation of transcription, DNA-templated	421	1	9.55	.10	-	1.29E-03	1.54E-02
↳positive regulation of nucleic acid-templated transcription	421	1	9.55	.10	-	1.29E-03	1.55E-02
↳positive regulation of RNA biosynthetic process	421	1	9.55	.10	-	1.29E-03	1.54E-02
↳positive regulation of macromolecule biosynthetic process	451	2	10.23	.20	-	5.26E-03	4.56E-02
↳positive regulation of RNA metabolic process	444	2	10.07	.20	-	5.11E-03	4.50E-02
↳positive regulation of nucleobase-containing compound metabolic process	461	2	10.46	.19	-	3.61E-03	3.52E-02
immune response-activating signal transduction	267	0	6.06	< 0.01	-	5.12E-03	4.50E-02

↳immune response-regulating signaling pathway	269	0	6.10	< 0.01	-	5.20E-03	4.55E-02
↳activation of immune response	278	0	6.31	< 0.01	-	3.35E-03	3.32E-02
negative regulation of transcription, DNA-templated	305	0	6.92	< 0.01	-	2.40E-03	2.54E-02
↳negative regulation of nucleic acid-templated transcription	305	0	6.92	< 0.01	-	2.40E-03	2.56E-02
↳negative regulation of RNA biosynthetic process	305	0	6.92	< 0.01	-	2.40E-03	2.53E-02
↳negative regulation of RNA metabolic process	316	0	7.17	< 0.01	-	1.55E-03	1.82E-02
↳negative regulation of nucleobase-containing compound metabolic process	329	0	7.46	< 0.01	-	1.03E-03	1.36E-02
↳negative regulation of cellular biosynthetic process	354	1	8.03	.12	-	5.61E-03	4.79E-02
↳negative regulation of biosynthetic process	354	1	8.03	.12	-	5.61E-03	4.81E-02
↳negative regulation of gene expression	409	0	9.28	< 0.01	-	2.30E-04	3.82E-03

© Copyright 2020 Paul Thomas All Rights Reserved.

Analysis Summary: Please report in publication

BM-MSC secretome from HFD mice

Analysis Type: PANTHER Overrepresentation Test (Released 20190711)

Annotation Version and Release Date: PANTHER version 15 Released 2020-02-14

Analyzed List: Client Text Box Input (Mus musculus) Change

Reference List: Mus musculus (all genes in database) Change

Annotation Data Set: PANTHER GO-Slim Biological Process

Test Type: Fisher's Exact Binomial

Correction: Calculate False Discovery Rate Use the Bonferroni correction for multiple testing No correction

Results

	Reference list	Client Text Box Input
Uniquely Mapped IDS:	22265 out of 22265	438 out of 438
Unmapped IDs:	0	3
Multiple mapping information:	0	9

Export Table XML with user input ids JSON with user input ids View: -- Please select a chart to display --

Displaying only results for FDR P < 0.05, click here to display all results

	Mus musculus (REF)	Client Text Box Input (Hierarchy NEW!)					
PANTHER GO-Slim Biological Process	#	#	expected	Fold Enrichment	+/-	raw P value	FDR
tissue remodeling	3	2	.06	33.89	+	3.57E-03	4.21E-02
collagen fibril organization	11	6	.22	27.73	+	5.15E-07	3.43E-05
↳supramolecular fiber organization	236	28	4.64	6.03	+	3.03E-13	6.26E-10
↳cellular process	7925	210	155.90	1.35	+	1.90E-07	1.45E-05
↳extracellular matrix organization	56	15	1.10	13.62	+	5.13E-12	5.30E-09
↳extracellular structure organization	63	15	1.24	12.10	+	2.17E-11	1.49E-08
↳protein-containing complex subunit organization	481	23	9.46	2.43	+	1.86E-04	3.59E-03
bone morphogenesis	14	6	.28	21.79	+	1.54E-06	8.80E-05
↳bone development	21	6	.41	14.52	+	1.05E-05	3.17E-04
↳skeletal system development	54	7	1.06	6.59	+	1.68E-04	3.31E-03
↳animal organ development	349	20	6.87	2.91	+	3.69E-05	8.96E-04
↳animal organ morphogenesis	104	11	2.05	5.38	+	1.43E-05	4.28E-04
↳anatomical structure morphogenesis	450	21	8.85	2.37	+	4.56E-04	7.85E-03
chondrocyte differentiation	17	6	.33	17.94	+	3.81E-06	1.60E-04
↳cartilage development	18	6	.35	16.94	+	4.99E-06	1.84E-04
↳connective tissue development	18	6	.35	16.94	+	4.99E-06	1.87E-04
↳tissue development	179	16	3.52	4.54	+	1.33E-06	8.07E-05
glucose 6-phosphate metabolic process	9	3	.18	16.94	+	1.38E-03	1.98E-02
cellular response to oxidative stress	16	5	.31	15.89	+	4.12E-05	9.88E-04
↳cellular response to chemical stimulus	641	27	12.61	2.14	+	2.97E-04	5.32E-03
↳response to chemical	915	35	18.00	1.94	+	2.55E-04	4.65E-03
↳response to stress	972	35	19.12	1.83	+	8.88E-04	1.36E-02
↳response to oxidative stress	25	7	.49	14.23	+	2.10E-06	1.06E-04

Arp2/3 complex-mediated actin nucleation	18	5	.35	14.12	+	6.60E-05	1.50E-03
↳positive regulation of actin filament polymerization	34	5	.67	7.48	+	8.76E-04	1.36E-02
↳regulation of actin filament polymerization	71	9	1.40	6.44	+	2.36E-05	6.48E-04
↳regulation of protein polymerization	75	9	1.48	6.10	+	3.49E-05	8.69E-04
↳regulation of protein complex assembly	94	9	1.85	4.87	+	1.73E-04	3.36E-03
↳regulation of cellular component biogenesis	140	10	2.75	3.63	+	6.85E-04	1.11E-02
↳regulation of actin polymerization or depolymerization	73	10	1.44	6.96	+	4.47E-06	1.81E-04
↳regulation of actin filament organization	81	10	1.59	6.28	+	1.03E-05	3.17E-04
↳regulation of actin cytoskeleton organization	106	10	2.09	4.80	+	8.52E-05	1.89E-03
↳regulation of actin filament-based process	107	10	2.10	4.75	+	9.15E-05	1.97E-03
↳regulation of cytoskeleton organization	146	11	2.87	3.83	+	2.44E-04	4.50E-03
↳regulation of supramolecular fiber organization	90	10	1.77	5.65	+	2.38E-05	6.45E-04
↳regulation of actin filament length	73	10	1.44	6.96	+	4.47E-06	1.84E-04
↳regulation of cellular component size	108	11	2.12	5.18	+	1.98E-05	5.52E-04
↳regulation of anatomical structure size	120	12	2.36	5.08	+	9.90E-06	3.10E-04
↳regulation of biological quality	905	32	17.80	1.80	+	2.13E-03	2.94E-02
↳positive regulation of supramolecular fiber organization	48	7	.94	7.41	+	8.65E-05	1.90E-03
↳positive regulation of cellular component organization	136	10	2.68	3.74	+	5.54E-04	9.46E-03
↳positive regulation of cytoskeleton organization	49	7	.96	7.26	+	9.73E-05	2.05E-03
↳positive regulation of organelle organization	79	9	1.55	5.79	+	5.07E-05	1.19E-03
↳positive regulation of protein polymerization	38	5	.75	6.69	+	1.38E-03	1.98E-02
↳positive regulation of protein complex assembly	43	5	.85	5.91	+	2.26E-03	2.95E-02
↳positive regulation of cellular component biogenesis	64	6	1.26	4.77	+	2.31E-03	2.99E-02
↳actin filament organization	159	19	3.13	6.07	+	1.87E-09	2.76E-07
↳actin cytoskeleton organization	238	22	4.68	4.70	+	7.97E-09	9.14E-07
↳cytoskeleton organization	560	26	11.02	2.36	+	9.04E-05	1.96E-03
↳actin filament-based process	246	22	4.84	4.55	+	1.39E-08	1.51E-06
response to reactive oxygen species	11	3	.22	13.86	+	2.22E-03	2.97E-02
granulocyte chemotaxis	38	10	.75	13.38	+	2.19E-08	2.15E-06
↳myeloid leukocyte migration	48	11	.94	11.65	+	1.48E-08	1.53E-06
↳leukocyte migration	57	12	1.12	10.70	+	7.47E-09	9.07E-07
↳cell migration	255	26	5.02	5.18	+	4.81E-11	1.98E-08
↳cell motility	285	27	5.61	4.82	+	9.37E-11	2.42E-08
↳movement of cell or subcellular component	549	31	10.80	2.87	+	3.74E-07	2.58E-05
↳locomotion	356	29	7.00	4.14	+	5.07E-10	9.52E-08
↳localization of cell	285	27	5.61	4.82	+	9.37E-11	2.76E-08
↳leukocyte chemotaxis	47	12	.92	12.98	+	1.13E-09	1.79E-07
↳cell chemotaxis	81	12	1.59	7.53	+	2.32E-07	1.71E-05
↳chemotaxis	186	15	3.66	4.10	+	9.06E-06	2.88E-04
↳taxis	186	15	3.66	4.10	+	9.06E-06	2.92E-04
glycolytic process	20	5	.39	12.71	+	1.01E-04	2.10E-03
↳generation of precursor metabolites and energy	103	9	2.03	4.44	+	3.25E-04	5.78E-03
↳nucleotide phosphorylation	20	5	.39	12.71	+	1.01E-04	2.08E-03
↳organic substance catabolic process	703	43	13.83	3.11	+	1.85E-10	4.25E-08
↳catabolic process	888	49	17.47	2.80	+	2.65E-10	5.47E-08
↳cellular aromatic compound metabolic process	2163	23	42.55	.54	-	1.01E-03	1.53E-02
↳organic cyclic compound metabolic process	2218	23	43.63	.53	-	6.32E-04	1.04E-02
↳organonitrogen compound metabolic process	2527	95	49.71	1.91	+	9.28E-10	1.60E-07
↳heterocycle metabolic process	2151	23	42.31	.54	-	1.30E-03	1.91E-02
↳nucleobase-containing compound metabolic process	2112	22	41.55	.53	-	8.87E-04	1.37E-02
chemokine-mediated signaling pathway	35	8	.69	11.62	+	1.44E-06	8.52E-05

↳cellular response to chemokine	36	8	.71	11.30	+	1.74E-06	9.68E-05
↳response to chemokine	36	8	.71	11.30	+	1.74E-06	9.43E-05
↳response to cytokine	162	11	3.19	3.45	+	5.59E-04	9.46E-03
↳response to organic substance	558	24	10.98	2.19	+	5.71E-04	9.59E-03
↳cellular response to organic substance	488	20	9.60	2.08	+	2.72E-03	3.41E-02
↳cytokine-mediated signaling pathway	119	8	2.34	3.42	+	3.28E-03	3.96E-02
membrane protein proteolysis	14	3	.28	10.89	+	3.97E-03	4.55E-02
↳proteolysis	693	44	13.63	3.23	+	3.62E-11	1.87E-08
↳protein metabolic process	2115	78	41.61	1.87	+	1.16E-07	9.54E-06
response to interleukin-1	29	6	.57	10.52	+	5.03E-05	1.19E-03
lymphocyte migration	30	6	.59	10.17	+	5.94E-05	1.36E-03
positive regulation of ERK1 and ERK2 cascade	37	6	.73	8.24	+	1.66E-04	3.33E-03
↳regulation of cellular protein metabolic process	533	21	10.49	2.00	+	4.06E-03	4.61E-02
↳regulation of protein metabolic process	581	23	11.43	2.01	+	2.23E-03	2.97E-02
↳regulation of ERK1 and ERK2 cascade	38	6	.75	8.03	+	1.89E-04	3.61E-03
ERK1 and ERK2 cascade	38	6	.75	8.03	+	1.89E-04	3.58E-03
↳cellular protein metabolic process	1711	65	33.66	1.93	+	5.45E-07	3.52E-05
developmental growth	51	8	1.00	7.97	+	1.69E-05	4.84E-04
↳growth	55	8	1.08	7.39	+	2.75E-05	7.29E-04
glucose metabolic process	26	4	.51	7.82	+	2.52E-03	3.21E-02
chaperone-mediated protein folding	26	4	.51	7.82	+	2.52E-03	3.19E-02
↳protein folding	81	16	1.59	10.04	+	5.33E-11	1.83E-08
cellular response to tumor necrosis factor	35	5	.69	7.26	+	9.86E-04	1.50E-02
↳response to tumor necrosis factor	39	5	.77	6.52	+	1.53E-03	2.17E-02
muscle contraction	43	6	.85	7.09	+	3.45E-04	6.09E-03
↳muscle system process	45	6	.89	6.78	+	4.31E-04	7.47E-03
negative regulation of endopeptidase activity	82	11	1.61	6.82	+	1.79E-06	9.48E-05
↳negative regulation of peptidase activity	87	11	1.71	6.43	+	3.02E-06	1.35E-04
↳negative regulation of hydrolase activity	102	12	2.01	5.98	+	2.14E-06	1.05E-04
↳regulation of hydrolase activity	287	17	5.65	3.01	+	9.59E-05	2.04E-03
↳negative regulation of catalytic activity	169	12	3.32	3.61	+	2.17E-04	4.03E-03
↳negative regulation of molecular function	196	12	3.86	3.11	+	7.58E-04	1.21E-02
↳regulation of peptidase activity	112	11	2.20	4.99	+	2.70E-05	7.24E-04
↳regulation of proteolysis	155	12	3.05	3.94	+	1.02E-04	2.08E-03
↳negative regulation of proteolysis	88	11	1.73	6.35	+	3.34E-06	1.47E-04
↳negative regulation of cellular protein metabolic process	164	12	3.23	3.72	+	1.67E-04	3.31E-03
↳negative regulation of protein metabolic process	168	13	3.30	3.93	+	5.30E-05	1.23E-03
↳regulation of endopeptidase activity	107	11	2.10	5.23	+	1.83E-05	5.17E-04
regulation of cellular response to growth factor stimulus	30	4	.59	6.78	+	4.01E-03	4.57E-02
actin filament bundle assembly	31	4	.61	6.56	+	4.46E-03	4.97E-02
actin filament polymerization	74	9	1.46	6.18	+	3.17E-05	8.19E-04
↳protein polymerization	89	9	1.75	5.14	+	1.18E-04	2.38E-03
↳actin polymerization or depolymerization	79	11	1.55	7.08	+	1.29E-06	8.05E-05
tissue morphogenesis	43	5	.85	5.91	+	2.26E-03	2.99E-02
positive regulation of cell migration	43	5	.85	5.91	+	2.26E-03	2.97E-02
↳regulation of cell migration	79	7	1.55	4.50	+	1.37E-03	2.00E-02
↳regulation of cell motility	86	7	1.69	4.14	+	2.16E-03	2.93E-02
↳regulation of locomotion	91	7	1.79	3.91	+	2.91E-03	3.57E-02
↳regulation of cellular component movement	93	7	1.83	3.83	+	3.26E-03	3.95E-02
↳positive regulation of cell motility	47	5	.92	5.41	+	3.22E-03	3.94E-02
↳positive regulation of cellular component movement	48	5	.94	5.30	+	3.50E-03	4.18E-02
↳positive regulation of locomotion	48	5	.94	5.30	+	3.50E-03	4.16E-02

negative regulation of cell death	54	6	1.06	5.65	+	1.03E-03	1.54E-02
↳regulation of cell death	123	8	2.42	3.31	+	3.96E-03	4.57E-02
cell-substrate adhesion	45	5	.89	5.65	+	2.71E-03	3.41E-02
↳cell adhesion	288	15	5.67	2.65	+	8.68E-04	1.37E-02
↳biological adhesion	288	15	5.67	2.65	+	8.68E-04	1.36E-02
inflammatory response	115	12	2.26	5.30	+	6.65E-06	2.25E-04
response to endoplasmic reticulum stress	65	6	1.28	4.69	+	2.48E-03	3.20E-02
peptidyl-lysine modification	91	7	1.79	3.91	+	2.91E-03	3.59E-02
cell morphogenesis involved in differentiation	181	12	3.56	3.37	+	3.90E-04	6.81E-03
↳cell morphogenesis	255	14	5.02	2.79	+	7.90E-04	1.25E-02
↳cellular component morphogenesis	281	15	5.53	2.71	+	6.84E-04	1.12E-02
oxidation-reduction process	220	14	4.33	3.23	+	1.95E-04	3.67E-03
proteasome-mediated ubiquitin-dependent protein catabolic process	194	12	3.82	3.14	+	6.97E-04	1.12E-02
↳proteasomal protein catabolic process	205	12	4.03	2.98	+	1.10E-03	1.62E-02
↳proteolysis involved in cellular protein catabolic process	331	25	6.51	3.84	+	3.27E-08	3.06E-06
↳cellular protein catabolic process	333	25	6.55	3.82	+	3.64E-08	3.13E-06
↳cellular macromolecule catabolic process	429	27	8.44	3.20	+	2.99E-07	2.13E-05
↳macromolecule catabolic process	463	30	9.11	3.29	+	3.55E-08	3.18E-06
↳cellular catabolic process	765	39	15.05	2.59	+	1.44E-07	1.14E-05
↳protein catabolic process	355	28	6.98	4.01	+	1.98E-09	2.73E-07
↳organonitrogen compound catabolic process	457	32	8.99	3.56	+	2.14E-09	2.76E-07
↳ubiquitin-dependent protein catabolic process	276	13	5.43	2.39	+	4.26E-03	4.81E-02
↳modification-dependent protein catabolic process	285	14	5.61	2.50	+	2.14E-03	2.93E-02
↳modification-dependent macromolecule catabolic process	290	14	5.70	2.45	+	2.49E-03	3.19E-02
enzyme linked receptor protein signaling pathway	213	11	4.19	2.63	+	4.34E-03	4.87E-02
peptide metabolic process	286	14	5.63	2.49	+	2.21E-03	2.98E-02
Unclassified	12808	208	251.96	.83	-	3.21E-05	8.19E-04
transport	1822	18	35.84	.50	-	1.03E-03	1.53E-02
↳establishment of localization	1847	19	36.33	.52	-	1.98E-03	2.80E-02
regulation of transcription by RNA polymerase II	745	3	14.66	.20	-	5.83E-04	9.71E-03
↳regulation of transcription, DNA-templated	1117	4	21.97	.18	-	5.63E-06	2.00E-04
↳regulation of gene expression	1368	7	26.91	.26	-	6.75E-06	2.25E-04
↳regulation of nucleic acid-templated transcription	1117	4	21.97	.18	-	5.63E-06	1.97E-04
↳regulation of RNA biosynthetic process	1117	4	21.97	.18	-	5.63E-06	1.94E-04
↳regulation of RNA metabolic process	1222	7	24.04	.29	-	6.67E-05	1.50E-03
↳regulation of nucleobase-containing compound metabolic process	1263	7	24.85	.28	-	3.50E-05	8.60E-04
↳regulation of cellular biosynthetic process	1234	6	24.28	.25	-	1.64E-05	4.82E-04
↳regulation of biosynthetic process	1240	6	24.39	.25	-	1.67E-05	4.85E-04
↳regulation of macromolecule biosynthetic process	1223	5	24.06	.21	-	5.08E-06	1.84E-04
↳regulation of cellular macromolecule biosynthetic process	1216	5	23.92	.21	-	4.95E-06	1.89E-04
transcription by RNA polymerase II	772	3	15.19	.20	-	2.84E-04	5.14E-03
↳transcription, DNA-templated	1159	4	22.80	.18	-	2.77E-06	1.27E-04
↳nucleic acid-templated transcription	1159	4	22.80	.18	-	2.77E-06	1.30E-04
↳RNA biosynthetic process	1166	4	22.94	.17	-	1.89E-06	9.76E-05
↳macromolecule biosynthetic process	1636	16	32.18	.50	-	2.04E-03	2.85E-02
↳nucleobase-containing compound biosynthetic process	1376	7	27.07	.26	-	6.78E-06	2.22E-04
↳cellular nitrogen compound biosynthetic process	1644	16	32.34	.49	-	2.07E-03	2.87E-02
↳aromatic compound biosynthetic process	1399	7	27.52	.25	-	4.87E-06	1.90E-04
↳organic cyclic compound biosynthetic process	1433	7	28.19	.25	-	2.46E-06	1.18E-04
↳heterocycle biosynthetic process	1399	7	27.52	.25	-	4.87E-06	1.93E-04
↳RNA metabolic process	1673	12	32.91	.36	-	3.01E-05	7.88E-04
↳nucleic acid metabolic process	1911	13	37.59	.35	-	3.51E-06	1.51E-04

↳cellular macromolecule biosynthetic process	1628	16	32.03	.50	-	2.03E-03	2.85E-02
positive regulation of transcription, DNA-templated	421	1	8.28	.12	-	3.89E-03	4.53E-02
↳positive regulation of nucleic acid-templated transcription	421	1	8.28	.12	-	3.89E-03	4.56E-02
↳positive regulation of RNA biosynthetic process	421	1	8.28	.12	-	3.89E-03	4.51E-02
↳positive regulation of macromolecule biosynthetic process	451	1	8.87	.11	-	2.74E-03	3.41E-02
nervous system process	320	0	6.30	< 0.01	-	3.37E-03	4.04E-02

© Copyright 2020 Paul Thomas All Rights Reserved.

Analysis Summary: Please report in publication

vWAT-MSC secretome from HFD mice

Analysis Type: PANTHER Overrepresentation Test (Released 20190711)

Annotation Version and Release Date: PANTHER version 15 Released 2020-02-14

Analyzed List: Client Text Box Input (Mus musculus) Change

Reference List: Mus musculus (all genes in database) Change

Annotation Data Set: PANTHER Pathways

Test Type: (•) Fisher's Exact ( ) Binomial

Correction: (•) Calculate False Discovery Rate ( ) Use the Bonferroni correction for multiple testing ( ) No correction

Results

	Reference list	Client Text Box Input
Uniquely Mapped IDS:	22265 out of 22265	377 out of 378
Unmapped IDs:	0	3
Multiple mapping information:	0	10

Export Table XML with user input ids JSON with user input ids View: -- Please select a chart to display --

Displaying only results for FDR P < 0.05, click here to display all results

	Mus musculus (REF)	Client Text Box Input					
PANTHER Pathways	#	#	expected	Fold Enrichment	+/-	raw P value	FDR
Pentose phosphate pathway	9	4	.15	26.18	+	4.85E-05	1.34E-03
Glycolysis	26	8	.44	18.12	+	6.96E-08	2.31E-06
Parkinson disease	95	18	1.61	11.16	+	5.39E-13	2.98E-11
De novo purine biosynthesis	29	5	.49	10.16	+	2.36E-04	4.89E-03
Cytoskeletal regulation by Rho GTPase	79	13	1.34	9.69	+	4.33E-09	1.80E-07
Integrin signalling pathway	190	24	3.23	7.44	+	2.12E-13	1.76E-11
Huntington disease	150	11	2.55	4.32	+	8.66E-05	2.05E-03
Inflammation mediated by chemokine and cytokine signaling pathway	258	13	4.38	2.97	+	6.79E-04	1.25E-02
Unclassified	19669	266	333.93	.80	-	1.51E-20	2.51E-18

© Copyright 2020 Paul Thomas All Rights Reserved.

PANTHER 15.0 released!

Analysis Summary: Please report in publication sWAT-MSC secretome from HFD mice

Analysis Type: PANTHER Overrepresentation Test (Released 20190711)

Annotation Version and Release Date: PANTHER version 15 Released 2020-02-14

Analyzed List: Client Text Box Input (Mus musculus) Change

Reference List: Mus musculus (all genes in database) Change

Annotation Data Set: PANTHER Pathways

Test Type: Fisher's Exact Binomial

Correction: Calculate False Discovery Rate Use the Bonferroni correction for multiple testing No correction

Results

	Reference list	Client Text Box Input
Uniquely Mapped IDS:	22265 out of 22265	506 out of 505
Unmapped IDs:	0	4
Multiple mapping information:	0	8

Export Table XML with user input ids JSON with user input ids View: -- Please select a chart to display --

Displaying only results for FDR P < 0.05, click here to display all results

	Mus musculus (REF)	Client Text Box Input					
PANTHER Pathways	#	#	expected	Fold Enrichment	+/-	raw P value	FDR
Pentose phosphate pathway	9	4	.20	19.60	+	1.46E-04	2.20E-03
Glycolysis	26	8	.59	13.57	+	6.05E-07	2.01E-05
Plasminogen activating cascade	17	4	.39	10.37	+	1.06E-03	1.35E-02
Parkinson disease	95	21	2.15	9.75	+	1.11E-13	6.17E-12
De novo purine biosynthesis	29	6	.66	9.12	+	1.09E-04	1.80E-03
Cytoskeletal regulation by Rho GTPase	79	16	1.79	8.93	+	2.98E-10	1.24E-08
Blood coagulation	51	9	1.16	7.78	+	6.54E-06	1.81E-04
Axon guidance mediated by semaphorins	23	4	.52	7.67	+	2.80E-03	2.90E-02
Integrin signalling pathway	190	33	4.31	7.66	+	5.64E-18	4.68E-16
Axon guidance mediated by Slit/Robo	24	4	.54	7.35	+	3.21E-03	3.13E-02
FGF signaling pathway	120	12	2.72	4.41	+	3.90E-05	8.10E-04
Huntington disease	150	14	3.40	4.11	+	1.85E-05	4.38E-04
EGF receptor signaling pathway	136	11	3.08	3.57	+	4.51E-04	6.24E-03
Inflammation mediated by chemokine and cytokine signaling pathway	258	18	5.85	3.08	+	4.94E-05	9.10E-04
CCKR signaling map	159	11	3.61	3.05	+	1.50E-03	1.66E-02
Gonadotropin-releasing hormone receptor pathway	236	14	5.35	2.62	+	1.47E-03	1.75E-02
Unclassified	19669	353	446.12	.79	-	7.17E-28	1.19E-25

© Copyright 2020 Paul Thomas All Rights Reserved.

PANTHER 15.0 released!

Analysis Summary: Please report in publication

BM-MSC secretome from HFD mice

Analysis Type: PANTHER Overrepresentation Test (Released 20190711)

Annotation Version and Release Date: PANTHER version 15 Released 2020-02-14

Analyzed List: Client Text Box Input (Mus musculus) Change

Reference List: Mus musculus (all genes in database) Change

Annotation Data Set: PANTHER Pathways

Test Type: (•) Fisher's Exact ( ) Binomial

Correction: (•) Calculate False Discovery Rate ( ) Use the Bonferroni correction for multiple testing ( ) No correction

Results

	Reference list	Client Text Box Input
Uniquely Mapped IDS:	22265 out of 22265	438 out of 438
Unmapped IDs:	0	3
Multiple mapping information:	0	9

Export Table XML with user input ids JSON with user input ids View: -- Please select a chart to display --

Displaying only results for FDR P < 0.05, click here to display all results

	Mus musculus (REF)	Client Text Box Input					
PANTHER Pathways	#	#	expected	Fold Enrichment	+/-	raw P value	FDR
Pentose phosphate pathway	9	4	.18	22.59	+	8.51E-05	1.57E-03
Glycolysis	26	8	.51	15.64	+	2.11E-07	8.74E-06
Plasminogen activating cascade	17	5	.33	14.95	+	5.25E-05	1.09E-03
Parkinson disease	95	17	1.87	9.10	+	5.24E-11	2.90E-09
Blood coagulation	51	9	1.00	8.97	+	2.11E-06	5.84E-05
Integrin signalling pathway	190	30	3.74	8.03	+	4.22E-17	3.50E-15
Cytoskeletal regulation by Rho GTPase	79	11	1.55	7.08	+	1.29E-06	4.27E-05
Alzheimer disease-presenilin pathway	125	9	2.46	3.66	+	1.19E-03	1.98E-02
Inflammation mediated by chemokine and cytokine signaling pathway	258	17	5.08	3.35	+	2.76E-05	6.55E-04
Unclassified	19669	314	386.93	.81	-	1.07E-20	1.78E-18

© Copyright 2020 Paul Thomas All Rights Reserved.
